# A Randomized, Single-Ascending-Dose, Ivermectin-Controlled, Double-Blind Study of Moxidectin in *Onchocerca volvulus* Infection

**DOI:** 10.1371/journal.pntd.0002953

**Published:** 2014-06-26

**Authors:** Kwablah Awadzi, Nicholas O. Opoku, Simon K. Attah, Janis Lazdins-Helds, Annette C. Kuesel

**Affiliations:** 1 Onchocerciasis Chemotherapy Research Centre, Hohoe, Ghana; 2 University of Ghana Medical School, Department of Microbiology, Accra, Ghana; 3 UNICEF/UNDP/World Bank/World Health Organization Special Programme for Research and Training in Tropical Diseases, World Health Organization, Geneva, Switzerland; University Clinic Bonn, Germany

## Abstract

**Background:**

Control of onchocerciasis as a public health problem in Africa relies on annual mass ivermectin distribution. New tools are needed to achieve elimination of infection. This study determined in a small number of *Onchocerca volvulus* infected individuals whether moxidectin, a veterinary anthelminthic, is safe enough to administer it in a future large study to further characterize moxidectin's safety and efficacy. Effects on the parasite were also assessed.

**Methodology/Principal Findings:**

Men and women from a forest area in South-eastern Ghana without ivermectin mass distribution received a single oral dose of 2 mg (N = 44), 4 mg (N = 45) or 8 mg (N = 38) moxidectin or 150 µg/kg ivermectin (N = 45) with 18 months follow up. All ivermectin and 97%–100% of moxidectin treated participants had Mazzotti reactions. Statistically significantly higher percentages of participants treated with 8 mg moxidectin than participants treated with ivermectin experienced pruritus (87% vs. 56%), rash (63% vs. 42%), increased pulse rate (61% vs. 36%) and decreased mean arterial pressure upon 2 minutes standing still after ≥5 minutes supine relative to pre-treatment (61% vs. 27%). These reactions resolved without treatment. In the 8 mg moxidectin and ivermectin arms, the mean±SD number of microfilariae/mg skin were 22.9±21.1 and 21.2±16.4 pre-treatment and 0.0±0.0 and 1.1±4.2 at nadir reached 1 and 3 months after treatment, respectively. At 6 months, values were 0.0±0.0 and 1.6±4.5, at 12 months 0.4±0.9 and 3.4±4.4 and at 18 months 1.8±3.3 and 4.0±4.8, respectively, in the 8 mg moxidectin and ivermectin arm. The reduction from pre-treatment values was significantly higher after 8 mg moxidectin than after ivermectin treatment throughout follow up (p<0.01).

**Conclusions/Significance:**

The 8 mg dose of moxidectin was safe enough to initiate the large study. Provided its results confirm those from this study, availability of moxidectin to control programmes could help them achieve onchocerciasis elimination objectives.

**Trial Registration:**

ClinicalTrails.gov NCT00300768

## Author Summary

Around 100 million Africans live in onchocerciasis endemic areas. Control of onchocerciasis as a public health problem and possibly even elimination of onchocerciasis infection relies on annual community-directed mass treatment with ivermectin. Given concerns about possible emergence of ivermectin resistance of the parasite *Onchocerca volvulus* and elimination of infection in areas where very high numbers of vectors can result in continued parasite transmission even when only few parasites are present in only a few people, research for drugs with higher effect on the parasite remains important. A series of non-clinical and clinical studies was planned to find out whether moxidectin, a veterinary anthelminthic, is sufficiently safe for mass treatment and has a better effect on the parasite than ivermectin. We report here results from the first study in infected people conducted to assess in small numbers of individuals the adverse reactions to the killing of parasites by moxidectin. A single dose of 8 mg moxidectin reduced skin parasite numbers better and for a longer time than ivermectin. The frequency and severity of adverse reactions was so low that a larger study to better characterize the adverse reactions to moxidectin and compare its efficacy with that of ivermectin was initiated.

## Introduction

Onchocerciasis is caused by the filarial nematode *Onchocerca volvulus* and is transmitted among humans through the bites of blackfly vectors, in Africa mainly by *Simulium damnosum s.l.*. Around 99% of people at risk live in Sub-saharan Africa. The African Programme for Onchocerciasis Control (APOC) estimated 89 million Africans at risk and 37 million infected in 19 APOC countries [1] based on rapid epidemiological mapping [2].

Since its launch in 1995, APOC and the public health systems have established annual community-directed treatment with ivermectin (CDTI) to eliminate onchocerciasis as a public health problem in the 17 APOC countries with areas where onchocerciasis is meso- and/or hyperendemic [3]. At the time, it was considered impossible for CDTI to interrupt transmission across Africa [4]. Thus, the UNICEF/UNDP/World Bank/World Health Organization Special Programme for Research and Training in Tropical Diseases (WHO/TDR) continued research for drugs or drug combinations which could eliminate onchocerciasis infection (e.g. [5]–[7]). Today, prospects for elimination of infection with CDTI appear better [8]–[10]. Questions remain as to whether CDTI alone can eliminate onchocerciasis in highly endemic areas [11]–[13].

Moxidectin, a milbemycin macrocyclic lactone, is registered worldwide as an anthelmintic in cattle, sheep, swine, horses and dogs [14]. Initiation of the clinical development of moxidectin was based on (i) published data [15]–[18] and investigator reports to TDR from *in vitro* (*O. volvulus*, *O. lienalis*, *O. gutturosa*) and *in vivo* models (*O. cervicalis* in horses, *O. lienalis* in mice, *O. ochengi* in cattle, *Brugia pahangi* in dogs and jirds) of onchocerciasis and lymphatic filariasis and (ii) toxicology data from development as a veterinary drug [19]. The objective of the development for human use is to assess through a series of non-clinical and clinical studies whether moxidectin could be safe for mass treatment for onchocerciasis control with an efficacy which modelling studies suggest could result in permanent interruption of transmission of *O. volvulus* after substantially less rounds of mass-treatment than ivermectin. Data from moxidectin use for veterinary parasites [14], *in vivo* models of human filarial infections and on the effects of ivermectin, an avermectin macrocyclic lactone, on *O. volvulus*, suggest several possible effects of moxidectin on *O. volvulus*. These include a microfilaricidal effect combined with an embryostatic effect after a single dose and/or an adult worm (macrofilariae) sterilizing effect and/or a macrofilaricidal effect upon repeated exposure. Furthermore, repeated exposure to moxidectin might reduce the life time of the macrofilariae as discussed by Geary and Mackenzie [20] for the effect of long term treatment with ivermectin. Given a half life of 20–40 days in healthy volunteers [21]–[25] (around 20 days in the participants in this study, unpublished data, manuscript in preparation), a potential effect of moxidectin on the viability or development of transmitted L3 larvae could also be considered.

The data from two studies in healthy volunteers [21], [24] and all toxicology data available at the time resulted in the decision to initiate the first study in individuals infected with *O. volvulus* [19], [26]. It was not known whether the putative microfilaricidal activity of moxidectin would be associated with a combination of severe Mazzotti reactions (i.e. the complex, acute inflammatory response of the body to the effect of the drug on microfilariae), similar to those seen after diethylcarbamazine treatment which make diethylcarbamazine unsuitable for mass treatment [27]–[32]. Consequently, the study was designed to determine in a small number of participants whether moxidectin induces severe reactions at a frequency suggesting that development of moxidectin should be discontinued. If that was not the case, the study was designed to determine the moxidectin dose(s) with an adverse reaction profile suitable for further clinical testing. Further clinical testing in a large number of participants would allow to better define moxidectin's safety profile and to quantify the difference in the effect of moxidectin and ivermectin on skin microfilariae levels.

The participant-safety driven design resulted in a study duration of ≥1.5 years. Therefore, participant follow up was expanded beyond that required for assessment of Mazzotti reactions to obtain pharmacokinetic data as well as initial data on moxidectin's effect on the parasite relative to that of ivermectin.

This paper summarizes the safety data with focus on statistically significant differences to ivermectin, presents the effect on the skin microfilariae (mf) and reports the results of the histological examination of the macrofilariae from subcutaneous nodules excised 18 months after treatment.

## Methods

### Ethics statement

This study was approved by the Ghana Food and Drugs Board, the Ghana Health Service Ethics Review Committee and the WHO Ethics Review Committee. Study conduct according to the principles laid down in the Declaration of Helsinki and in compliance with Good Clinical Practice and the protocol was monitored regularly.

Participants gave informed consent to study participation and testified to this by signature or thumbprint, as specified in the protocol approved by the Ethics Committees, in the presence of an independent literate witness in their villages before initiation of any study related procedures.

### Study design

**Figure 1:**
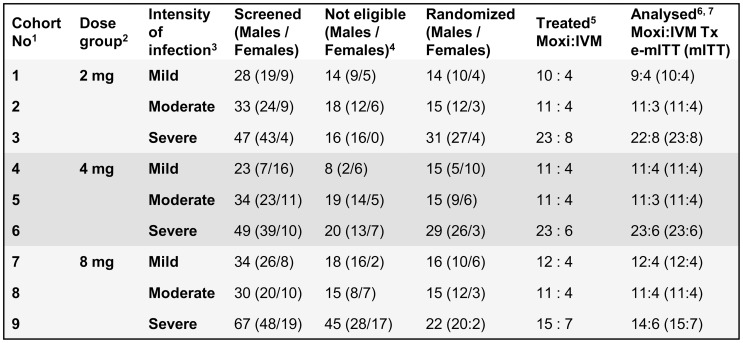
Number of participants by cohort screened, randomized, treated and analyzed. 1. Cohorts were screened for and eligible participants randomized and treated in sequential order. 2. In each dose group, subjects were randomized 3∶1 to the dose of moxidectin (Moxi) specified and ivermectin (IVM). 3. Mild: <10 microfilaria/mg skin, no ocular involvement, Moderate: 10–20 mf/mg skin, ocular involvement with sum of microfilariae in both eyes ≤10, Severe: >20 mf/mg skin without or with any level of ocular involvement. 4. Screen failure reasons: not meeting criteria for intensity of infection of the cohort for which screening was conducted (56%), laboratory values outside the protocol specified range (26%), ocular disease inconsistent with the eligibility criteria of the cohort for which screening was performed (7%), hypertension (6%) and others (5%, including age outside protocol specified range, orthostatic hypotension, pregnancy, weight below the limit specified in the eligibility criteria, history of neurologic/neuropsychiatric disorder/epilepsy). 5. All participants received the treatment they had been randomized to and completed the intervention (single dose study). 6. mITT modified intent to treat population including all treated participants. Safety analysis population. 7. e-mITT efficacy modified intent to treat population, including all participants who completed the study. Efficacy analysis was conducted for the e-mITT and the mITT population. 6 participants did not complete the study: 1 who died due to a snake bite, 1 who decided to withdraw from the study and 4 who were lost to follow up, i.e. could not be located despite several attempts.

The severity of many Mazzotti reactions correlates with the skin mf density [31]. Therefore, each of three dose levels of moxidectin (2 mg, 4 mg, 8 mg, established on the basis of the pharmacokinetic data from healthy volunteers [21], [24], 34–136 µg/kg or 0.05–0.21 µmol/kg for 59 kg body weight) was evaluated sequentially in three cohorts of participants with different levels of skin mf density and ocular involvement pre-treatment. In the first cohort, participants had a skin mf density <10 mf/mg skin and no ocular involvement (subsequently referred to as ‘mildly infected’). In the second cohort, participants had a skin mf density of 10 mf/mg to 20 mf/mg skin and the sum of microfilariae in both anterior chambers of the eye had to be ≤10 (subsequently referred to as ‘moderately infected’). Participants of the third cohort had skin mf density >20 mf/mg skin without or with any level of ocular involvement (subsequently referred to as ‘severely infected’) (Figure 1).

In each mildly and moderately infected cohort, 16 participants were planned to be enrolled and randomized in a ratio of 3∶1 to moxidectin or 150 µg/kg ivermectin (as per ivermectin labelling for use in onchocerciasis). This provided 4 ivermectin treated participants as concurrent controls for the safety data in the planned 12 moxidectin treated participants in each cohort. To increase the probability of detection of adverse events in participants with high skin mf density (who are most likely to experience Mazzotti reactions [31]), 32 severely infected participants were planned to be enrolled for each moxidectin dose level and randomized 3∶1 to moxidectin∶ivermectin. This provided 8 ivermectin treated participants as concurrent controls for the safety data of 24 moxidectin treated participants. Across all 9 cohorts, the planned number of participants resulted in 48 participants in each treatment group for comparison of the safety data as well as the effects on the parasite. Figure 1 shows the number of participants actually treated in each cohort and provides the screen failure reasons which resulted in these numbers being lower than the planned numbers.

**Figure 2:**
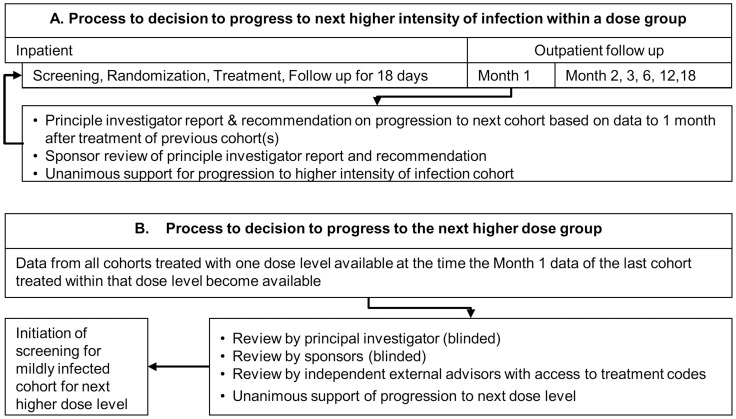
Cohort enrolment, treatment and follow up and decision making for progression to screening of the next intensity of infection cohort within one dose group (A) and for progression to screening for the mildly infected cohort at the next higher dose level (B).

Mazzotti reactions usually subside within one or two weeks after treatment [30]. Since no data on Mazzotti reactions following moxidectin treatment were available, the decision to treat the next cohort within one moxidectin dose level was made based on the safety data obtained during the first month after treatment of the previous cohort. Progression to the next dose level was decided upon based on all data available to Month 1 follow up of the last cohort at the previous dose level (Figure 2). To further decrease potential risk to participants, all participants remained in the study center for 18 days after treatment. Subsequent follow up to 18 months was on an outpatient basis.

### Participants

**Figure 3:**
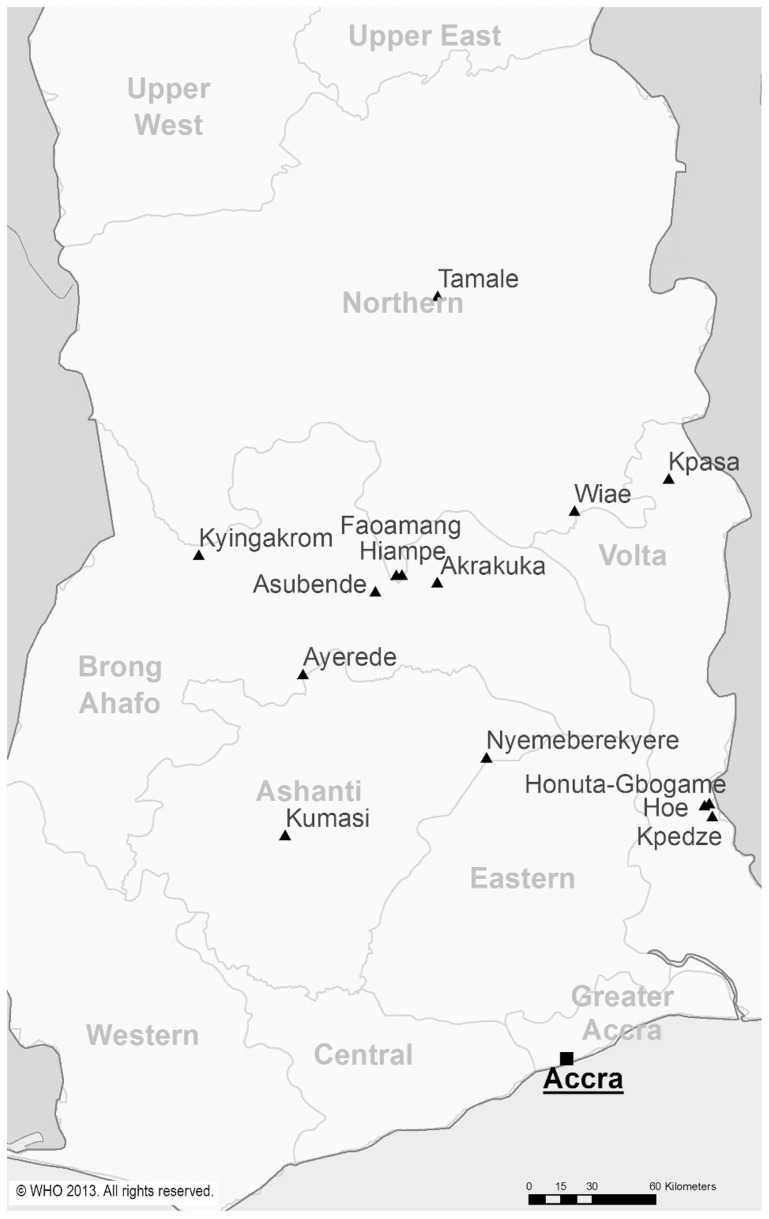
Map of Ghana showing the location of some towns/villages in the area from which participants were recruited. The boundaries and names shown and the designations used in this map do not imply the expression of any opinion whatsoever on the part of the World Health Organization concerning the legal status of any country, territory, city or area or of its authorities, or concerning the delimitation of its frontiers or boundaries.

Participants were recruited from onchocerciasis endemic villages between 0°30′ and 0°45′E, 6°45′ and 7°0′N within the River Tordzi basin in the Volta Region of South-eastern Ghana. The vast majority (90%) of participants came from the villages Honuta-Gbogame, Kpedze-Anoe, Togorme, Aflakpe, Luvudo, Kpoeta-Ashanti and Hoe, the remainder from 11 other villages in the area (Figure 3). This area was not included in vector control activities under the Onchocerciasis Control Programme because at the time of the OCP it was forested. Simuliid species were *Simulium yahense* and *Simulium squamosum* [33]. At the time of this study, the area was not yet included in the ivermectin mass distribution programme of the National Onchocerciasis Control Programme because it is overall hypoendemic with small meso- or hyperendemic foci. The area is not endemic for lymphatic filariasis or loiasis.

A total of 172 of 196 planned individuals meeting the intensity of infection criteria described above but otherwise regarded as healthy based on physical examination, electrocardiography, medical and medication history, serum biochemistry, haematology and semiquantitative urinalysis participated in the study. Volunteers with a history of or current neurological or neuropsychiatric disease or epilepsy, orthostatic hypotension at screening, hyperreactive onchodermatitis and antifilarial therapy within the previous 5 years as well as pregnant and breastfeeding women were excluded. Women of child-bearing potential who wanted to participate had to agree to contraception (depo-medroxyprogesterone acetate or levonorgestrel implants) during the first 150 days after treatment. The pre-treatment evaluations included those detailed in the footnote to Table 1 and height measurement. Vital signs were obtained 12 times during the pre-treatment evaluations and the mean was used to assess changes post-treatment.

**Table 1:** Overview of assessment of treatment effects.

Assessment	Days during stay in research center								Months outpatient		
	1,2	3,4	5,6	7,8	9–13	14,15	16,17	18	1,2,3,6	12	18
**Safety**											
Weight	X	X	X	X	X	X	X		X	X	X
PE^1^	X	X	X	X	X	X	X	X	X	X	X
VS^2^	X	X	X	X	X	X	X	X	X	X	X
ECG^3^	X	3									
OE^4^		3/4		7/8		14/15			X	X	X
LE^5^	X	4		8	13			X	X	X	X
Questioning	X	X	X	X	X	X	X	X	X	X	X
**Efficacy**											
Skin snips^6^				8					X	X	X
Nodulectomy^7^											X
OE^4^		3/4		7/8		14/15			X	X	X
PK^8^	X^8^	4^8^		8	13			18	X	X	

D: Day, M: Month. X indicates examination at each time point indicated in the top row, numbers indicate the day or alternative days for performing the examination.

1 PE: Physical examination including neurological examination and subcutaneous nodule palpation.

2 VS: Vital signs including temperature, respiratory rate, pulse rate (PR) and blood pressure (BP) after at least 5 minutes supine.

PR and BP were repeated after 2 minutes standing following ≥5 minutes supine.

Day 1: once before and 3 times after drug administration PR and BP after ≥5 minutes supine and subsequent 2 minutes standing still, Days 2–8: 5 times, PR and BP after ≥5 minutes supine and subsequent 2 minutes standing still, Days 9–17: twice, supine only, M1–M18: once, supine only.

3 12 lead ECG on day 1 approximately 4 hours after drug administration.

4 OE: Ocular Examination included visual acuity, visual fields (calibrated Goldman perimeter), colour vision, intraocular pressure, examination of the fundus, slit lamp examination of anterior segment, counting of microfilariae in anterior chamber, living and dead microfilariae in cornea and punctate opacities.

Colour fundus photography and fluorescein angiography to month 3, thereafter as per protocol only in participants with lesions or visual defects on OE (which was not applicable).

5 LE: Laboratory evaluations included: serum biochemistry (Na^+^, K^+^, Cl^−^, bicarbonate, glucose, total protein, albumin, urea, creatinine, alkaline phosphatase, lactic dehydrogenase, total bilirubin, gamma-glutamyl transpeptidase, aspartate aminotransferase, and alanine aminotransferase), hematology (prothrombin time, a complete blood cell count, hematocrit, hemoglobin, 5-part differential white blood cell count, platelet count), dipstick semiquantitative urinalysis microscopic urine evaluation, urine and blood microfilariae quantitation after nucleopore membrane filtration and Giemsa staining.

6 Minimum of 1 mg from each iliac crest and calf with a 2 mm corneoscleral punch.

7 Aseptical excision of all palpable nodules under 2% xylocaine anaesthesia.

8 Blood sampling time points for pharmacokinetics (PK) on day 1–4: within 2 hours of drug administration, 1, 2, 4, 8, 24, and 72 hours after treatment.

### Interventions, randomized treatment allocation, blinding and treatment

The 3 mg ivermectin tablets (purchased from Merck and Co. Inc), 2 mg moxidectin tablets developed for human use, as well as placebo were provided by Wyeth in identical looking capsules. Inactive ingredients of the moxidectin tablets were microcrystalline cellulose, anhydrous lactose, sodium croscarmellose, sodium lauryl sulfate, colloidal silicon dioxide, and magnesium stearate. Placebo capsules contained the inactive ingredients of the moxidectin tablets. Each participant received 4 capsules provided in an envelope labelled only with subject identifying information, resulting in participants and investigative team being blinded.

In each cohort, participants were stratified by sex and randomly allocated by a pharmacist in a ratio of 3∶1 to receive a single oral dose of moxidectin or ivermectin (150 µg/kg) based on computer-generated randomization schedules with a block size of 4 provided by the sponsors. The pharmacist prepared for each participant an envelope which contained 4 capsules, including 1, 2 or 4 capsules containing a 2 mg moxidectin tablet or 2, 3 or 4 capsules containing a 3 mg ivermectin tablet and the complementary number of placebo capsules. The pharmacist gave the sealed envelopes to the investigative team and was not otherwise involved in the study.

Treatment was administered on day 1 between 7:00 and around 7:40 under observation by members of the investigative team after an overnight fast and vital sign measurement.

### Outcomes

Treatment effects were evaluated daily during the first 18 days and 1, 2, 3, 6, 12 and 18 months after treatment (Table 1).

Safety outcomes: Adverse events (AEs), including (i) clinically significant changes in laboratory values from pre-treatment, (ii) clinically significant adverse changes from pre-treatment in systemic or ocular symptoms detected through examinations, spontaneous reporting by participants or questioning of participants, and (iii) changes in vital signs, whether clinically significant or not. AEs were to be classified as serious (SAE) if they met the criteria in the SAE definition in the ‘Guideline for Good Clinical Practice’ of the ‘International Conference on Harmonization of Technical Requirements for Registration of Pharmaceuticals for Human Use’, i.e. if they resulted in death, persistent or significant disability or incapacity, a congenital anomaly or a birth defect or were life-threatening or required inpatient hospitalization or prolongation of an existing hospitalization [34] or if they were important medical events, including cancer, that could jeopardize the subject and require medical or surgical intervention to prevent one of the outcomes above. The severity of all AEs was graded (grade 1–4) according to the Onchocerciasis Chemotherapy Research Center (OCRC) Common Toxicity Criteria (OCRC CTC) version 2.0 (Supporting Table S1), an expansion of the criteria developed at OCRC for quantitation of Mazzotti reactions [35], [36]. Severity of AEs not included in the OCRC CTC was graded according to the National Cancer Institute Common Toxicity Criteria version 2.0 [37]. All AEs were categorized by the Principal Investigator (KA) as Mazzotti reactions, non-Mazzotti adverse drug reactions or drug-unrelated adverse events.

Efficacy outcomes: Skin snips were obtained with a 2 mm Holth or Walser corneoscleral punch. One snip each was taken from the right and left iliac crest as well as the right and left calf for a total of 4 snips/participant pre-treatment, 8 days and 1, 2, 3, 6, 12 and 18 months after treatment. Each skin snip was weighed on an electronic balance to an accuracy of 0.1 mg. Snips were incubated individually in a well of a 96-well plate with flat bottom for at least 8 hours in 0.9% normal saline at approximately 22°C. Microfilariae were counted under an inverted microscope at a magnification of 60× supplemented by 100× when necessary. The skin mf density of each participant at each follow up time point was calculated as the arithmetic mean number of mf/mg across the four skin snips. The change from pre-treatment in *O. volvulus* skin mf densities was calculated as the difference between skin mf density at follow up and pre-treatment in absolute terms and as the percentage of pre-treatment density for each follow up time point. The proportion of participants with undetectable levels of skin mf was calculated as the proportion of participants without mf in each of the four skin snips.

The palpable nodules from the participants who attended the 18 month follow up visit and agreed to their excision were excised at that time (35/38 in the 2 mg moxidectin group, 37/37 in the 4 mg moxidectin group, 24/37 in the 8 mg group, 30/42 in the ivermectin group). Nodules were processed as described previously [6], [38]. The histological assessment was based on the examination of three 4 µm sections obtained along the longest axis of each nodule in a way that each third of a nodule was sampled. Worms were examined and classified by a single observer (SKA) according to the criteria described previously [38], [39]. Each macrofilaria identified was classified by sex and as alive, moribound, dead or dead and calcified based on the presence or absence of normal or degenerate cuticle, hypodermis and muscles, of normal, degenerate and/or pigmented intestine and genital tract wall, deposits in the body cavity, nearly complete resorption of the worm, calcification of organs or of the worm, and presence of giant cells. Female macrofilariae were furthermore classified as young or not. The reproductive status of each female macrofilaria was classified based on the presence or absence of an empty genital tract, normal or degenerate oocytes, morulae, gastrulae, coiled mf, stretched mf, sperm in the uterus, polymorphous material, presence of oocytes and remant mf only, or mixed development. Gravid female macrofilariae were categorized as in full production of developmental stages, as in less than full production of developmental stages, as having resumed production of developmental stages and showing the presence of pre-microfilarial stages (morulae and gastrulae) or as going out of embryonic production and showing the presence of coiled mf (pretzels) and stretched mf. Furthermore, the number of mf in the nodule was determined. The reproductive status of male macrofilariae was categorized based on an empty genital tract and presence or absence of normal or degenerate spermatogonia, primary spermatocytes, secondary spermatocytes, spermatids, or spermatozoa [38], [39].

### Sample size

The sample size for each pre-treatment intensity of infection category (mild, moderate, severely infected) and treatment arm was chosen to minimize the number of participants exposed to moxidectin while providing a relatively high probability of detecting frequent adverse events. The probabilities of events with given true event rates occurring with a given sample size can be calculated using the binomial distribution [40]. The planned sample size for each pre-treatment intensity of infection category combined with the 3∶1 moxidectin∶ivermectin randomization ratio provides for each moxidectin dose level a probability of >90% to detect at least 1 event with a true event rate of 20% among the 12 mildly or moderately infected participants, at least 1 event with a true event rate of 10% among the 24 severely infected participants and at least 1 event with a true event rate of 5% among the total of 48 participants with any intensity of infection exposed to a specific moxidectin dose or ivermectin.

The planned 48 participants per treatment group chosen in view of the adverse event detection probabilities, provided approximately 92% power to detect a statistically significant difference between any 2 treatment groups when the percentage of participants with undetectable levels of skin mf was approximately 99.9% in one and 80% in the other group. This estimate is based on a 2-sided Fisher's Exact test with Type I error of 0.05 without adjustment for multiple comparisons which was not necessary due to the hierarchical way comparisons for efficacy were performed (see Statistical Methods).

### Statistical methods

#### Number analysed

The analysis of the safety data included the data from all 172 participants treated (modified Intent-to-Treat population, mITT). The efficacy analyses were conducted for all participants who had completed the study (efficacy modified Intent-to-Treat population, e-mITT population, 163/172 treated, Figure 1) as well as for the mITT population.

All ivermectin treated participants enrolled in all 9 cohorts from September 2006 to June 2008 were pooled for the analyses (Figure 1). For the efficacy assessment, the appropriateness of pooling was tested with a mixed-effects model comparing the skin mf data in ivermectin treated participants with respect to the moxidectin dose-group and time they had been treated. Pooling was considered justified because dose-group and dose-group by time interaction were not significant at the 0.05 level and the 0.10 level of significance, respectively.

#### Safety data analysis

The number of subjects with each category and each type of AE were compared between all moxidectin treated and all ivermectin treated participants as well as pairwise between the 4 treatment groups with a 2-tailed Fisher's exact test.

#### Efficacy data analysis

The arithmetic mean skin mf densities of each participant at each follow up time point, calculated as described above, were logarithmically transformed (y = log(skin mf density+1)) prior to analysis of the change in skin mf density from pre-treatment with a mixed-effects model with repeated measures. The model included treatment group, time, treatment group-by-time, pre-treatment intensity of infection category (mild, moderate, severe), sex, 2-way interactions of treatment group, sex and pre-treatment intensity of infection category, and the 3-way interaction treatment group×pre-treatment intensity of infection×sex. Stratified Cochran-Mantel-Haenszel analysis with treatment group, sex and pre-treatment intensity of infection category (mild, moderate, severe) as factors in the analysis was used for analysis of the proportions of participants with undetectable levels of skin mf. All statistical tests were 2-sided tests at the 0.05 significance level and conducted in a hierarchical way to control for the Type I error: If the test of moxidectin 8 mg vs. ivermectin was not significant, no additional comparisons were made, else 4 mg moxidectin was tested against ivermectin. If that difference was not statistically significant, no additional comparisons were made, else 2 mg moxidectin was tested vs. ivermectin.

#### Unplanned analyses

**Figure 4:**
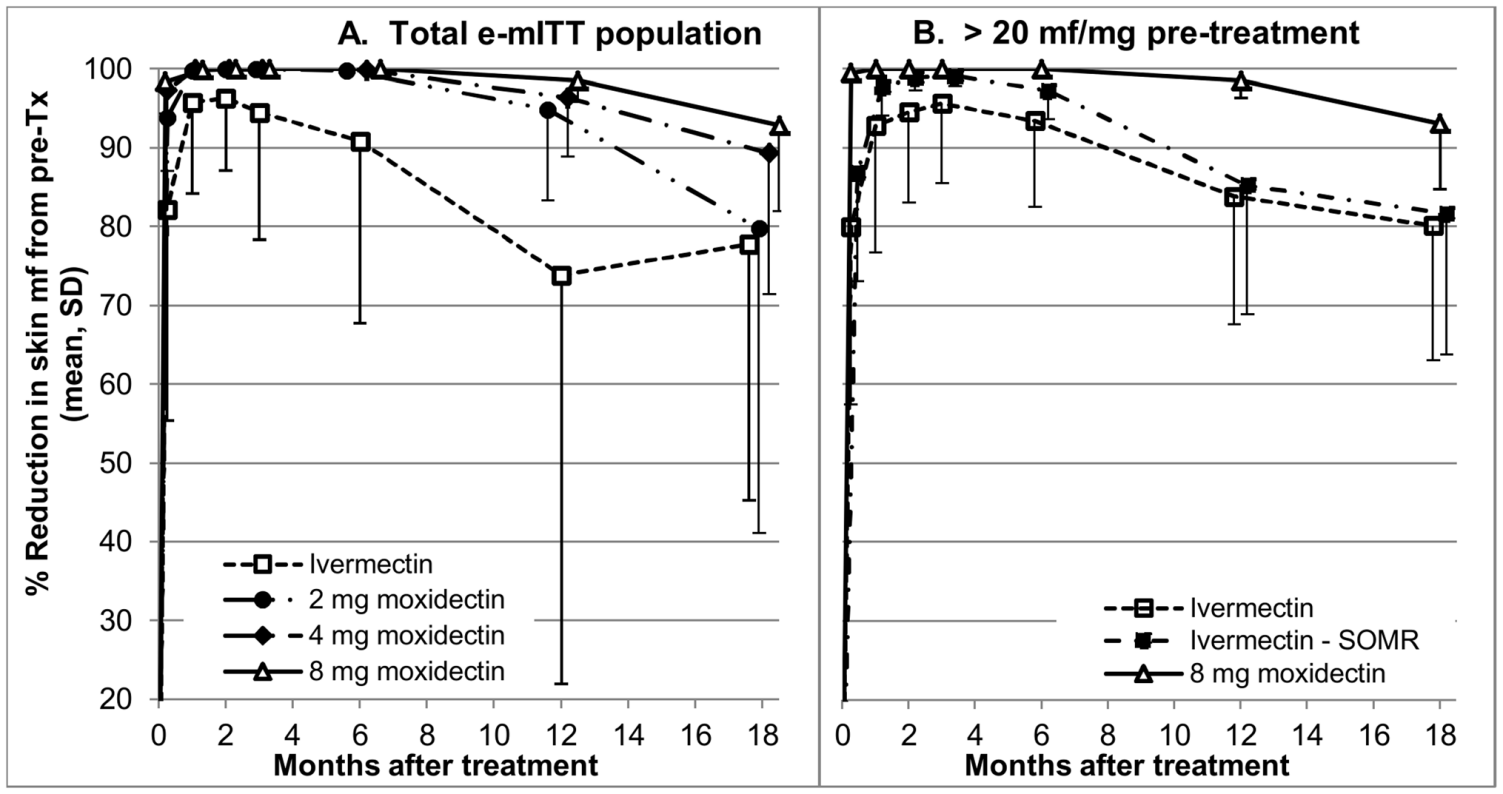
Percentage reduction from pre-treatment in skin microfilariae density (mean, standard deviation) 8 days, 1, 2, 3, 6, 12 and 18 months after treatment by treatment group. A Total e-mITT population, B Severely infected in the e-mITT population treated with ivermectin or 8 mg moxidectin. For the ivermectin treatment group, means and standard deviations are shown across all severely infected and without the suboptimal microfilariae responders (Ivermectin - SOMR). Tx – treatment, SD – standard deviation shown in one direction. Marker positions for different treatment groups have been placed around the measurement time point to allow, to the extent possible, differentiation between overlapping means and SD.

(i) Review of the unblinded data showed that the participants with a decrease in skin mf levels not meeting the criteria for ‘adequate response’ [41], [42] had all been treated with ivermectin. Consequently, as a sensitivity analysis, the analysis of the change in mean skin mf density in the e-mITT population as well as descriptive statistical analysis were conducted after exclusion of three ivermectin treated participants with inadequate response to the microfilaricidal effect of ivermectin. (ii) Review of the unblinded data further showed that the time after treatment at which the skin mf density reached the nadir and the time at which an increase in skin mf density started varied significantly, in particular among the ivermectin treated participants. This resulted in a substantial underestimate of the percentage of ivermectin treated subjects whose skin mf levels decreased below the level of detection in the planned ‘by time point’ analyses. To characterize better the variability in the response to the drugs, the follow up time point at which the skin mf density reached the lowest measured value (nadir) and the follow up time point at which a sustained increase of the skin mf density started (determined as the time of the first of two successive skin mf densities above the nadir or month 18, if first value above the nadir was at month 18) was determined for each participant. Subsequently, the percentage of participants who reached the skin mf nadir and whose skin mf density started to increase was calculated for each follow up time point and treatment group. (iii) After finalization of the statistical analysis plan, the results of modelling of onchocerciasis elimination through annual mass treatment with a stochastic transmission model were published [12]. This model quantifies the effect of an anti-onchocercal drug as the average reduction from pre-treatment of the skin mf levels in the population in the year following the treatment. This reduction (referred to in [12] as ‘CDTI efficacy’) will be referred to here as the ‘average annual reduction in skin mf density’ (AAR). The AAR is a function of the effect of the drug on skin mf levels over the year following the treatment in treated people and of the percentage of the infected population treated (which is lower than 100% in a mass treatment setting and 100% in the study presented here). The AAR was calculated as the area under the curve of the percentage reduction in skin mf density from pre-treatment to 12 months (Figure 4) for each participant in this study by triangulation and summarized by descriptive statistics per treatment group.

All planned analyses as well as the unplanned sensitivity analysis were performed with SAS Version 9.1. The other analyses were performed using Microsoft Excel 2010.

## Results

### Participant flow

A total of 172 of 196 planned participants were included in the study and treated (Figure 1). The difference between the number of planned and treated participants is due to the fact that it was not possible to recruit within the protocol specified timelines for each cohort 16 participants who met the eligibility criteria. Screen failure reasons included not meeting criteria for intensity of infection for the cohort for which screening was conducted (56%), laboratory values outside the protocol specified range (26%), ocular disease not meeting the criteria for the cohort for which screening was conducted (7%), hypertension (6%) and others (5%, including age outside protocol specified range, orthostatic hypotension, pregnancy, weight lower than specified in the protocol, history of neurologic/neuropsychiatric disorder/epilepsy).

Of the 172 treated participants (mITT population), 6 discontinued from the study before the 18 months follow up examination (Figure 1), including 1 who died due to a snake bite, 1 who decided to withdraw from the study and 4 who were lost to follow up, i.e. could not be located despite several attempts to find them.

### Pre-treatment characteristics

Table 2 shows the demographics and *O. volvulus* infection related pre-treatment characteristics. Testing for differences between treatment groups in age, sex, height, weight and subjects by intensity of infection showed no statistically significant differences. With the eligibility criteria precluding ocular involvement in approximately 25% of participants (mildly infected cohorts) and limiting it in a further approximately 25% (moderately infected cohorts), ocular involvement overall was very low. Table 2 also shows the number of onchocercal and non-onchocercal nodules determined at histological assessment of all excised nodules at Month 18.

**Table 2:** Demographics and pre-treatment characteristics of all participants treated (mITT population).

	Ivermectin	2 mg moxidectin	4 mg moxidectin	8 mg moxidectin
Number treated	45	44	45	38
Number (%) who completed study (e-mITT population)	42 (93.3)	42 (95.5)	45 (100)	37 (97.4)
Mean (range) age [years]	34.5 (17–58)	38.3 (18–58)	37.6 (19–57)	32.1 (18–60)
Mean (range) weight [kg]	58.01 (47.2–70.8)	59.9 (45.8–86.9)	57.6 (43.2–74.0)	59.19 (42.7–88.7)
Number (%) of women	12 (26.7)	8 (18.2)	14 (31.1)	7 (18.4)
Mean (range) skin mf density [mf/mg skin]	21.31 (0.51–64.00)	23.62 (0.19–84.38)	20.61 (1.78–61.50)	22.54 (0.30–83.13)
No (%) of participants				
No (%) with mild infection	12 (26.7)	10 (22.7)	11 (24.4)	12 (31.6)
Mean (SD) mf/mg skin in mildly infected	3.94 (3.07)	2.68 (2.46)	3.77 (2.15)	4.44 (2.65)
No (%) with moderate infection	12 (26.7)	11 (25.0)	11 (24.4)	11 (29.0)
Mean (SD) mf/mg skin in moderately infected	14.25 (3.05)	15.39 (2.84)	13.13 (2.23)	13.45 (2.61)
No (%) with severe infection	21 (46.7)	23 (52.3)	23 (51.1)	15 (39.5)
Mean (SD) mf/mg skin in severely infected	35.26 (11.31)	36.66 (14.64)	32.25 (10.03)	43.69 (17.81)
No (%) with palpable nodules pre-treatment*	34 (75.6)	38 (86.4)	40 (88.9)	25 (65.8)
No (%) with excised nodules at Month 18	30 (66.6)	35 (79.5)	37 (82.2)	24 (63.2)
No (%) with non-onchocercal nodules**	8/30 (26.7)	8/35 (22.9)	5/37 (13.5)	4/24 (16.6)
No (%) with onchocercal nodules**	24/30 (80.0)	32/35 (91.4)	35/37 (94.6)	22/24 (91.7)
No (%) with prior nodulectomy***	9 (20.0)	16 (36.4)	7 (15.6)	2 (5.3)
No (%) with mf in the blood	1 (2.2)		1 (2.2)	1 (2.6)
No (%) with mf in urine	2 (4.4)		2 (4.4)	
No (%) with mf in anterior chamber (MFAC)	8 (17.8)	9 (20.5)	10 (22.2)	11 (28.9)
MFAC mean (range) in those with MFAC	6.1 (1–25)	3.7 (1–8)	7.0 (1–39)	2.6 (1–5)
No (%) with live corneal mf [count]	1 (2.2) [5]	0	1 (2.2) [1]	0
No (%) with dead corneal mf [count]	1 (2.2) [3]	0	2 (4.4) [1], [3]	0
No (%) with Punctate opacities (count)	1 (2.2) [26]	0	1 (2.2) [21]	0
No (%) with visual acuity loss 6/9–6/12****	3 (6.7)	6 (13.6)	3 (6.7)	5 (13.2)
No (%) with visual acuity loss >6/12****	0	2 (4.5)	2 (4.4)	3 (7.9)
No (%) with visual field loss	2 (4.4)	3 (6.8)	4 (8.9)	5 (13.2)
No(%) *M. perstans* infected	2 (4.4)	1 (2.3)	5 (11.1)	0
Mean (range) *M. perstans* mf/ml blood	3.38 (0–116)	0.1 (0–4)	7.0 (0–234)	0
No (%) *M. streptocerca* infected	2 (4.4)	0	0	2 (5.3)
Mean (Range) *M. streptocerca* mf/mg	0.04 (0–1.77)	0	0	0.06 (0–1.9)

* Presence of palpable nodules was not an inclusion criterion.

** Based on histological examination of all palpable nodules excised at month 18.

Percentages calculated based on number of subjects with excised nodules.

***Nodulectomies >5 years ago.

**** Based on eye with lower acuity.

### Adverse events

Table 3 shows the number of subjects with different categories of AEs, with Mazzotti reactions for which Fisher's exact test between at least one moxidectin treatment group and the ivermectin treatment group resulted in a p-value of <0.05, and with severe symptomatic postural hypotension (SSPH).

**Table 3:** Number (%) of subjects with adverse events by adverse event category, with Mazzotti reactions for which Fisher's exact text between at least one moxidectin treatment group and ivermectin treatment group yielded p-value<0.05 and for SSPH (mITT population).

AE category or AE	Ivermectin (N = 45)	2 mg moxidectin (N = 44)	4 mg moxidectin (N = 45)	8 mg moxidectin (N = 38)
Any AE	45 (100)	43 (97.7)	45 (100)	37 (97.4)
Any UnAE	42 (93.3)	41 (93.2)	41 (91.1)	36 (94.7)
Any laboratory value based UnAE^1^	0	1 (2.2)	2 (4.4)	1 (2.6)
Any non-Mazzotti ADR	1 (2.2)	0	2 (4.4)	1 (2.6)
Any MAZ	43 (95.6)	38 (86.4)	45 (100)	37 (97.4)
Pruritus	25 (55.6)	20 (45.5)	27 (60.0)	33 (86.8)*
Rash	19 (42.2)	18 (40.9)	23 (51.1)	24 (63.2)*
Increase in pulse rate (standing^2^)	16 (35.6)	21 (47.7)	14 (31.1)	23 (60.5)*
MAP decrease (standing^2^)	12 (26.7)	8 (18.2)	12 (26.7)	23 (60.5)*
Grade 4 MAP decrease - SAPH^3^		3 (6.8)	1 (2.2)	6 (15.8)*
Grade 4 MAP decrease - SSPH^4^	1 (2.2)	1 (2.3)	4 (8.9)	5 (13.2)

UnAE Adverse event unrelated to study drug or study participation. ADR Adverse drug reaction, MAZ Mazzotti reaction, MAP mean arterial pressure: Diastolic pressure+1/3 (Systolic pressure - Diastolic Pressure).

1 2 mg: Anaemia from 144 days post-treatment, 4 mg: prolonged prothrombin time from 549 days post-treatment, haematuria 390 days post-treatment, 8 mg: prolonged prothrombin time 32 days after treatment.

2 After standing still for 2 minutes following at least 5 minutes supine.

3 SAPH severe asymptomatic postural hypotension, asymptomatic decrease in MAP by ≥35 mmHg relative to baseline after 2 min standing still following ≥5 minutes supine.

4 SSPH severe symptomatic postural hypotension, diagnosed when a subject cannot stand still for 2 minutes after ≥5 minutes supine due to drop in blood pressure.

* p<0.05 for pairwise comparison of moxidectin treatment group vs. ivermectin treatment group.

#### Adverse events unrelated to study drug or study participation

Nearly all participants experienced AEs regarded by the principal investigator as unrelated to study drug or study participation, reflecting the environment and health status of the local population (e.g. malaria and other infections, injuries). All SAEs (malaria, pneumonia, snake bite, coma, grand mal convulsion (attributed to participant's abuse of alcohol), schizophrenia (attributed to participant's customary beliefs) and typhoid fever) were regarded as unrelated to study drug or study participation.

#### Non-Mazzotti adverse drug reactions

AEs considered as possibly or probably caused by the intrinsic effect of the drug on the body included a mild headache in an ivermectin treated participant from 18 to 27 days after treatment and a mild papular rash in one 4 mg moxidectin treated participant from day 2 to 4 after treatment.

#### Mazzotti reactions

A total of 163/172 (94.8%) participants had Mazzotti reactions. Pruritus was mild in all but one severely infected participant treated with 2 mg moxidectin who was given chlorpheniramine for relief of symptoms. Rash was mild or moderate in all but one severely infected participant treated with 2 mg moxidectin and one moderately infected participant treated with ivermectin. All rashes resolved without treatment. Increase in standing pulse rate relative to pre-treatment was mild or moderate in all but one severely infected participant treated with 4 mg moxidectin and one mildly infected participant treated with 8 mg moxidectin.

The fall in mean arterial pressure (MAP) upon standing still for 2 minutes after ≥5 minutes in the supine position relative to pre-treatment was asymptomatic in the majority of participants including in participants with high (fall by ≥30-<35 mmHg) and with very high (fall by ≥35 mmHg, severe asymptomatic postural hypotension, SAPH) decrease. Symptomatic postural MAP changes, i.e. SSPH [43], occurred in more participants treated with 8 mg or 4 mg moxidectin than treated with 2 mg or ivermectin (p>0.05, Table 3). None of these participants required any treatment beyond the advice to lie down for a few minutes.

Supplementary Table S2 provides the frequency of the Mazzotti reactions in Table 3 broken down by intensity of infection pre-treatment. While the small number of participants, not chosen in view of comparison of the frequency of Mazzotti reactions between treatment groups or between participants with different intensities of infection, limits conclusions, the data in Table S2 suggests that a fall in MAP upon standing up and still for 2 minutes, whether symptomatic or not, is more likely to occur in people with moderate or high than in those with lower skin mf densities pre-treatment. This is consistent with observations on the frequency and severity of Mazzotti reactions after treatment with other anti-onchocercal drugs [31].

### Effect on skin microfilariae levels

**Figure 5:**
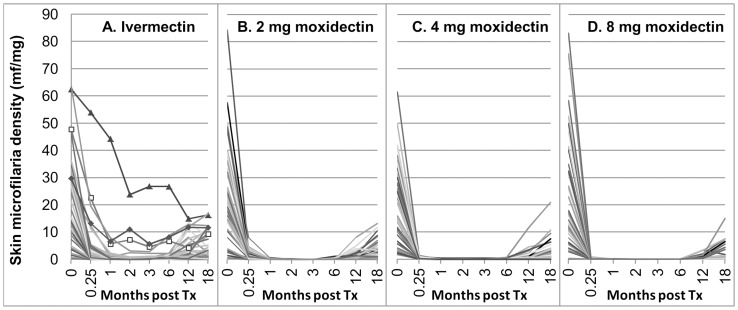
Skin microfilariae density in individual participants pre-treatment (0) and at the different times post-treatment (e-mITT population) in the four treatment groups. A Ivermectin. The data for the three participants treated with ivermectin whose decrease in skin microfilariae levels did not meet the criteria of ‘adequate response’ are indicated by markers at the evaluation time points. B 2 mg moxidectin, C 4 mg moxidectin, D 8 mg moxidectin.

The skin mf densities in the individual participants before (0) and after treatment are shown in Figure 5. Figure 4 shows the percent reduction from pre-treatment in mean mf density after treatment in each treatment group across all participants who completed the study (e-mITT population) and after treatment with ivermectin and 8 mg moxidectin for the subgroup with >20 mf/mg skin pre-treatment.

The decrease in skin mf density after ivermectin treatment (for overview of decrease seen in other studies see [44]) was also observed after treatment with 2 mg, 4 mg or 8 mg moxidectin, but was faster and more extensive (Figure 4, Figure 5). From day 8 onward, the decrease in skin mf density from pre-treatment was significantly higher after treatment with any of the moxidectin doses than after treatment with ivermectin in both the e-mITT and the mITT populations (<0.005). Supporting Table S3 shows descriptive statistics and the results of the statistical analysis of the data for the e-mITT population.

**Figure 6:**
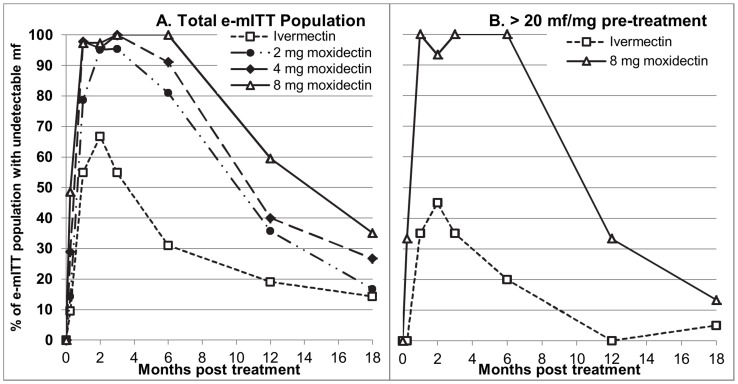
Percentage of participants with undetectable levels of skin microfilariae by treatment group and time post-treatment. A total e-mITT population, B Severely infected in the e-mITT population treated with ivermectin or 8 mg moxidectin.

The faster and more extensive decrease in skin mf density was reflected in the proportion of participants with undetectable levels of skin mf (Figure 6). This proportion was significantly higher than in the ivermectin group already on day 8 in the 4 mg and 8 mg moxidectin groups (p<0.02) and from month 1 onward in all moxidectin groups in both the e-mITT and the mITT populations (p<0.01). Supporting Table S4 shows the descriptive statistics and the results of the statistical analysis of the data for the e-mITT population.

In three ivermectin treated participants in the e-mITT population (skin mf density pre-treatment 29.7–62.5 mf/mg), as well as in one participant (skin mf density pre-treatment 19.3 mf/mg) who discontinued from the study after the 12 month follow up, the reduction in skin mf density from pre-treatment to day 8 was <60% and the skin mf density was >6% of the pre-treatment value 3 months after treatment. Thus, the reduction in skin mf density in these participants did not meet the criteria for an ‘adequate parasite response’ to ivermectin defined by Awadzi and coworkers (≥60% reduction in skin mf level from pre-treatment level on day 8 post-treatment, skin mf density ≤6% of pre-treatment density 3 months post-treatment) [41]. These participants are referred to below as ‘Suboptimal Microfilariae Responders’ (SOMR) to be distinguished from ‘Suboptimal Responders’ reported in other studies [41], [42], [45], [46] in whom the reduction in skin mf levels is as expected, but the increase in skin mf levels following the initial decrease is faster and/or more extensive than considered consistent with ‘adequate parasite response’ by Awadzi and coworkers (skin mf density ≤40% of pre-treatment density at 12 months after treatment). Figure 4 B shows the percentage reduction from pre-treatment across all severely infected ivermectin treated participants who completed the study as well as after exclusion of the SOMRs. The conclusions from the statistical analysis of the change in skin mf densities between moxidectin and ivermectin treated groups did not change when the three SOMRs in the e-mITT population were excluded. A response to ivermectin not meeting the criteria for adequate response was also observed in four other ivermectin treated participants in the e-mITT population. These participants are not considered here as SOMRs because the intensity of infection pre-treatment (10.2 mf/mg, <1 mf/mg for three of them) was below that from which the criteria were derived and at low intensity of infection the variability between mf counts in individual snips can have too large an impact on mean skin mf density to support definitive conclusions, even when the skin snip weight is taken into account. No participant treated with moxidectin showed a response fitting the criteria for SOMRs.

**Figure 7:**
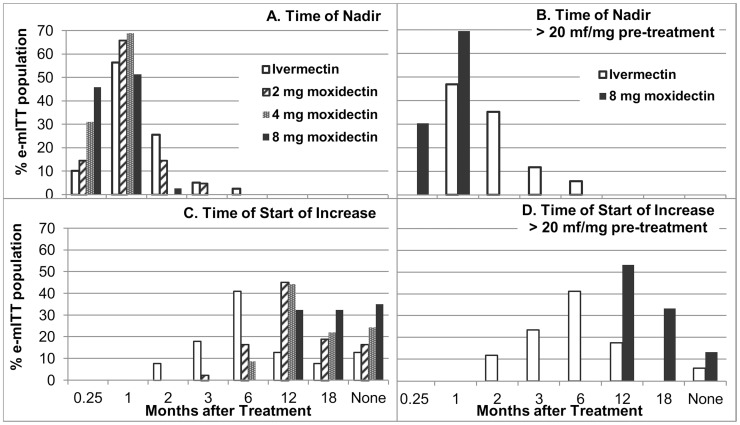
Time of nadir and start of sustained increase in skin microfilariae levels by treatment. A Time of nadir among participants with any intensity of infection, B Time of nadir among severely infected participants, C Time of start of sustained increase (time of two subsequent measurements above the nadir) among participants with any intensity of infection, D Time of start of sustained increase among severely infected participants. Data analyzed are those of participants who completed the study excluding participants with a decrease in skin microfilariae levels not meeting the criteria for ‘adequate response’ (SOMRs).

Figure 7 A and B show for the e-mITT population and for the subgroup of severely infected participants excluding the three SOMRs, respectively, the time from treatment to recorded skin mf density nadir. Figure 7 C and D show the time of start of recorded sustained increase in skin mf density. In the ivermectin group, three (7.7%) participants showed an increase in skin mf levels as early as 2 months after treatment (0.1–2.53 mf/mg) while three other participants reached the nadir only at 3 or 6 months (undetectable – 2.89 mf/mg). Overall, 85% of ivermectin treated participants who completed the study had undetectable levels of skin mf recorded at one point, including 76% of severely infected participants.

An increase in skin mf levels was first observed among 2 mg moxidectin treated participants 3 months after treatment (n = 1, 0.14 mf/mg skin), among 4 mg treated participants 6 months after treatment (n = 4, 0.08–0.69 mf/mg skin) and among 8 mg moxidectin treated participants 12 months after treatment (n = 12, 0.24–3.3 mf/mg skin, 0.5%–12% of pre-treatment value). The proportion of participants with undetectable levels of skin mf (Figure 6) was statistically significantly higher among moxidectin treated than ivermectin treated participants through month 12 for the 2 mg and 4 mg doses (p<0.02) and through month 18 for the 8 mg dose in both the mITT and the e-mITT population (p<0.05, Supporting Table S4).

The reduction in mean skin mf densities relative to pre-treatment densities was statistically significantly higher among moxidectin than ivermectin treated participants through month 12 for the 2 mg moxidectin dose (p<0.0001) and through month 18 for the 4 and 8 mg moxidectin dose in both the mITT and the e-mITT population (p<0.005, Supporting Table S3).

The average annual reduction in skin mf density from pre-treatment was 88% (median 94%, range 24%–99%, average 89% if the SOMRs are excluded) after ivermectin treatment, 97% (median 98%, range 81%–99%) after 2 mg moxidectin, 98% (median 99%, range 90%–99%) after 4 mg moxidectin and 98% (median 99%, range 96% to 99%) after 8 mg moxidectin.

### Results of histological assessment of excised palpable nodules

A total of 245 nodules were excised 18 months after treatment. Histological assessment showed 214 (87.3%) nodules to be onchocercal, including 46/56 (82.1%) nodules from ivermectin treated participants, 66/76 (86.8%) nodules from 2 mg moxidectin treated participants, 57/62 (91.9%) nodules from 4 mg moxidectin treated participants and 45/51 (88.2%) nodules from 8 mg moxidectin treated participants.

Non-onchocercal nodules (1–2/subject) included lipoma, lymphnodes and granulomas around foreign bodies. The types of non-onchocercal nodules include those found in other studies, but the frequency of non-onchocercal nodules was higher than observed in these studies [47], [48]. In 30/166 (18.1%) of participants who completed the study, the number of nodule sites palpated at Month 18 was lower than that palpated pre-treatment, suggesting that some of the nodules palpated pre-treatment were not onchocercal or onchocercal nodules whose resorption had been completed by Month 18.

The number of nodule sites palpated at month 18 was higher than pre-treatment by 1 in 23/163 (14.1%) and by 2 in 4/163 (2.5%) of participants who completed the study and agreed to nodule palpation. Investigator observations suggest that participants becoming more aware of nodule sites after the pre-treatment examination and nodule palpation for preparation of nodulectomies at month 18 follow up occurring under better lighting conditions contributed to the higher number of nodules palpated after than before treatment. An increase in the number of palpable onchocercal nodule sites due to new infections is also possible but unlikely to be significant given that some of the ‘additional’ nodule sites were already observed 1–6 months after treatment.

**Figure 8:**
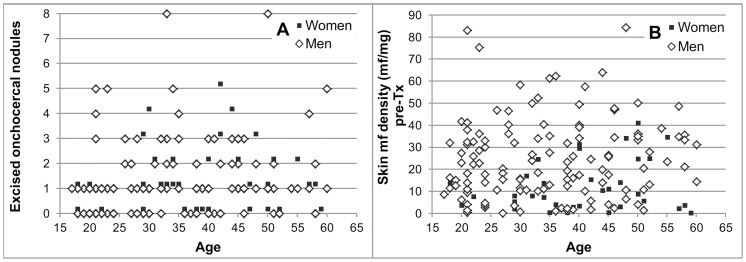
A. Number of excised onchocercal nodules by age and sex of participants. B. Pre-treatment skin mf density by age and sex of participants. Participants with 0 excised onchocercal nodules either had no palpable nodules or all excised nodules were non-onchocercal based on histological evaluation.

The number of excised onchocercal nodules/participant ranged from 0 (no palpable nodules or all palpable nodules were non-onchocercal) to 8. There was no trend suggesting a relationship between the age or sex of the participant and the number of excised onchocercal nodules (Figure 8 A), the skin mf density pre-treatment (Figure 8 B) or the skin mf level when the body surface area was taken into account [49] (data not shown).

**Figure 9:**
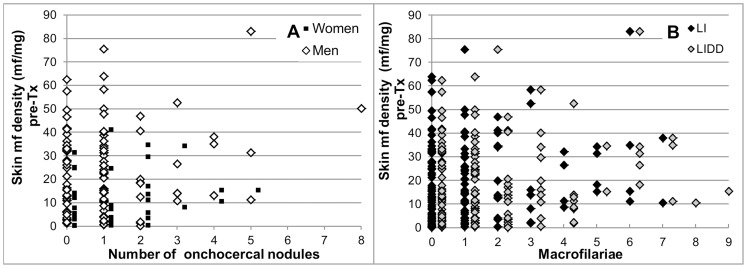
A. Skin microfilaria density pre-treatment by sex of participant and number of onchocercal nodules. B. Skin microfilaria density pre-treatment by number of live female macrofilariae and sum of live (LI) and dead, non-calcified female macrofilariae (LIDD). Only data from participants who had not more palpable nodule sites at month 18 than at pre-treatment and who had agreed to excision of all palpable nodules or who had no palpable nodules are included. For display reasons, the×axis maximum in Figure B was set to 9. The data from the man with 8 nodules in Figure A who had 13 live female macrofilariae and 3 dead, non-calcified female macrofilariae and 50.2 mf/mg skin pre-treatment are thus not displayed.

**Figure 10:**
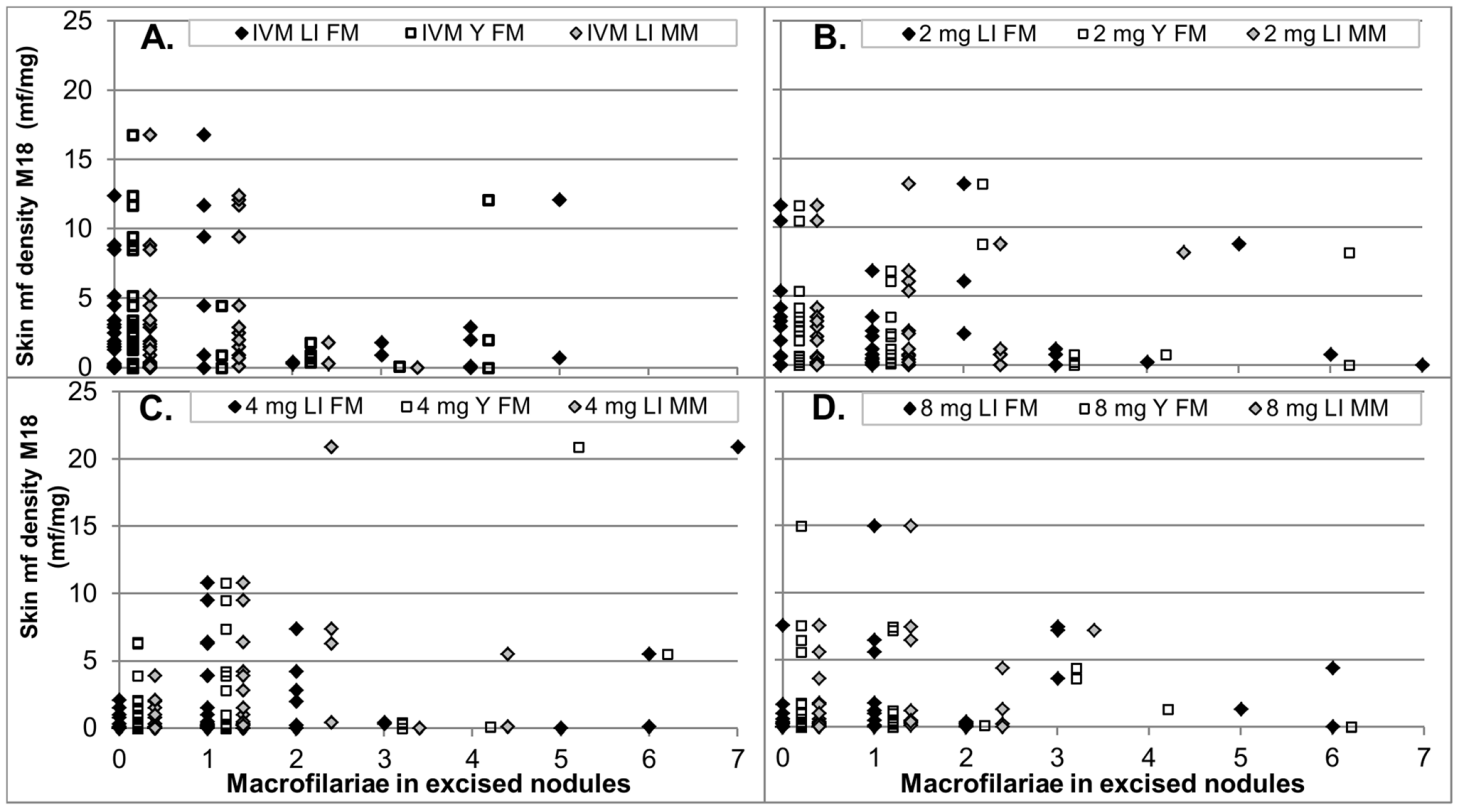
Number of live female macrofilaria, live young female macrofilaria and live male macrofilaria in excised nodules vs skin mf density 18 months after treatment by treatment. A. Ivermectin, B. 2×axis maximum was set to 7. The data for one participant treated with 2 mg moxidectin who had 13 live female macrofilaria and a skin mf density of 8.2 mf/mg are thus not displayed. Participants with 0 macrofilaria in excised nodules either had no palpable nodules or all excised nodules were non-onchocercal based on histological evaluation. Abbreviations: LI FM – live female macrofilariae, Y FM – live young female macrofilariae, LI MW – live male macrofilariae.

Figure 9A shows the pre-treatment skin mf densities by sex of the host vs. the number of onchocercal nodules for the 135 participants with a number of palpable nodule sites at month 18≤ the number of palpable nodule sites pre-treatment and who had agreed to excision of all palpable nodules, or who had no palpable nodules. High skin mf densities were observed in some participants with 0 or only 1 onchocercal nodule. This indicates that the palpable onchocercal nodules can represent only a small fraction of the nodules in the body as previously concluded by others (e.g. [50] and references therein). Pre-treatment skin mf densities of some participants with 0 or 1 excised onchocercal nodule were several times higher than those in some of the participants with ≥3 excised onchocercal nodules. This suggests that the fraction of onchocercal nodules accessible for excision varies significantly between individuals. Figure 9B shows for the same set of participants as for Figure 9A that there was no correlation between the number of live or the total number of live and dead female macrofilariae and the skin mf density pre-treatment. This suggests that the macrofilariae accessible through nodulectomy represent only a fraction of those present in the body as well as considerable inter-individual variability in this fraction. Since assessment of macrofilariae was only a secondary objective, nodules and macrofilariae were assessed by only one parasitologist (SKA). Given a level of agreement on the onchocercal nature of nodules and total number of female macrofilariae of 98% and 88%, respectively, between SKA and another parasitologist in a previous study [7], it is unlikely that reading of the slides from this study by a second parasitologist would have resulted in a significantly better correlation between number of nodules or total number of female macrofilariae and skin microfilariae than shown in Figure 9. Furthermore, a high degree of uncertainty about the true female adult parasite burden has been deduced previously from examination of the relationship between macrofilariae in palpable nodules and skin mf density in Burkina Faso and Liberia [51]. Figure 10 shows for each treatment group the skin mf density 18 months after treatment vs. the number of excised live female, live young female and live male macrofilariae. In all treatment groups, undetectable levels of skin mf as well as skin mf densities ≥5 mf/mg occurred in some participants with 0 or 1 live female or male macrofilaria as well as in some participants with ≥4 live female macrofilariae. This suggests again that the excised macrofilariae are not necessarily representative of the macrofilariae in the body.

Summary statistics of the results of the histological assessment are provided in Supporting Table S5. The percentage of female macrofilariae assessed as dead and/or dead and calcified was 50%, 36.5%, 32.2% and 27.7% in the ivermectin, 2 mg, 4 mg and 8 mg moxidectin treatment groups, respectively. Since a single dose of 150 µg/kg ivermectin does not kill macrofilariae (see e.g. [52]–[54]), this indicates a pre-treatment imbalance in the proportions of live and dead female macrofilariae between the ivermectin treatment group and the moxidectin treatment groups. Even if the excised macrofilariae were representative of all macrofilariae in the body, this imbalance makes conclusions about the relative effect of ivermectin and moxidectin on macrofilariae viability impossible. Consequently, the only conclusion on the effect of moxidectin on the macrofilariae the histology data support is that a single dose of 2 mg, 4 mg or 8 mg had neither sterilized all excised macrofilariae to month 18 nor killed all excised macrofilariae by 18 months follow up. Thus, the histology data do not provide any indication of the biological basis of the differences in the skin mf densities seen between treatment groups.

## Discussion

The clinical development plan for moxidectin includes (i) five pharmacokinetic and safety studies in healthy volunteers [21]–[25], (ii) the study reported here, (iii) a large (Phase 3) study in 1500 *O. volvulus* infected individuals ≥12 year old to determine adverse reactions, including those with a true frequency too low to have been detected in the study reported here, and to assess the relative efficacy of 8 mg moxidectin and ivermectin in individuals from different areas in Africa, and (iv) a paediatric pharmacokinetic and safety bridging study as per discussions with the European Medicines Agency.

The primary role of the study reported here was to determine in a small number of infected individuals whether moxidectin-associated Mazzotti reactions are infrequent enough or have a level of severity which allows to give moxidectin to several hundred people in the Phase 3 study. Review of the blinded data obtained to 1 month after treatment of the last cohort by the sponsors and an external advisory committee, as well as review by the external advisory committee with access to the treatment codes, resulted in the conclusion that 8 mg moxidectin is safe enough to initiate the Phase 3 study which compares 8 mg moxidectin to ivermectin. The investigators agreed based on their blinded assessment that the safety profile in the study was not different from what they had seen in previous studies with ivermectin using similarly close monitoring of participants for Mazzotti reactions. It is noteworthy that the publications of the safety data from these studies did, in contrast to Table 3 here, not include the total number of patients who had experienced at least one Mazzotti reaction (see e.g. [5], [55], [56]). The Phase 3 study has been completed (NCT00790998).

In the study reported here, there were no significant adverse drug reactions other than Mazzotti reactions, which is consistent with the data obtained in healthy volunteers [21]–[25].

The Mazzotti reactions pruritus, rash, increased pulse rate and decreased mean arterial pressure after 2 minutes standing still following ≥5 minutes supine occurred significantly more frequently among 8 mg moxidectin than ivermectin treated participants. The majority of these reactions were mild or moderate and all, including severe ones, resolved without treatment. This contributed to the decision to initiate the Phase 3 study.

While Fisher's exact test did not return a p value<0.05 for the comparison of the frequency of severe symptomatic postural hypotension (SSPH) after moxidectin and ivermectin treatment, consideration is given here also to SSPH. SSPH is a transient phenomenon observed at the Onchocerciasis Chemotherapy Research Center (OCRC) when a person cannot tolerate standing still for 2 minutes following ≥5 minutes supine (see OCRC Common Toxicity Criteria in Supporting Table S1). SSPH disappears rapidly after lying down [43] and OCRC staff observations of participant behaviour following an SSPH episode show that it does not interfere with resumption of their normal daily activities. In OCRC studies, SSPH incidence among participants treated with 150 µg/kg ivermectin was variable and sometimes high (e.g. 11% [57] or 22% [58]). In contrast, SSPH incidence reported from large scale use of ivermectin is in most cases low or 0, even in hyperendemic areas, when higher than standard doses were used or when significant decreases in standing MAP were measured [43], [59]–[64]. SSPH has furthermore not been an impediment to CDTI covering by now >75 million people [65]. OCRC experience shows that SSPH occurs when study participants get up (e.g. as part of the procedure to assess postural hypotension or after a bed rest) and immediately or shortly thereafter have to stand still (e.g. during the OCRC procedure or while urinating). SSPH is not usually seen when participants get up and move around naturally. This link between SSPH and standing still could explain the discrepancies between the results in OCRC studies and reports from large scale use of ivermectin. Underreporting by the treated individuals during large scale use is another possible explanation consistent with the OCRC observations that the symptoms of SSPH (severe dizziness, weakness, faintness) are shortlived and do not interfer with resumption of normal activities. This could result in SSPH not being a ‘memorable’ experience reported to the investigators. Consequently, the data to date do not suggest that a higher frequency of SSPH after moxidectin vs. ivermectin treatment, if shown to be statistically significant in the Phase 3 study (which used OCRC procedures), will be an impediment for evaluating moxidectin in community studies. These studies would allow to assess the potential significance of SSPH for mass treatment when appropriate advice is given to participants as in the early community studies of ivermectin [59].

Four participants treated with ivermectin did not show a decrease in skin mf levels meeting the criteria of adequate response to the microfilaricidal effect of ivermectin defined by Awadzi et al. [41]. Given that the participants were recruited from an area in Ghana without mass-treatment with ivermectin at the time of recruitment for this study, it is unlikely that this reflects drug pressure-induced selection of parasites with low susceptibility to ivermectin's microfilaricidal activity. It, thus, needs to be considered that the variability of the response of *O. volvulus* to the microfilaricidal activity is larger than had been observed by Awadzi et al. at the time they analysed their available data to derive criteria for adequate parasite response [41]. A larger variability of the response of *O. volvulus* to the embryostatic effect of ivermectin than considered ‘adequate’ in some studies [41], [66] has been deduced from a meta-analysis of the data from a large number of clinical and field studies [67].

In this study, a single dose of 2 mg, 4 mg or 8 mg moxidectin resulted in a significantly lower skin mf density and higher proportion of participants with undetectable skin mf earlier and for a longer period of time than ivermectin. Detectable levels of skin mf 18 months after treatment in 83%, 73% and 65% of participants treated with 2 mg, 4 mg and 8 mg, respectively, show that a single dose of moxidectin did not prevent skin repopulation with mf in all participants and thus did not kill or sterilize (permanently or to month 18) all macrofilariae in all participants. The histology data do not allow further conclusions about the biological basis of the long term differences in skin mf densities between the treatment groups, because of the pre-treatment imbalance between treatment groups in the percentage of dead and/or dead and calcified female macrofilaria (Supporting Table S5) and because the macrofilariae in the palpable onchocercal nodules were not representative of all macrofilariae in the body (Figures 9, 10). This is not surprising given the small sample size, chosen based on safety considerations, and not in view of allowing conclusions on treatment differences in the effect on the macrofilariae.

Determining a sample size sufficient to conclude with a pre-specified power and significance level that the effect of two treatments differs by at least a pre-specified amount requires a good estimate of the effect size in the comparator arm and the variance in the efficacy parameter (see e.g. [68], [69]). For macrofilariae, the comparator effect size may vary with the endemicity of the area and treatment history of the study area and participant. The effect size variance depends on biological and methodological factors which include: (i) inter-individual variability in the fraction of total nodules in the body which the excised nodules represent (Figure 9A), (ii) variability in the fraction of total worms of each category (female, male, live, dead, …) which the excised worms represent and which is not necessarily the same as the fraction of total nodules (Figure 9, Supporting Table S5), (iii) variability between the macrofilariae within each participant in the variable evaluated (e.g. age, reproductive activity [70]), (iv) method-specific factors such as for histology the extent to which the number of sections per nodule examined permits representative characterization of all worms in the nodule, quantitative vs. semiquantitative assessment, inter-observer variability (see e.g. [7], [71]) and (v) variability in macrofilariae exposure and susceptibility to the drug (e.g. macrofilariae age dependent, nodule location dependent). Analysis of the pooled raw data from different past studies may help quantitate some of these variabilities and provide the basis for calculating sample sizes for future studies. This may also help resolve the question of the extent to which cumulative doses of ivermectin affect macrofilariae reproductive capacity and viability. As pointed out for the reproductive capacity, this has significant implications for whether, where and how elimination of *O. volvulus* infection with ivermectin can be achieved [72].

The pre-treatment imbalance in this study in the percentage of female macrofilariae assessed as dead and/or dead and calcified between the ivermectin and moxidectin treatment groups (Supporting Table S5) shows the importance of stratification of study participants for randomization to treatment groups by number or at least proportion of dead and live macrofilariae in the palpable nodules (determined with a non-invasive method such as ultrasonography [73]) to reduce the probability of false conclusions from post-treatment data.

Given the absence of data on the biological basis for the low skin mf levels 12–18 months after moxidectin treatment, the relative value of moxidectin and ivermectin for reducing disease transmission will be considered without any assumptions about this basis. The transmission model developed by Duerr and coworkers [12], [13] includes examination of the effect of a drug which reduces skin mf density irrespective of the effect on the macrofilariae. It shows that mass-treatment with a skin mf reducing drug can lead to elimination by increasing the threshold biting rate (TBR), i.e. the annual biting rate (ABR, number of vectors taking a blood meal from one person/year) below which onchocerciasis cannot remain endemic. With increasing efficacy of the intervention, quantified as the annual average reduction (AAR) in skin mf density in the population, the TBR increases in a non-linear manner: in an area with a TBR without intervention of 700 bites per person per year, the model predicts a TBR of approximately 1200, 2000 and 5000 when the AAR is around 65%, 80% and 95%, respectively. The AAR after treatment with ivermectin and 8 mg moxidectin in this study were 88% and 98%, respectively. The AARs after treatment of communities would be different because of different distribution of pre-treatment skin mf densities and treatment coverage. However, and provided the relative superiority of 8 mg moxidectin over ivermectin is confirmed in the Phase 3 study, moxidectin would have a higher AAR than ivermectin suggesting that, according to this model, moxidectin could lead to elimination in areas with higher ABRs than ivermectin.

CDTI occurs at a time chosen by the community and is in many cases furthermore dependent on logistical conditions (e.g. availability of funds for activities the public health system needs to conduct to enable CDTI). This results in CDTI in areas with seasonal transmission not always happening within the time window optimal for achieving elimination, i.e. a time that results in the lowest skin mf levels in the population when the vector population is largest. The longer period of undetectable skin mf levels after moxidectin compared to ivermectin would reduce the negative impact of community treatment occurring outside the optimal time window on transmission.

## Supporting Information

Table S1 **Onchocerciasis Chemotherapy Research Center (OCRC) Common Toxicity Criteria.**
(DOC)

Table S2 **Number (%) of subjects with Mazzotti reactions for which Fisher's exact test between at least one moxidectin treatment group and the ivermectin treatment group yielded a p-value<0.05 and for SSPH by severity of infection pre-treatment (mITT population).**
(DOC)

Table S3 **Results of statistical analysis of the change in skin microfilaria density (mf/mg skin) from pre-treatment.**
(DOC)

Table S4 **Results of statistical analysis of the percentage of participants with undetectable levels of skin microfilariae.**
(DOC)

Table S5 **Results of histological assessment of excised onchocercal palpable nodules.**
(DOC)

Checklist S1 **CONSORT checklist.**
(DOCX)

Protocol S1 **Study protocol: Amendment 4 under which the study was initiated and Amendment 5 in track changes from Amendment 4.** Relative to Amendment 4, Amendment 5: • Specified that women already receiving parenterally administered contraceptive were excluded from the protocol requirement for protocol defined parenterally administered contraceptive (DMPA injections) • Allowed Informed Consent (to be obtained in the villages prior to admission, i.e., before transport to the study center) to be obtained earlier than within the time frame specified for screening procedures. • Clarified that the NCI CTC criteria were applicable only for AEs not included in the OCRC Common Toxicity Criteria (see Table S1) and attached more of these criteria to the protocol • Specified that back-up samples for pharmacokinetic analyses could be shipped to the PK laboratory as soon as confirmation had been obtained that the ‘front samples’ had been received and provided for the appropriate procedures, and adjusted the instructions for timing of shipments to allow arrangements with the carrier. • Provided for information previously to be documented by the unblinded pharmacist on paper to be reduced and documented on specifically provided labels.
(PDF)
