# Supplementary material for: A Randomized, Single-Ascending-Dose, Ivermectin-Controlled, Double-Blind Study of Moxidectin in Onchocerca volvulus Infection
Source: PLoS Negl Trop Dis. 2014 Jun 26;8(6):e2953. doi: 10.1371/journal.pntd.0002953 (PMC4072596; doi:10.1371/journal.pntd.0002953)
Supplement: Table S1 — Onchocerciasis Chemotherapy Research Center (OCRC) Common Toxicity Criteria. (DOC) [file pntd.0002953.s001.doc]

| **Grade** | | | | | |
| --- | --- | --- | --- | --- | --- |
| **Toxicity** | **0** | **1** | **2** | **3** | **4** |
| **MAZZOTTI TOXICITY CRITERIA (MTC) SYSTEMIC (1)** | | | | | |
| Itching | None | Mild | Moderate or ‘Severe’ but with only occasional scratching | ‘Severe’ and with fairly vigorous scratching “Windmill Effect” | ‘Severe’ and with restlessness, agitation, loss of epithelium or prolonged vigorous scratching |
| Note: ‘Severe’ is patient’s perception of grade of discomfort. “Windmill Effect” = arms in fairly continuous motion with scratching | | | | | |
| Headache | None | Mild | Moderate or ‘Severe’ but patient comfortable | ‘Severe’ and with obvious distress | Unbearable |
| Note: ‘Severe’ is patient’s perception of grade of discomfort | | | | | |
| Joint pain (arthralgia) | None | Mild | Moderate or ‘Severe’ but without change in gait or function | ‘Severe’ and with a definite limp or change in function due to joint pain | ‘Severe’ and with marked interference with motion or function (commonly “Pillar of Salt” Effect) |
| Note: ‘Severe’ is patient’s perception of grade of discomfort. “Pillar of salt Effect” = rooted to the spot and unable to walk due to severe pain | | | | | |
| Muscle pain (myalgia) | None | Mild | Moderate or ‘Severe’ but without change in gait or function | ‘Severe’ and with a definite limp or change in function due to muscle pain | ‘Severe’ and with marked interference with motion or function (commonly “Pillar of Salt” Effect) |
| Note: ‘Severe’ is patient’s perception of grade of discomfort. “Pillar of salt Effect” = rooted to the spot and unable to walk due to severe pain | | | | | |
| Gland pain | None | Mild | Moderate or ‘Severe’ but without change in gait or function | ‘Severe’ and with a definite limp or change in function due to gland pain (“Hydrocele Gait” may be present) | ‘Severe’ and with marked interference with motion or function (commonly “Pillar of Salt” Effect; “Knee Elbow Position”) |
| Note: ‘Severe’ is patient’s perception of grade of discomfort. “Pillar of salt Effect” = rooted to the spot and unable to walk due to severe pain  “Hydrocele Gait” = Walking on a broad base with trunk slightly flexed. | | | | | |
| Gland tenderness | None | Very firm pressure needed to elicit pain | Moderate pressure elicits pain | Very light touch elicits severe pain | Patient refuses palpation on account of severe pain |
| Note: Assessment carried out by Medical Officer | | | | | |
| Rash | None | < ⅓ body surface | ⅓-< ⅔ body surface | ≥⅔ body surface | - |
| Note: Assessment carried out by Medical Officer | | | | | |
| **MTC SYSTEMIC (2)** | | | | | |
| Temperature increase | <38^0^C | 38.0 - 39.0°C | 39.1 - 40.0°C | > 40.0°C for < 24hrs | > 40.0°C > 24hrs |
| Increase in Pulse Rate in beats per minute | 0 -20 | >20-<36 | ≥36-<52 | ≥52-<68 | ≥68 |
| Note: Readings taken lying as well as after standing for 2 minutes | | | | | |
| Fall in Mean Arterial Pressure | 0-20 | >20-<25 | ≥25-<30 | ≥30-<35 | ≥35 or CNS* |
| Note: Readings are taken lying as well as after standing for 2 minutes. * CNS means Could Not Stand long enough for pressure to be taken due to severe postural hypotension | | | | | |
| Increase in Respiratory Rate per minute | 0-<6 | ≥6-<12 | ≥12-<18 | ≥18-<24 | ≥24 |
| Note: Contributes little to overall score | | | | | |
| **MTC OTHER** | | | | | |
| Lymphatics | Normal | Mild lymphedema | Moderate lymphedema requiring compression; lymphocyst | Severe lymphedema limiting function; lymphocyst requiring surgery | Severe lymphedema limiting function with ulceration |
| Note: An acute brawny edema of one or more limbs a manifestation of the Mazzotti reaction | | | | | |
| Facial swelling | None | Mild swelling | Moderate swelling | Severe with eyes completely shut | - |
| Note: A fairly common manifestation of the Mazzotti reaction | | | | | |
| Neuropathy-sensory | Normal | Loss of deep tendon reflexes or paresthesia (including tingling) but not interfering with function | Objective sensory loss or paresthesia (including tingling), interfering with function, but not interfering with activities of daily living | Sensory loss or paresthesia interfering with activities of daily living | Permanent sensory loss that interferes with function |
| Note: Peripheral Sensory Phenomena (PSP) can be a manifestation of the Mazzotti reaction | | | | | |
| Rigors, chills | None | Mild, requiring symptomatic treatment (e.g., blanket) or non-narcotic medication | Severe and/or prolonged, requiring narcotic medication | not responsive to narcotic medication | - |
| Fatigue (lethargy, malaise, asthenia) | None | Increased fatigue over baseline, but not altering normal activities | Moderate (e.g., decrease in performance status by 1 ECOG level or 20% Karnofsky or *Lansky*) or causing difficulty performing some activities | Severe (e.g., decrease in performance status by ≥2 ECOG levels or 40% Karnofsky or *Lansky*) or loss of ability to perform some activities | Bedridden or disabling |
| Note: Performance status scales presented below | | | | | |
| **OTHER SYSTEMIC TOXICITY** | | | | | |
| Anorexia | None | Loss of appetite | oral intake significantly decreased | Requiring IV fluids | Requiring feeding tube or parenteral nutrition |
| Nausea | None | Able to eat | oral intake significantly decreased | no significant intake, requiring IV fluids | - |
| ‘Bitter’ mouth | None | Able to eat | oral intake significantly decreased | no significant intake, requiring IV fluids | - |
| Vomiting | None | 1 episode in 24 hours over pretreatment | 2-5 episodes in 24 hours over pretreatment | ≥6 episodes in 24 hours over pretreatment; or need for IV fluids | Requiring parenteral nutrition; or physiologic consequences requiring intensive care; hemodynamic collapse |
| Diarrhea | None | Increase of < 4 stools/day over pre-treatment | Increase of 4-6 stools/day, or nocturnal stools | Increase of ≥7 stools/day or incontinence; or need for parenteral support for dehydration | Physiologic consequence requiring intensive care; or hemodynamic collapse |
| Abdominal pain or cramping | None | Mild pain not interfering with function | Moderate pain: pain or analgesics interfering with function, but not interfering with activities of daily living | Severe pain: pain or analgesics severely interfering with activities of daily living | Disabling |
| Cough | Absent | Mild, relieved by non-prescription medication | Requiring narcotic antitussive | Severe cough or coughing spasms, poorly controlled or unresponsive to treatment | - |
| Chest pain (non-cardiac and non-pleuritic) | None | Mild pain not interfering with function | Moderate pain: pain or analgesics interfering with function, but not interfering with activities of daily living | Severe pain: pain or analgesics severely interfering with activities of daily living | Disabling |
| Dyspnoea (shortness of breath) | Normal | - | Dyspnea on exertion | Dyspnea at normal level of activity | Dyspnea at rest or requiring ventilator support |
| Palpitations | None | Present | - | - | - |
| Note: Grade palpitations only in the absence of a documented arrhythmia. | | | | | |
| Waistpain/Backache | None | Mild pain not interfering with function | Moderate pain: pain or analgesics interfering with function, but not interfering with activities of daily living | Severe pain: pain or analgesics severely interfering with activities of daily living | Disabling |
| Neckpain | None | Mild pain not interfering with function | Moderate pain: pain or analgesics interfering with function, but not interfering with activities of daily living | Severe pain: pain or analgesics severely interfering with activities of daily living | Disabling |
| Bodily pain/aches | None | Mild pain not interfering with function | Moderate pain: pain or analgesics interfering with function, but not interfering with activities of daily living | Severe pain: pain or analgesics severely interfering with activities of daily living | Disabling |
| Dizziness/ lightheadedness | None | Not interfering with function | Interfering with function, but not interfering with activities of daily living | Interfering with activities of daily living | Bedridden or disabling |
| Note: Grade only if unassociated with hypotension | | | | | |
| Constipation | None | Requiring stool softener or dietary modification or increased mobility | Requiring laxatives | Obstipation requiring manual evacuation or enema | Obstruction or toxic megacolon |
| Dyspepsia/heartburn | None | Mild | Moderate | Severe | - |
| Insomnia | Normal | Occasional difficulty sleeping not interfering with function | Difficulty sleeping interfering with function, but not interfering with activities of daily living | Frequent difficulty sleeping, interfering with activities of daily living | - |
| Note: This toxicity is graded when insomnia is related to treatment. (Sleepless for no reason). If pain or other symptoms interfere with sleep do NOT grade as insomnia. | | | | | |
| Bone pain | None | Mild pain not interfering with function | Moderate pain: pain or analgesics interfering with function, but not interfering with activities of daily living | Severe pain: pain or analgesics severely interfering with activities of daily living | Disabling |
| Catarrh | Absent | Present | - | - | - |
| Earache (otalgia) | None | Mild pain not interfering with function | Moderate pain: pain or analgesics interfering with function, but not interfering with activities of daily living | Severe pain: pain or analgesics severely interfering with activities of daily living | Disabling |
| Note: It is essential to determine that otalgia is not present pretreatment | | | | | |
| Toothache | None | Mild pain not interfering with function | Moderate pain: pain or analgesics interfering with function, but not interfering with activities of daily living | Severe pain: pain or analgesics severely interfering with activities of daily living | Disabling |
| Note: It is essential to determine that toothache is not present pretreatment | | | | | |
| Weight gain | < 5% | 5 - <10% | 10 - <20% | ≥ 20% | - |
| Weight loss | < 5% | 5 - <10% | 10 - <20% | ≥20% | - |
| Erectile impotence | Normal | Mild (erections impaired but satisfactory) | Moderate (erections impaired, unsatisfactory for intercourse) | no erections | - |
| Libido | Normal | Decrease in interest | Severe loss of interest | - | - |
| Dysmenorrhea | None | Mild pain not interfering with function | Moderate pain: pain or analgesics interfering with function, but not interfering with activities of daily living | Severe pain: pain or analgesics severely interfering with activities of daily living | Disabling |
| Irregular menses  (change from baseline) | Normal | Occasionally irregular or lengthened interval, but continuing menstrual cycles | Very irregular, but continuing menstrual cycles | Persistent amenorrhea | - |
| **MTC OCULAR** (Modified from Hero, M., A. C. Bird & K. Awadzi. (1992). Quantification of the ocular reactions to microfilaricides in the chemotherapy of onchocerciasis. Eye **6**(Pt 1): 93-96.) | | | | | |
| Ocular discomfort | Normal | mild: not interfering with function | Moderate: interfering with function, but not interfering with activities of daily living | Interfering with activities of daily living | - |
| Ocular itching | Normal | mild: not interfering with function | Moderate: interfering with function, but not interfering with activities of daily living | Interfering with activities of daily living | - |
| Tearing (watery eyes) | None | mild: not interfering with function | Moderate: interfering with function, but not interfering with activities of daily living | Interfering with activities of daily living | - |
| Vision- photophobia | Normal | - | Symptomatic and interfering with function, but not interfering with activities of daily living | Symptomatic and interfering with activities of daily living | - |
| Visual acuity | Unchanged or 6/4-6/6 | Loss of 1 line | Loss of 2 lines | Loss of 3 lines | Loss of >3 lines |
| Visual fields - IV/4e kinetic | None | 1-12 | 13-24 | 25-48 | 49-72 |
| Visual fields - I4/e kinetic | None | 1-12 | 13-24 | 25-36 | 37-60 |
| Visual fields - I/2e I2/e | None | 1-12 | 13-20 | 21-28 | 29-36 |
| Visual fields - I2/e | None | 3-12 | 13-20 | 21-35 | 36-52 |
| Note: Based on the number of missed targets | | | | | |
| Anterior Segment inflammation (ASI) Conjunctivitis | None | - | Hyperaemia | - | Chemosis |
| ASI - Limbitis-vascular | None | - | Dilated capillaries | - | Limbal oedema |
| ASI - Limbitis-globular infiltrates | None | 1-5 | 6-10 | 11-20 | >20 |
| ASI - Corneal punctate opacities | None | 1-5 | 6-10 | 11-20 | >20 |
| Anterior uveitis - no of cells/field * | None | 1-10 | 11-20 | 21-40 | >40 |
| Note: * Light beam at 45 degrees; slit 2mm high by 0.2mm wide. | | | | | |
| Anterior uveitis - Flare** | None | Seen with no filter | Seen with filter 1 | Seen with filter 2 | Plasmoid aqueous |
| Note: ** Light beam at 45 degrees; slit 2mm high by 0.1mm wide. Filters 1 and 2 are neutral density filters in-built into the optical column of the slit lamp | | | | | |
| Posterior segment - Optic neuritis (colour film) - Hyperaemia | None | Sectorial | Overall | - | - |
| Posterior segment  Optic neuritis (colour film) Swelling | None | - | - | Sectorial | Overall |
| Angiographic leakage - Within disc margin | None | Sectorial | Overall | - | - |
| Angiographic leakage - Beyond disc margin | None | - | - | Sectorial | Overall |
| Optic atrophy - Colour film | None | Linear nerve fibre loss | Sectorial atrophy | Overall atrophy | Atrophy with pigment |
| Optic atrophy - Red free | None | Linear nerve fibre loss | Sectorial fibre loss | Total loss | Total loss with pigment |
| Pigment Epithelial Atrophy - Distribution | None | Temporal to macula | More than temporal | Continuous round macula | Whole of macula |
| Pigment Epithelial Atrophy - Intensity | None | RPE mottling only | RPE mottling with <50% atrophy | RPE mottling with ≥50% atrophy | RPE mottling with hypertrophy |
| Other onchocercal lesions - Cotton wool spots | Absent | Present | - | - | - |
| Other onchocercal lesions - Vasculitis | Absent | Present | - | - | - |
| Other onchocercal lesions - Haemorrhage | Absent | Present | - | - | - |
| Note: Once an event has occurred the score is retained at subsequent visits whether the lesion persists or not. | | | | | |
| **OTHER OCULAR TOXICITY** | | | | | |
| Ocular discharge | None | Present | - | - | - |
| Vision - blurred vision | Normal | - | Symptomatic and interfering with function, but not interfering with activities of daily living | Symptomatic and interfering with activities of daily living | - |
| Vision - flashing lights / floaters | Normal | Mild, not interfering with function | Symptomatic and interfering with function, but not interfering with activities of daily living | Symptomatic and interfering with activities of daily living | - |
| Vision- night blindness (nyctalopia) | Normal | Present but asymptomatic | Symptomatic and interfering with function, but not interfering with activities of daily living | Symptomatic and interfering with activities of daily living | - |
| ALLERGIC REACTION commonly to FLUORESCEIN | | | | | |
| Allergic reaction/ hypersensitivity (including drug fever) | None | Transient rash, drug fever < 38°C (<100.4°F) | urticaria, drug fever ≥ 38°C (≥100.4°F), and/or asymptomatic bronchospasm | Symptomatic bronchospasm, requiring parenteral medication(s), with or without urticaria; allergy-related edema/angioedema | Anaphylaxis |
| Sneezing | Absent | Present | - | - | - |
| Dry cough, no bronchospasm | Absent | Present | - | - | - |
| Urticaria  (hives, welts, wheals) | None | Requiring no medication | requiring PO or topical treatment or IV medication or steroids for <24 hours | requiring IV medication or steroids for ≥24 hours | - |
| Note: Also anorexia, nausea, vomiting , rigors, chills | | | | | |
| **ELECTROCARDIOGRAPHY** | | | | | |
| Conduction abnormality / Atrioventricular heart block | None | Asymptomatic, not requiring treatment (e.g., Mobitz type I second-degree AV block, Wenckebach) | Symptomatic, but not requiring treatment | symptomatic and requiring treatment (e.g., Mobitz type II second-degree AV block, third-degree AV block) | Life-threatening (e.g., arrhythmia associated with CHF, hypotension, syncope, shock) |
| Nodal / junctional arrhythmia / dysrhythmia | None | Asymptomatic, not requiring treatment | symptomatic, but not requiring treatment | symptomatic and requiring treatment | Life-threatening (e.g., arrhythmia associated with CHF, hypotension, syncope, shock) |
| Palpitations | None | Present | - | - | - |
| Note: Grade palpitations only in the absence of a documented arrhythmia. | | | | | |
| Prolonged QTc interval (QTc > 0.48 seconds) | None | Asymptomatic, not requiring treatment | symptomatic, but not requiring treatment | symptomatic and requiring treatment | Life-threatening (e.g., arrhythmia associated with CHF, hypotension, syncope, shock) |
| Sinus bradycardia | None | Asymptomatic, not requiring treatment | symptomatic, but not requiring treatment | symptomatic and requiring treatment | Life-threatening (e.g., arrhythmia associated with CHF, hypotension, syncope, shock) |
| Sinus tachycardia | None | Asymptomatic, not requiring treatment | symptomatic, but not requiring treatment | symptomatic and requiring treatment of underlying cause | - |
| Supraventricular arrhythmias (SVT/atrial fibrillation/ flutter) | None | Asymptomatic, not requiring treatment | symptomatic, but not requiring treatment | symptomatic and requiring treatment | Life-threatening (e.g., arrhythmia associated with CHF, hypotension, syncope, shock) |
| Vasovagal episode | None | - | present without loss of consciousness | present with loss of consciousness | - |
| Ventricular arrhythmia (PVCs/ bigeminy/ trigeminy/ ventricular tachycardia) | None | Asymptomatic, not requiring treatment | symptomatic, but not requiring treatment | symptomatic and requiring treatment | Life-threatening (e.g., arrhythmia associated with CHF, hypotension, syncope, shock) |
| Peaking of T wave | None | Mild-moderate | Marked | - | - |
| Cardiac- ischemia/infarction | None | Non-specific T-wave flattening or changes | Asymptomatic, ST- and T- wave changes suggesting ischemia | angina without evidence of infarction | acute myocardial infarction |
| **HAEMATOLOGICAL** | | | | | |
| G6PD | Normal | Partial defect | Total defect | - | - |
| Hemoglobin | WNL | 10-<25% reduction | 25-<50% reduction | 50-<75% reduction | ≥75% |
| Leukocytes (total WBC) | WNL | < LLN – 2.5x10^9^ /L | 1.5--< 2.5 x10^9^ /L | 1.0 - < 1.5 x10^9^/L | < 1.0 x 10^9^ /L |
|  | WNL | > 11.3-19.0 x 10^9^ /L | >19.0-38.0 x 10^9^ /L | >38.0-57.0 x 10^9^ /L | > 57.0 x 10^9^ /L |
| Note: Contributory factors include Mazotti reaction (leucocytosis), coincidental infection (exclude) drug effect | | | | | |
| Neutrophils/granulocytes (ANC/AGC) | WNL | ≥1500 - <2000/mm^3^ | ≥1000 - <1500/mm^3^ | ≥500 - <1000/mm^3^ | < 500/mm^3^ |
|  |  | >6080-12160/mm^3^ | >12160-24320/mm^3^ | >24320-36480/mm^3^ | >36480/mm^3^ |
| Note: Contributory factors include Mazotti reaction (neutrophilia), coincidental infection (exclude) drug effect | | | | | |
| Lymphocytes | WNL | <LLN – 1000/mm^3^ | ≥500 - <1000/mm^3^ | <500/mm^3^ | - |
|  |  | >6365-12730/mm^3^ | >12730-25460/mm^3^ | >25460-38190/mm^3^ | >38190/mm^3^ |
| Note: Contributory factors include Mazotti reaction (initial lymphopenia, lymphocytosis), coincidental infection (exclude) drug effect | | | | | |
| Eosinophils | WNL | 20-26/mm^3^ | ≥14-19/mm^3^ | ≥7-13/mm^3^ | <7/mm^3^ |
|  |  | >1425-2850/mm^3^ | >2850-5700/mm^3^ | >5700-8550/mm^3^ | >8550/mm^3^ |
| Note: Contributory factors include Mazotti reaction (initial eosinopenia, eosinophilia), coincidental infection (exclude) drug effect | | | | | |
| Platelets | WNL | < LLN - <75.0 x 10^9^ /L | ≥50.0 - < 75.0 x 10^9^ /L | ≥10.0 - < 50.0 x 10^9^ /L | < 10.0 x 10^9^ /L |
|  |  | < LLN – 75000/mm^3^ | ≥50000 - < 75000/mm^3^ | ≥10000 - < 50000/mm^3^ | < 10000/mm^3^ |
| Hemolysis (e.g., immune hemolytic anemia, drug-related hemolysis, other) | None | Only laboratory evidence of hemolysis [e.g., direct antiglobulin test (DAT, Coombs’) schistocytes] | Evidence of red cell destruction and ≥ 2gm decrease in hemoglobin, no transfusion | requiring transfusion and/or medical intervention (e.g., steroids) | catastrophic consequences of hemolysis (e.g., renal failure, hypotension, bronchospasm, emergency splenectomy) |
| **BIOCHEMICAL** | | | | | |
| Hyponatremia | WNL | <LLN – 130 mmol/L | - | 120 - <130 mmol/L | <120 mmol/L |
| Hypernatremia | WNL | >ULN - 150 mmol/L | >150 - 155 mmol/L | >155 - 160 mmol/L | >160 mmol/L |
| Hypokalemia | WNL | <LLN - 3.0 mmol/L | - | 2.5 - <3.0 mmol/L | <2.5 mmol/L |
| Hyperkalemia | WNL | >ULN - 5.5 mmol/L | > 5.5 - 6.0 mmol/L | > 6.0 - 7.0 mmol/L | > 7.0 mmol/L |
| Bicarbonate | WNL | <LLN - 16 mEq/dl | 11 - 15 mEq/dl | 8 - 10 mEq/dl | <8 mEq/dl |
| Hyperuricemia | WNL | >ULN - ≤ 590 μmol/L without physiologic consequences | - | >ULN - ≤ 590 μmol/L with physiologic consequences | > 590 μmol/L |
| Note: Also consider Renal failure, Creatinine, Potassium. | | | | | |
| Creatinine | WNL | > ULN - 1.5 x ULN | > 1.5 - 3.0 x ULN | > 3.0 - 6.0 x ULN | > 6.0 x ULN |
| SGPT (ALT) (serum glutamic pyruvic transaminase) | WNL | > ULN - 2.5 x ULN | > 2.5 - 5.0 x ULN | > 5.0 - 20.0 x ULN | > 20.0 x ULN |
| Note: Elevations occur as part of the Mazzotti reaction but rarely reach 5 x ULN and usually last for less than 30 days | | | | | |
| SGOT (AST) (serum glutamic oxaloacetic transaminase) | WNL | > ULN - 2.5 x ULN | > 2.5 - 5.0 x ULN | > 5.0 - 20.0 x ULN | > 20.0 x ULN |
| Note: Elevations occur as part of the Mazzotti reaction but rarely reach 5 x ULN and usually last for less than 30 days | | | | | |
| GGT (γ - Glutamyl transpeptidase) | WNL | > ULN - 2.5 x ULN | > 2.5 - 5.0 x ULN | > 5.0 - 20.0 x ULN | > 20.0 x ULN |
| Note: Elevations occur as part of the Mazzotti reaction but rarely reach 5 x ULN and usually last for less than 30 days | | | | | |
| LDH (Lactate dehydrogenase) | WNL | > ULN - 2.5 x ULN | > 2.5 - 5.0 x ULN | > 5.0 - 20.0 x ULN | > 20.0 x ULN |
| Note: Elevations occur as part of the Mazzotti reaction but rarely reach 5 x ULN and usually last for less than 30 days | | | | | |
| Alkaline phosphatase | WNL | > ULN - 2.5 x ULN | > 2.5 - 5.0 x ULN | > 5.0 - 20.0 x ULN | > 20.0 x ULN |
| Total Bilirubin | WNL | > ULN - 1.5 x ULN | > 1.5 - 3.0 x ULN | > 3.0 - 10.0 x ULN | > 10.0 x ULN |
| Hypoalbuminemia | WNL | <LLN - 30 g/L | ≥20 - <30 g/L | <20 g/L | - |
| Hypoglycemia | WNL | <LLN - 3.0 mmol/L | 2.2 - < 3.0 mmol/L | 1.7 - < 2.2 mmol/L | < 1.7 mmol/L |
| Hyperglycemia | WNL | > ULN - 8.9 mmol/L | > 8.9 - 13.9 mmol/L | > 13.9 - 27.8 mmol/L | > 27.8 mmol/L or ketoacidosis |
| Hypercholesterolemia | WNL | > ULN - 7.75 mmol/L | > 7.75 - 10.34 mmol/L | >10.34 - 12.92 mmol/L | > 12.92 mmol/L |
| Hypertriglyceridemia | WNL | > ULN - 2.5 x ULN | > 2.5 - 5.0 x ULN | > 5.0 - 10 x ULN | > 10 x ULN |
| **Urinalysis** | | | | | |
| Proteinuria (Multistix 10 SG) | Normal or trace | 1+ | 2+ to 3+ | 4+ | Nephrotic syndrome |
| Hematuria (in absence of vaginal bleeding) | None | Microscopic only | Intermittent gross bleeding | Persistent gross bleeding or clots; may require catheterization or instrumentation or transfusion | Open surgery or necrosis or deep bladder ulceration |
| Microfilariae/10ml | None | 1-20 | >20 |  |  |

Abbreviations: LLN Lower limit of Normal Range, ULN Upper Limit of Normal Range, WNL within normal limits
