# Supplementary material for: A Randomized, Single-Ascending-Dose, Ivermectin-Controlled, Double-Blind Study of Moxidectin in Onchocerca volvulus Infection
Source: PLoS Negl Trop Dis. 2014 Jun 26;8(6):e2953. doi: 10.1371/journal.pntd.0002953 (PMC4072596; doi:10.1371/journal.pntd.0002953)
Supplement: Table S2 — Number (%) of subjects with Mazzotti reactions for which Fisher's exact test between at least one moxidectin treatment group and the ivermectin treatment group yielded a p-value<0.05 and for SSPH by severity of infection pre-treatment (mITT population). (DOC) [file pntd.0002953.s002.doc]

| Type of Mazzotti reaction | Intensity of infection | Ivermectin  **n** (%) | 2 mg moxidectin  **n** (%) | 4 mg moxidectin  **n** (%) | 8 mg moxidectin  **n** (%) |
| --- | --- | --- | --- | --- | --- |
| Subjects treated | Any | 45 | 44 | 45 | 38 |
|  | Mild | 12 | 10 | 11 | 12 |
|  | Moderate | 12 | 11 | 11 | 11 |
|  | Severe | 21 | 23 | 23 | 15 |
| Any MAZ | Any | 43 (95.6) | 38 (86.4) | 45 (100) | 37 (97.4) |
|  | Mild | 12 (100) | 9 (90.0) | 12 (100) | 12 (100) |
|  | Moderate | 11 (91.7) | 8 (72.7) | 11 (100) | 11 (100) |
|  | Severe | 20 (95.2) | 21 (91.3) | 23 (100) | 14 (93.3) |
| Pruritus | Any | 25 (55.6) | 20 (45.5) | 27 (60.0) | 33 (86.8)* |
|  | Mild | 8 (66.7) | 4 (40.0) | 6 (54.5) | 11 (91.7) |
|  | Moderate | 7 (58.3) | 6 (54.5) | 5 (45.4) | 8 (72.7) |
|  | Severe | 10 (47.6) | 10 (43.5) | 16 (69.6) | 14 (93.3) |
| Rash | Any | 19 (42.2) | 18 (40.9) | 23 (51.1) | 24 (63.2)* |
|  | Mild | 4 (33.3) | 3 (30.0) | 4 (36.4) | 5 (50.0) |
|  | Moderate | 4 (33.3) | 6 (54.5) | 4 (36.4) | 6 (54.5) |
|  | Severe | 10 (47.6) | 9 (39.1) | 15 (65.2) | 12 (80.0) |
| Increase in pulse rate (standing^1^) | Any | 16 (35.6) | 21 (47.7) | 14 (31.1) | 23 (60.5)* |
|  | Mild | 4 (33.3) | 1 (10.0) | 0 | 5 (41.7) |
|  | Moderate | 5 (41.7) | 4 (36.4) | 5 (45.5) | 8 (72.7) |
|  | Severe | 7 (33.3) | 16 (69.6) | 9 (39.1) | 10 (66.7) |
| MAP decrease (standing^2^) | Any | 12 (26.7) | 8 (18.2) | 12 (26.7) | 23 (60.5)* |
|  | Mild | 2 (16.7) | 0 | 1 (9.1) | 4 (33.3) |
|  | Moderate | 2 (16.7) | 0 | 2 (18.2) | 8 (72.7) |
|  | Severe | 8 (38.1) | 8 (34.8) | 8 (39.1) | 10 (66.7) |
| Grade 4 MAP decrease SAPH^3^ | Any | 0 | 3 (6.8) | 1 (2.2) | 6 (15.8)* |
|  | Mild | 0 | 0 | 0 | 0 |
|  | Moderate | 0 | 0 | 0 | 1 (9.1) |
|  | Severe | 0 | 3 (13.0) | 1 4.5) | 5 (33.3) |
| Grade 4 MAP decrease SSPH^4^ | Any | 1 (2.2) | 1 (2.3) | 4 (8.9) | 5 (13.2) |
|  | Mild | 0 | 0 | 0 | 1 (8.3) |
|  | Moderate | 0 | 0 | 0 | 3 (27.3) |
|  | Severe | 1 (4.8) | 1 (4.5) | 4 (17.4) | 1 (6.6) |

^1^ After standing still for 2 minutes following at least 5 minutes supine

^2^ MAP – Mean Arterial Pressure

^3^ SAPH severe asymptomatic postural hypotension , asymptomatic decrease in MAP by ≥35 mmHg relative to baseline after 2 min standing still following ≥5 minutes supine. SSPH severe symptomatic postural hypotension, diagnosed when a subject cannot stand still for 2 minutes after ≥5 minutes supine due to drop in blood pressure.

* p < 0.05 for pairwise comparison of moxidectin treatment group vs. ivermectin treatment group.
