# Supplementary material for: A Randomized, Single-Ascending-Dose, Ivermectin-Controlled, Double-Blind Study of Moxidectin in Onchocerca volvulus Infection
Source: PLoS Negl Trop Dis. 2014 Jun 26;8(6):e2953. doi: 10.1371/journal.pntd.0002953 (PMC4072596; doi:10.1371/journal.pntd.0002953)
Supplement: Table S3 — Results of statistical analysis of the change in skin microfilaria density (mf/mg skin) from pre-treatment. (DOC) [file pntd.0002953.s003.doc]

| **Time** | **Treatment** | **N** | **Raw Mean^1^ ± SD** | **Raw Median** | **Raw Mean^1^ Change from pre-Tx** | **Raw mean^1^ % reduction from pre-Tx** | **Adj. Mean Change from pre-Tx (SE) (95% CI)^2^** | **Diff of Adj. Mean Change (95% CI)^2^ IVM - Moxidectin** | **Pairwise p value ^3^** |
| --- | --- | --- | --- | --- | --- | --- | --- | --- | --- |
| Pre-Tx | IVM | 42 | 21.22 ± 16.35 | 16.91 |  |  |  |  |  |
|  | 2 mg moxi | 42 | 23.95 ± 18.15 | 21.96 |  |  |  |  |  |
|  | 4 mg moxi | 45 | 20.61 ± 14.43 | 20.46 |  |  |  |  |  |
|  | 8 mg moxi | 37 | 22.86 ± 21.08 | 14.51 |  |  |  |  |  |
| Day 8 | IVM | 42 | 4.38 ± 9.39 | 1.13 | -16.84 | 79.37 | 0.17 (1.14) (0.14,0.23) |  |  |
|  | 2 mg moxi | 42 | 1.38 ± 1.61 | 0.82 | -22.57 | 94.23 | 0.11 (1.07) (0.10,0.13) | 0.65 (0.49,0.86) | 0.0030 |
|  | 4 mg moxi | 45 | 0.38 ± 0.39 | 0.29 | -20.24 | 98.17 | 0.08 (1.04) (0.07,0.09) | 0.46 (0.35,0.60) | <0.0001 |
|  | 8 mg moxi | 37 | 0.16 ± 0.23 | 0.05 | -22.69 | 99.29 | 0.07 (1.04) (0.06,0.08) | 0.40 (0.31,0.52) | <0.0001 |
| M 1 | IVM | 42 | 1.76 ± 6.95 | 0.00 | -19.46 | 91.71 | 0.09 (1.13) (0.07,0.12) |  |  |
|  | 2 mg moxi | 42 | 0.06 ± 0.13 | 0.00 | -23.89 | 99.76 | 0.06 (1.04) (0.06,0.07) | 0.64 (0.50,0.83) | 0.0007 |
|  | 4 mg moxi | 45 | 0.00 ± 0.02 | 0.00 | -20.61 | 99.99 | 0.06 (1.03) (0.06,0.06) | 0.65 (0.50,0.83) | 0.0007 |
|  | 8 mg moxi | 37 | 0.00 ± 0.02 | 0.00 | -22.85 | 99.99 | 0.06 (1.04) (0.06,0.07) | 0.65 (0.50,0.84) | 0.0010 |
| M 2 | IVM | 42 | 1.24 ± 4.12 | 0.00 | -19.98 | 94.16 | 0.09 (1.14) (0.07,0.12) |  |  |
|  | 2 mg moxi | 41 | 0.01 ± 0.03 | 0.00 | -22.47 | 99.97 | 0.06 (1.04) (0.05,0.06) | 0.65 (0.50,0.85) | 0.0018 |
|  | 4 mg moxi | 45 | 0.01 ± 0.03 | 0.00 | -20.61 | 99.97 | 0.06 (1.03) (0.06,0.07) | 0.68 (0.53,0.88) | 0.0038 |
|  | 8 mg moxi | 37 | 0.00 ± 0.01 | 0.00 | -22.85 | 99.99 | 0.06 (1.04) (0.06,0.07) | 0.68 (0.52,0.88) | 0.0040 |
| M 3 | IVM | 42 | 1.12 ± 4.24 | 0.00 | -20.10 | 94.71 | 0.09 (1.12) (0.07,0.11) |  |  |
|  | 2 mg moxi | 42 | 0.01 ± 0.03 | 0.00 | -23.94 | 99.97 | 0.06 (1.05) (0.05,0.06) | 0.65 (0.51,0.83) | 0.0006 |
|  | 4 mg moxi | 45 | 0.00 ±0.00 | 0.00 | -20.61 | 100.00 | 0.06 (1.03) (0.06,0.06) | 0.68 (0.54,0.87) | 0.0017 |
|  | 8 mg moxi | 37 | 0.00 ± 0.00 | 0.00 | -22.86 | 100.00 | 0.06 (1.04) (0.06,0.07) | 0.69 (0.54,0.87) | 0.0022 |
| M 6 | IVM | 42 | 1.64 ± 4.46 | 0.35 | -19.59 | 92.29 | 0.11 (1.13) (0.08,0.13) |  |  |
|  | 2 mg moxi | 42 | 0.06 ± 0.20 | 0.00 | -23.89 | 99.73 | 0.06 (1.04) (0.06,0.07) | 0.57 (0.44,0.72) | <0.0001 |
|  | 4 mg moxi | 45 | 0.03 ± 0.13 | 0.00 | -20.58 | 99.85 | 0.06 (1.03) (0.06,0.07) | 0.59 (0.46,0.74) | <0.0001 |
|  | 8 mg moxi | 37 | 0.00 ± 0.00 | 0.00 | -22.86 | 100.00 | 0.06 (1.04) (0.06,0.07) | 0.57 (0.45,0.73) | <0.0001 |
| M 12 | IVM | 42 | 3.38 ± 4.37 | 1.30 | -17.84 | 84.06 | 0.18 (1.14) (0.14,0.23) |  |  |
|  | 2 mg moxi | 42 | 0.94 ± 1.61 | 0.19 | -23.01 | 96.08 | 0.09 (1.07) (0.08,0.10) | 0.51 (0.38,0.69) | <0.0001 |
|  | 4 mg moxi | 45 | 0.81 ± 1.92 | 0.19 | -19.80 | 96.07 | 0.09 (1.07) (0.08,0.10) | 0.50 (0.37,0.67) | <0.0001 |
|  | 8 mg moxi | 37 | 0.42 ± 0.85 | 0.00 | -22.44 | 98.17 | 0.08 (1.05) (0.07,0.09) | 0.44 (0.33,0.58) | <0.0001 |
| M 18 | IVM | 42 | 4.02 ± 4.82 | 1.84 | -17.20 | 81.06 | 0.21 (1.15) (0.16,0.27) |  |  |
|  | 2 mg moxi | 42 | 2.80 ± 3.42 | 1.25 | -21.15 | 88.32 | 0.16 (1.10) (0.13,0.19) | 0.75 (0.54,1.04) | 0.0822 |
|  | 4 mg moxi | 45 | 2.16 ± 3.93 | 0.26 | -18.46 | 89.53 | 0.12 (1.12) (0.10,0.15) | 0.60 (0.42,0.84) | 0.0035 |
|  | 8 mg moxi | 37 | 1.84 ± 3.25 | 0.30 | -21.02 | 91.96 | 0.12 (1.11) (0.09,0.14) | 0.56 (0.40,0.79) | 0.0009 |

Abbreviations: Adj.=Adjusted, CI = confidence interval; IVM=ivermectin; SD = standard deviation; SE=standard error; moxi= moxidectin; M=Month, N= Number of participants, Pre-Tx =pre-treatment

^1^ Arithmetric means

^2^ Microfilarial counts were logarithmically transformed (y=log(y+1)) before analysis and the results of adjusted means anti-log transformed.  Values obtained from mixed model (SAS version 9.1 procedure Procmixed using restricted maximum likelihood estimates): change from baseline = treatment + time + treatment*time + microfilarial density at baseline + gender + treatment* microfilarial density at baseline*gender + treatment* microfilarial density at baseline + treatment*gender in initial model. Paired comparisons of ivermectin with each dose of moxidectin was conducted using a hierarchical procedure.

Overall p-value <0.0001

Tests of interaction: Treatment x Microfilarial Density at Baseline x Gender p-value <0.0001

Treatment x Microfilarial Density at Baseline p-value 0.0089

Treatment x Gender p-value 0.8730

^3^ p values for comparison of each moxidectin dose level with ivermectin
