# Supplementary material for: A Randomized, Single-Ascending-Dose, Ivermectin-Controlled, Double-Blind Study of Moxidectin in Onchocerca volvulus Infection
Source: PLoS Negl Trop Dis. 2014 Jun 26;8(6):e2953. doi: 10.1371/journal.pntd.0002953 (PMC4072596; doi:10.1371/journal.pntd.0002953)
Supplement: Table S4 — Results of statistical analysis of the percentage of participants with undetectable levels of skin microfilariae. (DOC) [file pntd.0002953.s004.doc]

| **Timepoint** | **Treatment** | **Number (%)with undetectable skin microfilariae** | **Overall p-value*** | **Pairwise p-value*** |
| --- | --- | --- | --- | --- |
| Day 8 | Ivermectin | 4/42 (9.5) | 0.0008 |  |
|  | 2 mg moxi | 6/42 (14.3) |  | 0.5843 |
|  | 4 mg moxi | 13/45 (28.9) |  | 0.0186 |
|  | 8 mg moxi | 18/37 (48.6) |  | 0.0008 |
| Month 1 | Ivermectin | 23/42 (54.8) | <0.0001 |  |
|  | 2 mg moxi | 33/42 (78.6) |  | 0.0082 |
|  | 4 mg moxi | 44/45 (97.8) |  | <0.0001 |
|  | 8 mg moxi | 36/37 (97.3) |  | <0.0001 |
| Month 2 | Ivermectin | 28/42 (66.7) | <0.0001 |  |
|  | 2 mg moxi | 39/41 (95.1) |  | 0.0003 |
|  | 4 mg moxi | 43/45 (95.6) |  | 0.0004 |
|  | 8 mg moxi | 36/37 (97.3) |  | 0.0004 |
| Month 3 | Ivermectin | 23/42 (54.8) | <0.0001 |  |
|  | 2 mg moxi | 40/42 (95.2) |  | <0.0001 |
|  | 4 mg moxi | 45/45 (100) |  | <0.0001 |
|  | 8 mg moxi | 37/37 (100) |  | <0.0001 |
| Month 6 | Ivermectin | 13/42 (31.0) | <0.0001 |  |
|  | 2 mg moxi | 34/42 (81.0) |  | <0.0001 |
|  | 4 mg moxi | 41/45 (91.1) |  | <0.0001 |
|  | 8 mg moxi | 37/37 (100) |  | <0.0001 |
| Month 12 | Ivermectin | 8/42 (19.0) | <0.0001 |  |
|  | 2 mg moxi | 15/42 (35.7) |  | 0.0165 |
|  | 4 mg moxi | 18/45 (40.0) |  | 0.0030 |
|  | 8 mg moxi | 22/37 (59.5) |  | <0.0001 |
| Month 18 | Ivermectin | 6/42 (14.3) | 0.0980 |  |
|  | 2 mg moxi | 7/42 (16.7) |  | 0.4817 |
|  | 4 mg moxi | 12/45 (26.7) |  | 0.1487 |
|  | 8 mg moxi | 13/37 (35.1) |  | 0.0473 |

Abbreviations: moxi= moxidectin;

*P-value calculated using Cochran Mantel Hostel on baseline skin microfilarial density category and gender of subjects. Paired comparisons of ivermectin with each dose of moxidectin was conducted using a hierarchical procedure.
