# Supplementary material for: A Randomized, Single-Ascending-Dose, Ivermectin-Controlled, Double-Blind Study of Moxidectin in Onchocerca volvulus Infection
Source: PLoS Negl Trop Dis. 2014 Jun 26;8(6):e2953. doi: 10.1371/journal.pntd.0002953 (PMC4072596; doi:10.1371/journal.pntd.0002953)
Supplement: Table S5 — Results of histological assessment of excised onchocercal palpable nodules. (DOC) [file pntd.0002953.s005.doc]

A) Ivermectin treated participants

| **Onchocercal Nod/participant*** | **1** | **2** | **3** | **5** | **8** | **Any (1-8)** |
| --- | --- | --- | --- | --- | --- | --- |
| **Participants** | **14** | **5** | **3** | **1** | **1** | **24** |
| **Onchocercal Nod** | **14** | **10** | **9** | **5** | **8** | **46** |
|  |  |  |  |  |  |  |
| **FM live (% of total FM)** | **16 (61.5)** | **14 (60.9)** | **8 (50.0)** | **4 (40.0)** | **4 (23.5)** | **46 (50.0)** |
| Average±SD** | 1.1±1.2 | 2.8±1.6 | 2.7±2.5 | NA | NA | 1.9±1.7 |
| Range** | 0–4 | 1–5 | 0–5 | NA | NA | 0-5 |
| **Young FM (% of total FM)** | **9 (34.6)** | **4 (17.4)** | **6 (37.5)** | **4 (40.0)** | **4 (23.5)** | **27 (29.3)** |
| Average±SD | 0.6±0.9 | 0.8±1.1 | 2.0±2.0 | NA | NA | 1.1±1.4 |
| Range | 0-3 | 0-2 | 0-4 | NA | NA | 0-4 |
| **FM dead (% of total FM)** | **7 (26.9)** | **6 (26.1)** | **4 (25.0)** | **4 (40.0)** | **7 (41.2)** | **28 (30.4)** |
| Average±SD | 0.5±0.5 | 1.2±1.3 | 1.3±1.5 | NA | NA | 1.2±1.7 |
| Range | 0-1 | 0-3 | 0-3 | NA | NA | 0-3 |
| **FM moribound (% of total FM)** | **0** | **0** | **0** | **0** | **0** | **0** |
| **FM calcified (% of total FM)** | **3 (11.5)** | **3 (13.0)** | **4 (25.0)** | **2 (20.0)** | **6 (35.3)** | **18 (19.6)** |
| Average±SD | 0.2±0.4 | 0.6±0.9 | 1.3±0.5 | NA | NA | 0.8±1.4 |
| Range | 0-1 | 0-2 | 0-3 | NA | NA | 0-6 |
|  |  |  |  |  |  |  |
| **MM live (% of total MM)** | **10 (100)** | **5 (100)** | **4 (100)** | **1 (100)** | **3 (100)** | **23 (100)** |
| Average±SD | 0.7±0.6 | 1.0±0.7 | 1.3±0.6 | NA | NA | 1.0±0.8 |
| Range | 0-2 | 0-2 | 1-2 | NA | NA | 0-3 |
| **MM dead (% of total MM)** | **0** | **0** | **0** | **0** | **0** | **0** |
| **MM moribound (% of total MM)** | **0** | **0** | **0** | **0** | **0** | **0** |
| **MM calcified (% of total MM)** | **0** | **0** | **0** | **0** | **0** | **0** |
|  |  |  |  |  |  |  |
| **FM prod E (% of live FM)** | **8 (50.0)** | **5 (35.7)** | **4 (50.0)** | **1 (25.0)** | **1 (25.0)** | **19 (41.3)** |
| Average±SD | 0.6±0.6 | 1.0±0.7 | 1.3±1.5 | NA | NA | 0.8±0.8 |
| Range | 0-2 | 0-2 | 0-3 | 1 | 1 | 0-3 |
| **FM deg E (% of FM prod E)** | **2 (25.0)** | **3 (60.0)** | **1 (25.0)** | **0** | **1 (100)** | **7 (36.8)** |
| Average±SD | 0.2±0.4 | 0.6±0.9 | 0.3±0.6 | NA | NA | 0.3±0.6 |
| Range | 0-1 | 0-2 | 0-1 | NA | NA | 0-2 |
| **FM w rel/deg E** | **0** | **0** | **0** | **0** | **0** | **0** |
| **FM w sperm (% of live FM)** | **6 (37.5)** | **5 (35.7)** | **0** | **1 (25.0)** | **0** | **12 (26.1)** |
| Average±SD | 0.4±0.7 | 1.0±0.7 | NA | NA | NA | 0.5±0.7 |
| Range | 0-2 | 0-2 | NA | NA | NA | 0-2 |
|  |  |  |  |  |  |  |
| **MM normal spermatogenesis (% of live MM)** | **10 (100)** | **5 (100)** | **4 (100)** | **1 (100)** | **3 (100)** | **23 (100)** |
| Average±SD | 0.7±0.6 | 1.0±0.7 | 1.3±0.6 | NA | NA | 0.96±0.75 |
| Range | 0-2 | 0-2 | 1-2 | NA | NA | 0-3 |
|  |  |  |  |  |  |  |
| **Nod with mf (% of Nod)** | **5 (35.7)** | **4 (40.0)** | **2 (22.2)** | **0** | **0** | **11 (23.9)** |
| Average±SD | 0.4±0.5 | 0.8±0.5 | 0.7±1.2 | NA | NA | 0.5±0.6 |
| Range | 0-1 | 0-1 | 0-2 | NA | NA | 0-2 |
| **Nod without MM (% of Nod)** | **5 (35.7)** | **5 (50.0)** | **5 (55.6)** | **4 (80.0)** | **5 (62.5)** | **24 (52.2)** |
| Average±SD | 0.4±0.5 | 1.0±0.7 | 1.7±0.6 | NA | NA | 1.0±1.3 |
| Range | 0-1 | 0-2 | 1-2 | NA | NA | 0-5 |

B) 2 mg moxidectin treated participants

| **Onchocercal Nod/participant** | **1** | **2** | **3** | **4** | **5** | **8** | **Any (1-8)** |
| --- | --- | --- | --- | --- | --- | --- | --- |
| **Participants** | **16** | **6** | **7** | **1** | **1** | **1** | **32** |
| **Onchocercal Nod** | **16** | **12** | **21** | **4** | **5** | **8** | **66** |
|  |  |  |  |  |  |  |  |
| **FM live (% of total FM)** | **19 (63.3)** | **7 (38.9)** | **21 (63.6)** | **7 (77.8)** | **6 (75.0)** | **13 (76.5)** | **73 (63.5)** |
| Average±SD | 1.2±1.3 | 1.2±1.5 | 3.0±0.8 | NA | NA | NA | 2.3±2.7 |
| Range | 0-5 | 0-4 | 2-4 | NA | NA | NA | 0-13 |
| **Young FM (% of total FM)** | **13 (43.3)** | **5 (27.8)** | **14 (42.4)** | **6 (66.7)** | **4 (50.0)** | **6 (35.3)** | **48 (41.7)** |
| Average±SD | 0.8±0.9 | 0.8±1.2 | 2.0±1.0 | NA | NA | NA | 1.5±1.6 |
| Range | 0-3 | 0-3 | 1-3 | NA | NA | NA | 0-6 |
| **FM dead (% of total FM)** | **7 (23.3)** | **7 (38.9)** | **8 (24.2)** | **1 (11.1)** | **1 (12.5)** | **3 (17.6)** | **27 (23.5)** |
| Average±SD | 0.4±0.8 | 1.2±0.8 | 1.1±0.4 | NA | NA | NA | 0.4±0.8 |
| Range | 0-3 | 0-2 | 1-2 | NA | NA | NA | 0-3 |
| **FM moribound (% of total FM)** | **0** | **0** | **0** | **0** | **0** | **0** | **0** |
| **FM calcified (% of total FM)** | **4 (13.3)** | **4 (22.2)** | **4 (12.1)** | **1 (11.1)** | **1 (12.5)** | **1 (5.9)** | **15 (13.0)** |
| Average±SD | 0.3±0.4 | 0.7±0.8 | 0.6±0.5 | NA | NA | NA | 0.5±0.6 |
| Range | 0-1 | 0-2 | 0-1 | NA | NA | NA | 0-2 |
|  |  |  |  |  |  |  |  |
| **MM live (% of total MM)** | **13 (100)** | **4 (80.0)** | **10 (100)** | **2 (100)** | **2 (100)** | **4 (80.0)** | **35 (94.6)** |
| Average±SD | 0.8±0.7 | 0.7±0.8 | 1.4-1.0 | NA | NA | NA | 1.1±1.0 |
| Range | 0-2 | 0-2 | 0-3 | NA | NA | NA | 0-4 |
| **MM dead (% of total MM)** | **0** | **1 (20.0)** | **0** | **0** | **0** | **1 (20.0)** | **2 (5.4)** |
| **MM moribound (% of total MM)** | **0** | **0** | **0** | **0** | **0** | **0** | **0** |
| **MM calcified (% of total MM)** | **0** | **0** | **0** | **0** | **0** | **0** | **0** |
|  |  |  |  |  |  |  |  |
| **FM prod E (% of live FM)** | **5 (26.3)** | **1 (14.3)** | **7 (33.3)** | **0** | **1 (16.7)** | **4 (30.8)** | **18 (24.7)** |
| Average±SD | 0.3±0.5 | 0.2±0.4 | 1.0±0.6 | NA | NA | NA | 0.6±0.8 |
| Range | 0-1 | 0-1 | 0-2 | NA | NA | NA | 0-4 |
| **FM deg E (% of FM prod E)** | **0** | **0** | **0** | **0** | **1 (100)** | **0** | **1 (5.6)** |
| **FM rel/deg E** | **0** | **0** | **0** | **0** | **0** | **0** | **0** |
| **FM w sperm (% of live FM)** | **6 (31.6)** | **0** | **7 (33.3)** | **4 (57.1)** | **0** | **5 (38.5)** | **22 (30.1)** |
| Average±SD | 0.4±0.5 |  | 1.0±1.2 | NA | NA | NA | 0.7±1.2 |
| Range | 0-1 |  | 0-3 | NA | NA | NA | 0-5 |
|  |  |  |  |  |  |  |  |
| **MM normal sp-genesis (% of live MM)** | **13 (100)** | **4 (100)** | **10 (100)** | **2 (100)** | **2 (100)** | **4 (100)** | **35 (100)** |
| Average±SD | 0.8±0.7 | 0.7±0.8 | 1.4±1.0 | NA | NA | NA | 1.1±1.0 |
| Range | 0-2 | 0-2 | 0-3 | NA | NA | NA | 0-4 |
|  |  |  |  |  |  |  |  |
| **Nod with mf (% of Nod)** | **2 (12.5)** | **1 (8.3)** | **5 (23.8)** | **0** | **0** | **2 (25.0)** | **10 (15.2)** |
| Average±SD | 0.1±0.3 | 0.2±0.4 | 0.7±0.5 | NA | NA | NA | 0.3±0.5 |
| Range | 0-1 | 0-1 | 0-1 | NA | NA | NA | 0-2 |
| **Nod without MM (% of Nod)** | **5 (31.3)** | **7 (58.3)** | **12 (57.1)** | **2 (50.0)** | **3 (60.0)** | **4 (50.0)** | **33 (50.0)** |
| Average±SD | 0.3±0.5 | 1.2±0.8 | 1.7±0.8 | NA | NA | NA | 1.0±1.1 |
| Range | 0-1 | 0-2 | 1-3 | NA | NA | NA | 0-4 |

C) 4 mg moxidectin treated participants

| **Onchocercal Nod/participant** | **1** | **2** | **3** | **4** | **5** | **Any (1-5)** |
| --- | --- | --- | --- | --- | --- | --- |
| **Participants** | **22** | **8** | **2** | **2** | **1** | **35** |
| **Onchocercal Nod** | **22** | **16** | **6** | **8** | **5** | **57** |
| **FM live (% of total FM)** | **19 (52.8)** | **15 (71.4)** | **9 (90.0)** | **12 (100)** | **6 (54.5)** | **61 (67.8)** |
| Average±SD | 0.9±0.6 | 1.9±0.6 | NA | NA | NA | 1.7±1.7 |
| Range | 0-2 | 1-3 | 3-6 | 5-7 | NA | 0-7 |
| **Young FM (% of total FM)** | **8 (22.2)** | **7 (33.3)** | **9 (90.0)** | **8 (66.7)** | **4 (36.4)** | **36 (40.0)** |
| Average±SD | 0.4±0.5 | 0.9±1.0 | NA | NA | NA | 1.0±1.5 |
| Range | 0-1 | 0-3 | 3-6 | 3-5 | NA | 0-6 |
| **FM dead (% of total FM)** | **10 (27.8)** | **4 (19.0)** | **1 (10.0)** | **0** | **3 (27.3)** | **18 (20.0)** |
| Average±SD | 0.5±0.6 | 0.5±0.5 | NA | NA | NA | 0.5±0.7 |
| Range | 0-2 | 0-1 | 0-1 | NA | NA | 0-3 |
| **FM moribound (% of total FM)** | **0** | **0** | **0** | **0** | **0** | **0** |
| **FM calcified (% of total FM)** | **7 (19.4)** | **2 (9.5)** | **0** | **0** | **2 (18.2)** | **11 (12.2)** |
| Average±SD | 0.3±0.6 | 0.3±0.5 | NA | NA | NA | 0.3±0.6 |
| Range | 0.2 | 0.1 | NA | NA | NA | 0-2 |
|  |  |  |  |  |  |  |
| **MM live (% of total MM)** | **16 (100)** | **8 (100)** | **6 (100)** | **5 (100)** | **4 (100)** | **39 (100)** |
| Average±SD | 0.7±0.6 | 1.0±0.5 | NA | NA | NA | 1.1±1.0 |
| Range | 0-2 | 0-2 | 2-4 | 2-3 | NA | 0-4 |
| **MM moribound (% of total MM)** | **0** | **0** | **0** | **0** | **0** | **0** |
| **MM dead (% of total MM)** | **0** | **0** | **0** | **0** | **0** | **0** |
|  |  |  |  |  |  |  |
| **FM prod E (% of live FM)** | **9 (47.4)** | **4 (26.7)** | **7 (77.8)** | **7 (58.3)** | **5 (83.3)** | **32 (52.5)** |
| Average±SD | 0.4±0.6 | 0.5±0.5 | NA | NA | NA | 0.9±1.3 |
| Range | 0-2 | 0-1 | 3-4 | 3-4 | NA | 0-5 |
| **FM deg E (% of FM prod E)** | **1 (11.1)** | **2 (50.0)** | **2 (28.6)** | **4 (57.1)** | **3 (60.0)** | **12 (37.5)** |
| Average±SD | 0.05±0.2 | 0.3±0.5 | NA | NA | NA | 0.3±0.8 |
| Range | 0-1 | 0-1 | 0-2 | 2 | 3 | 0-3 |
| **FM rel/Deg E (% of FM not prod E)** | **0** | **0** | **0** | **1 (20)** | **0** | **1 (3.4)** |
| **FM w sperm (% of live FM)** | **9 (47.4)** | **4 (26.7)** | **3 (33.3)** | **1 (8.3)** | **2 (33.3)** | **19 (31.1)** |
| Average±SD | 0.4±0.5 | 0.5±0.5 | NA | NA | NA | 0.5±0.6 |
| Range | 0-1 | 0-1 | 1-2 | 0-1 | NA | 0-2 |
|  |  |  |  |  |  |  |
| **MM normal sp-genesis (% of live MM)** | **16 (100)** | **8 (100)** | **6 (100)** | **4 (80)** | **4 (100)** | **38 (97.4)** |
| Average±SD | 0.7±0.6 | 1.0±0.5 | NA | NA | NA | 1.1±1.0 |
| Range | 0-2 | 0-2 | 2-4 | 1-3 | NA | 0-4 |
|  |  |  |  |  |  |  |
| **Nod with mf (% of Nod)** | **6 (27.3)** | **5 (31.3)** | **3 (50.0)** | **3 (37.5)** | **2 (40.0)** | **19 (33.3)** |
| Average±SD | 0.3±0.5 | 0.6±0.7 | NA | NA | NA | 0.5±0.7 |
| Range | 0-1 | 0-2 | 1-2 | 1-2 | NA | 0-2 |
| **Nod without MM (% of Nod)** | **7 (31.8)** | **8 (50.0)** | **2 (33.3)** | **3 (37.5)** | **1 (20.0)** | **21 (36.8)** |
| Average±SD | 0.3±0.5 | 1.0±0.5 | NA | NA | NA | 0.6±0.6 |
| Range | 0-1 | 0-2 | 1 | 1-2 | NA | 0-2 |

D) 8 mg moxidectin treated participants

| **Onchocercal Nod/participant** | **1** | **2** | **3** | **4** | **5** | **Any (1-5)** |
| --- | --- | --- | --- | --- | --- | --- |
| **Participants** | **12** | **3** | **3** | **2** | **2** | **22** |
| **Onchocercal Nod** | **12** | **6** | **9** | **8** | **10** | **45** |
|  |  |  |  |  |  |  |
| **FM live (% of total FM)** | **15 (68.2)** | **6 (100)** | **7 (53.8)** | **8 (72.7)** | **11 (84.6)** | **47 (72.3)** |
| Average±SD | 1.3±0.8 | 2.0±0.0 | 2.3±1.2 | NA | NA | 2.1±1.6 |
| Range | 0-3 | 2 | 1-3 | 2-6 | 5-6 | 0-6 |
| **Young FM (% of total FM)** | **9 (40.9)** | **3 (50.0)** | **3 (23.1)** | **7 (63.6)** | **7 (53.8)** | **29 (44.6)** |
| Average±SD | 0.8±1.0 | 1.0±0.0 | 1.0±0.0 | NA | NA | 1.3±1.5 |
| Range | 0-3 | 1 | 1 | 1-6 | 3-4 | 0-6 |
| **FM dead (% of total FM)** | **5 (22.7)** | **0** | **4 (30.8)** | **3 (27.3)** | **1 (7.7)** | **13 (20.0)** |
| Average±SD | 0.4±0.7 | NA | 1.3±0.6 | NA | NA | 0.6±0.7 |
| Range | 0-2 | NA | 1-2 | 1-2 | 0-1 | 0-2 |
| **FM moribound (% of total FM)** | **0** | **0** | **0** | **0** | **0** | **0** |
| **FM calcified (% of total FM)** | **2 (9.1)** | **0** | **2 (15.4)** | **0** | **1 (7.7)** | **5 (7.7)** |
| Average±SD | NA | NA | NA | NA | NA | 0.2±0.5 |
| Range | 0-1 | NA | 0-2 | NA | NA | 0-2 |
|  |  |  |  |  |  |  |
| **MM live (% of total MM)** | **6 (100)** | **3 (100)** | **4 (100)** | **2 (40.0)** | **4 (80.0)** | **19 (82.6)** |
| Average±SD | 0.5±0.5 | 1.0±1.0 | 1.3±1.5 | NA | NA | 0.9±0.9 |
| Range | 0-1 | 0-2 | 0-3 | 0-2 | 2-2 | 0-3 |
| **MM dead (% of total MM)** | **0** | **0** | **0** | **3 (60.0)** | **1 (20.0)** | **4 (17.4)** |
| Range | NA | NA | NA | 0-3 | 0-1 | 0-3 |
| **MM moribound (% of total MM)** | **0** | **0** | **0** | **0** | **0** | **0** |
| **MM calcified (% of total MM)** | **0** | **0** | **0** | **0** | **0** | **0** |
|  |  |  |  |  |  |  |
| **FM prod E (% of live FM)** | **6 (40.0)** | **2 (33.3)** | **3 (42.9)** | **2 (25.0)** | **5 (45.5)** | **18 (38.3)** |
| Average±SD | 0.5±0.7 | 0.7±0.6 | 1.0±1.7 | NA | NA | 0.8±1.0 |
| Range | 0-2 | 0-1 | 0-3 | 1-1 | 2-3 | 0-3 |
| **FM deg E (% of FM prod E)** | **3 (50.0)** | **1 (50.0)** | **2 (66.7)** | **1 (50.0)** | **2 (40.0)** | **9 (50.0)** |
| Average±SD | 0.3±0.5 | 0.3±0.6 | 0.7±0.2 | NA | NA | 0.4±0.7 |
| Range | 0-1 | 0-1 | 0-2 | 0-1 | 0-2 | 0-2 |
| **FM rel/Deg E (% of FM not prod E)** | **1 (11.1)** | **0** | **0** | **0** | **0** | **1 (3.4)** |
| **FM w sperm (% of live FM)** | **1 (6.7)** | **0** | **2 (28.6)** | **0** | **3 (27.3)** | **6 (12.8)** |
| Average±SD | 0.1±0.3 | NA | 0.7±1.2 | NA | NA | 0.3±0.6 |
| Range | 0-1 | NA | 0-2 | NA | 1-2 | 0-2 |
|  |  |  |  |  |  |  |
| **MM normal sp-genesis (% of live MM)** | **6 (100)** | **3 (100)** | **4 (100)** | **2 (100)** | **4 (100)** | **19 (100)** |
| Average±SD | 0.5±0.5 | 1.0±1.0 | 1.3±1.5 | NA | NA | 0.9±0.9 |
| Range | 0-1 | 0-2 | 0-3 | 0-2 | 2-2 | 0-3 |
|  |  |  |  |  |  |  |
| **Nod with mf (% of Nod)** | **3 (25.7)** | **1 (16.7)** | **0** | **0** | **2 (20.0)** | **6 (13.3)** |
| Average±SD | 0.3±0.5 | 0.3±0.6 | NA | NA | NA | 0.3±0.5 |
| Range | 0-1 | 0-1 | NA | NA | 1-1 | 0-1 |
| **Nod without MM (% of Nod)** | **6 (50.0)** | **3 (50.0)** | **5 (55.6)** | **3 (37.5)** | **5 (50.0)** | **22 (48.9)** |
| Average±SD | 0.5±0.5 | 1.0±1.0 | 1.7±1.5 | NA | NA | 1.0±1.0 |
| Range | 0-1 | 0-2 | 0-3 | 1-2 | 2-3 | 0-3 |

* Unless otherwise specified in the first column, total numbers are presented

**All Average±SD (arithmetric mean ± standard deviation) and range calculated per subject, not per nodule.

Abbreviations: E – embryos, FM – female macrofilariae, mf – microfilariae, MM – male macrofilariae, Nod – nodule, sp-genesis – spermatogenesis, w - with
