# Supplementary material for: A Randomized, Single-Ascending-Dose, Ivermectin-Controlled, Double-Blind Study of Moxidectin in Onchocerca volvulus Infection
Source: PLoS Negl Trop Dis. 2014 Jun 26;8(6):e2953. doi: 10.1371/journal.pntd.0002953 (PMC4072596; doi:10.1371/journal.pntd.0002953)
Supplement: Protocol S1 — Study protocol: Amendment 4 under which the study was initiated and Amendment 5 in track changes from Amendment 4. Relative to Amendment 4, Amendment 5: • Specified that women already receiving parenterally administered contraceptive were excluded from the protocol requirement for protocol defined parenterally administered contraceptive (DMPA injections) • Allowed Informed Consent (to be obtained in the villages prior to admission, i.e., before transport to the study center) to be obtained earlier than within the time frame specified for screening procedures. • Clarified that the NCI CTC criteria were applicable only for AEs not included in the OCRC Common Toxicity Criteria (see Table S1) and attached more of these criteria to the protocol • Specified that back-up samples for pharmacokinetic analyses could be shipped to the PK laboratory as soon as confirmation had been obtained that the ‘front samples’ had been received and provided for the appropriate procedures, and adjusted the instructions for timing of shipments to allow arrangements with the carrier. • Provided for information previously to be documented by the unblinded pharmacist on paper to be reduced and documented on specifically provided labels. (PDF) [file pntd.0002953.s007.pdf]

A Randomized, Single-Ascending-Dose, Ivermectin-Controlled, Double-Blind, Safety, Tolerability, Pharmacokinetic, and Efficacy Study of Orally Administered Moxidectin in Subjects with *Onchocerca volvulus* Infection

Onchocerciasis Chemotherapy Research Centre protocol number: 33

Wyeth Research protocol number: 3110A1-200-GH

Amendment 4

Date of Original Protocol: 18 November 2003

Date of Protocol Amendment 1: 21 April 2004

Date of Protocol Amendment 2: 14 July 2004

Date of Protocol Amendment 3: 27 October 2005

Date of Protocol Amendment 4: 10 May 2006

Wyeth Medical Monitor:		Gilbert Rose, MD
	Country:	United States
	Phone (Business Hours):	+1(484) 865-5516
	Phone (After Hours):	+1(610) 692-3787
	Fax:	+1(484) 865-0071
Wyeth Medical Monitor:		Robert Maroko, MD
	Country:	United States
	Phone (Business Hours):	+1(484) 865-8566
	Phone (After Hours):	+1(610) 202-0223
	Fax:	+1(484) 865-9262
WHO Project Manager:		Janis Lazdins-Helds, MD, PhD
	Country:	Switzerland

	Phone (Business Hours):	+41 (22) 791-3818
	Phone (After Hours, Mobile)	+41 (79) 509-0669
	Fax:	+41 (22) 791-4774
WHO Clinical Coordinator		Juntra Karbwang Laothavorn, MD, PhD
	Country:	Switzerland
	Phone (Business Hours):	+41 (22) 791-3867
	Phone (After Hours, Mobile):	+41 (79) 629-2163
	Fax:	+41 (22) 791-4854
Primary Investigator		Kwablah Awadzi, MB FRCP DCMT
	Country:	Ghana
	Phone (Business Hours):	+233 (0) 935 22 132<br>+871 762 858 781 (Satellite)
	Phone (Mobile):	+233 (0) 20 201 7114
	Fax:	+233 (0) 935 22111<br>+871 762 858 783 (Satellite)

Sponsors: Wyeth Research Division of Wyeth Pharmaceuticals Inc.
Clinical Research and Development
P.O. Box 8299
Philadelphia, Pennsylvania 19101

Product Development and Evaluation Unit
TDR
World Health Organization
20 Avenue Appia
CH-1211 Geneva 27
Switzerland

TABLE OF CONTENTS

1. CONTACTS 7
  1.1 EMERGENCY CONTACTS 7
    1.1.1 WYETH 7
    1.1.2 NATIONAL CENTER FOR PHARMACOVIGILANCE 8
    1.1.3 WORLD HEALTH ORGANIZATION 8
    1.1.4 PRIMARY INVESTIGATOR 8
    1.1.5 TRIAL CENTER IN GHANA (ALL SCREENING TO BE CONDUCTED AT THIS LOCATION) 9
  1.2 ADDITIONAL CONTACTS 9
2. MEDICAL MONITOR SIGNATURE 10
3. INVESTIGATOR SIGNATURE 11
4. INDEPENDENT ETHICS COMMITTEE (IEC) 12
5. DISCLOSURE STATEMENT 13
6. LIST OF ABBREVIATIONS 14
7. DEFINITION OF TERMS 16
8. SYNOPSIS 18
9. SUBJECT FLOWCHART 25
10. INTRODUCTION 27
11. TRIAL DESIGN 36
  11.1 DESCRIPTION 36
12. STUDY OBJECTIVES 39
  12.1 PRIMARY 39
  12.2 SECONDARY 40
13. SELECTION OF STUDY POPULATION 40
  13.1 INCLUSION CRITERIA 44
  13.2 EXCLUSION CRITERIA 44
  13.3 NUMBER OF SUBJECTS 45
  13.4 SCREEN FAILURES 45
14. PRIOR TREATMENT 46
15. CONCOMITANT TREATMENT 46
  15.1 ADDITIONAL REQUIRED DRUGS 46
  15.2 PERMITTED TREATMENT 46
  15.3 PROHIBITED TREATMENT 47
16. TREATMENT OF SUBJECTS 47
  16.1 PROCEDURES 47
    16.1.1 DAYS -4 TO -2 (VISIT 1) 47
    16.1.2 DAY -1 (VISIT 2) 48
    16.1.3 DAY 1 (VISIT 3) 48
    16.1.4 DAY 2 (VISIT 4) 48
    16.1.5 DAY 3 (VISIT 5) 49

16.1.6 DAY 4 (VISIT 6)....49
16.1.7 DAY 5 (VISIT 7)....50
16.1.8 DAY 6 (VISIT 8)....50
16.1.9 DAY 7 (VISIT 9)....50
16.1.10 DAY 8 (VISIT 10)....50
16.1.11 DAY 9 (VISIT 11)....51
16.1.12 DAY 10 (VISIT 12)....51
16.1.13 DAY 11 (VISIT 13)....51
16.1.14 DAY 12 (VISIT 14)....52
16.1.15 DAY 13 (VISIT 15)....52
16.1.16 DAY 14 (VISIT 16)....52
16.1.17 DAY 15 (VISIT 17)....52
16.1.18 DAY 16 (VISIT 18)....53
16.1.19 DAY 17 (VISIT 19)....53
16.1.20 DAY 18 (VISIT 20)....53
16.1.21 1-MONTH (VISIT 21)....53
16.1.22 2-MONTHS (VISIT 22)....54
16.1.23 3-MONTHS (VISIT 23)....54
16.1.24 6-MONTHS (VISIT 24)....55
16.1.25 12-MONTHS (VISIT 25)....56
16.1.26 18-MONTHS (VISIT 26)....56
16.2 DISCONTINUATION AND WITHDRAWAL OF SUBJECTS....57
16.3 SPECIAL METHODS....58
16.3.1 PHYSICAL EXAMINATIONS AND MEDICAL HISTORY....58
16.3.2 VITAL SIGNS....58
16.3.3 ELECTROCARDIOGRAMS....59
16.3.4 OCULAR EXAMINATION....59
16.3.5 LABORATORY EVALUATIONS/SPECIMEN COLLECTION....59
16.3.6 SKIN SNIPS....60
17. TEST ARTICLE....60
17.1 TEST ARTICLE(S) AND ADMINISTRATION....60
17.2 PACKAGING AND LABELING....61
17.3 STORAGE AND STABILITY....62
17.4 PREPARATION....63
17.5 TEST ARTICLE ACCOUNTABILITY, RECONCILIATION, AND RETURN....63
17.6 STUDY SUBJECT COMPLIANCE....64
17.7 OTHER SUPPLIES....64
18. MEASURES TO MINIMIZE/AVOID BIAS....65
18.1 SUBJECT IDENTIFICATION....65
18.2 RANDOMIZATION....66
18.3 BLINDING AND UNBLINDING....67
19. SAFETY....68
19.1 SAFETY VARIABLES....68
19.2 SAFETY ASSESSMENT METHODS AND DOSE PROGRESSION DECISION....70
19.3 SAFETY LABORATORY DETERMINATIONS....71

20. EFFICACY 71
20.1 EFFICACY VARIABLES 71
20.1.1 PRIMARY 71
20.1.2 SECONDARY 72
20.2 EFFICACY ASSESSMENT METHODS 73
21. PHARMACOKINETIC ANALYSIS 73
22. STATISTICAL ANALYSIS 74
22.1 STATISTICAL METHODS 74
22.2 METHODOLOGY 74
22.3 SAMPLE SIZE AND POWER 75
23. CONTRAINDICATIONS, PRECAUTIONS, AND WARNINGS 76
24. ADVERSE EVENTS 77
24.1 DEFINITIONS 77
24.2 TIMING FOR REPORTING SERIOUS ADVERSE EVENTS 80
24.3 REPORTABLE EVENTS/INFORMATION 81
24.4 RECORDING AND REPORTING 82
25. DATA QUALITY ASSURANCE 83
26. INVESTIGATORS REGULATORY OBLIGATIONS 84
26.1 INDEPENDENT ETHICS COMMITTEE (GFDB/IEC) APPROVAL 84
26.2 PRESTUDY DOCUMENTATION 84
26.3 INFORMED CONSENT 85
26.4 DECLARATION OF HELSINKI 86
26.5 CASE REPORT FORMS 86
26.6 ADVERSE EVENT REPORTING 86
26.7 REVIEW OF SOURCE RECORDS 86
26.8 MONITORING OF THE STUDY 87
26.9 PROTOCOL AMENDMENTS 87
26.10 CHANGE IN INVESTIGATOR 88
26.11 TERMINATION OF THE STUDY 88
26.11.1 TERMINATION BY THE SPONSORS 88
26.11.2 TERMINATION BY THE INVESTIGATOR 88
26.12 FINAL STUDY REPORT 88
26.13 CONFIDENTIALITY 89
26.14 RECORDS RETENTION 89
26.15 PUBLICATIONS 89
26.16 SUBJECT INJURY 90

27. SPONSOR OBLIGATIONS IN CASE OF PREMATURE TRIAL TERMINATION 90
28. REFERENCES 91
ATTACHMENTS 93
ATTACHMENT 1. RESPONSIBILITIES OF THE UNBLINDED THIRD PARTY PHARMACIST 94
ATTACHMENT 2. COLLECTION, PREPARATION, AND LABELING OF PLASMA SAMPLES 95
ATTACHMENT 3. INSTRUCTIONS FOR SHIPPING FROZEN SAMPLES 96
ATTACHMENT 4. INSTRUCTIONS FOR PREPARATION OF STUDY DRUG 98
ATTACHMENT 5. RECOMMENDATIONS FOR GRADING OF OCULAR EXAMINATIONS 104
ATTACHMENT 6. POTENTIAL MAZZOTTI LABORATORY MANIFESTATIONS 107

1. CONTACTS

1.1 Emergency Contacts

1.1.1 Wyeth

Contact 1 of the persons indicated below for serious adverse events and other emergencies:

Wyeth Medical Monitor

Name:	Gilbert Rose, MD
Country:	United States
Phone (Business Hours):	+1 (484) 865-5516
Phone (After Hours):	+1 (610) 692-3787
Fax:	+1 (484) 865-0071

Wyeth Medical Monitor

Name:	Robert Maroko, MD
Country:	United States
Phone (Business Hours):	+1(484) 865-8566
Phone (After Hours):	+1(610) 202-0223
Fax:	+1(484) 865-0071

Wyeth Clinical Project Team Leader

Name:	Angel Cooper
Country:	United States
Phone (During business hours):	+1 (484) 865-4238
Phone (After business hours):	+1 (215) 527-1808
Fax:	+1 (484) 865-0071

Clinical Scientist

Name:	Russ Orrico
Country:	United States
Phone (During business hours):	+1 (484) 865-5587
Phone (After business hours):	+1 (610) 653-6104
Fax:	+1 (484) 865-0071

Emergency Telephone Number: If the persons named above cannot be reached, call +1 (610)-902-1200 and indicate that you have a product-related emergency.

1.1.2 National Center for Pharmacovigilance

Name:	E. K. Agyarko
Country:	Ghana
Phone (1):	+233 (0) 21 661248
Phone (2):	+233 (0) 21 673090
Phone (3)	+233 (0) 21 779838
Phone (4)	+233 (0) 21 778196
Fax:	+233 (0) 21 660389

1.1.3 World Health Organization

WHO Clinical Coordinator

Name:	Juntra Karbwang Laothavorn, MD, PhD
Country:	Switzerland
Phone (Business Hours):	+41 (22) 791-3867
Phone (After Hours, Mobile):	+41 (79) 629-2163
Fax:	+41 (22) 791-4854

WHO Project Manager

Name:	Janis Lazdins-Helds, MD, PhD
Country:	Switzerland
Phone (During business hours):	+41 (22) 791-3818
Phone (After Hours, Mobile):	+41 (79) 509-0669
Fax:	+41 (22) 791-4774

1.1.4 Primary Investigator

Primary Investigator	Kwablah Awadzi, MB FRCP DCMT
Country:	Ghana
Phone (Business Hours):	+ 871 762 858781 (Satellite)
	+233 (0) 935 22 132
Phone (Mobile):	+233 (0) 20 201 7114

Fax:	+233 (0) 935 22111
	+871 762 858 783 (Satellite)

1.1.5 Trial Center in Ghana (all screening to be conducted at this location)

Onchocerciasis Chemotherapy Research Centre
Government Hospital
PO Box 144
Hohoe, Volta Region
Ghana

1.2 Additional Contacts

Contact the following for all other inquiries and information about this study:

Clinical Scientist

Name:	Russ Orrico
Country:	United States
Phone (During business hours):	+1 (484) 865-5587
Phone (After business hours):	+1 (610) 653-6104
Fax:	+1 (484) 865-0071

2. MEDICAL MONITOR SIGNATURE

I have read and approve this protocol. I assure that this study will be conducted according to all stipulations of the protocol, including all statements regarding confidentiality.

Wyeth Medical Monitor's Signature	Date of Signature (DDMmmYYYY)	Time (24-hour clock, time zone)
Wyeth Medical Monitor's Name (print)		
WHO Clinical Coordinator's Signature	Date of Signature (DDMmmYYYY)	Time (24-hour clock, time zone)
Juntra Karbwang, M.D, PhD<br>WHO Clinical Coordinator's Name (print)		

.

Time Zone Abbreviations:
Atlantic Time Eastern Time – AT ET, Central European Time – CET, Central Time – CT, East Australia Time – EAT, Greenwich Mean Time – GMT, Japan Time – JT, Mountain Time – MT, Pacific Time – PT)

3. INVESTIGATOR SIGNATURE

I have read this protocol and agree that it contains all necessary details for carrying out the study as described. I will conduct this protocol as outlined therein, including all statements regarding confidentiality. I will make a reasonable effort to complete the study within the time designated. I will provide copies of the protocol and access to all information furnished by the sponsors to study personnel under my supervision. I will discuss this material with them to ensure that they are fully informed about the drug and the study. I understand that the study may be terminated or enrollment suspended at any time by the sponsors, with or without cause, or by me if it becomes necessary to protect the best interests of the study subjects.

I agree to conduct this study in full accordance with all applicable regulations and Good Clinical Practice (GCP).

Investigator's Signature	Date of Signature (DDMmmYYYY)	Time (24-hour clock, time zone)

Kwablah Awadzi, MB FRCP DCMT

Investigator's Name (print)

4. INDEPENDENT ETHICS COMMITTEE (IEC)

The independent ethics committee (IEC) and its affiliation and address for *each site* implementing this protocol shall be identified below.

IEC:	Ghana IEC Ethical Review Committee (ERC)
Affiliation:	Ghana Health Service
Address:	P.O. Box GP-184
	Accra, Ghana

IEC:	WHO IEC (Research Ethics Review Committee (ERC))
Affiliation:	WHO
Address:	20 Avenue Appia
	CH 1211 Geneva 27
	Switzerland

5. DISCLOSURE STATEMENT

Restricted Distribution of Documents

This document contains information that is confidential and proprietary to the sponsors. This information is being provided to you solely for the purpose of evaluating and/or conducting a clinical trial for the sponsors. You may disclose the contents of this document only to study personnel under your supervision, IECs, or duly authorized representatives of regulatory agencies for this purpose under the condition that they maintain confidentiality. The contents of this document may not be used in any other clinical trial, disclosed to any other person or entity, and/or published without the prior written permission of the sponsors. The foregoing shall not apply to disclosure required by any regulations; however, you will give prompt notice to the sponsors of any such disclosure.

Any information that may be added to this document also is confidential and proprietary to the sponsors and must be kept in confidence in the same manner as the contents of this document.

The Wyeth Research protocol number for this study is 3110A1-200-GH.

The Onchocerciasis Chemotherapy Research Centre protocol number is 33.

6. LIST OF ABBREVIATIONS

Abbreviation	Term
AE	Adverse event
ALT	Alanine aminotransferase (SGPT)
AP	Alkaline phosphatase
AST	Aspartate aminotransferase (SGOT)
ß-hCG	ß-human Chorionic Gonadotropin
BUN	Blood urea nitrogen
CBC	Complete blood count
CERT	Clinical Expert Review Team
CNS	Central nervous system
CRF	Case report form
CV	Curriculum vitae
DMPA	Depo-medroxyprogesterone acetate
EC	Ethics committee
ECG	Electrocardiogram
EMEA	European Medicines Evaluations Agency
ERC	Ethical Review Committee
FDA	United States Food and Drug Administration
G6PD	Glucose-6-phosphate dehydrogenase
GABA	Gamma aminobutyric acid
GCP	Good clinical practice
GGT	Gamma glutamyl transferase
GFDB	Ghana Food and Drugs Board
GHS	Ghana Health Services
GNCP	Ghana National Center for Pharmacovigilance
HPMC	Hypromellose (Hydroxypropyl Methylcellulose)
ICH	International Conferences on Harmonization
IV	Intravenous
IEC	Independent ethics committee
LDH	Lactate dehydrogenase
Mf	Microfilariae
mITT	Modified Intent-to-Treat
NOEL	No observed effect level
NTEL	No toxic effect level
NCI	National Cancer Institute
OCP	Onchocerciasis Control Programme
OCRC	Onchocerciasis Chemotherapy Research Center
OTC	Over the counter
PD	Pharmacodynamic
PRD	Product Research and Development Unit of TDR
PK	Pharmacokinetic
PI	Principal investigator
PRT	Project Review Team
SAE	Serious adverse event
$t^{1/2}$	Terminal elimination half-life
TEAE	Treatment Emergent Adverse Event

Abbreviation	Term
TIC	Tablets in capsules
TDR	UNICEF/United Nations Development Program/Worldbank/WHO Special Programme for Research and Training in Tropical Diseases
ULN	Upper limit of normal
URTI	Upper Respiratory Tract Infection
WBC	White blood cell or White Blood Cell Count
WHO	World Health Organization
WNL	Within normal limits

7. DEFINITION OF TERMS

Term	Definition
Regulation	Throughout this document the term *regulation* refers to all appropriate regulations, laws, and guidelines. This study will be conducted according to all appropriate regulations. The regulations may be international, national, or local and may include but not be limited to the Code of Federal Regulations (United States); the Good Clinical Practice: Consolidated Guideline (Canada); the International Conference on Harmonisation Guideline for Good Clinical Practice; the Therapeutic Goods Administration Annotated International Conference on Harmonisation Guidelines (Australia); the Standard Operating Procedures for Clinical Investigators (UNDP/World Bank/WHO Special Programme for Research and Training in Tropical Diseases); the World Medical Association Declaration of Helsinki: Ethical Principles for Medical Research Involving Human Subjects (See Declaration of Helsinki section).
Regulatory Agency	Throughout this document the term *regulatory agency* refers to all appropriate health and regulatory agencies. These may be international, national, or local and may include but not be limited to the Australian Therapeutic Goods Administration (TGA), the Canadian Health Products and Food Branch (HPFB), the European Agency for the Evaluation of Medicinal Products (EMEA), the US Food and Drug Administration (FDA), and the Ghana Food and Drugs Board (GFDB).
Sponsors	Throughout this document the term *sponsors* refers to all appropriate research departments. These may include but not be limited to Wyeth Research Division of Wyeth Pharmaceuticals Inc. and Product Research and Development Unit (TDR), World Health Organization.
Study Start Date	Date the first subject is admitted to the hospital for the study.
Subject	Any person who participates in a study, either as the recipient of a test article or as a control subject. A subject may be healthy or have a disease.
Test Article	Any drug, device, biological agent (including placebo) required by the

Term	Definition
	protocol and supplied by the sponsors for use in the sponsor's clinical research and development studies.

8. SYNOPSIS

**Study Title**	A Randomized, Single-Ascending-Dose, Ivermectin-Controlled, Double-Blind, Safety, Tolerability, Pharmacokinetic, and Efficacy Study of Orally Administered Moxidectin in Subjects with *Onchocerca volvulus* infection.
**Clinical Phase**	Phase 2
**Study Rationale**	Moxidectin, an anthelmintic, is currently licensed and marketed as an antiparasitic for use in cattle, sheep, swine, horses, and dogs. Preclinical experiments have shown that orally administered moxidectin may be useful treatment for onchocerciasis (river blindness) in humans. Moxidectin kills the microfilariae and may kill adult worms as well. Ivermectin, a related drug that is currently the standard treatment for river blindness, kills only the microfilariae. The first-in-man (FIM) study (protocol 3110A1-100-EU) investigating the effect of single doses of 3 mg to 36 mg of moxidectin in healthy subjects has been completed. In this study, 37 subjects were treated with single doses of 3 mg to 36 mg (approximately 50 µg/kg to 600 µg/kg). Subjects were randomized into placebo, 3 mg fasting, 9 mg fasting, 9 mg fed, 18 mg fasting, 36 mg fed and 36 mg fasting groups. Only 1 adverse event (AE) higher than grade 2 was reported in this study (enteritis due to food poisoning [grade 3] in the 36 mg fasting group) and was considered unrelated to study drug. The most common AEs were headache (35%) and infection (29%). Only half of the headaches reported and none of the infections reported were regarded as study drug related. The reported infections, including type of infection, time of occurrence after moxidectin administration, and dose group were: upper respiratory tract infection (URTI) on Day 19 and Day 63 (same subject), and a viral URTI on Day 14 in the 3 mg fasted group; toe infection on Day 52 in the 9 mg fasted group – URTI on Day 30 and Day 78 (different subjects) in the 9 mg fed group; tooth abscess on Day 47 in the 36 mg fasted group; head cold on Day 50 and Day 66 (different subjects) in the 36 mg fasted group; cold on Day 42 and cold-like symptoms on Day 59 (same subject) in the 36 mg fed group. No serious adverse events (SAEs) were reported. Four (4) subjects discontinued from the study (1 before receiving study drug and 3 after dose administration [one each in the 3 mg fasting, 9 mg fed, and 36 mg fasting groups]). The withdrawal from the study was unrelated to AEs. Overall, moxidectin appeared to be safe at the doses evaluated in this study.<br>In a bioavailability study comparing the tablet and liquid forms of

	moxidectin (protocol 3110A1-101-EU) 58 healthy male subjects received a single dose of 10 mg of moxidectin either as the liquid formulation (29 subjects) used in the FIM study or as tablets (29 subjects) and were followed up for 180 days. During the course of this study, there were no SAEs and no subjects discontinued the study due to an AE. A total of 36 (62.1%) subjects had treatment emergent AEs (TEAEs) during the study, with the same number and percentage of subjects (18; 62.1%) reporting TEAEs in both the moxidectin liquid and tablet groups. During the first 7 days post treatment, 10 subjects (34.5%) receiving liquid moxidectin and 9 subjects (31.0%) receiving tablet reported AEs, including asthenia (10.3% in the tablet group), headache (13.8% and 6.9% in the liquid and tablet groups, respectively), infection (6.9% in the liquid group), diarrhea (6.9% in the liquid group), myalgia (6.9% in the tablet group), and dizziness (6.9% in the tablet group). Between day 8 and 180 of follow up, 10 subjects (34.5%) receiving liquid and 12 subjects (41.4%) receiving tablet reported AEs. The most commonly reported events were flu syndrome (17.2% and 20.7% in liquid and tablet groups, respectively), headache (6.9% in the tablet group), and infection (6.9% in the tablet group). All of the TEAEs were mild to moderate in intensity, and none were considered to be related to treatment. No clinically relevant abnormalities were observed in vital sign measurements, ECGs, or laboratory tests during the study.<br>The data from the normal subject studies (protocol 3110A1-100-EU, 3110A1-101-EU) suggests that progression to a safety and tolerability study in subjects with different degrees of severity of infection with *Onchocerca volvulus* as described in this protocol is warranted.<br>This will be the first study of moxidectin in subjects with *O. volvulus* infection and will examine the safety, tolerability, pharmacokinetics, and efficacy of oral moxidectin in subjects to determine safe and effective doses of moxidectin and provide proof of concept. The lowest dose of moxidectin to be used in this study (2 mg) is lower than the lowest dose used in the FIM study (3 mg). As noted above, the 3 mg dose was well tolerated in the FIM study.
**Trial Design**	This is a randomized, single-ascending dose, double blind, active drug-controlled, parallel design, inpatient/outpatient, study at a single center. See the flowchart(s) for the procedures to be performed at each visit.
**Approximate Duration of Subject Participation**	Each subject will participate for approximately 18 months including an initial 18-day inpatient period.

**Approximate Duration of Study**	The total duration of the study will be up to 36 months.
**Study Objective(s)**	**Primary:** To determine the safety and tolerability of orally administered moxidectin in subjects with *O. volvulus* infection, as measured by the incidence of clinical adverse events and clinically significant laboratory test results.<br>**Secondary:**<br>1. To determine doses that effectively eliminate microfilariae and prevent their return to the skin as measured by the skin microfilarial loads at day 8, 1 month, 2 months, 3 months, 6 months, 12 months and 18 months after treatment.<br>2. To determine the viability and fertility of adult worms at 18 months.<br>3. To assess the pharmacokinetics of moxidectin in male and female adult subjects.
**Diagnosis and Main Criteria for Inclusion**	Men and women in good general health, with *O. volvulus* infection and:<br>a) Written, signed (or thumb-printed), and dated informed consent<br>b) Aged 18 to 60 years, inclusive<br>c) Body weight ≥ 40 kg for women and ≥ 45 kg for men<br>d) Nonpregnant, nonbreastfeeding women. Women of child bearing potential must agree to use birth control during the first 150 days after treatment.<br>e) Healthy, as determined by a physician on the basis of a physical examination, ECG, and a thorough review of the medical history and clinical laboratory results<br>f) Adequate hematologic, renal, and hepatic function, defined as:<br>1) White blood cell (WBC) count ≥ 2,800 and ≤ 11,300 cells/mL<br>2) Hemoglobin: ≥ 11.0 g/dL for men and ≥ 10.0 g/dL for women<br>3) Platelet count: ≥ 110,000 mm^3<br>4) Serum creatinine: ≤ 1.25 x upper limit of normal (ULN)<br>5) Total bilirubin: ≤ 1.25 x ULN<br>6) AST/SGOT: ≤ 1.25 x ULN<br>7) AP: ≤ 1.25 x ULN<br>8) Prothrombin time WNL<br>9) Urinalysis WNL<br>g) Skin microfilarial density within the required range for the cohort
**Main Criteria for Exclusion**	a) Participation in any studies other than purely observational ones, within 4 weeks before test article administration.<br>b) Any vaccination within 4 weeks before test article administration<br>c) Acute infection requiring therapy within the last 10 days before test article administration

	d) Administration of any medication (with the exception of medication required to treat any reactions during the screening fluorescein angiography (chlorpheniramine) or paracetamol) or herbal preparation within 10 days prior to test article administration or any condition currently requiring regular medication<br>e) Clinically significant ECG abnormalities or history of cardiac abnormality<br>f) Past or current history of neurological or neuropsychiatric disease or epilepsy<br>g) Subjects with orthostatic hypotension at the screening evaluation<br>h) History of drug or alcohol abuse or regular use of ≥ 3 cigarettes per day<br>i) Use of alcohol or other drugs of abuse within 72 hours before test article administration<br>j) Any condition, in the investigator's opinion, that places the subject at undue risk<br>k) Subjects who have donated blood within 8 weeks before study entry<br>l) Subjects with ocular onchocerciasis in cohorts intended to enroll subjects with mild infection. Ocular onchocerciasis is defined by the presence of live or dead microfilariae, onchocercal punctate opacities, onchocercal lesions of the posterior segment or lesions that mimic those seen in onchocerciasis.<br>m) Subjects with hyperreactive onchodermatitis<br>n) Antifilarial therapy within the previous 5 years<br>o) Coincidental infection with Loa Loa<br>p) Female subjects of childbearing potential with a contraindication to DMPA if not on Norplant<br>q) Any other condition which the investigator feels would exclude the subject from the study
**Approximate Number of Subjects**	A total of 192 subjects will be enrolled in 9 consecutive cohorts:<br>2 mg: 16 subjects with > 0 and < 10 microfilariae/mg of skin<br>2 mg: 16 subjects with 10-20 microfilariae/mg of skin and the sum of microfilariae in the two eyes must be ≤ 10<br>2 mg: 32 subjects with > 20 microfilariae/mg of skin with or without ocular involvement (to be enrolled in 2 consecutive groups of 16 subjects)<br><br>4 mg: 16 subjects with > 0 and < 10 microfilariae/mg of skin<br>4 mg: 16 subjects with 10-20 microfilariae/mg of skin and the

	sum of microfilariae in the two eyes must be ≤ 10<br>4 mg: 32 subjects with > 20 microfilariae/mg of skin with or without ocular involvement (to be enrolled in 2 consecutive groups of 16 subjects)<br><br>8 mg: 16 subjects with > 0 and < 10 microfilariae/mg of skin<br>8 mg: 16 subjects with 10-20 microfilariae/mg of skin and the sum of microfilariae in the two eyes must be ≤ 10<br>8 mg: 32 subjects with > 20 microfilariae/mg of skin with or without ocular involvement (to be enrolled in 2 consecutive groups of 16 subjects)
**Approximate Number of Study Centers**	1
**Concomitant Treatment**	Depo-medroxyprogesterone acetate (DMPA), 150 mg, will be given twice to all women of childbearing potential. The first dose will be given on study day 11 and the second dose will be at month 2 (±1 week). Women who have contraceptive protection by the Norplant system will be excluded from the DMPA injections.<br>Chlorpheniramine may be administered to reduce adverse reactions to fluorescein sodium and paracetamol may be administered for painful conditions. All medication given after admission to the hospital will be documented in the CRF. Treatment with ivermectin, albendazole, or other anthelmintic agents will be prohibited for the duration of the study.
**Test Article(s) and Administration**	Moxidectin, ivermectin and placebo capsules are identical in appearance. Moxidectin tablets in capsules (TIC) will be supplied as 2 mg tablets encapsulated in hydroxypropyl methylcellulose (HPMC) capsules. The capsules are filled with a mixture of inert ingredients including lactose, microcrystalline cellulose, sodium starch glycolate and magnesium stearate. One (1) moxidectin TIC is required for a 2 mg dose; 2 TICs are required for a 4 mg dose; 4 TICs are required for an 8 mg dose.<br>Ivermectin TIC will be supplied as 3 mg tablets encapsulated in HPMC capsules filled with the same inert ingredients as the moxidectin capsules. Ivermectin will be dosed according to the weight-based dosing schedule approved for the treatment of onchocerciasis in the ivermectin package insert ranging from 6-12 mg. Placebo will be supplied as HPMC capsules filled with the same inert ingredients as the moxidectin and ivermectin capsules. Each subject will receive a single dose of 4 capsules, including moxidectin, moxidectin plus placebo, ivermectin or ivermectin plus placebo depending on body weight and treatment assignment.

**Safety Evaluation**	Vital signs, ECGs, physical examinations (including a neurologic examination), ocular examinations, AEs, serum chemistries, hematology studies, prothrombin times, and urinalyses.
**Efficacy Evaluation**	Efficacy evaluations (skin snips) will be performed at day 8, 1 month (± 1 week), 2 months (± 1 week), 3 months (± 1 week), 6 months (± 1 month), 12 months (± 1 month) and 18 months (± 1 month) after dosing and the number of microfilariae/mg of skin determined. Evaluation of the viability and fertility of the adult worms will occur at 18 months. All located nodules will be excised at 18 months, processed for histopathology, and slides interpreted by one or more observers. Microfilarial migration into blood and urine will be used as indirect evidence of the speed of microfilaricidal activity.<br>All subjects who have skin microfilariae at the 18-month visit will be treated with the approved dosage of ivermectin.
**Pharmacokinetics**	Blood samples for determination of moxidectin plasma concentrations will be collected within 2 hours prior to dosing (0 hr), and at 1, 2, 4, 8, 24, and 72 hours after study drug administration and on study days 8, 13, 18, 1 month (± 1 week), 2 months (± 1 week), 3 months (± 1 week), 6 months (± 1 month), and 12 months (± 1 month). Pharmacokinetic (PK) samples will be drawn from all subjects in all cohorts with the exception of the 2nd group of 16 subjects with >20 microfilariae/mg of skin in cohorts 3, 6, and 9. Samples from subjects will be analyzed by a validated bioanalytical method.<br>A PK analysis will be performed using model independent methods to characterize the PK parameters of moxidectin, such as maximum plasma concentration (C_{max}), time to reach the maximum plasma concentration (t_{max}), area under the plasma concentration vs. time curve (AUC) and the apparent terminal elimination half-life ($t_{1/2}$).
**Pharmacodynamics**	Analysis of a correlation between PK parameters, gender, severity of infection, efficacy, and efficacy-related AEs.
**Statistical Analysis**	This is a randomized, double blind, active-control, dose-escalation trial comparing 3 doses of moxidectin (2, 4, and 8 mg) with ivermectin, stratified by microfilariae density (mild, moderate, severe) and gender. Subjects will be treated as inpatients for the first 18 days and as outpatients for the remainder of the 18-month study.<br>The primary analysis population for safety will be the modified intent-to-treat (mITT) population, which consists of all subjects who receive study medication. For efficacy, the primary analysis population will be the

	evaluable subject population, which is defined as all subjects who receive study medication and have baseline and 18 month microfilariae count data. An mITT sensitivity analysis will also be performed to evaluate the effect on the efficacy results of dropouts occurring before 18 months.<br>The primary objective of the study is to evaluate the safety of escalating moxidectin regimens. Both the incidence of clinical AEs and clinically significant laboratory test results will be used to assess safety. The primary efficacy endpoint is the reduction from baseline in skin microfilariae at 18 months. Microfilariae count will be defined as the geometric mean of skin microfilariae/mg taken at 4 sites (iliac crests and calves).<br>In addition, important secondary endpoints are the reduction from baseline in skin microfilariae at 8 days, 1 month, 2 months, 3 months), 6 months, and at 12 months, area under the curve (calculated by using the trapezoidal method through the 18-month visit), ocular examination (time of maximum reduction of microfilariae and number (%) of subjects with complete clearance) and nodulectomy (viability and fertility of macrofilariae) at 18 months.
**Rationale for Number of Subjects**	At each dose level, 16 mildly, 16 moderately and 32 severely infected subjects will be treated with moxidectin or ivermectin in a 3:1 ratio. The probability of detecting at least 1 AE of grade 2 or higher among 12 moxidectin-treated subjects will be 0.114 and 0.718 when the true rates are 1% and 10%, respectively. The probability of detecting at least 1 AE of grade 2 or higher among 24 moxidectin-treated subjects will be 0.214 and 0.92 when the true rates are 1% and 10%, respectively.

9. SUBJECT FLOWCHART

	Visit Number																									
**Clinical Planned Events**	1	2	3	4	5	6	7	8	9	10	11	12	13	14	15	16	17	18	19	20	21	22	23	24	25	26
	Study Day																									
**Study Procedures**	**-4 to -2**	**-1**	**1**	**2**	**3**	**4**	**5**	**6**	**7**	**8**	**9**	**10**	**11**	**12**	**13**	**14**	**15**	**16**	**17**	**18**	**1 mo ±1 wk**	**2 mo ±1 wk**	**3 mo ±1 wk**	**6 mo ± 1 mo**	**12 mo ±1 mo**	**18 mo ±1 mo**
**Informed consent**	X[a]																									
**Demographics**	X																									
**Admission**	X																									
**Medical history**	X																									
**Medication history**	X																									
**Physical examination**	X																				X	X	X	X	X	X
**Height**	X																									
**Weight**	X		X	X	X	X	X	X	X	X	X	X	X	X	X	X	X	X	X		X	X	X	X	X	X
**Vital signs**	X	X	X	X	X	X	X	X	X	X	X	X	X	X	X	X	X	X	X	X	X	X	X	X	X	X
**ECG**	X		X[b]	X	X					X																
**Ocular examination**	X				X				X							X					X	X	X’	X	X	X
**Retinal Colour Photographs and Fluorescein angiogram**[c]	X				X				X							X					X	X	X	X	X	X
**Interim physical examination**			X	X	X	X	X	X	X	X	X	X	X	X	X	X	X	X	X							
**Hematology**[d]	X		X	X		X				X					X					X	X	X	X	X	X	X
**Serum chemistry**	X		X	X		X				X					X					X	X	X	X	X	X	X
**Pregnancy test**		X																								
**Urinalysis**[3]	X		X	X		X				X					X					X	X	X	X	X	X	X
**Clinical Planned Events**	1	2	3	4	5	6	7	8	9	10	11	12	13	14	15	16	17	18	19	20	21	22	23	24	25	26

a. Will be obtained in the village prior to admission.
b. ECG to be done approximately 4 hours after study drug administration. Can be repeated on days 4 through 7 if clinically indicated.
c. For all subjects until month 3, then only in subjects with lesions or visual defects.
d. All blood and urine samples will be filtered and stained for microfilariae.

SUBJECT FLOWCHART (Continued)

	--- Visit Number ---																									
**Clinical Planned Events**	**1**	**2**	**3**	**4**	**5**	**6**	**7**	**8**	**9**	**10**	**11**	**12**	**13**	**14**	**15**	**16**	**17**	**18**	**19**	**20**	**21**	**22**	**23**	**24**	**25**	**26**
	--- Study Day ---																									
**Study Procedures**	**-4 to -2**	**-1**	**1**	**2**	**3**	**4**	**5**	**6**	**7**	**8**	**9**	**10**	**11**	**12**	**13**	**14**	**15**	**16**	**17**	**18**	**1 mo ±1 wk**	**2 mo ±1 wk**	**3 mo ±1 wk**	**6 mo ± 1 mo**	**12 mo ±1 mo**	**18 mo ±1 mo**
**Skin snips**	X									X											X	X	X	X	X	X
**Randomization**		X																								
**Drug packaging and dispensing**		X																								
**Double-blind drug administration**			X[e]																							
**Moxidectin Plasma Samples**		X[f]	X[g]	X		X				X					X					X	X	X	X	X	X	
**Adverse events**	X	X	X	X	X	X	X	X	X	X	X	X	X	X	X	X	X	X	X	X	X	X	X	X	X	X
**DMPA[h]**													X									X				
**Discharge**																				X						
**Nodulectomy**																										X[i]
**Clinical Planned Events**	1	2	3	4	5	6	7	8	9	10	11	12	13	14	15	16	17	18	19	20	21	22	23	24	25	26

e. Will be given after an overnight fast and 2 hours before breakfast.
f. Day -1 sample will be taken approximately 2 hours before drug administration.
g. Day 1 samples will be taken at 1, 2, 4, and 8 hours after drug administration.
h. Depo-medroxyprogesterone acetate (DMPA) on women of childbearing potential not protected by Norplant system.
i. All located nodules will be processed for histopathology and slides read by 1 or more blinded observers.

10. INTRODUCTION

Moxidectin is a macrocyclic lactone drug that is derived from the actinomycete *Streptomyces cyanogriseus* (eg, milbemycin). The compound was developed by Fort Dodge Animal Health as a veterinary product, and is registered worldwide for the prevention of canine heartworm (ProHeart®) and for the treatment of internal and external parasites in cattle, sheep, and horses (Cydectin®).

The World Health Organization (WHO) is interested in using moxidectin for the control of human onchocerciasis. Onchocerciasis, often referred to as river blindness, is a parasitic disease caused by the filarial worm *Onchocerca volvulus* and is transmitted between humans through the bite of black flies of the genus *Simulium*. Onchocerciasis is still endemic in 34 countries: 27 in WHO's African region, 6 in the region of the Americas, and 1 in the Eastern Mediterranean region. Each adult female worm, which can live for an average of 11 years but up to 18 years in the human body, produces millions of microfilariae that migrate throughout the body. The manifestations of onchocerciasis are caused by the microfilariae. Onchocerciasis primarily affects the skin, eyes, and lymph nodes. Severe itching and rash are the most frequent manifestations of the disease. Visual impairment, including blindness, is the most serious consequence. Both manifestations have serious social and economic consequences that justify the vigorous efforts being made to control the disease. The current estimate is that approximately 18 million people are infected, of whom 6.5 million suffer from severe itching or dermatitis, 270,000 are blind, and a further 500,000 are severely visually disabled.

The ultimate goal of the WHO's African Programme for Onchocerciasis Control is to eliminate onchocerciasis as a disease of public health and socioeconomic importance. Currently, the strategies to control the disease include killing the larvae of the black fly vector with insecticides and the treatment of populations at risk with the macrocyclic lactone drug ivermectin (Mectizan®). Ivermectin is a microfilaricide that must be administered at least once per year to control the disease by reducing the numbers of microfilariae in the skin and eyes. Because this treatment has to be given for the life span of adult worms and does not protect subjects from reinfection, it is widely recognized that an alternate treatment is needed, preferably a macrofilaricide that can effect a cure. In addition to its microfilaricidal properties, ivermectin has been shown to have some activity against other stages of *Onchocerca volvulus*: it has a partial in-vivo effect against third-stage, but not fourth-stage, larvae in experimentally infected

chimpanzees[1]; after multiple dosage, it reduces insemination and impairs oogenesis in adult female worms[2]; and causes some decrease in adult worm numbers and viability.[3]

Moxidectin has some known mechanistic differences from ivermectin. It is considered significantly more potent than ivermectin and may also disrupt additional stages within the parasitic life cycle. Although structurally related to ivermectin, moxidectin is more lipophilic and is therefore expected to have a longer duration of action in humans than ivermectin after oral administration.

Pharmacology

The exact mechanism of action of moxidectin is still under investigation. Studies indicate that moxidectin binds to glutamate-gated chloride channels in the neurons and muscle cells of parasites.[4] Binding to the ion channel results in hyperpolarization of the nerve and muscle fibers leading to paralysis and death of the parasitic organism. Specificity of moxidectin for the parasite versus the mammalian host results from this compound having a low affinity for the mammalian chloride channels. An additional action of moxidectin is its activity at the GABA-A (gamma aminobutyric acid-A) receptor complex.[5] The multidrug transporter P-glycoprotein has been implicated in the mechanism of resistance to macrocyclic lactones.[6] However, moxidectin has been shown to control parasites that are resistant to other macrocyclic lactones as well as benzimidazoles[7,8] suggesting a different mechanism or susceptibility to resistance. A different binding activity has been demonstrated for a GABA-gated chloride ion channel between ivermectin and moxidectin.[9]

Various in vitro and in vivo studies in mice, jirds, dogs, and cattle have evaluated the activity of moxidectin against *Onchocerca* species and *Brugia pahangi*. In in vitro test systems, moxidectin, ivermectin, doramectin, and milbemycin oxime were evaluated against microfilariae from *Onchocerca volvulus* and *Onchocerca lienalis* in 7-day motility assays. Worm motility was significantly reduced by all 4 compounds at 2 hours, followed by a steady decline up to 120 hours. At the end of the study, the mean percentage reductions in worm motility for ivermectin, moxidectin, doramectin, and milbemycin oxime were 86%, 93%, 93%, and 74%, respectively.[10,11]

Studies in CBA/Ca mice experimentally infected with *Onchocerca lienalis* and *Onchocerca volvulus* consistently showed that subcutaneous or oral moxidectin was more efficacious than ivermectin in clearing microfilariae.[10, 11]

Investigation of Guadali cattle in Cameroon addressed the effect of moxidectin and ivermectin on preventing infection of *Onchocerca ochengi* L3 to L4 stage larvae in naïve calves under very high natural challenge (infection) conditions. Ivermectin (150 μg/kg) or moxidectin (200 μg/kg) was subcutaneously administered monthly or at 3-month intervals. At the end of the study, 11 of 14 control animals had 110 nodules. Animals treated with ivermectin every month had no nodules, but 2 of 10 animals treated with ivermectin every 3 months had 2 nodules. In moxidectin-treated animals, no nodules were found in animals treated monthly or once every 3 months.[12]

The activity of moxidectin against *Brugia pahangi* was evaluated in a jird and a dog model. In the jird model, the effect of moxidectin, ivermectin, and doramectin on microfilariae was determined. Moxidectin induced a rapid reduction in microfilariae, with none detectable after day 224. Ivermectin and doramectin treatment resulted in a gradual reduction in microfilariae through day 336; however, most jirds still had few microfilariae remaining. The effect on macrofilariae (adult worms) was also assessed. Treatment of jirds with moxidectin resulted in >90% killing of adult worms, whereas ivermectin or doramectin did not have any effect on adult worm burden.[13] In the dog model, moxidectin at doses of 250 μg/kg or 1000 μg/kg was given 1, 3, 6, or 12 times over a 12-month period. After the first dose of moxidectin, the microfilariae count for all treated dogs gradually declined from pretreatment levels during the first 2 weeks after treatment. By 3 months, the microfilariae counts were suppressed 90% or more from pretreatment levels. The microfilariae counts remained suppressed to the end of the study at 12 months, with 3 of the 78 moxidectin-treated dogs not clearing microfilariae.[14] In the same study, the effect of moxidectin on the macrofilariae was evaluated. At 6 months after the first dose, dogs treated with moxidectin at 250 μg/kg for 3 or 6 doses had 77% and 92% fewer worms, respectively, than controls. At 12 months after the first dose, dogs given 1 or more doses of moxidectin at either 250 μg/kg or 1000 μg/kg had at least 92% fewer worms than controls, with only 6 of 48 treated dogs having live adult worms at necropsy. The worm recoveries from all of the moxidectin treatments were significantly less ($p<0.05$) than the recoveries for the controls, but there was no significant difference ($p> 0.05$) among the worm recoveries for the different moxidectin treatments. In conclusion, moxidectin was a potent microfilaricidal agent. The suppression of microfilariae is independent of the dosage or the number of treatments and, in the dog model, the effect is sustained for at least 1 year. Furthermore, moxidectin was also effective against *B. pahangi* adults.[14]

Animal data indicate that moxidectin is highly lipophilic, residing primarily in fatty tissue. The compound has a long half-life, approximately 20 days in dogs, sheep, and horses. Based on initial PK

data for the 3 mg to 36 mg dose groups in the single-ascending-dose FIM study (protocol 3110A1-100-EU) conducted in volunteer subjects, mean half-life ($t_{1/2}$) in humans ranges from approximately 19.9 to 37.4 days.

Toxicology

Safety studies have been completed in mice, rats, dogs, sheep, cattle, horses, and humans. Repeated-dose toxicity was evaluated in oral (diet) studies in mice (4 weeks), rats (4 and 13 weeks), and dogs (4 and 13 weeks and 1 year), and in oral (diet) carcinogenicity studies in mice and rats (2 years). Results of the repeated-dose studies indicate that moxidectin was well tolerated with no toxicologic effects at calculated dosages of up to 6.9 mg/kg/day in mice for 4 weeks, 3.9 mg/kg/day in rats for 13 weeks, or 1.1 mg/kg/day in dogs for 1 year. At toxic dose levels, events such as decreased activity, prostration, tremors, chromodacryorrhea, decreased respiration, diarrhea, hypersensitivity to touch and sound, and epistaxis were observed. However, the no–toxicologic-effect levels (NTELs) in all animal models were high multiples of the anticipated human dosage on the basis of body weight. The anticipated maximum human dose is a single 8 mg dose per year (0.16 mg/kg for a 50 kg human). The lowest NTEL in single dose studies in the most sensitive species (mice) was 78 mg/kg, the NTEL in repeated dose studies were 6.9 mg/kg/day in mice for 4 weeks, 3.9 mg/kg/day in rats for 13 weeks, and 0.8 mg/kg/day and 1.1 mg/kg/day in dogs for 4 week and 1 year, respectively.

Reproductive Toxicology

Moxidectin was administered by oral gavage at dosages of 0, 2.5, 5, 10, and 12 mg/kg/day once daily to presumed pregnant rats from gestation day 6 through 15. Moxidectin was maternally toxic at dosages of ≥ 10 mg/kg/day as evidenced by decreased body weight, body-weight gain, and food consumption during the daily dosing period. Although not statistically significant, fetal body weights at ≥ 5 mg/kg/day tended to be decreased (≤ 6%) in comparison with controls. At ≥ 10 mg/kg/day, there were statistically significant increases in the number of fetuses with malformations and/or variations (3.1%, 4.3%, 5.5%, 7.9% and 8.4% at 0, 2.5, 5, 10, and 12 mg/kg/day, respectively). The numbers of litters with malformations and/or variations also increased (26.1%, 41.7%, 40.0%, 54.2% and 54.2% at 0, 2.5, 5, 10, and 12 mg/kg/day, respectively). These increases largely reflected increases in cleft palate and/or reversible delays in ossification that were not considered related to reduced fetal weight. It is well

established in the scientific literature that maternal toxicity can result in fetal alterations including malformations and delays in ossification.

There were no moxidectin-related effects on any other maternal or embryo-fetal parameter examined. The maternal and developmental no-toxic-effect level (NTEL) was concluded to be 5 mg/kg/day, with increased incidences of cleft palate and delayed ossification in the presence of maternal toxicity at the higher dosages of 10 and 12 mg/kg/day.

Moxidectin was also administered by oral gavage at dosages of 0, 1, 5, or 10 mg/kg/day to groups of artificially inseminated rabbits from gestation day 7 through 19. Maternal toxicity was evident at dosages ≥ 5 mg/kg/day as evidenced by decreased body weight, body-weight gain, and food consumption during the dosing period. Reduced litter size (6.2 compared with 7.1 in controls), reflective of increased early resorptions (1.2 compared with 0.2 in controls) was observed at 10 mg/kg/day. There were no other moxidectin-related effects, including malformations or other fetal alterations, observed in this study. The maternal and developmental NTELs were concluded to be 1 and 5 mg/kg/day, respectively.

The highest dose of moxidectin to be studied in Phase 2 and possibly Phase 3 clinical trials is a single, oral dose of 8 mg, which is a dosage of 0.16 mg/kg or 5.4 mg/m^2 (based on body surface area) for a 50kg human. Moxidectin was well tolerated in healthy male subjects after oral administration (liquid formulation) of single doses as high as 36 mg (24.5 mg/m^2). The developmental NTEL in both the rat and rabbit studies was 5 mg/kg/day administered in 10 and 13 consecutive, daily doses in rats and rabbits, respectively. The NTEL of 5 mg/kg is approximately 32 times the highest single dose (8 mg) to be administered to humans based on body weight, and approximately 3 times (rat) or 8 times (rabbit) based on body surface area. In the female rat, moxidectin has a half-life of 45 hours (data not available for the rabbit). Therefore, daily administration of moxidectin to pregnant rats, as was done in the teratogenicity study of moxidectin, would likely result in accumulation of the drug in the body, further increasing exposure of the fetus to the drug. Based on the dose per unit of body weight, or dose based on body surface area, the intake by a woman of a single 8 mg dose of moxidectin would be unlikely to pose a risk to the developing human fetus.

Developmental risk can also be assessed based on actual exposure of the body to moxidectin, assuming placental transfer is similar in rats and humans. In a Phase 1 clinical trial using the liquid formulation of moxidectin, a single oral dose to healthy males of 18 mg of moxidectin resulted in an area under the

concentration-time curve (AUC) value of 227 ng/day/mL, which is equivalent to 9.55 ng/hr/mL. In female rats, a single oral gavage dosage of 0.2 mg/kg of moxidectin resulted in an AUC value of 470 ng/hr/mL, which is approximately 50 times the human AUC after a single dose of 18 mg of moxidectin. Assuming linear PK in rats, the developmental NTEL of 5 mg/kg would correspond to an AUC value of approximately 11,750 ng/hr/mL, which is more than 1200 times the human AUC after a single dose of 18 mg of moxidectin. Assuming similar bioavailability of the liquid and tablet formulations of moxidectin in humans (as demonstrated in protocol 3110A1-101-EU), and based on the above exposure considerations, the oral administration of up to 8 mg of moxidectin is unlikely to pose a risk to the developing human fetus.

Prior Human Experience-Potential Risks and Benefits to Human Subjects

The results of nonclinical studies supported the development of moxidectin for the control of human onchocerciasis and the initiation of study 3110A1-100: a single-ascending dose, placebo-controlled, double-blind, safety, tolerability, and pharmacokinetic study of orally administered moxidectin in normal subjects. In this study, 38 healthy subjects were treated with single doses of 3 mg to 36 mg (approximately 50 µg/kg to 600 µg/kg). Subjects were randomized into placebo, 3 mg fasting, 9 mg fasting, 9 mg fed, 18 mg fasting, 36 mg fed and 36 mg fasting groups. Rapid absorption was seen in all dose groups. The results of the comparison of time of maximum concentration (T_{max}) between fasted and fed groups suggested that high-fat food may delay the absorption of moxidectin. Only 1 adverse event (AE) higher than grade 2 was reported in this study (enteritis due to food poisoning [grade 3] in the 36 mg fasting group) and was considered unrelated to study drug. The most common treatment emergent AEs (TEAEs) were headache (35%) and infection (29%). Only half of the headaches reported and none of the infections reported were regarded as study drug related. The reported infections, including type of infection, time of occurrence after moxidectin administration, and dose group were: upper respiratory tract infection (URTI) on Day 19 and Day 63 (same subject) and viral URTI on Day 14 in the 3 mg fasted group; toe infection on Day 52 in the 9 mg fasted group; URTI on Day 30 and Day 78 (different subjects) in the 9 mg fed group; tooth abscess on Day 47 and head cold on Day 50 and Day 66 (different subjects) in the 36 mg fasted group; cold on Day 42 and cold-like symptoms on Day 59 (same subject) in the 36 mg fed group. No SAEs were reported. Four (4) subjects discontinued from the study (1 before receiving study drug and 3 after dose administration [in the 3 mg fasting, 9 mg fed, and 36 mg fasting groups]). The withdrawal from the study was unrelated to AEs. Overall, moxidectin appears to be safe at the doses evaluated in this study.

In another study (protocol 3110A1-101-EU) the relative bioavailability of a tablet (to be used in this study) and a liquid formulation (used in the first study in healthy subjects; protocol 31101A1-100-EU) of moxidectin at the 10-mg dose strength were compared. This was an open-label, randomized, single-dose, parallel-design study conducted in 58 young healthy male subjects. Subjects' mean age (32 years, range 20 – 45 years) and weight (81 kg, range 57 – 107 kg) were similar for those receiving the tablet formulation (n = 29) and those receiving the liquid formulation (n =29). Each subject received 1 orally administered dose of moxidectin, either as five 2-mg tablets, or as a liquid. In both cases, moxidectin was administered under fasting conditions. Subjects were followed for 180 days.

During the course of the study, there were no SAEs and no subjects discontinued the study due to an AE.

A total of 36 (62.1%) subjects had TEAEs during the study, with the same number and percentage of subjects (18; 62.1%) reporting TEAEs in both the moxidectin liquid and tablet groups. The most commonly reported (by ≥ 5% of subjects) TEAEs were flu syndrome (17.2% and 20.7% in the liquid and tablet groups, respectively), headache (17.2% and 13.8% in the liquid and tablet groups, respectively), infection (13.8% and 6.9% in the liquid and tablet groups, respectively), diarrhea (10.3% in the liquid group), myalgia (6.9% in the tablet group), and dizziness (6.9% in the tablet group).

A total of 19 of 58 (32.8%) subjects reported TEAEs on or prior to day 7 post treatment (during the inpatient period) including 10 of 29 (34.5%) subjects in the group receiving moxidectin liquid and 9 of 29 (31.0%) subjects in the group receiving moxidectin tablets. The most commonly reported (by ≥ 5% of subjects) TEAEs during this period were asthenia (10.3% in the tablet group), headache (13.8% and 6.9% in the liquid and tablet groups, respectively), infection (6.9% in the liquid group), diarrhea (6.9% in the liquid group), myalgia (6.9% in the tablet group), and dizziness (6.9% in the tablet group).

A total of 22 (37.9%) subjects reported TEAEs during the outpatient period of the study (after study day 7 and up to study day 180) including 10 (34.5%) subjects in the liquid group and 12 (41.4%) subjects in the tablet group. The most commonly reported events were flu syndrome (17.2% and 20.7% in liquid and tablet groups, respectively), headache (6.9% in the tablet group), and infection (6.9% in the tablet group).

All of the TEAEs that were reported during the study were mild to moderate in intensity, and none were considered to be related to treatment. No clinically relevant abnormalities were observed in vital sign measurements, ECGs or laboratory tests during the study.

The data from the normal subject studies (protocol 3110A1-100-EU and 3110A1-101 EU) suggests that progression to a safety and tolerability study in subjects with different degrees of severity of infection with *Onchocerca volvulus* as described in this protocol is warranted. Based on the preclinical pharmacology data, moxidectin may be microfilaricidal and in addition may eliminate the reproductive capacity of the macrofilaria or be macrofilaricidal. If moxidectin has either of these effects on the macrofilaria, it has the potential to cure the disease in the trial subjects who are recruited from an area without onchocerciasis control, i.e. areas without vector control or ivermectin distribution. If moxidectin does not have either this effect on macrofilaria or a microfilaricidal effect, subjects will benefit from participating in the protocol by the nodulectomies performed, which will free them of all palpable nodules and reduce the number of macrofilaria in their bodies that can generate microfilariae.

Route of Administration, Dosage and Treatment Period

In this study, a tablet formulation will be available for both ivermectin and moxidectin. A single dose regimen was chosen for this study, as well as for the whole development program, because a longer dosing regimen is not compatible with the ultimate use of moxidectin within onchocerciasis control programs.

Moxidectin dose selection (2 mg, 4 mg and 8 mg) was based on comparison of the maximum plasma levels obtained during the first study in human subjects (protocol 3110A1-100-EU, 3 mg dose: mean 22 ng/mL range 17.1-28.5 ng/mL; 9 mg: mean 54-58 ng/mL, range 38.9 – 66.5ng/mL) with those of the approved dose of ivermectin (mean 46.6 ng/mL after a 12 mg dose, equivalent to 150 µg/kg for an 80 kg adult), and on the pre-clinical pharmacology data on the relative efficacy of moxidectin and ivermectin. The frequency and severity of AEs after ivermectin treatment are related to the amount of microfilariae killed. Most of the serious and/or severe AEs after ivermectin treatment occur within the first 72 hours after dosing. To reduce the risk associated with AEs related to microfilaria killing after moxidectin treatment, three measures have been included in this protocol: (1) the initial dose of moxidectin in subjects was chosen to be only 2 mg, (2) subjects are enrolled at each dose level sequentially by increasing severity of infection and (3) subjects are hospitalized during the first 18 days post treatment (see section 11).

Animal veterinary experience

Moxidectin Canine SR Injectable (ProHeart® 6, PH6) is a novel sustained release injectable product, consisting of 10% moxidectin in microspheres containing glyceryltristerate, developed by Fort Dodge Animal Health (FDAH) for the prevention of heartworm. PH6 has been marketed in the United States since June 2001, and is also approved for use in Canada, Italy, Japan, France, Greece, Portugal, Spain and Korea. The same formulation providing 12 months protection (ProHeart SR® 12, PH12) is marketed in Australia since October 2000. PH12 contains three times as much moxidectin as PH6.

After the launch of the product, the Center of Veterinary Medicine (CVM) of the United States Food and Drug Administration (FDA) expressed concern about the number and seriousness of adverse event reports (AERs), most of which were submitted by FDAH to FDA based on field reports from veterinarians and dog owners. On 03 Sep 2004, based on continued FDA concerns, FDAH voluntarily recalled the product from the US market. The recall prompted regulatory authorities in Canada, Japan, Europe and Australia to review the safety of PH6 and PH12, respectively, including the PH6 adverse event reports from the US. These authorities subsequently allowed continued marketing of PH6 or PH12, respectively, for canine heartworm control. Neither the US FDA, nor any of the other regulatory agencies voiced any concerns about an oral tablet formulation of moxidectin for the prevention of heartworm in dogs, nor any other moxidectin containing drugs approved for veterinary use in other species.

The initiation of this study was put on hold to thoroughly examine the PH6-related data, all moxidectin related toxicology data and the adverse event data from the human volunteer studies within the organization of each sponsor as well as with outside consultants. The conclusion of these examinations was that the PH6 related data do not suggest that the assessment of the risk for subjects in this trial needs to be changed. This conclusion is based on the following facts:

- The additional review of the existing Phase I safety data by Wyeth medical personnel, did not reveal any signals for potential safety concerns.
- The additional preclinical data on moxidectin acquired by FDAH in collaboration with Wyeth have reconfirmed that moxidectin is a very safe drug candidate.

- In May 2005, WHO consulted with independent experts on the safety data for moxidectin and Proheart 6 and their implications for the conduct of the planned study in Ghana. The experts unanimously concluded that the data on PH6 have no relevance for the evaluation of moxidectin tablets in humans and that they do not provide any scientific reason not to initiate the study in Ghana and recommended that the study be initiated as soon as possible.

- In May 2005, FDA CVM approved an injectable formulation of moxidectin for treatment of cattle (CYDECTIN®), which clearly shows that any FDA CVM concerns are related to the Proheart 6 formulation, not moxidectin.

11. TRIAL DESIGN

11.1 Description

This is a randomized, ivermectin-controlled, double-blind, single-ascending-dose, parallel design, inpatient/outpatient study of moxidectin administered to subjects of both sexes with different degrees of severity of *O. volvulus* infection. A total of 192 subjects will be enrolled in consecutive cohorts and will receive a single oral dose of moxidectin or a standard dose of ivermectin. Moxidectin is available in 2 mg TIC (tablets in capsules). Three (3) dose levels of moxidectin will be studied: 2 mg, 4 mg, and 8 mg. Therefore, the dose of moxidectin will be either 1 TIC for the 2mg, 2 TIC for the 4 mg, or 4 TIC for the 8 mg dose group.

Ivermectin will be dosed according to the approved weight based dosing schedule (see section 17.1).

To ensure blinding, moxidectin-treated and ivermectin-treated subjects may receive a number of placebo capsules so that each subject receives 4 capsules. All doses will be given after an overnight fast and breakfast will be delayed for about 2 hours.

Enrollment will be by dose and severity of infection as indicated below. The intensity of infection will be based on the mean of the densities at the iliac crests and calves. At each dose level, subjects with > 0 and < 10 microfilariae/mg of skin must have no ocular involvement. The sum of microfilariae in the two eyes must be ≤ 10 in subjects with 10-20 microfilariae/mg of skin.

- Cohort 1: 2 mg, 16 subjects with > 0 and < 10 microfilariae/mg of skin

- Cohort 2: 2 mg, 16 subjects with 10-20 microfilariae/mg of skin and the sum of microfilariae in the two eyes must be ≤ 10
- Cohort 3: 2 mg, 32 subjects with > 20 microfilariae/mg of skin with or without ocular involvement (this cohort will be enrolled in two groups of 16 subjects due to the capacity of the site)

- Cohort 4: 4 mg, 16 subjects with > 0 and < 10 microfilariae/mg of skin
- Cohort 5: 4 mg, 16 subjects mg, with 10-20 microfilariae/mg of skin and the sum of microfilariae in the two eyes must be ≤ 10
- Cohort 6: 4 mg, 32 subjects with > 20 microfilariae/mg of skin with or without ocular involvement (this cohort will be enrolled in two groups of 16 subjects due to the capacity of the site)

- Cohort 7: 8 mg, 16 subjects with > 0 and < 10 microfilariae/mg of skin
- Cohort 8: 8 mg, 16 subjects with 10-20 microfilariae/mg of skin and the sum of microfilariae in the two eyes must be ≤ 10
- Cohort 9: 8 mg, 32 subjects with > 20 microfilariae/mg of skin with or without ocular involvement (this cohort will be enrolled in two groups of 16 subjects due to the capacity of the site)

The decision to move to the next cohort within one dose group will be made by the PI based on evaluation of all blinded data obtained during the first 30 days post treatment of the previously enrolled cohort(s). The PI will inform the sponsors of this decision prior to enrollment of the next cohort. If either of the sponsors disagrees, cohort progression will be put on hold until the disagreement is resolved.

The decision to move to the next higher dose group, will be made based on blinded review of the safety and microfilaricidal data obtained during the first 30 days post treatment of all previously enrolled cohorts. The PI will submit a recommendation to proceed or not to proceed after a detailed examination of the blinded data. The Wyeth Medical Monitor, the WHO Clinical Monitor and the WHO Project Manager will conduct their separate review of the blinded data. Wyeth will include a representative of its safety surveillance group in addition to the medical monitor for purposes of review. WHO may include its Clinical Coordinator in the review.

The Project Review Team (PRT), consisting of Wyeth and WHO/TDR reviewers and the PI, will discuss the blinded data and formulate a recommendation to progress or not. The recommendation will be provided together with the rationale for this recommendation to the Clinical Expert Review Team (CERT)

consisting of three clinical experts (not otherwise associated with the conduct of the study) who have access to the randomized dose assignments in patient envelopes and have reviewed the data in parallel with the PRT. The CERT will initially review the data in a blinded fashion. They can make their recommendation on dose progression based on blinded data. If they need to unblind, they may do so for individual patient(s), or they may unblind the entire cohort. In either case, they will inform the PRT of their recommendation on dose progression without disclosing if the decision was made on blinded or unblinded data. If there is a difference of opinion within the PRT regarding the dose progression recommendation, the different recommendations and their rationale will be provided to the CERT. The CERT will provide their recommendation to PRT. If there is not unanimous agreement among the CERT, they will provide their differing recommendations and their rationale. If there is not unanimous agreement on proceeding to the next dosing cohort between the PRT, and a majority of the CERT, the data will be discussed in a blinded fashion. If no agreement can be reached, unblinded data will be made available to the PRT and discussed. If upon discussion of the unblinded data there is no unanimous agreement to proceed to the next higher dose group, dose progression will not occur.

The diagram below provides an overview of the process from enrollment of the first cohort (2 mg) to decision to progress to the 4 mg dose as well as treatment and study participation for each subject. The same process will be followed for the cohorts in the 4 mg dose group and the decision to move to the 8 mg dose.

Enrollment Process and Dose Progression Decision Diagram

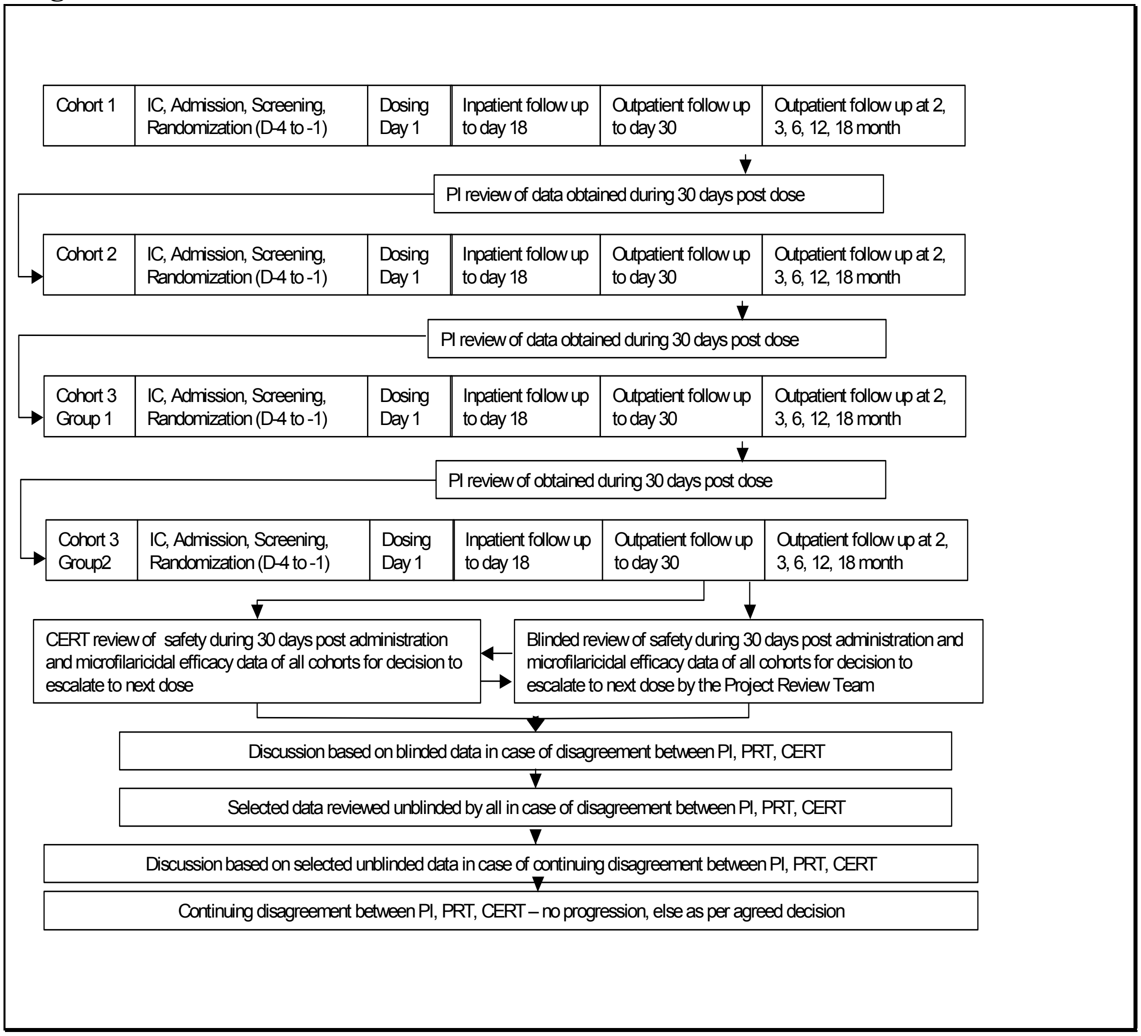

A schematic presentation of procedures is provided in the subject flowchart (section 9).

12. STUDY OBJECTIVES

12.1 Primary

The primary objective of this study is to determine the safety and tolerability of orally administered moxidectin in subjects with *O. volvulus* infection, as measured by the incidence of clinical AEs and clinically significant laboratory test results.

12.2 Secondary

The secondary objectives of this study are:

1. To determine doses that effectively eliminate microfilariae and prevent their reaccumulation in the skin as measured by the skin microfilarial loads at day 8, 1 month, 2 months, 3 months, 6 months, 12 months and 18 months after treatment.
2. To determine the viability and fertility of adult worms at 18 months.
3. To assess the pharmacokinetics of moxidectin in adult male and female subjects.

13. SELECTION OF STUDY POPULATION

This study will be conducted in otherwise healthy subjects with *O. volvulus* infection from the OCRC study area in the basin of River Tordzi, located in the forest area of south-eastern Ghana and within a 2 to 2⅔ hours journey from Hohoe, the location of the study site. The Onchocerciasis Control Programme (OCP) had established in 1988, during the southern extension of the program, that transmission of onchocerciasis in the area involved forest vectors exclusively and hence no control activities were instituted. The area was also not included in the ivermectin distribution program launched by the Directorate for Onchocerciasis Control of the Ministry of Health, as the area is largely hypoendemic for onchocerciasis. Extensive clinical, ophthalmologic and parasitological surveys carried out by the OCRC showed that morbidity due to onchocerciasis was low. The vast majority of adults had no skin or ocular lesions. Where ocular lesions were found they were mainly non-onchocercal. This was in line with the observation during initial contact with the area that most villages had never heard of onchocerciasis, the *Simulium* fly, or of ivermectin and very few had attended any health institution for skin or ocular problems or for the excision of nodules. Thus the OCRC only offered individualized treatment with ivermectin to those found during surveys to have clinically significant onchocercal lesions. The communities were informed of the existence elsewhere of mass treatment with ivermectin and the availability of the drug at nearby centers.

Subjects will be members of a "grand cohort" who were previously prescreened in their villages and the hospital and who have been dormant, pending further testing and enrollment in the appropriate clinical trial.

The 'grand cohort' was identified previously in a step-wise process consisting of (1) identification of potentially suitable villages based on entomological data collected by the Onchocerciasis Control Programme, and/or indirectly via OCRC out-patients with high skin microfilaria counts from these

villages, and/or known breeding sites of *Simulium* close to these village and/or village residents reporting a high prevalence of onchocerciasis and *Simulium* bite rates, (2) an initial meeting with the village chief, elders and opinion leaders during which the information provided in a 'generic information sheet' (e.g. background on onchocerciasis, purpose of the OCRC, purpose and procedures for screening, general purpose and procedures for three types of studies (clinical drug efficacy and safety study, 'ex vivo' study of the efficacy of drugs on the macrofilariae, PK study) is presented and discussed, (3) a meeting with the community in which the same information was presented and discussed, after the village chief, elders and opinion leaders had provided their agreement to such a meeting, (4) a village survey, after the community had agreed to that, (5) a screen at Onchocerciasis Chemotherapy Research Centre in the Government Hospital at Hohoe for those subjects that, based on the village survey, had *O. volvulus* infection, were 18-60 years old, had no obvious exclusion factors, and had agreed to the hospital screen. The results of the screening were communicated to the subjects. Subjects who could potentially qualify for a clinical trial form the 'grand cohort' from which subjects for future studies will be recruited. Informed consent was obtained at all stages of contact with the communities listed above. The "generic" informed consent form was the basis for the transfer of information and the subsequent discussions. The consent prior to the hospital-screening phase was verbal and involved thousands of people in the initial stages and a few hundred in the hospital-screening phase.

For recruitment for this study, subjects on the 'grand cohort' list will be asked to come for screening per cohort, based on position of their name on the list, gender and microfilaria counts. If a subject asked to come for screening for a particular cohort meets all inclusion criteria and has none of the exclusion criteria, but has a microfilaria count too high or too low for that cohort, they will be offered the choice between (1) enrollment into the next cohort for which they qualify based on their microfilaria count or (2) nodulectomy and ivermectin treatment. If their microfilaria count is too low or too high for any cohort planned in the future, they will be offered nodulectomy and ivermectin treatment. The same will happen, if they do not meet one of the other inclusion criteria or meet an exclusion criterion (see figure below) or if 16 subjects higher on the grand cohort list have already qualified for the study.

All members of the grand cohort who are invited for this study participate in a written and signed or thumb printed informed consent process. During this process, the PI or sub-investigator informs the subjects of all the elements specific to the study. An information sheet that is specific to the study and is available in English and the local language replaces the generic information sheet. Adequate time is then allowed, as during the earlier discussion, for the subject to ask questions and make a voluntary decision.

No protocol-specific procedures, including transport to the study site and screening, will be performed until the subject has signed or thumb printed and dated an ethics committee (EC) approved informed consent form. The form will be witnessed by a member of the community (impartial witness) and countersigned by the investigator. A copy will be given to the subject. The study begins at the time the first subject is admitted to the hospital for the study. Subjects must meet all the inclusion and none of the exclusion criteria to be enrolled in the study.

OCRC informs each subject individually about the details of the study, which concern him/her from the time of enrollment to completion. This includes AEs, lab results etc. and the final status. If there are any outstanding medical issues, further advice is given.

Following completion of the study, the PI and his staff will seek an appointment with the chiefs and the elders and discuss the overall findings and conclusions of the study with them. Subsequently, study results and conclusions are presented and discussed at a village durbar. The discussions with chiefs and elders as well as those at the village durbar include the implications of the study results for future studies on moxidectin as well as other research.

Consent Process Diagram

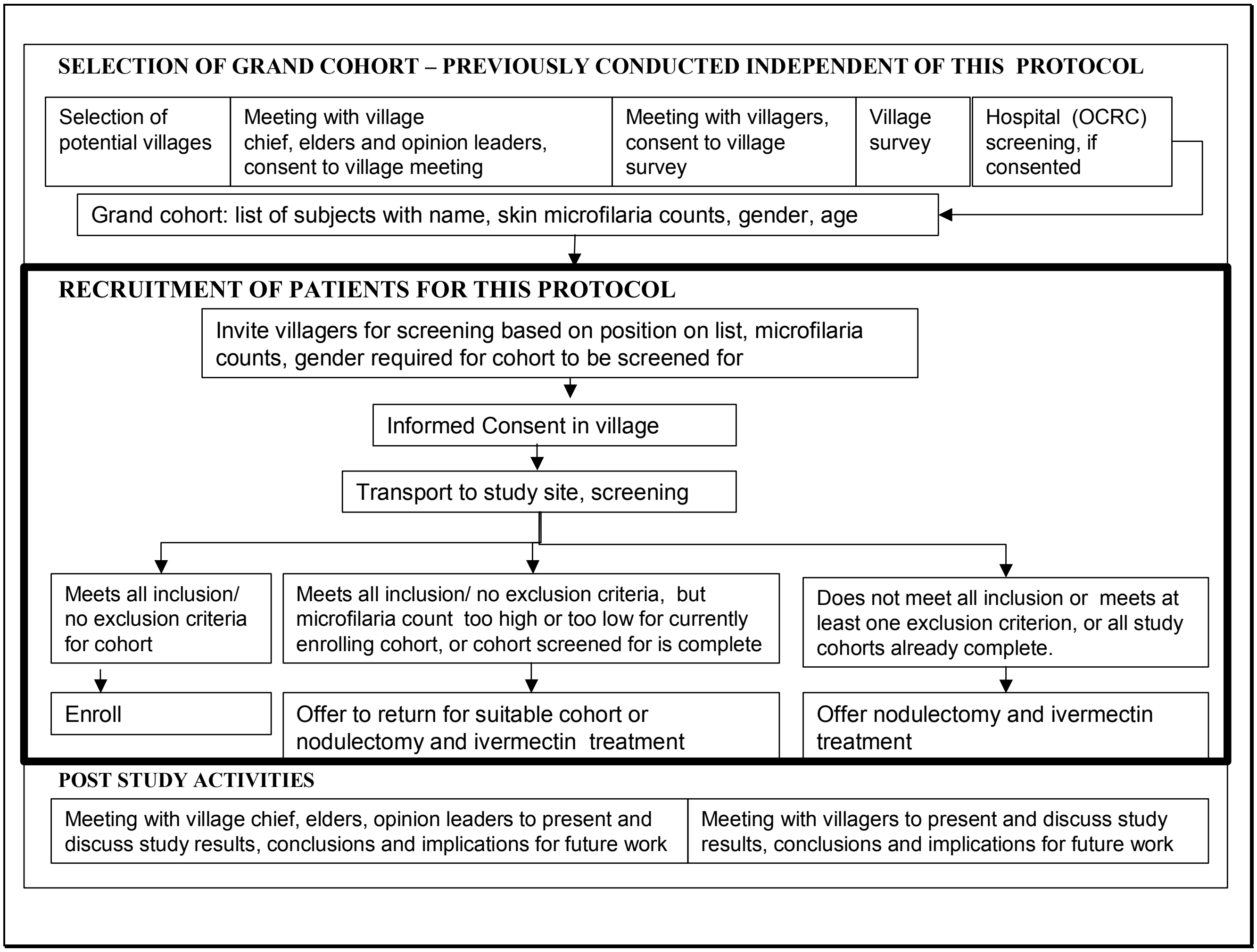

13.1 Inclusion Criteria

Men and women in good general health, with *O. volvulus* infection and:

a) Written, signed (or thumb-printed), and dated informed consent
b) Aged 18 to 60 years, inclusive
c) Body weight ≥ 40 kg for women and ≥ 45 kg for men
d) Nonpregnant, nonbreastfeeding women. Women of child-bearing potential must agree to use birth control during the first 150 days after treatment.
e) Healthy, as determined by a physician on the basis of a physical examination, ECG, and a thorough review of the medical history and clinical laboratory results
f) Adequate hematologic, renal, and hepatic function, defined as:
    1) White blood cell (WBC) count ≥ 2,800 and ≤ 11,300 cells/mL
    2) Hemoglobin: ≥ 11.0 g/dL for men and ≥ 10.0 g/dL for women
    3) Platelet count: ≥ 110,000 mm3
    4) Serum creatinine: ≤ 1.25 x upper limit of normal (ULN)
    5) Total bilirubin: ≤ 1.25 x ULN
    6) AST/SGOT: ≤ 1.25 x ULN
    7) AP: ≤ 1.25 x ULN
    8) Prothrombin time WNL
    9) Urinalysis WNL
g) Skin microfilarial density within the required range for the cohort

13.2 Exclusion Criteria

a) Participation in any studies other than purely observational ones, within 4 weeks before test article administration.
b) Any vaccination within 4 weeks before test article administration
c) Acute infection requiring therapy within the last 10 days before test article administration
d) Administration of any medication (with the exception of medication required to treat any reactions during the screening fluorescein angiography (chlorpheniramine) or paracetamol) or herbal preparation within 10 days prior to test article administration or any condition currently requiring regular medication
e) Clinically significant ECG abnormalities or history of cardiac abnormality

f) Past or current history of neurological or neuropsychiatric disease or epilepsy
g) Subjects with orthostatic hypotension at the screening evaluation
h) History of drug or alcohol abuse or regular use of ≥ 3 cigarettes per day
i) Use of alcohol or other drugs of abuse within 72 hours before test article administration
j) Any condition, in the investigator's opinion, that places the subject at undue risk
k) Subjects who have donated blood within 8 weeks before study entry
l) Subjects with ocular onchocerciasis in cohorts intended to enroll subjects with mild infection. Ocular onchocerciasis is defined by presence of live or dead microfilariae, onchocercal punctate opacities, onchocercal lesions of the posterior segment or lesions that mimic those seen in onchocerciasis.
m) Subjects with hyperreactive onchodermatitis
n) Antifilarial therapy within the previous 5 years
o) Coincidental infection with *Loa Loa*
p) Female subjects of childbearing potential with a contraindication to DMPA if not on Norplant
q) Any other condition which the investigator feels would exclude the subject from the study

Subjects who are excluded from the study or subjects who are withdrawn from the study for medical reasons will be advised where to seek assistance.

13.3 Number of Subjects

One hundred and ninety-two (192) subjects including both sexes, aged 18 to 60 years are expected to enroll in this study. There will be 9 cohorts. The first 2 cohorts at each dose level will comprise 16 subjects while the third cohort will comprise 32 subjects, which will be enrolled in 2 successive groups of 16. The study will aim to enroll equal numbers of men and women at each dose level and intensity of infection.

13.4 Screen Failures

Subjects who sign an informed consent form and fail to meet the inclusion and/or exclusion criteria are defined as screen failures. All screen failures should be recorded in the screening and enrollment log. A copy of the log should be retained in the investigator's study files. No case report forms (CRFs) will be completed for screen failures. Subjects who are excluded on medical grounds will be advised where to seek assistance. All screen failures will be offered nodulectomy and ivermectin treatment.

14. PRIOR TREATMENT

Reasonable efforts will be made to determine all relevant treatment received by the subject within 10 days prior to study drug administration. All relevant information, including the names of any procedures, drugs, or herbal or dietary supplements used will be recorded in the subject's CRF. Include the name of the procedure or drug and other information required on the CRF. Subjects must not have taken any medication, prescription or over-the-counter (OTC), within 10 days before administration of the study drug (with the exception of medication required to treat any reactions during the screening fluorescein angiography (chlorpheniramine) or paracetamol). Paracetamol 1.0g as required (maximum 3.0g per day) will be given to subjects with painful conditions. Subjects are transported on a 2½-hour journey on bumpy roads from their villages to the centre. Inevitably, diffuse aches and pains that require relief result. Additionally, headaches often occur after maximal papillary dilation for colour fundus photography and fluorescein angiography using phenylephrine hydrochloride 2.5% followed by tropicamide 1%. Subjects must also have discontinued use of herbal and dietary supplements at least 10 days before study drug administration.

15. CONCOMITANT TREATMENT

15.1 Additional Required Drugs

Depo-medroxyprogesterone acetate (DMPA), 150 mg, will be administered twice to all women of childbearing potential. The first dose will be given on study day 11 and the second dose will be at month 2 (±1 week). Women who have contraceptive protection by the Norplant system will be excluded from the DMPA injections. Dosing will be collected on the CRF.

15.2 Permitted Treatment

All medication given after admission to the hospital will be documented in the CRF.

Chlorpheniramine 4mg will be administered 30 minutes before injection to subjects who have reacted to a previous dose of fluorescein sodium. Adverse reactions to fluorescein sodium occur in 18 to 38% of subjects. They manifest as nausea, vomiting, abdominal pain, itching urticaria, facial or lip swelling, chills and dry cough. Pretreatment with chlorpheniramine ameliorates the adverse events in most, but not all subjects

15.3 Prohibited Treatment

Smoking, the ingestion of alcoholic beverages, grapefruit, or grapefruit juice, and the use of non-prescribed drugs will not be permitted during the 18 day admission period. Treatment with other anthelmintic agents including ivermectin will be prohibited for the duration of the study. If anthelmintic treatment has been taken for any reason, the subject will be withdrawn from the study.

16. TREATMENT OF SUBJECTS

16.1 Procedures

See flowchart(s) for the procedures to be performed at each visit.

16.1.1 Days -4 to -2 (Visit 1)

Subjects will be admitted to the OCRC at the beginning of this period and the following procedures and evaluations will be performed:

- Informed consent will be obtained prior to transport of the potential study participants from the village to the study center and before any study-related procedures are performed.
- Demographics (age, tribal origin, gender), medical history, medication history (all prescription and over-the-counter medications taken within 10 days prior to study drug administration) will be recorded.
- A complete physical examination, including the subject’s height and weight, and a review of body systems, will be performed by a licensed physician.
- A twelve-lead ECG will be performed (See section 16.3.3, Special Methods, Electrocardiograms).
- An ocular examination, including fundus colour photography and fluorescein angiography for all subjects.
- Vital sign measurements (supine pulse rate and blood pressure [see section 16.3.2 Vital signs], repeated after standing for at least 2 minutes; respiratory rate, and oral temperature) will be taken up to 12 times throughout this screening period.
- An evaluation of any AEs will be conducted during waking hours as other activities permit.
- A complete laboratory evaluation including hematology with prothrombin time, G6PD, hemoglobin electrophoresis and nucleopore filtration for microfilariae, serum chemistry (first morning fasted values) and a routine urinalysis and nucleopore filtration for microfilariae (see section 16.3.5, Special Methods, Laboratory Evaluations/Specimen Collection).
- Skin snips will be taken from both iliac crests and calves to determine microfilarial density.

16.1.2 Day -1 (Visit 2)

On day -1, the final list of study participants will be drawn up.

- A pregnancy test (serum β-hCG) will be performed for all women of childbearing potential and the results known prior to study drug administration.
- Vital sign measurements (supine pulse rate and blood pressure [see section 16.3.2 Vital signs], repeated after standing for at least 2 minutes; respiratory rate, and oral temperature).
- Adverse events assessment
- Randomization (once all screening data have been completed, and the subject meets the inclusion/exclusion criteria, a subject will be randomly assigned to receive moxidectin or ivermectin therapy).
- Packaging, dispensing and labeling of test articles. These will be locked up until the morning of day 1.

16.1.3 Day 1 (Visit 3)

On day 1, the following evaluations/procedures will be performed:

- A pre-treatment blood sample for PK analysis (obtained within 2 hours before drug administration)
- Test articles administration at 0700 ±1hours
- Vital sign measurements (supine pulse rate and blood pressure [see section 16.3.2 Vital signs], repeated after standing for at least 2 minutes; respiratory rate, and oral temperature) will be taken approximately 3 times in the 24 hour period.
- Adverse events assessment
- Blood samples for PK analysis at 1, 2, 4, and 8 hours (each time point ±15 minutes) post study drug administration
- Interim physical examination including weight
- Twelve-lead ECG approximately 4 hours after dose administration
- Laboratory evaluations including hematology with prothrombin time and nucleopore filtration for microfilariae, serum chemistries (first morning fasted values), and routine urinalysis and nucleopore filtration for microfilariae.

16.1.4 Day 2 (Visit 4)

On day 2, the following evaluations/procedures will be performed:

- Vital sign measurements (supine pulse rate and blood pressure [see section 16.3.2 Vital signs], repeated after standing for at least 2 minutes; respiratory rate, and oral temperature) will be taken approximately 5 times in the 24 hour period.
- Adverse events assessment
- Interim physical examination including weight
- Twelve-lead ECG
- Laboratory evaluations including hematology with prothrombin time and nucleopore filtration for microfilariae, serum chemistries (first morning fasted values), and routine urinalysis and nucleopore filtration for microfilariae.
- Blood sample for 24 hour (±15 min) post study drug administration for PK analysis.

16.1.5 Day 3 (Visit 5)

On day 3, the following evaluations/procedures will be performed:

- Vital sign measurements (supine pulse rate and blood pressure [see section 16.3.2 Vital signs], repeated after standing for at least 2 minutes; respiratory rate, and oral temperature) will be taken approximately 5 times in the 24 hour period.
- Adverse events assessment
- Interim physical examination including weight
- Twelve-lead ECG
- Ocular examination for all subjects, including fundus colour photography and fluorescein angiography (Day 3 or 4)

16.1.6 Day 4 (Visit 6)

On day 4, the following evaluations/procedures will be performed:

- Vital sign measurements (supine pulse rate and blood pressure [see section 16.3.2 Vital signs], repeated after standing for at least 2 minutes; respiratory rate, and oral temperature) will be taken approximately 5 times in the 24 hour period.
- Adverse events assessment
- Interim physical examination including weight
- Blood sample for 72 hour (±15 min) post study drug administration for PK analysis.
- Laboratory evaluations including hematology with prothrombin time and nucleopore filtration for microfilariae, serum chemistries (first morning fasted values), and routine urinalysis and nucleopore filtration for microfilariae.

16.1.7 Day 5 (Visit 7)

On day 5, the following evaluations/procedures will be performed:

- Vital sign measurements (supine pulse rate and blood pressure [see section 16.3.2 Vital signs], repeated after standing for at least 2 minutes; respiratory rate, and oral temperature) will be taken approximately 5 times in the 24 hour period.
- Adverse events assessment
- Interim physical examination including weight

16.1.8 Day 6 (Visit 8)

On day 6, the following evaluations/procedures will be performed:

- Vital sign measurements (supine pulse rate and blood pressure [see section 16.3.2 Vital signs], repeated after standing for at least 2 minutes; respiratory rate, and oral temperature) will be taken approximately 5 times in the 24 hour period.
- Adverse events assessment
- Interim physical examination including weight

16.1.9 Day 7 (Visit 9)

On day 7, the following evaluations/procedures will be performed:

- Vital sign measurements (supine pulse rate and blood pressure [see section 16.3.2 Vital signs], repeated after standing for at least 2 minutes; respiratory rate, and oral temperature) will be taken approximately 5 times in the 24 hour period.
- Adverse events assessment
- Interim physical examination including weight
- Ocular examination for all subjects, including fundus colour photography and fluorescein angiography (Day 7 or 8)

16.1.10 Day 8 (Visit 10)

On day 8, the following evaluations/procedures will be performed:

- Vital sign measurements (supine pulse rate and blood pressure [see section 16.3.2 Vital signs], repeated after standing for at least 2 minutes; respiratory rate, and oral temperature) will be taken approximately 5 times in the 24 hour period.
- Adverse events assessment

- Twelve lead ECG
- Interim physical examination including weight
- Blood sample for Day 8 (±1 hour) post study drug administration for PK analysis.
- Laboratory evaluations including hematology with prothrombin time and nucleopore filtration for microfilariae, serum chemistries (first morning fasted values), and routine urinalysis and nucleopore filtration for microfilariae.
- Skin snips

16.1.11Day 9 (Visit 11)

On day 9, the following evaluations/procedures will be performed:

- Vital sign measurements (supine pulse rate and blood pressure [see section 16.3.2 Vital signs]; respiratory rate, and oral temperature) will be taken 2 times per day.
- Adverse events assessment
- Interim physical examination including weight

16.1.12 Day 10 (Visit 12)

On day 10, the following evaluations/procedures will be performed:

- Vital sign measurements (supine pulse rate and blood pressure [see section 16.3.2 Vital signs], respiratory rate, and oral temperature) will be taken 2 times per day.
- Adverse events assessment
- Interim physical examination including weight

16.1.13 Day 11 (Visit 13)

On day 11, the following evaluations/procedures will be performed:

- Vital sign measurements (supine pulse rate and blood pressure [see section 16.3.2 Vital signs], respiratory rate, and oral temperature) will be taken 2 times per day.
- Adverse events assessment
- Interim physical examination including weight
- DMPA injection to women of child bearing potential (except those protected by the Norplant system)

16.1.14 Day 12 (Visit 14)

On day 12, the following evaluations/procedures will be performed:

- Vital sign measurements (supine pulse rate and blood pressure [see section 16.3.2 Vital signs], respiratory rate, and oral temperature) will be taken 2 times per day.
- Adverse events assessment
- Interim physical examination including weight

16.1.15 Day 13 (Visit 15)

On day 13, the following evaluations/procedures will be performed:

- Vital sign measurements (supine pulse rate and blood pressure [see section 16.3.2 Vital signs], respiratory rate, and oral temperature) will be taken 2 times per day.
- Adverse events assessment
- Interim physical examination including weight
- Laboratory evaluations including hematology with prothrombin time and nucleopore filtration for microfilariae, serum chemistries (first morning fasted values), and routine urinalysis and nucleopore filtration for microfilariae.
- Blood sample for Day 13 (±1 hour) post study drug administration for PK analysis

16.1.16 Day 14 (Visit 16)

On day 14, the following evaluations/procedures will be performed:

- Vital sign measurements (supine pulse rate and blood pressure [see section 16.3.2 Vital signs], respiratory rate, and oral temperature) will be taken 2 times per day.
- Adverse events assessment
- Interim physical examination including weight
- Ocular examination for all subjects, including fundus colour photography and fluorescein angiography (Day 14 or 15)

16.1.17 Day 15 (Visit 17)

On day 15, the following evaluations/procedures will be performed:

- Vital sign measurements (supine pulse rate and blood pressure [see section 16.3.2 Vital signs], respiratory rate, and oral temperature) will be taken 2 times per day.
- Adverse events assessment

- Interim physical examination including weight

16.1.18Day 16 (Visit 18)

On day 16, the following evaluations/procedures will be performed:

- Vital sign measurements (supine pulse rate and blood pressure [see section 16.3.2 Vital signs], respiratory rate, and oral temperature) will be taken 2 times per day.
- Adverse events assessment
- Interim physical examination including weight

16.1.19 Day 17 (Visit 19)

On day 17, the following evaluations/procedures will be performed:

- Vital sign measurements (supine pulse rate and blood pressure [see section 16.3.2 Vital signs], respiratory rate, and oral temperature) will be taken 2 times per day.
- Adverse events assessment
- Interim physical examination including weight

16.1.20 Day 18 (Visit 20)

On day 18, the following evaluations/procedures will be performed. Subjects will be discharged after all procedures are completed.

- Vital sign measurements (supine pulse rate and blood pressure [see section 16.3.2 Vital signs], respiratory rate, and oral temperature) will be taken 1 time prior to discharge.
- Adverse events assessment
- Laboratory evaluations including hematology with prothrombin time and nucleopore filtration for microfilariae, serum chemistries (first morning fasted values), and routine urinalysis and nucleopore filtration for microfilariae
- Blood sample for Day 18 (±1 hour) post study drug administration for PK analysis.

16.1.21 1-month (Visit 21)

At 1 month (±1 week), subjects will return to the center for an outpatient visit. They will be questioned about their overall health, including any changes in health status and the indication for use of any medication. The following evaluations/procedures will be performed:

- Vital sign measurements (supine blood pressure and heart rate [see section 16.3.2 Vital signs], respiratory rate, and oral temperature)

- Adverse events assessment
- Physical examination including weight
- Laboratory evaluations including hematology with prothrombin time and nucleopore filtration for microfilariae, serum chemistries (first morning fasted values), and routine urinalysis and nucleopore filtration for microfilariae
- Skin snips
- Blood sample for 1 month (±1 week) post study drug administration for PK analysis.
- Ocular examination for all subjects, including fundus colour photography and fluorescein angiography

16.1.22 2-Months (Visit 22)

At 2 months (±1 week), subjects will return to the center for an outpatient visit. They will be questioned about their overall health, including any changes in health status and the indication for use of any medication. The following evaluations/procedures will be performed:

- Vital sign measurements (supine blood pressure and heart rate [see section 16.3.2 Vital signs], respiratory rate, and oral temperature)
- Adverse events assessment
- Physical examination including weight
- Laboratory evaluations including hematology with prothrombin time and nucleopore filtration for microfilariae, serum chemistries (first morning fasted values), and routine urinalysis and nucleopore filtration for microfilariae
- Skin snips
- Blood sample for 2 months (±1 week) post study drug administration for PK analysis.
- Ocular examination for all subjects, including fundus colour photography and fluorescein angiography.
- DMPA injection to women of child bearing potential (except those protected by the Norplant system)

16.1.23 3-Months (Visit 23)

At 3 months (±1 week), subjects will return to the center for an outpatient visit. They will be questioned about their overall health, including any changes in health status and the indication for use of any medication. The following evaluations/procedures will be performed:

- Vital sign measurements (supine blood pressure and heart rate[see section 16.3.2 Vital signs], respiratory rate, and oral temperature)
- Adverse events assessment
- Physical examination including weight
- Laboratory evaluations including hematology with prothrombin time and nucleopore filtration for microfilariae, serum chemistries (first morning fasted values), and routine urinalysis and nucleopore filtration for microfilariae
- Skin snips
- Blood sample for 3 months (±1 week) post study drug administration for PK analysis.
- Ocular examination for all subjects, including fundus colour photography and fluorescein angiography.

16.1.24 6-Months (Visit 24)

At 6 months (±1 month), subjects will return to the center for an outpatient visit. They will be questioned about their overall health, including any changes in health status and the indication for use of any medication. The following evaluations/procedures will be performed:

- Vital sign measurements (supine blood pressure and heart rate [see section 16.3.2 Vital signs], respiratory rate, and oral temperature)
- Adverse events assessment
- Physical examination including weight
- Laboratory evaluations including hematology with prothrombin time and nucleopore filtration for microfilariae, serum chemistries (first morning fasted values), and routine urinalysis and nucleopore filtration for microfilariae.
- Skin snips
- Ocular examination for all subjects. Colour fundus photography and fluorescein angiography only for subjects with lesions or visual defects determined during ocular examination or previous fundus color photography or fluorescein angiography.
- Blood sample for 6 months (±1 month) post study drug administration for PK analysis.
- Blood and urine samples will be filtered and examined for microfilariae and ultrasonography of all located nodules will be performed for the WHO substudies.

16.1.25 12-Months (Visit 25)

At 12 months (± 1 month), subjects will return to the center for an outpatient visit. They will be questioned about their overall health, including any changes in health status and the indication for use of any medication. The following evaluations/procedures will be performed:

- Vital sign measurements (supine blood pressure and heart rate [see section 16.3.2 Vital signs], respiratory rate, and oral temperature)
- Adverse events assessment
- Physical examination including weight
- Laboratory evaluations including hematology with prothrombin time and nucleopore filtration for microfilariae, serum chemistries (first morning fasted values), and routine urinalysis and nucleopore filtration for microfilariae
- Skin snips
- Ocular examination for all subjects. Colour fundus photography and fluorescein angiography only for subjects with lesions or visual defect determined during ocular examination or previous fundus color photography or fluorescein angiography.
- Blood sample for 12 months (±1 week) post study drug administration for PK analysis.

16.1.26 18-months (Visit 26)

At month 18 (± 1 month), subjects will return to the center for an outpatient visit. They will be questioned about their overall health, including any changes in health status and the indication for use of any medication. The following evaluations/procedures will be performed:

- Vital sign measurements (supine blood pressure and heart rate [see section 16.3.2 Vital signs], respiratory rate, and oral temperature)
- Adverse events assessment
- Physical examination including weight
- Laboratory evaluations including hematology with prothrombin time and nucleopore filtration for microfilariae, serum chemistries (first morning fasted values), and routine urinalysis and nucleopore filtration for microfilariae
- Skin snips
- Ocular examination for all subjects. Color fundus photography and fluorescein angiography only for subjects with lesions or visual defect determined during ocular examination or previous fundus color photography or fluorescein angiography.
- Nodulectomy

- All subjects who have skin microfilariae will be treated with the approved dosage of ivermectin.

16.2 Discontinuation and Withdrawal of Subjects

- Subjects who withdraw their consent before administration of the test article will be replaced.
- Subjects cannot be withdrawn from therapy because this study involves only a single dose administration.
- A subject can be withdrawn from the study after test article administration if continued follow up jeopardizes the subject's health in the judgment of the principal investigator (e.g. an illness that contra-indicates follow up visits).
- A subject who terminates participation or is withdrawn from the study by the PI after test article administration will be considered as having discontinued from the study and will not be replaced unless the number exceeds 2 subjects in any 16 subject group and the terminations occur before day 7.
- PK data will not be collected on subjects who replace those who have withdrawn post drug administration.
- Replacement subjects will be assigned to the same cohort and treatment as the subjects leaving the study and will need to complete safety evaluations at 1 month (± 1 week) before subsequent cohorts are enrolled.
- Replacement subject numbers will be the number of the subject that they replace plus 10000. Subject numbers for the 9 cohorts without replacements are from 101-1216, replacements would be assigned numbers 10101-11216.

Subjects manifesting serious medical illnesses should be discussed with the clinical coordinator and medical monitor(s). The PI will be responsible for any decision regarding the subject's discontinuation from the study.

Subjects that terminate before the end of the study should have the procedures performed that are indicated for the 18-month visit on the study flow chart. Nodulectomy will be performed if the subject agrees but the results will not be collected on the CRF. A reasonable effort will be made to ascertain the reasons for withdrawal from the study or failure to return and these will be recorded in the CRF. This protocol only requires administration of one dose of study drug; therefore, subjects with AEs will not have to be withdrawn since no further study drug will be administered.

The administration of the test article(s) may be delayed for up to 2 days to permit all predose examinations to be performed on replacement subjects.

16.3 Special Methods

16.3.1 Physical Examinations and Medical History

A complete physical examination will be performed at baseline, and at 1, 2, 3, 6, 12, and 18 months. These will include height (cm, on admission only), weight (kg), vital signs, supine pulse rate and blood pressure, respiratory rate, and oral temperature, and a complete review of body systems. A summary of the dermatologic findings including nodules and onchocercal skin lesions will be entered in the CRFs.

Medical history will be obtained at baseline and will include a history of both systemic and ocular symptoms. These symptoms will be monitored for any changes in severity that could be attributed to test drug administration.

Interim physical examinations conducted during the admission period will include weight (kg), vital signs, and a review of body systems to determine whether there have been any significant changes since the previous examination.

At each post-discharge follow-up visit the subject will be questioned regarding any recent illness or changes in health status, and any medication taken and for what indication. All findings will be recorded, any significant changes investigated, and relationship to test drug administration defined.

16.3.2 Vital Signs

Vital signs including pulse rate, blood pressure, respiratory rate, and oral temperature (centigrade) will be measured after the subject has been in a supine position for at least 5 minutes. During the screening period and on Day 1 to Day 8, pulse rate and blood pressure measurements will be repeated after the subject has been standing for 2 minutes. Measurements will be taken up to 12 times during the 4 day screening period, approximately 3 times per day on day 1, 5 times per day on day 2 to day 8, and twice a day for the remainder of the inpatient period. Vital signs measurements including pulse rate, supine blood pressure, respiratory rate, and oral temperature (centigrade) will also be made at each outpatient visit.

16.3.3 Electrocardiograms

Twelve-lead ECGs will be performed during baseline and on day 1 (approximately 4 hours following test article administration) and on days 2, 3, and 8. A physician experienced in evaluating ECGs will provide the interpretations of all ECGs. This should include the rate, rhythm, length of the PR, QRS and Q-T intervals, and any abnormalities noted. The results will be recorded in the CRFs. Additional ECGs will be performed at the discretion of the investigator, or where clinically indicated.

16.3.4 Ocular Examination

Ocular examinations will include examination of visual acuity, visual fields using a calibrated Goldmann perimeter, color vision, external ocular structures, ocular mobility and pupillary reflex. The anterior segment will be examined with a Haag-Streit 900 slit-lamp. Microfilariae in the anterior chamber will be counted after head-down positioning for 5 minutes. Living and dead microfilariae in the cornea and punctate opacities will be counted. Intraocular pressure will be measured and the dilated fundus examined by direct and indirect ophthalmoscopy. Color fundus photography and fluorescein angiography (using 20% fluorescein sodium) will be done on all subjects before dosage and at days 3 or 4, 7 or 8, 14 or 15, and at 1 and 3 months and will not be repeated unless lesions or visual defects are demonstrated during ocular examination or previous fundus color photography or fluorescein angiography. The color photographs and fluorescein angiograms will be retained at the study site. Descriptive abnormalities will be recorded on the case report forms.

16.3.5 Laboratory Evaluations/Specimen Collection

Hematologic studies, including prothrombin time, a complete blood cell count (CBC), including hematocrit, hemoglobin, WBC with differential, and platelet count, will be obtained. A G6PD and hemoglobin electrophoresis will also be obtained with the screening CBC. Blood will be filtered through a nucleopore membrane, stained with Giemsa and examined for microfilariae.

Serum chemistry tests will be performed, including sodium, potassium, chloride, bicarbonate, glucose, total protein, albumin, urea, creatinine, alkaline phosphatase, lactic dehydrogenase (LDH), total bilirubin, gamma-glutamyl transpeptidase (GGT), aspartate aminotransferase (AST/SGOT), and alanine aminotransferase (ALT/SGPT).

Routine urinalysis, using a 10-parameter dipstick (specific gravity, pH, protein, glucose, ketones, blood, bilirubin, urobilinogen, nitrite, leukocyte esterase) and microscopic evaluation will be performed. Urine will be filtered through a nucleopore membrane, stained with Giemsa and examined for microfilariae.

All specimens for hematologic and serum chemistry tests will be obtained with subjects in the fasting state.

A pregnancy test (serum β-hCG) will be performed at baseline on all women of childbearing potential.

Blood samples for PK analysis for moxidectin will be collected in 10 mL collection tubes containing heparin as an anticoagulant. The plasma will be processed and separated into 2 tubes according to Attachment 2. One set of samples will be sent to the bioanalytical laboratory for analyses. The other set of samples will be stored at Hohoe until all analyses are complete. All samples will then be discarded. PK data will not be collected on subjects who replace those who have withdrawn post drug administration.

16.3.6 Skin Snips

A total of 4 skins snips will be taken at each time point (from iliac crests and calves) using a corneoscleral punch (Walser or Holth-type). Each snip will be weighed on an analytical balance and incubated overnight in isotonic saline in a well of a flat-bottomed microtitre plate. The microfilariae that have emerged will be counted using an inverted microscope. The skin microfilarial density at each site will be documented as the number per mg of skin. One punch will be used per subject and punches will be sterilized between subjects using steam under pressure (autoclave).

17. TEST ARTICLE

17.1 Test Article(s) and Administration

Test article is administered only to subjects who have provided informed consent, fulfill all inclusion and exclusion criteria and have been assigned a randomization number by the unblinded third party pharmacist (see section 18.1). Once a randomization number and the associated test article has been assigned to a subject it must not be reassigned to another subject. All subjects will receive a total of 4

capsules. Matching placebo capsules will be administered as necessary so that each subject receives the same number of capsules independent of their treatment assignment.

The doses of moxidectin for this study are 2 mg, 4 mg, and 8 mg and will be administered to subjects in TIC, each HPMC capsule containing 2 mg moxidectin.

The moxidectin group will receive either 1, 2 or 4 moxidectin capsules based on the dosage for that group. (see Table 17.4.A and Attachment 4).

Ivermectin will be supplied as 3 mg tablets encapsulated in HPMC capsules and will be dosed according to the weight based dosing schedule approved for the treatment of onchocerciasis in the ivermectin package insert. (See Table 17.4.A and Attachment 4).

The recommended dosage of ivermectin in the treatment of onchocerciasis is a single oral dose designed to provide approximately 150 µg/kg of body weight.

The capsules will be swallowed with water.

**Ivermectin Weight based Dosing Schedule**[15]	
**Bodyweight (kg)**	**Dose<br>Number of 3 mg tablets**
26 to 44	Two
45 to 64	Three
65 to 84	Four

The doses of moxidectin for this study were selected based on safety data from an initial PK study in man and expected therapeutic blood levels based on those of ivermectin, a similar compound. The starting dose (2 mg or approximately 33 µg/kg for a 60 kg subject) is similar to the minimal effective dose of ivermectin (30 µg/kg).

17.2 Packaging and Labeling

The moxidectin tablets used in this study contains the following ingredients:

Component	% w/w
Moxidectin solid	2.00
Microcrystalline Cellulose	44.625
Lactose, Anhydrous	44.625
Croscarmellose Sodium	5.00
Sodium Lauryl Sulfate	3.00
Colloidal Silicon Dioxide	0.25
Magnesium Stearate	0.5

The placebo contains all ingredients listed above except the moxidectin solid.

Each ivermectin tablet contains 3 mg of active drug. In addition, each tablet contains the following inactive ingredients: microcrystalline cellulose, pregelatinized starch, magnesium stearate, butylated hydroxyanisole, and citric acid powder (anhydrous).

For blinding purposes each 2 mg moxidectin tablet and each 3 mg ivermectin tablet was inserted into an HPMC capsule filled with a mixture of inert ingredients (lactose, microcrystalline cellulose, magnesium stearate and sodium starch glycolate). Detailed instructions for the unblinded designated individual who prepares the drug are provided in Section 17.4 and Attachment 4.

The three capsules used in this study contain the following ingredients:

Component	Moxidectin	Placebo	Ivermectin
	% w/w		
Moxidectin 2 mg Tablet	1 Tablet	0	0
Ivermectin 3 mg Tablet	0	0	1 Tablet
Microcrystalline Cellulose	40	96.5	40
Lactose, Anhydrous	59	0	59
Sodium Starch Glycolate	0.5	0	0.5
Magnesium Stearate	0.5	0.5	0.5
Croscarmellose Sodium	0	3.0	0
Capsule #0 HPMC Opaque (Brown)	1 capsule	1 capsule	1 capsule

17.3 Storage and Stability

Moxidectin 2 mg TICs and Ivermectin 3 mg TICs as well as the placebo capsules should be stored at room temperature up to 30°C with protection from light.

17.4 Preparation

The drug will be prepared by an unblinded pharmacist on the day before dosing according to Table 17.4A and Attachment 4. The prepared drug, identified for each subject by subject name, initials, age, sex, weight, subject number and randomization number, will then be provided to the clinical study staff who will administer the drug.

Table 17.4.A: STUDY DRUG PREPARATION

Cohort	2 mg						4 mg						8 mg					
Treatment Assignment	Moxidectin			Ivermectin			Moxidectin			Ivermectin			Moxidectin			Ivermectin		
Capsule[a]	M	P	I	M	P	I	M	P	I	M	P	I	M	P	I	M	P	I
Weight	Number of Capsules																	
26-44 kg	1	3	0	0	2	2	2	2	0	0	2	2	4	0	0	0	2	2
45-64 kg	1	3	0	0	1	3	2	2	0	0	1	3	4	0	0	0	1	3
65-84 kg	1	3	0	0	0	4	2	2	0	0	0	4	4	0	0	0	0	4

a: M - Capsule containing 2 mg moxidectin tablet, P - Placebo capsule, I - Capsule containing 3 mg ivermectin tablet

17.5 Test Article Accountability, Reconciliation, and Return

The European Medicines Evaluation Agency (EMEA), and Special Program for Research and Training in Tropical Diseases (TDR) require accounting for the disposition of all investigational drugs received by each clinical site. Information on drug disposition required by law consists of the date received, date administered, quantity administered, and the subject to whom the drug was administered. Storage conditions must also be documented. The PI is responsible for accounting for all unused test articles and all used test article containers.

Supplies will be shipped to the principal investigator site for the study initiation. The OCRC will use the relevant form to document test article disposition and record storage conditions from initial receipt through the completion of the study. This form will be completed by the unblinded third party pharmacist

and kept locked in a cabinet accessible to no one else. Each time a dose is prepared for a subject, the following information will be recorded by the unblinded third party pharmacist:

- the subject's initials
- the subject number
- randomization number
- the total dose prepared
- the number and type of capsules
- the packaging control number (lot number)from which the different types of capsules were taken
- the initials of the person preparing the dose

At a minimum, drug accounting will be reviewed by an unblinded monitor not involved in subject treatment or dose progression decision after the 30 day safety review of the last cohort to be enrolled has been completed.

At the termination of the study, a final drug accountability review and reconciliation will be completed, any discrepancies will be investigated, and their resolution will be documented. All unused test articles will be returned to Wyeth or shall be properly disposed of at the direction of Wyeth, including all empty containers. The test article accountability, reconciliation, and return procedures also apply to all other test articles (i.e. comparator, placebo) that are required by the protocol and supplied by the sponsors.

17.6 Study Subject Compliance

The study staff will directly observe compliance. Moxidectin and ivermectin will be administered in a single oral dose under medical supervision in the hospital and administration recorded on the source documents and CRF.

17.7 Other Supplies

Wyeth will provide additional clinical supplies such as CRFs, SAE reporting forms, PK specimen tubes and PK specimen labels.

18. MEASURES TO MINIMIZE/AVOID BIAS

18.1 Subject Identification

Subjects will be enrolled by dose and severity of *O. volvulus* infection. The intensity of infection will be based on the mean of the densities at the iliac crests and calves. Each cohort will consist of 16 subjects (12:4 moxidectin:ivermectin). They will receive a single oral dose followed by frequent safety monitoring over a 30-day period. There will be a total of 9 cohorts, based on the dose of moxidectin to be administered and the intensity of infection as follows:

- 2 mg: subjects with > 0 and < 10 microfilariae/mg of skin (16 subjects)
- 2 mg: subjects with 10-20 microfilariae/mg of skin and the sum of microfilariae in the two eyes must be ≤ 10 (16 subjects)
- 2 mg: subjects with > 20 microfilariae/mg of skin with or without ocular involvement (32 subjects) to be enrolled in 2 consecutive groups of 16 subjects.

- 4 mg: subjects with > 0 and <10 microfilariae/mg of skin (16 subjects)
- 4 mg: subjects with 10-20 microfilariae/mg of skin and the sum of microfilariae in the two eyes must be ≤ 10 (16 subjects)
- 4 mg: subjects with > 20 microfilariae/mg of skin with or without ocular involvement (32 subjects) to be enrolled in 2 consecutive groups of 16 subjects.

- 8 mg: subjects with > 0 and < 10 microfilariae/mg of skin (16 subjects)
- 8 mg: subjects with 10-20 microfilariae/mg of skin and the sum of microfilariae in the two eyes must be ≤ 10 (16 subjects)
- 8 mg: subjects with > 20 microfilariae/mg of skin with or without ocular involvement (32 subjects) to be enrolled in 2 consecutive groups of 16 subjects.

At the time of initiation of screening, each subject is assigned a unique subject number in the order in which they are scheduled for screening. Each subject randomized will keep this subject number for the duration of the study. Subject numbers will not be reassigned or reused for any reason. However, a subject who does not qualify for one cohort based on his/her skin microfilaria count or because the cohort currently being screened for is already complete, but qualifies for another cohort, can be re-screened prior to enrollment of the cohort for which he/she qualifies based on skin microfilaria counts and assigned the next subject number in sequence.

Screening evaluations (history, physical and ocular exam, ECG, lab evaluations, skin snip) will be performed during the 4-day screening period prior to administration of test article on day 1.

All subjects who are screened (including screen failures) will be recorded in an Onchocerciasis Chemotherapy Research Centre (OCRC) screening and enrollment log. Information to be collected includes:

- Date of screening
- Subject number
- Subject initials
- Ethnic origin
- Reason for screen failure (if applicable)

After completing the baseline investigations and qualifying for the study, each subject will be assigned a randomization number by an unblinded third party pharmacist. This number will automatically assign him/her to 1 of 2 treatment regimens (12:4 moxidectin:ivermectin) according to a predetermined schedule (section 18.2, Randomization). Subjects will be dosed according to the schedule in Table 17.4.A and Attachment 4. Treatment will be given after an overnight fast of at least 8 hours on day 1 under medical supervision. Breakfast will be delayed for 2 hours. Smoking, the ingestion of alcoholic beverages, grapefruit, or grapefruit juice, and the use of non-prescribed drugs will not be permitted during the 18-day admission period. Subjects will be encouraged to continue this practice at least until the day 30-31 follow-up and to maintain their normal exercise patterns.

The unblinded third party pharmacist will maintain confidentiality of the randomization number lists and prepare the test articles for administration. The PI will provide the unblinded third party pharmacist with the names, subject numbers, baseline severity and gender of those who are eligible for the trial.

Subjects should be identified to the sponsors only by their assigned numbers, initials, age, and sex. Te investigator must maintain a list of subject names and the identifying information indicated above.

18.2 Randomization

Subjects will be assigned a randomization number by the unblinded third party pharmacist. The unblinded third party pharmacist will be supplied with 12 randomization schedules - one for each of the 3

severity levels (mild, moderate, severe) within each of the 3 dose cohorts (2 mg, 4 mg, 8 mg) and 3 additional schedules for the most severe (> 20 mf/mg of skin) subjects. Each randomization schedule will have 16 unique randomization numbers. The randomization numbers will be assigned by the unblinded third party pharmacist at the time of preparing the study medication and will be recorded on to the case report forms. Treatment groups will be blocked in groups of 4 with a 3 to 1 ratio of moxidectin to ivermectin. When the first male subject is enrolled, the assigned treatment (randomization number) will be selected from the top of the randomization list that corresponds to the subject's assigned stratum. The treatment (randomization number) for the first female subject will be selected from the bottom of the randomization list that corresponds to the subject's assigned stratum. The second male subject will be assigned the next available treatment from the top of the randomization list that corresponds to the subject's assigned stratum. The second female subject will be assigned the next available treatment from the bottom of the randomization list that corresponds to the subject's assigned stratum. Assignment to treatment group will continue in this manner until the entire cohort has been assigned to a treatment group. A new randomization schedule should be used when a new cohort is started.

At the end of the study, the randomization numbers will be used to identify the treatment group assigned to the subject.

18.3 Blinding and Unblinding

The study monitor, the investigator(s), and the subjects at the site will be blinded to treatment assignment. The unblinded third party pharmacist will not participate in the evaluation of any study subject. This will permit all other participants to remain blinded. The unblinded third party pharmacist will prepare and label all treatments with the names and randomization numbers of the subjects based on the randomization list. Individuals who are unaware of the test article random assignment will administer the treatments to the subjects. Contact between the unblinded third party pharmacist and study subjects will be kept to a minimum. The investigator, sub-investigator, research nurse, and any study participants other than the unblinded third party pharmacist will not be allowed to know the treatment assigned to any study subject and will not have access to the randomization treatment records.

The subject's treatment will be unblinded electively only if an AE occurs that the investigator, the Wyeth medical monitor and the WHO clinical coordinator or project manager believe requires identification of the treatment assignment. This may occur in the case of a SAE, as the investigator's decision to enroll the next cohort within the same dose group may be influenced by whether a SAE occurred in a subject or

subjects who received moxidectin or in one who received ivermectin. This will also affect the sponsors' agreement with the investigator's decision. In this case, the unblinded third party pharmacist will inform the investigator, who will inform the Wyeth medical monitor of the subject's treatment assignment and the reason(s) for breaking the randomization code.

Routine unblinding of SAEs will not be performed in this protocol. Ivermectin and moxidectin are macrocyclic lactones and are expected to have similar adverse event profiles. This is a single-dose study and thus there will be no question regarding continuation of therapy after an SAE. The treatment of SAEs will not be affected by whether the subject receives ivermectin or moxidectin.

Emergency unblinding will be at the discretion of the Principal Investigator. The investigator must submit a written explanation describing the event to the sponsors within 5 working days. All randomization envelopes must be returned to the sponsors at the completion of the trial.

19. SAFETY

19.1 Safety Variables

All subjects enrolled in this study will be closely monitored for safety and evaluated for any AEs. Safety evaluation will include: symptoms, physical examinations, vital signs measurement, ECGs, ocular exams, and laboratory evaluations. All evaluations will be monitored as outlined in section 16.1, Procedures, and section 16.3, Special Methods. A key safety component of the study drug will be determined by the presence and severity of the Mazzotti reaction. An important consideration in the administration of microfilaricides to *O. volvulus* infected subjects is the potential for the combination of AEs due to the pharmacologic properties of the drug with the constellation of symptoms, signs and laboratory events that occur when microfilariae are killed (the Mazzotti reaction).[16] These need to be differentiated one from the other and from any coincidental illnesses that may occur in temporal relationship with test drug administration. Common systemic clinical manifestations of the Mazzotti reaction include pruritus, rash, lymphadenitis, headache, myalgia, arthralgia, hypotension, fever and swellings of the face and limbs. Ocular events include epiphora, photophobia, conjunctival injection, limbitis, anterior uveitis, chorioretinitis and optic neuritis.

The clinical laboratory changes involve the peripheral blood leucocytes, aspartate aminotransferase (AST/SGOT) and alanine aminotransferase (ALT/SGPT) and sometimes lactate dehydrogenase (LDH) and gamma-glutamyl-transferase (GGT); microfilariae also appear in blood, urine, and other body fluids. The eosinophils exhibit the most prominent changes. There may be an initial eosinopenia followed several days later by a marked increase above pretreatment levels. A complete disappearance of eosinophils from the peripheral blood is the laboratory hallmark of a severe reaction. Lymphocyte counts may fall initially, followed by lymphocytosis, but not to the same extent as with eosinophils. Leucocytosis with neutrophilia is less common. Elevations in liver enzymes occur, but rarely exceed grade 2 and usually normalize by day 30. Levels of bilirubin and alkaline phosphatase are usually unchanged. Any deviation from this pattern of liver function abnormalities should prompt investigations for an intrinsic drug effect or a coincidental illness. Proteinuria may occur. Although the laboratory changes per se have little clinical significance, they give indirect evidence of the death of microfilariae and may even indicate the speed and severity of the reaction to the event. [17]

The factors that govern the Mazzotti reaction include the intensity of infection,[18] the dose regimen, and the microfilaricide used. These determine the onset, evolution, reaction severity, extent of the laboratory changes, and whether the reaction is mono- or biphasic. A notable exception occurs in subjects with hyper-reactive onchodermatitis (Sowda),[19] where severe, predominantly cutaneous adverse effects occur even with very low skin microfilarial counts.

For a given intensity of infection, the severity of the Mazzotti reaction to ivermectin is independent of dose within the range of 150 to 800 µg/kg (approximately 9 mg-48 mg).[20] Dangerous and alarming reactions result from the simultaneous occurrence of severe reactions in multiple systems.[21] This phenomenon is rare with ivermectin. Biphasic reactions, characterized by a marked recrudescence of cutaneous or lymph node symptoms or the development of an acute febrile polyarthritis, [22]several days after the initial reaction, have also not been observed with ivermectin. There is no reason to believe that the Mazzotti response to moxidectin would differ radically from those due to ivermectin. However, a more rapid elimination of microfilariae may result in an earlier onset, or a more severe reaction, for a given initial infection intensity.

Severe reactions can usually be aborted by the administration of 100 to 200 mg of hydrocortisone hemisuccinate intravenously aided by supportive therapy.

The clinical events will be graded according to the National Cancer Institute common toxicity table, version 2.0, with additions specific for this protocol summarized in Attachments 5 and 6.

More frequent safety evaluations may be made if clinically indicated or at the discretion of the investigator. All adverse effects (AEs) will be recorded in the CRFs throughout the entire study.

19.2 Safety Assessment Methods and Dose Progression Decision

The decision to move to the next higher dose group, will be made based on blinded review of the safety and microfilaricidal data obtained during the first 30 days post treatment of all previously enrolled cohorts.

The PI will submit a recommendation to proceed or not to proceed after a detailed examination of the blinded data. The Wyeth medical monitor, the WHO clinical monitor and the WHO Project Manager will conduct their separate reviews. Wyeth will include a representative of its safety surveillance group in addition to the medical monitor for purposes of review. WHO may include its Clinical Coordinator or another physician in the review.

The PRT will discuss the blinded data and the recommendation to progress or not. The recommendation will be provided together with the rationale for this recommendation to CERT who have access to the randomized dose assignments and have reviewed the data in parallel with the PRT. If there is a difference of opinion within the PRT regarding the dose progression recommendation, the different recommendations and their rationale will be provided to the CERT. The CERT will provide their recommendation to the PRT.

The experts will have access to the treatment codes and will review the data initially in a blinded fashion with the option to unblind at a patient or cohort level, with special focus on the following:

- The number of possibly, probably or definitively drug related non-CNS AEs ≥ grade 2 among moxidectin treated subjects.
- The number of subjects with possibly, probably or definitively drug related CNS AEs >grade 1 that are not related to the efficacy of moxidectin (i.e. not characterized by the PI as Mazzotti reactions) among moxidectin treated subjects.

- Any moxidectin treated subject with a possibly, probably or definitively drug related SAE not related to the efficacy of moxidectin (i.e. not characterized by the PI as Mazzotti reactions).

The review will take the clinical significance of the AEs in the context of the control programs into consideration.

If there is not unanimous agreement among the CERT, they will provide their differing recommendations and their rationale. If there is not unanimous agreement on proceeding to the next dosing cohort between the PRT and a majority of the CERT, the data will be discussed in a blinded fashion. If no agreement can be reached, unblinded data will be made available to the PRT for the subject(s) in question and discussed. If upon discussion of the unblinded data there is no unanimous agreement to proceed to the next higher dose group, dose progression will not occur. A more detailed description of the procedures in the dose progression monitoring plan will be reviewed and approved by the PRT and the CERT.

19.3 Safety Laboratory Determinations

All clinical and laboratory evaluations that deviate from baseline values after study drug administration and are considered to be clinically significant will be repeated and followed until normalization. If normalization does not occur within 2 weeks or by day 30, the etiology will be identified, if possible, and the Wyeth medical monitor, the WHO clinical monitor, and the WHO project manager, will be notified.

20. EFFICACY

20.1 Efficacy Variables

Efficacy against the microfilariae will be determined by reductions from initial counts in skin and ocular microfilariae (where applicable).

20.1.1 Primary

This study will give initial indications of the efficacy of moxidectin against the microfilariae of *O. volvulus*. The primary efficacy variable will be:

- The change from baseline in the mean number of skin microfilariae per milligram of skin sampled at 4 body locations (right and left iliac crests, right and left calves) at 18 months after test article administration

20.1.2 Secondary

Secondary efficacy variables will include:

- The mean reduction from baseline, as well as the percentage of subjects with undetectable microfilariae, will be calculated by dose and severity of infection for each post treatment evaluation time point.
- The change from baseline in the mean number of skin microfilariae per milligram of skin sampled at 4 body locations (right and left iliac crests, right and left calves) at 12 months after test article administration.
- The change from baseline in the mean number of skin microfilariae per milligram of skin sampled at day 8, 1 month, 2 months, 3 months, and 6 months.
- The change from trough values to 12 months and 18 months post treatment in the mean number of skin microfilariae per milligram of skin sampled at 4 body locations (right and left iliac crests, right and left calves) to quantify the effect on the reproductive capacity of adult worms.
- The area under the curve (calculated by using the trapezoidal method through the 18 month visit)
- Ocular examination (time of maximum reduction of microfilariae and number (%) of subjects with complete clearance). The change from baseline in the mean number of ocular microfilariae measured in the anterior chamber. Living and dead microfilariae in the cornea and punctate opacities will also be counted. Retinal changes secondary to the disease will also be examined. Ocular examinations will be performed at baseline and on days 3 or 4, 7 or 8, 14 or 15, and at 1 month, 3 months, 6 months, 12 months and 18 months.
- Evidence of an effect on the viability and/or fertility of the macrofilaria will be obtained from the histologic examination of nodules excised at 18 months. Parameters to be evaluated include:
  a. Viability (number live, moribund, dead)
  b. Calcification (presence/absence)
  c. Reproductive status:
     i. Females:

Proportion:
1. Number producing embryos up to the microfilarial stage
2. Degenerated microfilariae in uterus
3. Relict embryos

ii. Males:
Proportion with:
1. Spermatogenesis normal or abnormal

iii. Other criteria:
Proportion of nodules with:
1. Microfilariae in the capsule
2. No male worms

20.2 Efficacy Assessment Methods

- The maximal microfilaricidal effect on skin microfilariae will be determined by skin snips taken at day 8 and 1 month (± 1 week).
- Nodules will be processed at 18 months for histological examination and assessed by the OCRC parasitology department (blinded to the treatment).

21. PHARMACOKINETIC ANALYSIS

Blood samples for determination of moxidectin plasma concentrations will be collected within 2 hours prior to dosing (0 hr), and at 1, 2, 4, 8, 24, and 72 hours after study drug administration, on study days 8, 13, and 18 and on months 1 (± 1 week), 2 (± 1 week), 3 (± 1 week), 6 (± 1 month) and 12 (± 1 month). PK samples will be drawn from all subjects in all cohorts with the exception of the 2nd group of 16 subjects with > 20 microfilariae/mg of skin in cohorts 3, 6, and 9. Samples from subjects will be analyzed by a validated bioanalytical method.

A PK analysis will be performed using model independent methods to characterize the PK parameters of moxidectin, such as maximum plasma concentration (C_{max}), time to reach the maximum plasma concentration (t_{max}), area under the plasma concentration vs. time curve (AUC) and the apparent terminal elimination half-life ($t_{1/2}$).

22. STATISTICAL ANALYSIS

22.1 Statistical Methods

This is a randomized, double-blind, active-control, dose-escalation trial comparing 3 doses of moxidectin (2, 4, and 8 mg) with ivermectin, stratified by microfilariae density (mild, moderate, severe) and gender. Subjects will be treated as in subjects for the first 18 days and as outpatients for the remainder of the 18 month study.

The primary analysis population for safety will be the modified intent-to-treat (mITT) population, which consists of all subjects who receive study medication. For efficacy, the primary analysis population will be the evaluable subject population, which is defined as all subjects who receive study medication and have baseline and 18-month microfilariae count data. An mITT sensitivity analysis will also be performed to evaluate the effect on the efficacy results of dropouts occurring before 18 months.

The primary objective of the study is to evaluate the safety of escalating moxidectin regimens. Both the incidence of clinical AEs and clinically significant laboratory test results will be used to access safety. The primary efficacy endpoint is the reduction from baseline in skin microfilariae at 18 months. Microfilariae count will be defined as the geometric mean of skin microfilariae/mg taken at 4 sites (iliac crests and calves).

In addition, important secondary endpoints are the reduction from baseline in skin microfilariae load at day 8, 1 month, 2 months, 3 months, 6 months and 12 months, area under the curve (calculated by using the trapezoidal method through the 18 month visit), ocular examination (time of maximum reduction of microfilariae and number (%) of subjects with complete clearance) and nodulectomy (viability and fertility of macrofilariae) at 18 months.

22.2 Methodology

Initially, an overall among-group test will be performed to determine if any significant differences exist among the 4 treatment groups (2 mg, 4 mg, 8 mg moxidectin, and ivermectin). For this analysis the ivermectin control will be pooled over all nine cohorts (three dose groups). Following a significant overall result, paired comparisons (i.e., each moxidectin group versus ivermectin) will be performed to characterize the results. Prior to pooling the nine cohorts the ivermectin response for the nine cohorts will

be graphically evaluated to ensure that there is no time effect on the pooled ivermectin results. For safety, moxidectin will be compared with ivermectin with respect to the incidence of AEs by using the Fisher's exact test. Laboratory data will be analyzed by using the analysis of covariance, adjusting for baseline. For efficacy, the microfilariae counts will be logarithmically transformed (Mosteller and Tukey) and analyzed by using the analysis of covariance, adjusting for baseline counts and the number of sites from which the microfilariae were taken. The latter covariate will be used to adjust for subjects who have fewer than 4 microfilarial collection sites. Factors in the model will include treatment group, covariates, level of microfilariae density (mild, moderate, severe), and gender. Also, a sensitivity analysis will be performed to examine the effect on results of second-order interactions. Data results will be summarized as adjusted geometric means. If the assumptions of normality or log-normality are not met, the data will be analyzed by using the Friedman Test, blocking on baseline skin microfilariae density and gender. In order to evaluate the robustness of the results, the microfilariae data will also be analyzed as a percent change from baseline. The proportion of subjects cured (i.e. with undetectable microfilariae) will also be evaluated.

For the analysis of microfilariae viability and fertility endpoints, proportions will be analyzed by using the Cochran-Mantel-Haenszel procedure blocking on baseline skin microfilariae density. If the overall test is significant, paired comparisons will be made comparing each moxidectin group with ivermectin.

For ocular microfilariae, time to maximum reduction will be analyzed by using the Friedman Test blocking on baseline skin microfilariae density. The proportion of subjects with complete clearance will be analyzed by the Cochran-Mantel-Haenszel procedure blocking on baseline skin microfilariae density. If the overall test is significant, paired comparisons will be made comparing each moxidectin group with ivermectin.

All tests will be performed as 2-sided tests with $\alpha = 0.05$.

22.3 Sample Size and Power

For the mild and moderate severity levels in each dose group, 16 subjects will be randomly assigned in a 3:1 ratio to either moxidectin or ivermectin, for the severe level in each dose group 32 subjects will be randomly assigned to treatment using the same ratio. The probability of detecting at least 1 AE of NCI grade 2 or higher among 12 moxidectin-treated subjects will be 0.114 and 0.718 when the true rates are

1% and 10%, respectively. The probability of detecting at least 1 AE of NCI grade 2 or higher among 24 moxidectin-treated subjects will be 0.214 and 0.92 when the true rates are 1% and 10%, respectively.

For efficacy, given that the subjects in the three severity levels within a moxidectin dose group can be pooled and that all ivermectin subjects can be pooled, there will be 48 subjects per moxidectin dose group and 48 subjects in the pooled ivermectin treatment group. With 48 subjects per treatment group there is 78.3% power to detect a statistically significant difference between a response (% of baseline microfilariae) of approximately 0.1% (moxidectin) and 20% (ivermectin) at 18 months after treatment.

23. CONTRAINDICATIONS, PRECAUTIONS, AND WARNINGS

Safety studies have been completed in mice, rats, dogs, sheep, cattle, horses, and humans. At toxic dose levels, events such as decreased activity, prostration, tremors, chromodacryorrhea, decreased respiration, diarrhea, hypersensitivity to touch and sound, and epistaxis were observed. However, the NTELs in all of the animal models were high multiples of the anticipated human dosage on the basis of body weight. Further information is given in the Moxidectin Investigator's Brochure provided. The data from the first study in man, a single ascending dose study in uninfected subjects followed for 80 days after dose administration show that moxidectin is safe and well tolerated at single doses between 3 mg and 36 mg. No SAEs and no grade 4 AEs were reported. Only 1 AE higher than grade 2 was reported in this study (enteritis due to food poisoning [grade 3] in the 36 mg fasting group) and was considered unrelated to study drug. The most common AEs were headache (35%) and infection (29%). Only half of the headaches reported and none of the infections reported were regarded as study drug related. The reported infections, including dose group, infection type and days after moxidectin administration, were: 3 mg fasted – upper respiratory tract infection (URTI) on Day 19 and Day 63 (same subject), viral URTI on Day 14; 9 mg fasted – toe infection on Day 52; 9 mg fed – URTI on Day 30 and Day 78 (different subjects); 36 mg fasted – tooth abscess on Day 47, head cold on Day 50 and Day 66 (different subjects); 36 mg fed – cold on Day 42 and cold-like symptoms on Day 59 (same subject). Four (4) subjects discontinued from the study (1 before receiving study drug and 3 after dose administration [in the 3 mg fasting, 9 mg fed, and 36 mg fasting groups]). The withdrawal from the study for these subjects was unrelated to AEs.

In a bioavailability study comparing the tablet and liquid forms of moxidectin (protocol 3110A1-101-EU) 58 healthy male subjects received a single dose of 10 mg of moxidectin either as the liquid formulation

(29 subjects) used in the FIM study or as tablets (29 subjects) and were followed up for 180 days. During the course of this study there were no SAEs and no subjects discontinued the study due to an AE. A total of 36 (62.1%) subjects had treatment emergent AEs (TEAEs) during the study, with the same number and percentage of subjects (18; 62.1%) reporting TEAEs in both the moxidectin liquid and tablet groups. During the first 7 days post treatment, 10 subjects (34.5%) receiving liquid moxidectin and 9 subjects (31.0%) receiving tablet reported AEs, including asthenia (10.3% in the tablet group), headache (13.8% and 6.9% in the liquid and tablet groups, respectively), infection (6.9% in the liquid group), diarrhea (6.9% in the liquid group), myalgia (6.9% in the tablet group), and dizziness (6.9% in the tablet group). Between day 8 and 180 of follow up, 10 subjects (34.5%) receiving liquid and 12 subjects (41.4%) receiving tablet reported AEs. The most commonly reported events were flu syndrome (17.2% and 20.7% in liquid and tablet groups, respectively), headache (6.9% in the tablet group), and infection (6.9% in the tablet group). All of the TEAEs were mild to moderate in intensity, and none were considered to be related to treatment. No clinically relevant abnormalities were observed in vital sign measurements, ECGs, or laboratory tests during the study.

For a complete description of all (AEs) reported during the Phase 1 clinical studies with moxidectin, please refer to the most recent moxidectin Investigator's Brochure provided.

24. ADVERSE EVENTS

24.1 Definitions

The term "adverse event," as used by the sponsors, is synonymous with the term "adverse experience," which is used by the FDA.

An ***adverse event*** is any untoward, undesired, unplanned clinical event in the form of signs, symptoms, disease, or laboratory or physiological observations occurring in a human being participating in a clinical study with a sponsor's test article, regardless of causal relationship. This includes the following:

- Any clinically significant worsening of a preexisting condition.
- An AE occurring from overdose of a sponsor test article whether accidental or intentional (ie, a dose higher than that prescribed by a health care professional for clinical reasons).
- An AE occurring from abuse of a sponsor's test article (ie, use for nonclinical reasons).
- An AE that has been associated with the discontinuation of the use of a sponsor's test article.

Note: A procedure is not an AE, but the reason for a procedure may be an AE.

A ***preexisting condition*** is a clinical condition (including a condition being treated and AEs occurring between ICF signing to the subject's arrival at the clinic) that is diagnosed or occurs before the screening of the subject for this study is initiated in the hospital. A preexisting condition can be part of the subject's medical history or part of baseline conditions and are recorded in the source records and on the appropriate page of the CRF (for randomized subjects).

Baseline conditions are all those clinical conditions, including those related to infection with Onchocerca volvulus, present at the time of screening. Medical history are all clinical conditions known to have been present prior to screening. A condition can be both part of the medical history and baseline conditions if the condition started prior to screening and is still present at screening (e.g. any condition related to infection with Onchocerca volvulus).

Adverse events occurring during and due to screening procedures will be recorded as adverse events.

AE collection on source documents starts on the date of subject's arrival at the clinic / start of screening. From this time point, any SAEs are reported to Wyeth within 1 business day from their occurrence.

The questions concerning whether the condition existed before the start of the active phase (i.e. treatment) of the study and whether it has increased in severity and/or frequency will be used to determine whether an event is a treatment-emergent AE (TEAE). An AE is considered to be treatment emergent if (1) it was not present when the active phase of the study began and is not a chronic condition that is part of the subject's medical history, or (2) it was present at the start of the active phase of the study or as part of the subject's medical history, but the severity or frequency increased during the active phase.

The active phase of the study begins at the time of the administration of the test article and ends at the 18 month visit.

A ***serious adverse event*** (SAE) is any AE occurring at any dose that meets 1 or more of the following criteria:

- Results in death
- Is life threatening (see below)

- Requires inpatient hospitalization or prolongation of an existing hospitalization (see below)
- Results in a persistent or significant disability or incapacity (see below)
- Results in cancer
- Results in a congenital anomaly or birth defect

Additionally, important medical events that may not result in death, be life threatening, or require hospitalization may be considered SAEs when, based on appropriate medical judgment, they may jeopardize the subject and may require medical or surgical intervention to prevent one of the outcomes listed above. Examples of such events include allergic bronchospasm requiring intensive treatment in an emergency room or at home, blood dyscrasias or convulsions that do not require hospitalization, or development of drug dependency or drug abuse.

A ***life threatening adverse event*** is any AE that places the subject at immediate risk of death from the event as it occurred. A life-threatening event does not include an event that might have caused death had it occurred in a more severe form but that did not create an immediate risk of death as it actually occurred. For example, drug-induced hepatitis that resolved without evidence of hepatic failure would not be considered life threatening, even though drug-induced hepatitis of a more severe nature can be fatal.

Hospitalization is to be considered only as an overnight admission. Hospitalization or prolongation of a hospitalization is a criterion for considering an AE to be serious. In the absence of an AE, the participating investigator should not report hospitalization or prolongation of hospitalization on a Serious Adverse Event form (a 7443 form). This is the case in the following situations:

- Hospitalization or prolongation of hospitalization is needed for a procedure required by the protocol. Day or night survey visits for biopsy or surgery required by the protocol are not considered serious.

- Hospitalization or prolongation of hospitalization is part of a routine procedure followed by the study center (eg, stent removal after surgery). This should be recorded in the study file.

- Hospitalization for survey visits or annual physicals fall in the same category.

In addition, a hospitalization planned before the start of the study for a preexisting condition that has not worsened does not constitute an SAE (eg, elective hospitalization for a total knee replacement due to a preexisting condition of osteoarthritis of the knee that has not worsened during the study).

Disability is defined as a substantial disruption in a person's ability to conduct normal life functions.

If there is any doubt whether the information constitutes an AE or SAE, the information is treated as an AE or SAE.

24.2 Timing for Reporting Serious Adverse Events

Any SAE, regardless of causal relationship, must be reported to the Wyeth medical monitor, the WHO clinical monitor, the WHO project manager and the Coordinator of the Ghana National Center for Pharmacovigilance (GNCP) (or their designees) immediately (no later than 24 hours after the investigator becomes aware of the SAE) by faxing a completed serious adverse event form (form 7443) to the numbers indicated in the front of this protocol and then confirming by telephone that the faxes were received. Compliance with this time requirement is essential so that the sponsors may comply with their regulatory obligations.

Follow-up information relating to an SAE must be reported to the Wyeth medical monitor, the WHO clinical monitor, the WHO project manager and the Coordinator of the Ghana National Center for Pharmacovigilance (or their affiliates or designees) within 24 hours of receipt by the investigator by faxing a completed serious adverse event form (form 7443) to the numbers indicated in the front of this protocol and confirming by telephone that the faxes were received. The subject should be observed and monitored carefully until the condition resolves or stabilizes or its cause is identified.

Any emergency must be reported to the Wyeth medical monitor, the WHO clinical monitor, the WHO project manager and the Coordinator of the Ghana National Center for Pharmacovigilance (or their affiliates or designees) immediately (within 24 hours).

For all other inquiries and information about this study, contact a clinical scientist listed in the front of this protocol.

24.3 Reportable Events/Information

- An AE or SAE can occur from the time that the subject begins the hospital screening period to the completion of the final study visit at month 18; regardless of test article or protocol relationship. All AEs and SAEs will be recorded on source documents. All AEs and SAEs for subjects who are not screen failures will be recorded in the CRFs. The investigator must follow up as is medically necessary on all AEs, SAEs, and other reportable events until the event has subsided or values have returned to baseline, or in case of permanent impairment, until the condition stabilizes.

  For SAEs: The investigator will provide all documentation pertaining to the event (eg, additional laboratory tests, consultation reports, discharge summaries, postmortem reports, etc) to the Wyeth medical monitor, the WHO clinical monitor, the WHO project manager and the Coordinator of the Ghana National Center for Pharmacovigilance (or their affiliates or designees) in a timely manner. Reports relative to the subject's subsequent course must be submitted to the Wyeth medical monitor, the WHO clinical monitor, the WHO project manager and the Coordinator of the Ghana National Center for Pharmacovigilance (or their affiliates or designees) until the event has subsided or, in case of permanent impairment, until the condition stabilizes.

- The following events will be recorded and reported in the same time frame and following the same process as for SAEs:

  1. All pregnancies that occur during the study and their outcome. If a pregnancy is confirmed during the study, the Wyeth medical monitor, the WHO clinical monitor, the WHO project manager and the Coordinator of the Ghana National Center for Pharmacovigilance (or their affiliates or designees) should be notified immediately. When possible, all reports of pregnancy must be followed up for information about the course of the pregnancy and delivery, as well as the condition of the newborn. When the newborn is healthy, additional follow-up is not needed. The investigator will provide follow-up information concerning the outcome of the pregnancy to the sponsors and the Coordinator of the GNCP in a timely manner. This information will be provided regardless of whether the subject has discontinued participation in the study.

2. Test article abuse and overdose (ie, use for nonclinical reasons) with or without AEs. An overdose is a dose higher than that prescribed by a health care professional for clinical reasons. It is up to the participating investigator to decide whether a dose was an overdose.

3. Inadvertent or accidental exposure to test article with or without an AE.

   - Poststudy test article-related SAEs.
   - SAEs occurring after unauthorized or accidental use in persons not participating in the study.
   - Abnormal biological or vital signs values that are considered clinically relevant by the participating investigator. These must be reported in the same time frame and following the same process as for an AE or an SAE.

24.4 Recording and Reporting

At each required study visit, all AEs that have occurred since the previous visit must be recorded in the adverse event record of the subject's CRF. The information recorded should be based on the signs and symptoms detected during the physical examination and clinical evaluation of the subject. In addition to the information obtained from those sources, the subject should be asked the following nonspecific question: "How have you been feeling since your last visit?" Signs and symptoms should be recorded using standard medical terminology.

The following AE information must be included (when applicable): the specific condition or event and direction of change; whether the condition was preexisting (ie, an acute condition present at the start of the study or history of a chronic condition) and, if so, whether it has worsened (eg, in severity and/or frequency); the dates and times of occurrence; severity; causal relationship to test article; action taken; and outcome. The causal relation between an AE and the test article will be determined by the investigator on the basis of his or her clinical judgment and the following definitions:

- ***Definitely related***: Event can be fully explained by administration of the test article.
- ***Probably related***: Event is most likely to be explained by administration of the test article rather than the subject's clinical state or other agents/therapies.
- ***Possibly related***: Event may be explained by administration of the test article or by the subject's clinical state or other agents/therapies.
- ***Probably not related***: Event is most likely to be explained by the subject's clinical state or other agents/therapies, rather than the test article.

- ***Definitely not related***: Event can be fully explained by the subject's clinical state or other agents/therapies.

When assessing the relationship between administration of a test article and an AE, the following should be considered:

- Temporal relationship between administration of the test article and the AE
- Biological plausibility of relationship
- Subject's underlying clinical state or concomitant agents and/or therapies
- When applicable, whether the AE abates on discontinuation of the test article (dechallenge)
- When applicable, whether the AE reappears on repeat exposure to the test article (rechallenge)

SAEs that are not test article related may nevertheless be considered by the participating investigator or the medical monitor (or designee) to be related to the conduct of the clinical study, ie, to a subject's participation in the study. For example, a protocol-related SAE may be an event that occurs during a washout period or that is related to a procedure required by the protocol.

The severity of AEs will be assessed according to the National Cancer Institute's common toxicity criteria (CTC), version 2.0, April 30, 1999.[23] Additions to this grading scale for ocular, hematological and biochemical adverse events can be found in Attachments 5 and 6 respectively

25. DATA QUALITY ASSURANCE

The sponsors perform quality control and assurance checks on all clinical studies that they sponsor. Before enrolling any subjects in this study, sponsors' personnel and the investigator review the protocol, the brochure for clinical investigators, the CRFs and instructions for their completion, the procedure for obtaining informed consent, and the procedure for reporting AEs and SAEs. A qualified representative of the sponsors monitors the conduct of the study by visiting the site and by contacting the site by telephone. During the visits, information recorded on the CRFs is verified against source documents. All data on the CRFs will have corresponding source records in patient charts or laboratory records. After the sponsors receive the CRFs, the data are entered into the database by using a double data entry procedure. The sponsors' medical monitor reviews the data for safety information. The sponsor clinical data associates review the data for legibility, completeness, and logical consistency. Additionally, the sponsors clinical data associates use automated validation programs to help identify missing data, selected protocol

violations, out-of-range data, and other data inconsistencies. Requests for data clarification or correction are forwarded to the investigative site for resolution. A sample set of records from the final database will be fully audited against the corresponding CRFs.

26. INVESTIGATORS REGULATORY OBLIGATIONS

26.1 Independent Ethics Committee (GFDB/IEC) Approval

The protocol and the informed consent document must have the initial and at least annual (when required) approval of the GFDB/IEC. The signed GFDB/IEC approval letter must identify the documents approved (ie, list the investigator's name, the protocol number and title, the date of the protocol and informed consent document, and the date of approval of the protocol and the informed consent document). Written information to be provided to the subject (eg, diary cards, instructions for test article administration) and any advertisement used to recruit subjects must also be reviewed by the GFDB/IEC. The sponsors will not ship clinical supplies until a signed approval letter from the GFDB/IEC has been received and a contractual agreement has been signed by the sponsors and the clinical site. Copies of the regulations relating to IECs are available from the sponsors.

26.2 Prestudy Documentation

The investigator must provide the sponsors with the following documents BEFORE enrolling any subjects:

- All applicable country-specific regulatory forms.
- Current signed and dated curricula vitae for the investigator, subinvestigators, and all key personnel listed on the clinical study information form.
- Copy of the GFDB/IEC approval letter for the protocol and informed consent. Written assurance of continuing approval (at least annually) as well as a copy of the annual progress report submitted to the GFDB/IEC must also be provided to the sponsors. Any changes in this study or unanticipated problems involving risks to the subjects must be reported promptly to the GFDB/IEC. An investigator must not make any changes in a study without GFDB/IEC and sponsors approval except when necessary to eliminate apparent immediate hazards to the subjects. All protocol amendments must be submitted to the GFDB/IEC and approved.
- Copy of the GFDB/IEC-approved informed consent document to be used.
- When applicable, a list of the GFDB/IEC members and their qualifications, and a description of the committee’s working procedure.

- Copy of the protocol sign-off page signed by the investigator.
- Fully executed clinical study agreement (CSA).
- A written document containing the name, location, certification number, and date of certification of the laboratory to be used for laboratory assays and those of other facilities conducting tests. This document should be returned along with the statement of investigator form. The sponsors must be notified if the laboratory is changed or if any additional laboratory is to be used.
- List of normal laboratory values and units of measure for all laboratory tests required by the protocol. This is required for each laboratory to be used during the study. The sponsors must be notified if normal values or units of measurement change.

26.3 Informed Consent

Regulatory agencies have issued regulations to provide protection for human subjects in clinical investigations and to describe the general requirements for informed consent.

A copy of your proposed informed consent document should be submitted to the sponsors for review and comment before submission to your IEC. The study should not begin until the document has been reviewed by the sponsors and must not begin until the document has been approved by the IEC. In some instances the study must not begin until the document has been approved by a regulatory agency.

The informed consent document shall contain all of the elements of informed consent specified in the regulations. Some regulations may require the disclosure of additional information to the subject and/or inclusion of additional information in an informed consent document. Copies of the regulations relating to informed consent and the protection of human subjects in clinical studies are available from the sponsors.

Nothing in these regulations is intended to limit the authority of a physician to provide emergency medical care under applicable regulations. In addition, the PI should be aware that some regulations require that he/she permit regulatory agencies to conduct inspections and review records pertaining to this clinical investigation.

The delegation of investigator responsibilities including informed consent will be documented on the clinical study information form.

26.4 Declaration of Helsinki

This study will be conducted in accordance with the ethical principles that have their origin in the Declaration of Helsinki and that are consistent with good clinical practice (GCP) and the applicable regulatory requirements.

26.5 Case Report Forms

1) All data will be recorded on CRFs provided by the sponsors. Black ballpoint pens should be used.
2) The white and yellow originals, or the white originals for North American studies, must be returned to the sponsors; the investigator must retain the pink copy for his/her file. For some studies, the specified copy will be sent to the central lab.
3) CRFs and other pertinent records are to be submitted to the sponsors during and/or at completion or termination of the study. The investigator also must submit all incomplete CRFs that document subject experience with the test article, including retrievable data on subjects who withdraw before completion of the study.

26.6 Adverse Event Reporting

The investigator agrees to report all AEs to the sponsors as described in the Adverse Events section. Furthermore, the investigator is responsible for ensuring that any sub-investigator promptly brings AEs to the attention of the investigator. The investigator is responsible for reporting any AE to the Ghana National Center for Pharmacovigilance (GNCP) and to send copies of all correspondence with the GNCP to Wyeth and the WHO. If applicable, the investigator also is responsible for informing the participating IEC of any SAEs.

26.7 Review of Source Records

The investigator agrees that qualified representatives of the sponsors and regulatory agencies will have the right, both during and after this study, to conduct inspections and to audit and review medical records pertinent to the clinical study as permitted by the regulations. Subjects will not be identified by name, and confidentiality of information in medical records will be preserved. The confidentiality of the subject will be maintained unless disclosure is required by regulations. Accordingly, the following statement (or similar statement) will be included in the informed consent document:

Representatives of regulatory agencies GFDB/IECs, the sponsors, the WHO, the Review Board of the Ghana National Center for Pharmacovigilance, and your personal physician may review your medical records and all information related to this study as permitted by law. Identifying information will not appear on any record received by the sponsors. Your identity will remain confidential unless disclosure is required by law.

26.8 Monitoring of the Study

This study is monitored by a representative from the WHO. Site visits are made before the study begins, at regular intervals during the study, and at the study closeout. Communication by telephone and mail and e-mail may be used as needed to supplement site visits. The investigator and study personnel will cooperate with the sponsors, provide all appropriate documentation, and be available to discuss the study. The purpose of the site visits is to verify the following:

1) Adherence to the protocol. (The investigator should document and explain any deviation from the approved protocol.)
2) The completeness and accuracy of the CRFs and the dispensing and inventory record. (Adequate time and space for these visits should be allocated by the investigator.)
3) Compliance with regulations. The verification will require comparison of the source documents to the CRFs.

26.9 Protocol Amendments

Any significant change in the study protocol will require an amendment. The investigator will outline the reasons and justification for the amendment in line with the TDR SOP for clinical investigators, and will discuss with the WHO clinical monitor. The monitor will append his comments and recommendations to the document that will then be submitted to the Wyeth medical monitor and WHO Project manager. A protocol amendment will be generated and signed by the Wyeth medical monitor and the WHO Clinical Coordinator. A protocol amendment may be implemented at this stage. Where applicable, the amendment must receive a favorable opinion from the GFDB/IEC and WHO ERC.

A protocol change intended to eliminate an apparent immediate hazard to subjects may be implemented immediately, but the change must then be documented in an amendment and submitted to the Ghana Food and Drugs Board (IEC) and WHO ERC within 5 working days. The protocol amendment will be sent in the same time period to the Wyeth medical monitor and the WHO project manager.

26.10 Change in Investigator

If the investigator retires, relocates, or otherwise withdraws from conducting a study, the responsibility for maintaining records may be transferred to the sponsors, GFDB/IEC, or another investigator. The sponsors must be notified of and agree to the change. The Ghana Food and Drugs Board will be notified with the appropriate documentation.

26.11 Termination of the Study

26.11.1 Termination by the Sponsors

Either sponsor may terminate the study at any time for any of the following reasons:

1. Failure to enroll subjects.
2. Protocol violations.
3. Inaccurate or incomplete data.
4. Unsafe or unethical practices.
5. Questionable safety of the test article.
6. Suspected lack of efficacy of the test article.
7. Administrative decision.

26.11.2 Termination by the Investigator

If the investigator terminates the study prematurely, the investigator does the following:

1. Returns all test articles, CRFs, and related study materials to the sponsors.
2. Provides the GFDB and the sponsors with a written statement describing why the study was terminated prematurely. Prompt compliance with this requirement is essential so that the sponsors may comply with its regulatory obligations.

26.12 Final Study Report

The investigator should complete a report notifying the GFDB of the conclusion of the clinical study. This report should be made within 3 months of completion or termination of the study.

The final report sent to the GFDB should also be sent to the sponsors and, along with the completed CRFs, constitutes the final summary to the sponsors, thereby fulfilling the investigator's regulatory responsibility.

26.13 Confidentiality

All unpublished information that the sponsors give to the investigator shall be kept confidential and shall not be published or disclosed to a third party without the prior written consent of the sponsors.

When the sponsors generate reports for presentations to regulatory agencies, the investigator may be asked to endorse the final report. The endorsement is required by some regulatory agencies.

The investigator shall not make a patent application based on the results of this study and shall not assist any third party in making such an application without the written authorization of the sponsors unless otherwise specified in the CSA.

26.14 Records Retention

The investigator shall retain and preserve one copy of all data generated in the course of the study, specifically including but not limited to those documents defined by GCP as essential documents, for the longer of:

i. 2 years after the last marketing authorization for the study drug has been approved or the sponsors have discontinued its research with respect to such drug or
ii. Such longer period as required by applicable global regulatory requirements. At the end of such period, the investigator shall notify in writing the sponsors of its intent to destroy all such material. The sponsors shall have 30 days to respond to the investigator’s notice, and the sponsors shall have a further opportunity to retain such materials at the sponsor’s expense.

26.15 Publications

If on completion of the study the data warrant publication, the investigator may publish the results in recognized (refereed) scientific journals subject to the provisions of the CSA. Unless otherwise specified in the CSA, the following process shall occur:

The principal investigator shall submit reports, abstracts, manuscripts and or other presentation materials to the sponsors for review prior to submission for publication or presentation. The sponsors shall have 60 calendar days to respond with any requested revisions, including without limitation, the deletion of confidential information. The principal investigator shall act in good faith upon such requested revisions, except the principal investigator shall delete any confidential information from such proposed publication. The principal investigator shall delay submission of such publication or presentation materials for up to an additional 90 calendar days in order to have a patent application(s) filed.

26.16 Subject Injury

WYETH shall indemnify WHO, the Principal Investigator and his/her institution, and pay, for the medical expenses of reasonable and necessary medical treatment if a Study patient is injured during the Study and the injury is a result of the effects of the Study Drug, to the extent that the medical expenses are not covered by the Study patient's medical insurance, a government program or any other responsible third party, and provided that the Study patient has reasonably followed the instructions of the Principal Investigator, and the WHO, Principal Investigator and each Other Clinical Investigator involved in administering the Study Drug to the patient or otherwise providing medical care to such patient have reasonably complied with relevant obligations in this Agreement, the Protocol and all applicable laws and regulations.

WHO shall procure and maintain liability insurance with reputable and financially secure insurance carriers to cover its obligations that may arise in connection with any injury to any patient for which, pursuant to the second paragraph of Section 13.1, WYETH shall not be liable.

27. SPONSOR OBLIGATIONS IN CASE OF PREMATURE TRIAL TERMINATION

If the sponsors should jointly decide to end the clinical trial prematurely, they will notify the investigator, the ethics committees and the relevant authorities of this decision and the reasons for the termination.

28. REFERENCES

[1] Taylor HR, Trpis M, Cupp EW, et at. Ivermectin prophylaxis against experimental Onchocerca volvulus infection in chimpanzees. Am J Trop Med Hyg. 1988;39(1):86-90.

[2] Chavasse DC, Post RJ, Davies, JB, Whitworth JA. Absence of sperm from the seminal receptacle of female Onchocerca volvulus following multiple doses of ivermectin. Trop Med Parasitol. 1993;44(3):155-158.

[3] Duke BO, Zea-Flores G, Castro J, Cupp E, Munoz B. Effects of multiple monthly doses of ivermectin on adult Onchocerca volvulus. Am J Trop Med Hyg. 1990;43(6):657-664.

[4] Fisher MH. Structure-activity relationships of the avermectins and milbemycins. American Chemical Society Symposium Series, Phytochemicals for Pest Control. 1997;17:220-238.

[5] Ingle D, Wood IB. Princeton, NJ, Wyeth (Fort Dodge) Report, 1990.

[6] Sangster NC, Bannan SC, Weiss AS, Nulf SC. Haemonchus contortus: Sequence heterogeneity of internucleotide binding domains from P-glycoproteins and an association with avermectin/milbemycin resistance. Exp Parasitol. 1999; 91:250-257.

[7] Besier RB, Lyon J, Kieran PJ. The effect of moxidectin against benzimidazole- and levamisole-resistant nematodes of sheep in Western Australia. Aust Vet J.1993;70(11):422-423.

[8] Oosthuizen WTJ, Erasmus JB. Efficacy of moxidectin against a strain of Haemonchus contortus resistant to ivermectin, a benzimidazole and a salicylanilide. J S Afr Vet Assoc. 1993;64(1):9-12.

[9] Cole LM, Casida JE. GABA-gated chloride channel: Binding site for 4-ethynyl-4-n-[2,3-3H2] propylbicyclo-ortho benzoate ([3H]EBOB) in vertebrate brain and insect head. Pesticide Biochemistry and Physiology. 1992;44: 1-8.

[10] Townson S. TDR project 960089 progress report, 23 December 1998.

[11] Tagboto SK, Townson S. Onchocerca volvulus and O. lienalis: The microfilaricidal activity of moxidectin compared with that of ivermectin in vitro and in vivo. Ann Trop Med Parasitol. 1996;90(5):497-505.

[12] Trees A. TDR project 980383 progress report, January 2000.

[13] Anonymous. TDR progress report project 980386 & 980389, January 2000.

[14] McCall JW, Dzimianski MT, Supakomdej P, Jun JJ. The antifilarial activity of moxidectin against patent infections of Brugia Pahangi in dogs. Abstract 707. Am J Trop MedHyg. (1999);61 (Suppl 444).

[15] MECTIZAN Package Insert, 5 July 1999.

[16] Awadzi, K, Dadzie KY, De Sole G, Remme J. Reactions to ivermectin treatment in onchocerciasis patients. Acta Leiden. 1990;59: 93-199.

[17] Awadzi K: The chemotherapy of onchocerciasis II. Quantitation of the clinical reaction to microfilaricides. Ann Trop Med Parasitol. 1980;74:189-197.

[18] Francis H, Awadzi K, Ottesen EA. The Mazzotti reaction following treatment of onchocerciasis with diethylcarbamazine: clinical severity as a function of infection intensity. Am J Trop Med Hyg. 1985;34:529-536.

[19] Baraka OZ, Mahmoud BM, Ali MM, Ali MH, el Sheikh EA, Homeida MM, Mackenzie CD, Williams JF. Ivermectin treatment in severe asymmetric reactive onchodermatitis (sowda) in Sudan. Trans R Soc Trop Med Hyg. 1995;89:312-315.

[20] Awadzi K, Opoku NO, Addy ET, Quartey BT: The chemotherapy of onchocerciasis XIX: the clinical and laboratory tolerance of high dose ivermectin. Trop Med Parasitol. 1995;46:131-137.

[21] Bryceson AD, Warrell DA, Pope HM: Dangerous reactions to treatment of onchocerciasis with diethylcarbamazine. Br Med J. 1977;1:742-744.

[22] Awadzi K, Gilles HM: Diethylcarbamazine in the treatment of patients with onchocerciasis. Br J Clin Pharmacol. 1992;34: 281-288.

[23] Cancer Therapy Evaluation Program; Common Toxicity Criteria, Version 2.0, DCTD, NCI, DHHS, April 1999.

ATTACHMENTS

ATTACHMENT 1. RESPONSIBILITIES OF THE UNBLINDED THIRD PARTY PHARMACIST

1. The unblinded third party pharmacist signs a statement (Wyeth form 6732C) that assures Wyeth Research and the regulatory authorities that he or she understands his or her obligations and responsibilities as specified in the protocol, ie, correct preparation of the study medication and the manner in which it is to be administered. The original signed statement is forwarded to the Wyeth Research clinical scientist. Copies of this statement are retained by the unblinded third party pharmacist and the investigator.

2. The unblinded third party pharmacist is responsible for all clinical materials provided by Wyeth Research, such as the study medication, randomization chart, drug accountability and dispensing forms, and any other supplies.

3. The unblinded third party pharmacist will be responsible for maintaining the confidentiality and security of the randomization chart.

4. The unblinded third party pharmacist will

    a. Identify the correct treatment for the subject based on the randomization chart generated by Wyeth Research and prepare the doses according to the detailed instructions provided (see Attachment 4).
    b. Adhere to good preparation practices, eg, clear an appropriate space; minimize distractions; open only 1 medication container at a time.
    c. Prepare medication that is appropriately labeled with the subject or subject number and initials, and the randomization number (include other label requirements specific to the protocol).
    d. Safely and efficiently deliver the prepared drug directly to the investigator or his or her designee.
    e. Complete all drug accountability and dispensing information on the drug accountability and dispensing records, including any amount returned by the subject or investigator. Quantities that are wasted or discarded must also be recorded.
    f. Return the randomization chart to the Wyeth Research Clinical Pharmacy Section.
    g. Not participate in the evaluation of any subject or subject.

5. The unblinded third party pharmacist may not administer the medication to the subjects. Contact between the unblinded third party pharmacist and subjects should be kept to a minimum.

ATTACHMENT 2. COLLECTION, PREPARATION, AND LABELING OF PLASMA SAMPLES

1. **Plasma (for drug concentration data):**
   - Collect 6 mL of blood into a single evacuated blood tube containing lithium heparin.
   - Invert the tubes gently 4 or 5 times (avoid shaking).
   - Blood must be placed on ice immediately, and centrifuged (at 1500g for 10 minutes) in a refrigerated centrifuge within 15 minutes following collection.
   - Aliquot into **two** watertight, labeled polypropylene containers. Distribute plasma evenly between containers
   - Store frozen in an upright position at about -70°C until shipped.

2. Pre-printed labels with a bar code will be provided by Wyeth which will identify each sample with the analyte to be assayed, protocol number, investigator name, subject number, matrix (plasma) and protocol day and time of sample collection. Hand writing information on the labels is not required, but in the event that it should be necessary to hand write information, only indelible ink should be used. Additional information handwritten on a computer-printed label **must not obscure the bar code**.

3. Apply preprinted labels to tubes at room temperature at least 2 hours before refrigerating or freezing. Adequate adhesion cannot be guaranteed if the labels are applied to cold tubes.

4. Apply the labels along the length of the tube with the colored bar at the top. Do not spiral or wrap around the circumference of the tube as this interferes with reading the bar code.

5. Secure the labels by wrapping with clear tape.

ALL SAMPLES MUST BE KEPT FROZEN AT APPROXIMATELY -70°C AFTER COLLECTION AND UNTIL SHIPMENT IS INITIATED.

ATTACHMENT 3. INSTRUCTIONS FOR SHIPPING FROZEN SAMPLES

1. The shipping list is included as the white page of the PK shipping worksheet from the case report form. If a separate shipping list is used, be sure to include the same information collected on the tube label.

2. Place the watertight plastic transfer tubes from each subject in watertight plastic bags, preferably 1 to 10 tubes per bag. Place the plastic bags in an insulated shipping container with the appropriate completed case report form shipping page(s). Fill the container with sufficient dry ice to maintain the samples in the frozen state for at least 48 hours (72 hours for international shipments), and seal with tape.

3. Label the containers as clinical specimens. If further information is needed, contact the WHO monitor.

4. Ship the sealed containers in sturdy outside packaging in accordance with the shipper's guidelines.

5. Make shipments as early in the week as possible - not later than Wednesday noon. They never should be made on a Friday or two days before a holiday. Consult your analytical laboratory representative for the dates of site-specific holidays.

6. Choose the carrier in collaboration with the WHO monitor.

7. Be sure that the carrier will pick up the container from your office. If they do not, however, a member of your staff should deliver the containers to the carrier's office.

8. It is extremely important that the airway bill prepared by the shipping agent contain the exact instructions and address given on the package label. The fact that the shipment contains a frozen biological packed in dry ice should be noted on the airway bill.

9. At the time shipment is initiated, notify the recipients (see instruction 11) and the clinical scientist (see instruction 12) by fax and (if appropriate) e-mail with the following information:
   - Carrier, Airway bill number, Expected time of arrival, Number of pieces in the shipment, Protocol number, and your name and fax number.
   - Note: The e-mail notification described here serves as an adjunct to the fax notification but does not replace it.

10. Ship plasma samples for moxidectin to:

Dr. Larry Fleckenstein
College of Pharmacy
University of Iowa
Iowa City, IA 52242

Phone: +1 (319)-335-8804
Fax: +1 (319)-353-5646

11. The recipient will acknowledge receipt of the sample shipment by fax and (if appropriate) e-mail within 24 hours to the responsible party at the clinical site, with a copy to the clinical scientist. Any irregularities in the shipment received will be promptly and duly noted by the recipient in the sample receipt notification.
12. Similarly, the status of delayed or missing shipments will be communicated within 24 hours of the expected time of arrival. Such issues will be resolved by follow-up communication between the responsible clinical site personnel and the recipient.
13. If you have any questions regarding shipment, please do not hesitate to call:

Russ Orrico
Senior Clinical Scientist
Wyeth Research-US
Phone: +1 (484)-865-5587
Fax: +1 (484)-865-0071
E-mail: orricor@wyeth.com

ATTACHMENT 4. INSTRUCTIONS FOR PREPARATION OF STUDY DRUG

1. The drugs are encapsulated moxidectin 2 mg tablets (TIC), encapsulated 3 mg ivermectin tablets (TIC) or matching placebo capsules. Moxidectin 2 mg TIC is the only strength available. Therefore, 1 TIC is required for the 2 mg dose; 2 TICs are required for the 4 mg dose; 4 TICs are required for the 8 mg dose.

2. Three dose regimens of moxidectin (2 mg, 4 mg, 8 mg) will be studied at 3 levels of intensity of infection (mild, moderate, severe). The weight-based ivermectin dosing schedule approved for the treatment of onchocerciasis will be used. The first 2 cohorts at each moxidectin dose level will comprise 16 subjects while the third cohort will comprise 32 subjects enrolled in 2 successive groups of 16. Thus, one hundred and ninety-two (192) subjects including both sexes are expected to enroll in this study; and will be admitted in 12 groups.

3. Subjects will be assigned a number on admission (subject number). The unblinded third party pharmacist will be supplied with 12 randomization schedules (one for each of the three severity levels within each of the three treatment strata and 3 additional schedules for the most severely infected subjects). Each randomization schedule will contain the 16 randomization numbers, as well as the corresponding treatment assignments. Treatment groups will be blocked in groups of 4, with a 3 to 1 ratio of moxidectin to ivermectin. Where there are less than 16 subjects enrolled, the leftover randomization numbers will be ignored and a new randomization schedule will be used when a new cohort is started.

4. The PI or clinical assessor will provide the unblinded third party pharmacist with a list of subjects who qualify for enrollment, and will identify the cohort for which drug is to be prepared by the dose group and the severity of infection. For the severely infected cohorts, the PI will add the information whether this is the first or the second 16 subject group to be treated. For each subject, the name, initials, age, sex, weight and subject number will be stated. The unblinded third party pharmacist will assign the randomization numbers at the time of preparing the study medication. Each randomization number will be matched with the corresponding subject number and, WITH THE EXCLUSION OF ALL PREPARATION INFORMATION, be provided to the PI to be placed onto the Case Report Form. At the end of the study, they will be used to identify the treatment group assigned to the subject.

5. The drugs will be prepared the night before administration (day –1). Moxidectin/ivermectin/placebo capsules will be stored locked at room temperature up to 30˚C.

Materials

Moxidectin capsules (containing 2 mg tablets)
Ivermectin capsules (containing 3 mg tablets)
Matching placebo capsules
Plastic cups (about 600 mL) with lids
Ordinary tap water
Markers
Sterile Disposable gloves
Refrigerator with key
Metal cabinet with key
Teaspoon (to dispense the tablets)
Dispensing tray
Small plastic envelops for dispensing tablets
Medium sized envelops (opaque)
Medium size trays-2
Self adhesive labels
Disposal Bin

Procedure

A. Day –1.

1. Treatment Allocation (Unblinded Third Party Pharmacist)

Using the randomization schedule supplied by Wyeth Research and the subject list provided, allocate each subject to the appropriate treatment regimen.

- Select the first male subject to be enrolled from the top of the randomization list that corresponds to the subject's assigned stratum. Similarly, select the first female from the bottom of the randomization list that corresponds to the subject's assigned stratum.

- Assign the second male subject to the next available treatment from the top of the randomization list that corresponds to the subject's assigned stratum. Assign the second female subject to the next available treatment from the bottom of the randomization list that corresponds to the subject's assigned stratum. Continue in this manner until the entire cohort has been assigned to the relevant treatment group.

- Complete the treatment allocation on the drug accountability form.

THIS FORM MUST NOT BE AVAILABLE TO ANYONE EXCEPT THE UNBLINDED MONITOR UNTIL THE END OF THE STUDY.

2. Preparing and Dispensing the Study Drugs (Unblinded Third Party Pharmacist)

- Label 16 medium size envelopes (one for each subject) with the subject's identification i.e. name, subject number, randomization number, age, sex, and date of drug administration.
- Wear the disposable gloves.
- Using the teaspoon and dispensing tray, dispense the appropriate number of moxidectin, placebo and/or ivermectin capsules for each subject into the small plastic envelopes and seal them. There should be a total number of 4 capsules in each envelope as per the tables below:

**Cohort**	**2 mg**					
Treatment Assignment	Moxidectin			Ivermectin		
Capsule	Moxidectin Capsule	Placebo Capsule	Ivermectin Capsule	Moxidectin Capsule	Placebo Capsule	Ivermectin Capsule
Subject Weight	Number of Capsules					
26-44 kg	1	3	0	0	2	2
45-64 kg	1	3	0	0	1	3
65-84 kg	1	3	0	0	0	4

**Cohort**	**4 mg**					
Treatment Assignment	Moxidectin			Ivermectin		
Capsule	Moxidectin Capsule	Placebo Capsule	Ivermectin Capsule	Moxidectin Capsule	Placebo Capsule	Ivermectin Capsule
Subject Weight	Number of Capsules					
26-44 kg	2	2	0	0	2	2
45-64 kg	2	2	0	0	1	3
65-84 kg	2	2	0	0	0	4

**Cohort**	**8 mg**					
Treatment Assignment	Moxidectin			Ivermectin		
Capsule	Moxidectin Capsule	Placebo Capsule	Ivermectin Capsule	Moxidectin Capsule	Placebo Capsule	Ivermectin Capsule
Subject Weight	Number of Capsules					
26-44 kg	4	0	0	0	2	2
45-64 kg	4	0	0	0	1	3
65-84 kg	4	0	0	0	0	4

- Put the small envelopes containing the tablets capsules into the corresponding labeled medium size envelopes. Seal the envelopes. Lock them up in a small cabinet at ≤30°C and keep the key.
- Dispose of all used disposable items (e.g. gloves, facemask) into a bin provided for that purpose.

- Prepare a list with the name, initials, age, sex, weight, subject number, and randomization number.

B. Day 1.

1. Morning of Drug Administration (Unblinded Third Party Pharmacist)

- Remove the medium size envelopes containing the capsules from the cabinet. Re-check all the labeling and arrange them according to subject numbers on the tray.
- Hand over the tray to the clinical staff in-charge of administering the drugs. Ensure he/she acknowledges receipt.
- Hand over the list with the name, initials, age, sex, weight, subject number and randomization number to the PI.
- After administration of the drugs, collect all the empty drug envelopes from the personnel that has dispensed the drugs. Check that all have been handed over.
- Write the name, subject number, randomization number, age, sex, weight, treatment regimen and date for each subject on a piece of paper and insert into the corresponding opaque envelope. Ensure that each tightly sealed envelope has been locked in a cabinet by the hospital pharmacist only to be opened if required for emergency unblinding.
- Record the drugs dispensed on the “Drug Stocks, Distribution and Accountability” Form.
- Lock up all documents in the cabinet for drug accounting purposes.

2. Administration of Study Drugs (Clinical Staff)

The study drugs will be administered after an overnight fast. Breakfast will be delayed for about 2 hours.

- Retrieve the drug envelopes for the subject from the tray. Put them in a kidney dish.
- Half fill the 600 mL plastic cups with ordinary tap water.

- With the assistance of one or more nurses, check the labeling on the drug envelopes containing the capsules. Ensure that the subject name and number correspond with those on the board above the head of the bed. Check the date.

- Call out the name on the label and listen to the subject verifying that he/she is the named individual.

- Open the drug envelope and give the capsules and the plastic cup containing about 300 mL of tap water to the subject.

- Observe the subject swallow the capsules and inspect the mouth to ensure compliance.

- Record the date and time in the source documentation that the capsules were swallowed.

ATTACHMENT 5. RECOMMENDATIONS FOR GRADING OF OCULAR EXAMINATIONS

Toxicity	Grade 0	1	2	3	4
**SYMPTOMS**					
Ocular discomfort	None	Mild: not interfering with function	Moderate: interfering with function, but not with ADL	Interfering with ADL	----
Ocular itching	None	Mild: not interfering with function	Moderate: interfering with function, but not with ADL	Interfering with ADL	----
Tearing (watery eyes)	None	Mild: not interfering with function	Moderate: interfering with function, but not with ADL	Interfering with ADL	----
Photophobia	None	Mild: not interfering with function	Moderate: interfering with function, but not with ADL	Interfering with ADL	----
Discharge	None	Present	----	----	----
Vision- blurred vision	Normal	Mild, not interfering with function	Symptomatic and interfering with function, but not interfering with ADL	Symptomatic and interfering with ADL	
**VISUAL ACUITY**					
(uncorrected) Snellen/ illiterate E	Unchanged or 6/4-6/6	Loss of 1 line	Loss of 2 lines	Loss of 3 lines	Loss of >3 lines
**COLOUR VISION**	Normal	Abnormal	----	----	----
**VISUAL FIELDS**[a]					
IV/4e kinetic	None	1-12	13-24	25-48	49-72
I4/e kinetic	None	1-12	13-24	25-36	37-60
I/2e	None	1-12	13-20	21-28	29-36
[a]Note: Based on the number of missed targets					
**PUPILLARY REFLEX**	Normal	Abnormal	----	----	----
**EXTERNAL EXAMINATION**					

Toxicity	Grade 0	1	2	3	4
			----	----	----
Eyelids	Normal	Abnormal	----	----	----
Conjunctiva	Normal	Abnormal	----	----	----
Cornea	Normal	Abnormal	----	----	----
Iris	Normal	Abnormal	----	----	----
Ocular movement	Normal	Abnormal			
**ANTERIOR SEGMENT INFLAMMATION**					
Conjunctivitis	None	Hyperaemia	Chemosis	-	
Limbitis					
Vascular changes	None	Mild: not interfering with function	Moderate: interfering with function but not with ADL	Interfering with ADL	
Globular infiltrates	None				
Anterior chamber					
Flare	None	Mild	Moderate	Severe	
Cells	None	Mild	Moderate	Severe	
**POSTERIOR SEGMENT**					
**OPTIC DISC**					
Hyperaemia	None	Sectorial	Overall	-	-
Swelling	None	-	-	Sectorial	Overall
**MACULA**	Normal	Mild: not interfering with function	Moderate: interfering with function, but not with ADL	Interfering with ADL	----
**PERIPHERAL RETINA**					
Retinitis	Absent	Present	----	----	----
Hemorrhage	Absent	Present	----	----	----
Vasculitis	Absent	Present	----	----	----
Cotton wool spots	Absent	Present	----	----	----
**RETINAL PHOTOGRAPHY**					
**Optic atrophy**					
Colour film	None	Linear nerve fibre loss	Sectorial atrophy	Overall atrophy	Atrophy with pigment
Red free	None	Linear nerve fibre loss	Sectorial fibre loss	Total loss	Total loss with pigment
**Pigment epithelial atrophy**					
Distribution	None	Temporal to macula	More than temporal	Continuous round macula	Whole of macula
Intensity	None	RPE mottling only	RPE mottling with <50% atrophy	RPE mottling with ≥50% atrophy	RPE mottling with hypertrophy

Toxicity	Grade 0	1	2	3	4
**FLUORESCEIN ANGIOGRAPHY**					
**Optic disc leakage**					
Within disc margin	None	Sectorial	Overall		
Beyond disc margin	None			Sectorial	Overall
**Optic atrophy**					
Red free	None	Linear nerve fibre loss	Sectorial fibre loss	Total loss	Total loss with pigment
**Pigment epithelial atrophy**					
Distribution	None	Temporal to macula	More than Temporal	Continuous round macula	Whole of macula
Intensity	None	RPE mottling only	RPE mottling with <50% atrophy	RPE mottling with ≥50% atrophy	RPE mottling with hypertrophy
Note: Once an event has occurred the score is retained at subsequent visits whether the lesion persists or not.					

Modified from Hero, M., A. C. Bird & K. Awadzi. (1992). *Quantification of the ocular reactions to microfilaricides in the chemotherapy of onchocerciasis*. Eye **6**(Pt 1): 93-96.

ATTACHMENT 6. POTENTIAL MAZZOTTI LABORATORY MANIFESTATIONS

Toxicity	Grade 0	1	2	3	4
**HEMATOLOGICAL**					
**G6PD (Additional)**	Normal	Partial defect	Total defect		
**Hemoglobin (Hgb)**	WNL	< LLN – 100 g/L	80 - < 100 g/L	65 – 80 g/L	< 6.5 g/dl
**Leukocytes (total WBC)**	WNL	< LLN - 3.0 x 10^9 /L	≥2.0 - < 3.0 x 10^9 /L	≥1.0 - < 2.0 x 10^9 /L	< 1.0 x 10^9 /L
	WNL	> 9.5-19.0 x 10^9 /L	>19.0-38.0 x 10^9 /L	>38.0-57.0 x 10^9 /L	> 57.0 x 10^9 /L
Note: Contributory factors include Mazzotti reaction (leucocytosis), coincidental infection (exclude) drug effect					
**Neutrophils/granulocytes** (ANC/AGC)	WNL	≥1500 - <2000/mm^3	≥1000 - <1500/mm^3	≥500 - <1000/mm^3	< 500/mm^3
	WNL	>6080-12160/mm^3	>12160-24320/mm^3	>24320-36480/mm^3	>36480/mm^3
Note: Contributory factors include Mazzotti reaction (neutrophilia), coincidental infection (exclude) drug effect					
**Lymphocytes**	WNL	<LLN – 1000/mm^3	≥500 - <1000/mm^3	<500/mm^3	-
	WNL	>636 5-12730/mm^3	>12730-25460/mm^3	>25460-38190/mm^3	>38190/mm^3
Note: Contributory factors include Mazzotti reaction (initial lymphopenia, lymphocytosis), coincidental infection (exclude) drug effect					
**Eosinophils**	WNL	20-26/mm^3	≥14-19/mm^3	≥7-13/mm^3	<7/mm^3
	WNL	>1425-2850/mm^3	>2850-5700/mm^3	>5700-8550/mm^3	>8550/mm^3
Note: Contributory factors include Mazzotti reaction (initial eosinopenia, eosinophilia), coincidental infection (exclude) drug effect					
**Platelets**	WNL	< LLN - <75.0 x 10^9 /L<br>< LLN – 75000/mm^3	≥50.0 - < 75.0 x 10^9 /L<br>≥50000 - < 75000/mm^3	≥10.0 - < 50.0 x 10^9 /L<br>≥10000 - < 50000/mm^3	< 10.0 x 10^9 /L<br>< 10000/mm^3
**Microfilariae/mL**	None	1-30	>30		
**Hemolysis** (e.g., immune hemolytic anemia, drug-related hemolysis, other)	None	Only laboratory evidence of hemolysis [e.g., direct antiglobulin test (DAT, Coombs') schistocytes]	Evidence of red cell destruction and ≥ 2gm decrease in hemoglobin, no transfusion	requiring transfusion and/or medical intervention (e.g., steroids)	catastrophic consequences of hemolysis (e.g., renal failure, hypotension, bronchospasm, emergency splenectomy)
**BIOCHEMICAL**					
**Hyponatremia**	WNL	<LLN – 130 mmol/L	-	120 - <130 mmol/L	<120 mmol/L
**Hypernatremia**	WNL	> ULN - 150 mmol/L	>150 - 155 mmol/L	>155 - 160 mmol/L	>160 mmol/L

Toxicity	Grade 0	1	2	3	4
**Hypokalemia**	WNL	<LLN - 3.0 mmol/L	-	2.5 - <3.0 mmol/L	<2.5 mmol/L
**Hyperkalemia**	WNL	> ULN - 5.5 mmol/L	> 5.5 - 6.0 mmol/L	> 6.0 - 7.0 mmol/L	> 7.0 mmol/L
**Bicarbonate**	WNL	< LLN - 16 mEq/dl	11 - 15 mEq/dl	8 - 10 mEq/dl	
**Hyperuricemia**	WNL	> ULN - ≤ 590 micromol/L without physiologic consequences	-	>ULN - ≤ 590 micromol/L with physiologic consequences	> 590 micromol/L
Note: Also consider Renal failure, Creatinine, Potassium.					
**Creatinine**	WNL	> ULN - 1.5 x ULN	> 1.5 - 3.0 x ULN	> 3.0 - 6.0 x ULN	> 6.0 x ULN
**SGPT (ALT)** (serum glutamic pyruvic transaminase)	WNL	> ULN - 2.5 x ULN	> 2.5 - 5.0 x ULN	> 5.0 - 20.0 x ULN	> 20.0 x ULN
Note: Elevations occur as part of the Mazzotti reaction but rarely reach 5 x ULN and usually last for less than 30 days					
**SGOT (AST)** (serum glutamic oxaloacetic transaminase)	WNL	> ULN - 2.5 x ULN	> 2.5 - 5.0 x ULN	> 5.0 - 20.0 x ULN	> 20.0 x ULN
Note: Elevations occur as part of the Mazzotti reaction but rarely reach 5 x ULN and usually last for less than 30 days					
**GGT** (γ - Glutamyl transpeptidase)	WNL	> ULN - 2.5 x ULN	> 2.5 - 5.0 x ULN	> 5.0 - 20.0 x ULN	> 20.0 x ULN
Note: Elevations occur as part of the Mazzotti reaction but rarely reach 5 x ULN and usually last for less than 30 days					
**LDH** (Lactate dehydrogenase)	WNL	> ULN - 2.5 x ULN	> 2.5 - 5.0 x ULN	> 5.0 - 20.0 x ULN	> 20.0 x ULN
Note: Elevations occur as part of the Mazzotti reaction but rarely reach 5 x ULN and usually last for less than 30 days					
**Alkaline phosphatase**	WNL	> ULN - 2.5 x ULN	> 2.5 - 5.0 x ULN	> 5.0 - 20.0 x ULN	> 20.0 x ULN
**Total Bilirubin**	WNL	> ULN - 1.5 x ULN	> 1.5 - 3.0 x ULN	> 3.0 - 10.0 x ULN	> 10.0 x ULN
**Hypoalbuminemia**	WNL	<LLN - 30 g/l	≥20 - <30 g/l	<20 g/dl	-
**Hypoglycemia**	WNL	<LLN - 3.0 mmol/L	2.2 - < 3.0 mmol/L	1.7 - < 2.2 mmol/L	< 1.7 mmol/L
**Hyperglycemia**	WNL	> ULN - 8.9 mmol/L	> 8.9 - 13.9 mmol/L	> 13.9 - 27.8 mmol/L	> 27.8 mmol/L or ketoacidosis
**Hypercholesterolemia**	WNL	> ULN - 7.75 mmol/L	> 7.75 - 10.34 mmol/L	>10.34 - 12.92 mmol/L	> 12.92 mmol/L
**Hypertriglyceridemia**	WNL	> ULN - 2.5 x ULN	> 2.5 - 5.0 x ULN	> 5.0 - 10 x ULN	> 10 x ULN

Toxicity	Grade<br>0	1	2	3	4
**URINALYSIS**					
**Proteinuria**	Normal or Trace	1+	2+ to 3+	4+	Nephrotic syndrome
**Hematuria (in absence of vaginal bleeding)**	None	Microscopic only	Intermittent gross bleeding	Persistent gross bleeding or clots; may require catheterization or instrumentation or transfusion	Open surgery or necrosis or deep bladder ulceration
**Microfilariae/10mL**	None	1-20	>20		

A Randomized, Single-Ascending-Dose, Ivermectin-Controlled, Double-Blind, Safety, Tolerability, Pharmacokinetic, and Efficacy Study of Orally Administered Moxidectin in Subjects with *Onchocerca volvulus* Infection

Onchocerciasis Chemotherapy Research Centre protocol number: 33

Wyeth Research protocol number: 3110A1-200-GH

Amendment 5

Date of Original Protocol:	18 November 2003
Date of Protocol Amendment 1:	21 April 2004
Date of Protocol Amendment 2:	14 July 2004
Date of Protocol Amendment 3:	27 October 2005
Date of Protocol Amendment 4:	10 May 2006
Date of Protocol Amendment 5:	10 Jan 2007

Sponsors:
Wyeth Research Division of Wyeth Pharmaceuticals Inc.
Clinical Research and Development
P.O. Box 8299
Philadelphia, Pennsylvania 19101

Product Development and Evaluation Unit
TDR
World Health Organization
20 Avenue Appia
CH-1211 Geneva 27
Switzerland

TABLE OF CONTENTS

1. CONTACTS 6
1.1 EMERGENCY CONTACTS 6
1.1.1 WYETH 6
1.1.2 NATIONAL CENTER FOR PHARMACOVIGILANCE 6
1.1.3 WORLD HEALTH ORGANIZATION 7
1.1.4 PRIMARY INVESTIGATOR 7
1.1.5 TRIAL CENTER IN GHANA (ALL SCREENING TO BE CONDUCTED AT THIS LOCATION) 7
2. INVESTIGATOR SIGNATURE 9
3. INDEPENDENT ETHICS COMMITTEE (IEC) 10
4. DISCLOSURE STATEMENT 11
5. LIST OF ABBREVIATIONS 12
6. DEFINITION OF TERMS 14
7. SYNOPSIS 16
8. SUBJECT FLOWCHART 23
9. INTRODUCTION 25
10. TRIAL DESIGN 34
10.1 DESCRIPTION 34
11. STUDY OBJECTIVES 37
11.1 PRIMARY 37
11.2 SECONDARY 37
12. SELECTION OF STUDY POPULATION 38
12.1 INCLUSION CRITERIA 42
12.2 EXCLUSION CRITERIA 42
12.3 NUMBER OF SUBJECTS 43
12.4 SCREEN FAILURES 43
13. PRIOR TREATMENT 44
14. CONCOMITANT TREATMENT 44
14.1 ADDITIONAL REQUIRED DRUGS 44
14.2 PERMITTED TREATMENT 44
14.3 PROHIBITED TREATMENT 45
15. TREATMENT OF SUBJECTS 45
15.1 PROCEDURES 45
15.1.1 DAYS -4 TO -2 (VISIT 1) 45
15.1.2 DAY -1 (VISIT 2) 46
15.1.3 DAY 1 (VISIT 3) 46
15.1.4 DAY 2 (VISIT 4) 46
15.1.5 DAY 3 (VISIT 5) 47
15.1.6 DAY 4 (VISIT 6) 47
15.1.7 DAY 5 (VISIT 7) 48

15.1.8 DAY 6 (VISIT 8) 48
15.1.9 DAY 7 (VISIT 9) 48
15.1.10 DAY 8 (VISIT 10) 48
15.1.11 DAY 9 (VISIT 11) 49
15.1.12 DAY 10 (VISIT 12) 49
15.1.13 DAY 11 (VISIT 13) 49
15.1.14 DAY 12 (VISIT 14) 50
15.1.15 DAY 13 (VISIT 15) 50
15.1.16 DAY 14 (VISIT 16) 50
15.1.17 DAY 15 (VISIT 17) 50
15.1.18 DAY 16 (VISIT 18) 51
15.1.19 DAY 17 (VISIT 19) 51
15.1.20 DAY 18 (VISIT 20) 51
15.1.21 1-MONTH (VISIT 21) 51
15.1.22 2-MONTHS (VISIT 22) 52
15.1.23 3-MONTHS (VISIT 23) 52
15.1.24 6-MONTHS (VISIT 24) 53
15.1.25 12-MONTHS (VISIT 25) 54
15.1.26 18-MONTHS (VISIT 26) 54
15.2 DISCONTINUATION AND WITHDRAWAL OF SUBJECTS 55
15.3 SPECIAL METHODS 56
15.3.1 PHYSICAL EXAMINATIONS AND MEDICAL HISTORY 56
15.3.2 VITAL SIGNS 56
15.3.3 ELECTROCARDIOGRAMS 57
15.3.4 OCULAR EXAMINATION 57
15.3.5 LABORATORY EVALUATIONS/SPECIMEN COLLECTION 57
15.3.6 SKIN SNIPS 58
16. TEST ARTICLE 59
16.1 TEST ARTICLE(S) AND ADMINISTRATION 59
16.2 PACKAGING AND LABELING 60
16.3 STORAGE AND STABILITY 61
16.4 PREPARATION 61
16.5 TEST ARTICLE ACCOUNTABILITY, RECONCILIATION, AND RETURN 62
16.6 STUDY SUBJECT COMPLIANCE 63
16.7 OTHER SUPPLIES 63
17. MEASURES TO MINIMIZE/AVOID BIAS 63
17.1 SUBJECT IDENTIFICATION 63
17.2 RANDOMIZATION 65
17.3 BLINDING AND UNBLINDING 66
18. SAFETY 67
18.1 SAFETY VARIABLES 67
18.2 SAFETY ASSESSMENT METHODS AND DOSE PROGRESSION DECISION 68
18.3 SAFETY LABORATORY DETERMINATIONS 70
19. EFFICACY 70
19.1 EFFICACY VARIABLES 70
19.1.1 PRIMARY 70

19.1.2 SECONDARY 70
19.2 EFFICACY ASSESSMENT METHODS 71
20. PHARMACOKINETIC ANALYSIS 72
21. STATISTICAL ANALYSIS 72
21.1 STATISTICAL METHODS 72
21.2 METHODOLOGY 73
21.3 SAMPLE SIZE AND POWER 74
22. CONTRAINDICATIONS, PRECAUTIONS, AND WARNINGS 74
23. ADVERSE EVENTS 76
23.1 DEFINITIONS 76
23.2 TIMING FOR REPORTING SERIOUS ADVERSE EVENTS 78
23.3 REPORTABLE EVENTS/INFORMATION 79
23.4 RECORDING AND REPORTING 80
24. DATA QUALITY ASSURANCE 82
25. INVESTIGATORS REGULATORY OBLIGATIONS 82
25.1 INDEPENDENT ETHICS COMMITTEE (GFDB/IEC) APPROVAL 82
25.2 PRESTUDY DOCUMENTATION 83
25.3 INFORMED CONSENT 84
25.4 DECLARATION OF HELSINKI 84
25.5 CASE REPORT FORMS 84
25.6 ADVERSE EVENT REPORTING 85
25.7 REVIEW OF SOURCE RECORDS 85
25.8 MONITORING OF THE STUDY 86
25.9 PROTOCOL AMENDMENTS 86
25.10 CHANGE IN INVESTIGATOR 86
25.11 TERMINATION OF THE STUDY 87
25.11.1 TERMINATION BY THE SPONSORS 87
25.11.2 TERMINATION BY THE INVESTIGATOR 87
25.12 FINAL STUDY REPORT 87
25.13 CONFIDENTIALITY 88
25.14 RECORDS RETENTION 88
25.15 PUBLICATIONS 88
25.16 SUBJECT INJURY 89

26. SPONSOR OBLIGATIONS IN CASE OF PREMATURE TRIAL TERMINATION 89
27. REFERENCES 90
ATTACHMENTS 92
ATTACHMENT 1. RESPONSIBILITIES OF THE UNBLINDED THIRD PARTY PHARMACIST 93
ATTACHMENT 2. COLLECTION, PREPARATION, AND LABELING OF PLASMA SAMPLES 94
ATTACHMENT 3. INSTRUCTIONS FOR SHIPPING FROZEN SAMPLES 95
ATTACHMENT 4. INSTRUCTIONS FOR PREPARATION OF STUDY DRUG 97
ATTACHMENT 5. OCRC CTC VERSION 2.0 103

1. CONTACTS

1.1 Emergency Contacts

1.1.1 Wyeth

Contact 1 of the persons indicated below for serious adverse events and other emergencies:

Wyeth Medical Monitor

Name:	Robert Maroko, MD
Country:	United States
Phone (Business Hours):	+1(484) 865-8566
Phone (After Hours):	+1(610) 202-0223
Fax:	+1(484) 865-0071

Wyeth Clinical Project Team Leader

Name:	Russ Orrico
Country:	United States
Phone (During business hours):	+1 (484) 865-5587
Phone (After business hours):	+1 (610) 653-6104
Fax:	+1 (484) 865-0071

Emergency Telephone Number: If the persons named above cannot be reached, call +1 (610)-902-1200 and indicate that you have a product-related emergency.

1.1.2 National Center for Pharmacovigilance

Name:	E. K. Agyarko
Country:	Ghana
Phone (1):	+233 (0) 21 661248
Phone (2):	+233 (0) 21 673090
Phone (3)	+233 (0) 21 779838
Phone (4)	+233 (0) 21 778196
Fax:	+233 (0) 21 660389

1.1.3 World Health Organization

WHO Clinical Coordinator

Name:	Juntra Karbwang Laothavorn, MD, PhD
Country:	Switzerland
Phone (Business Hours):	+41 (22) 791-3867
Phone (After Hours, Mobile):	+41 (79) 629-2163
Fax:	+41 (22) 791-4854

WHO Project Manager

Name:	~~Janis Lazdins-Helds, MD,~~ Annette C. Kuesel, PhD
Country:	Switzerland
Phone (During business hours):	+41 (22) 791-~~3818~~1541
Phone (After Hours, Mobile):	+41 (79) ~~509-0669~~596 5718
Fax:	+41 (22) 791-4774

1.1.4 Primary Investigator

Primary Investigator	Kwablah Awadzi, MB FRCP DCMT
Country:	Ghana
Phone (Business Hours):	+ 871 762 858781 (Satellite)
	+233 (0) 935 22 132
Phone (Mobile):	+233 (0) 20 201 7114
Fax:	+233 (0) 935 22111
	+871 762 858 783 (Satellite)

1.1.5 Trial Center in Ghana (all screening to be conducted at this location)

Onchocerciasis Chemotherapy Research Centre
Government Hospital
PO Box 144
Hohoe, Volta Region
Ghana

MEDICAL MONITOR SIGNATURE

I have read and approve this protocol. I assure that this study will be conducted according to all stipulations of the protocol, including all statements regarding confidentiality.

Wyeth Medical Monitor's Signature	Date of Signature (DDMmmYYYY)	Time (24-hour clock, time zone)

Wyeth Medical Monitor's Name (print)

WHO Clinical Coordinator's Signature	Date of Signature (DDMmmYYYY)	Time (24-hour clock, time zone)

Juntra Karbwang, M.D, PhD

WHO Clinical Coordinator's Name (print)

.

Time Zone Abbreviations:
Atlantic Time Eastern Time – AT ET, Central European Time – CET, Central Time – CT, East Australia Time – EAT, Greenwich Mean Time – GMT, Japan Time – JT, Mountain Time – MT, Pacific Time – PT)

2. INVESTIGATOR SIGNATURE

I have read this protocol and agree that it contains all necessary details for carrying out the study as described. I will conduct this protocol as outlined therein, including all statements regarding confidentiality. I will make a reasonable effort to complete the study within the time designated. I will provide copies of the protocol and access to all information furnished by the sponsors to study personnel under my supervision. I will discuss this material with them to ensure that they are fully informed about the drug and the study. I understand that the study may be terminated or enrollment suspended at any time by the sponsors, with or without cause, or by me if it becomes necessary to protect the best interests of the study subjects.

I agree to conduct this study in full accordance with all applicable regulations and Good Clinical Practice (GCP).

Investigator's Signature	Date of Signature (DDMmmYYYY)	Time (24-hour clock, time zone)

Kwablah Awadzi, MB FRCP DCMT
Investigator's Name (print)

3. INDEPENDENT ETHICS COMMITTEE (IEC)

The independent ethics committee (IEC) and its affiliation and address for *each site* implementing this protocol shall be identified below.

IEC:	Ghana IEC Ethical Review Committee (ERC)
Affiliation:	Ghana Health Service
Address:	P.O. Box GP-184
	Accra, Ghana

IEC:	WHO IEC (Research Ethics Review Committee (ERC))
Affiliation:	WHO
Address:	20 Avenue Appia<br>CH 1211 Geneva 27
	Switzerland

4. DISCLOSURE STATEMENT

Restricted Distribution of Documents

This document contains information that is confidential and proprietary to the sponsors. This information is being provided to you solely for the purpose of evaluating and/or conducting a clinical trial for the sponsors. You may disclose the contents of this document only to study personnel under your supervision, IECs, or duly authorized representatives of regulatory agencies for this purpose under the condition that they maintain confidentiality. The contents of this document may not be used in any other clinical trial, disclosed to any other person or entity, and/or published without the prior written permission of the sponsors. The foregoing shall not apply to disclosure required by any regulations; however, you will give prompt notice to the sponsors of any such disclosure.

Any information that may be added to this document also is confidential and proprietary to the sponsors and must be kept in confidence in the same manner as the contents of this document.

The Wyeth Research protocol number for this study is 3110A1-200-GH.

The Onchocerciasis Chemotherapy Research Centre protocol number is 33.

5. LIST OF ABBREVIATIONS

Abbreviation	Term
AE	Adverse event
ALT	Alanine aminotransferase (SGPT)
AP	Alkaline phosphatase
AST	Aspartate aminotransferase (SGOT)
ß-hCG	ß-human Chorionic Gonadotropin
BUN	Blood urea nitrogen
CBC	Complete blood count
CERT	Clinical Expert Review Team
CNS	Central nervous system
CRF	Case report form
CV	Curriculum vitae
DMPA	Depo-medroxyprogesterone acetate
EC	Ethics committee
ECG	Electrocardiogram
EMEA	European Medicines Evaluations Agency
ERC	Ethical Review Committee
FDA	United States Food and Drug Administration
G6PD	Glucose-6-phosphate dehydrogenase
GABA	Gamma aminobutyric acid
GCP	Good clinical practice
GGT	Gamma glutamyl transferase
GFDB	Ghana Food and Drugs Board
GHS	Ghana Health Services
GNCP	Ghana National Center for Pharmacovigilance
HPMC	Hypromellose (Hydroxypropyl Methylcellulose)
ICH	International Conferences on Harmonization
IV	Intravenous
IEC	Independent ethics committee
LDH	Lactate dehydrogenase
Mf	Microfilariae
mITT	Modified Intent-to-Treat
NOEL	No observed effect level
NTEL	No toxic effect level
NCI	National Cancer Institute
OCP	Onchocerciasis Control Programme
OCRC	Onchocerciasis Chemotherapy Research Center
OTC	Over the counter
PD	Pharmacodynamic
PRD	Product Research and Development Unit of TDR
PK	Pharmacokinetic
PI	Principal investigator
PRT	Project Review Team
SAE	Serious adverse event
t½	Terminal elimination half-life
TEAE	Treatment Emergent Adverse Event

Abbreviation	Term
TIC	Tablets in capsules
TDR	UNICEF/UNDP/Worldbank/WHO Special Programme for Research and Training in Tropical Diseases
ULN	Upper limit of normal
URTI	Upper Respiratory Tract Infection
WBC	White blood cell or White Blood Cell Count
WHO	World Health Organization
WNL	Within normal limits

6. DEFINITION OF TERMS

Term	Definition
Regulation	Throughout this document the term *regulation* refers to all appropriate regulations, laws, and guidelines. This study will be conducted according to all appropriate regulations. The regulations may be international, national, or local and may include but not be limited to the Code of Federal Regulations (United States); the Good Clinical Practice: Consolidated Guideline (Canada); the International Conference on Harmonisation Guideline for Good Clinical Practice; the Therapeutic Goods Administration Annotated International Conference on Harmonisation Guidelines (Australia); the Standard Operating Procedures for Clinical Investigators (UNDP/World Bank/WHO Special Programme for Research and Training in Tropical Diseases); the World Medical Association Declaration of Helsinki: Ethical Principles for Medical Research Involving Human Subjects (See Declaration of Helsinki section).
Regulatory Agency	Throughout this document the term *regulatory agency* refers to all appropriate health and regulatory agencies. These may be international, national, or local and may include but not be limited to the Australian Therapeutic Goods Administration (TGA), the Canadian Health Products and Food Branch (HPFB), the European Agency for the Evaluation of Medicinal Products (EMEA), the US Food and Drug Administration (FDA), and the Ghana Food and Drugs Board (GFDB).
Sponsors	Throughout this document the term *sponsors* refers to all appropriate research departments. These may include but not be limited to Wyeth Research Division of Wyeth Pharmaceuticals Inc. and Product Research and Development Unit (TDR), World Health Organization.
Study Start Date	Date the first subject is admitted to the hospital for the study.
Subject	Any person who participates in a study, either as the recipient of a test article or as a control subject. A subject may be healthy or have a disease.
Test Article	Any drug, device, biological agent (including placebo) required by the

Term	Definition
	protocol and supplied by the sponsors for use in the sponsor's clinical research and development studies.

7. SYNOPSIS

**Study Title**	A Randomized, Single-Ascending-Dose, Ivermectin-Controlled, Double-Blind, Safety, Tolerability, Pharmacokinetic, and Efficacy Study of Orally Administered Moxidectin in Subjects with *Onchocerca volvulus* infection.
**Clinical Phase**	Phase 2
**Study Rationale**	Moxidectin, an anthelmintic, is currently licensed and marketed as an antiparasitic for use in cattle, sheep, swine, horses, and dogs. Preclinical experiments have shown that orally administered moxidectin may be useful treatment for onchocerciasis (river blindness) in humans. Moxidectin kills the microfilariae and may kill adult worms as well. Ivermectin, a related drug that is currently the standard treatment for river blindness, kills only the microfilariae. The first-in-man (FIM) study (protocol 3110A1-100-EU) investigating the effect of single doses of 3 mg to 36 mg of moxidectin in healthy subjects has been completed. In this study, 37 subjects were treated with single doses of 3 mg to 36 mg (approximately 50 μg/kg to 600 μg/kg). Subjects were randomized into placebo, 3 mg fasting, 9 mg fasting, 9 mg fed, 18 mg fasting, 36 mg fed and 36 mg fasting groups. Only 1 adverse event (AE) higher than grade 2 was reported in this study (enteritis due to food poisoning [grade 3] in the 36 mg fasting group) and was considered unrelated to study drug. The most common AEs were headache (35%) and infection (29%). Only half of the headaches reported and none of the infections reported were regarded as study drug related. The reported infections, including type of infection, time of occurrence after moxidectin administration, and dose group were: upper respiratory tract infection (URTI) on Day 19 and Day 63 (same subject), and a viral URTI on Day 14 in the 3 mg fasted group; toe infection on Day 52 in the 9 mg fasted group – URTI on Day 30 and Day 78 (different subjects) in the 9 mg fed group; tooth abscess on Day 47 in the 36 mg fasted group; head cold on Day 50 and Day 66 (different subjects) in the 36 mg fasted group; cold on Day 42 and cold-like symptoms on Day 59 (same subject) in the 36 mg fed group. No serious adverse events (SAEs) were reported. Four (4) subjects discontinued from the study (1 before receiving study drug and 3 after dose administration [one each in the 3 mg fasting, 9 mg fed, and 36 mg fasting groups]). The withdrawal from the study was unrelated to AEs. Overall, moxidectin appeared to be safe at the doses evaluated in this study.<br>In a bioavailability study comparing the tablet and liquid forms of

	moxidectin (protocol 3110A1-101-EU) 58 healthy male subjects received a single dose of 10 mg of moxidectin either as the liquid formulation (29 subjects) used in the FIM study or as tablets (29 subjects) and were followed up for 180 days. During the course of this study, there were no SAEs and no subjects discontinued the study due to an AE. A total of 36 (62.1%) subjects had treatment emergent AEs (TEAEs) during the study, with the same number and percentage of subjects (18; 62.1%) reporting TEAEs in both the moxidectin liquid and tablet groups. During the first 7 days post treatment, 10 subjects (34.5%) receiving liquid moxidectin and 9 subjects (31.0%) receiving tablet reported AEs, including asthenia (10.3% in the tablet group), headache (13.8% and 6.9% in the liquid and tablet groups, respectively), infection (6.9% in the liquid group), diarrhea (6.9% in the liquid group), myalgia (6.9% in the tablet group), and dizziness (6.9% in the tablet group). Between day 8 and 180 of follow up, 10 subjects (34.5%) receiving liquid and 12 subjects (41.4%) receiving tablet reported AEs. The most commonly reported events were flu syndrome (17.2% and 20.7% in liquid and tablet groups, respectively), headache (6.9% in the tablet group), and infection (6.9% in the tablet group). All of the TEAEs were mild to moderate in intensity, and none were considered to be related to treatment. No clinically relevant abnormalities were observed in vital sign measurements, ECGs, or laboratory tests during the study.<br>The data from the normal subject studies (protocol 3110A1-100-EU, 3110A1-101-EU) suggests that progression to a safety and tolerability study in subjects with different degrees of severity of infection with *Onchocerca volvulus* as described in this protocol is warranted.<br>This will be the first study of moxidectin in subjects with *O. volvulus* infection and will examine the safety, tolerability, pharmacokinetics, and efficacy of oral moxidectin in subjects to determine safe and effective doses of moxidectin and provide proof of concept. The lowest dose of moxidectin to be used in this study (2 mg) is lower than the lowest dose used in the FIM study (3 mg). As noted above, the 3 mg dose was well tolerated in the FIM study.
**Trial Design**	This is a randomized, single-ascending dose, double blind, active drug-controlled, parallel design, inpatient/outpatient, study at a single center. See the flowchart(s) for the procedures to be performed at each visit.
**Approximate Duration of Subject Participation**	Each subject will participate for approximately 18 months including an initial 18-day inpatient period.

**Approximate Duration of Study**	The total duration of the study will be up to 36 months.
**Study Objective(s)**	**Primary:** To determine the safety and tolerability of orally administered moxidectin in subjects with *O. volvulus* infection, as measured by the incidence of clinical adverse events and clinically significant laboratory test results.<br>**Secondary:**<br>1. To determine doses that effectively eliminate microfilariae and prevent their return to the skin as measured by the skin microfilarial loads at day 8, 1 month, 2 months, 3 months, 6 months, 12 months and 18 months after treatment.<br>2. To determine the viability and fertility of adult worms at 18 months.<br>3. To assess the pharmacokinetics of moxidectin in male and female adult subjects.
**Diagnosis and Main Criteria for Inclusion**	Men and women in good general health, with *O. volvulus* infection and:<br>a) Written, signed (or thumb-printed), and dated informed consent<br>b) Aged 18 to 60 years, inclusive<br>c) Body weight ≥ 40 kg for women and ≥ 45 kg for men<br>d) Nonpregnant, nonbreastfeeding women. Women of child bearing potential must agree to use birth control during the first 150 days after treatment.<br>e) Healthy, as determined by a physician on the basis of a physical examination, ECG, and a thorough review of the medical history and clinical laboratory results<br>f) Adequate hematologic, renal, and hepatic function, defined as:<br>1) White blood cell (WBC) count ≥ 2,800 and ≤ 11,300 cells/mL<br>2) Hemoglobin: ≥ 11.0 g/dL for men and ≥ 10.0 g/dL for women<br>3) Platelet count: ≥ 110,000 mm^3<br>4) Serum creatinine: ≤ 1.25 x upper limit of normal (ULN)<br>5) Total bilirubin: ≤ 1.25 x ULN<br>6) AST/SGOT: ≤ 1.25 x ULN<br>7) AP: ≤ 1.25 x ULN<br>8) Prothrombin time WNL<br>9) Urinalysis WNL<br>g) Skin microfilarial density within the required range for the cohort
**Main Criteria for Exclusion**	a) Participation in any studies other than purely observational ones, within 4 weeks before test article administration.<br>b) Any vaccination within 4 weeks before test article administration<br>c) Acute infection requiring therapy within the last 10 days before test article administration

	d) Administration of any medication (with the exception of medication required to treat any reactions during the screening fluorescein angiography (chlorpheniramine) or paracetamol) or herbal preparation within 10 days prior to test article administration or any condition currently requiring regular medication<br>e) Clinically significant ECG abnormalities or history of cardiac abnormality<br>f) Past or current history of neurological or neuropsychiatric disease or epilepsy<br>g) Subjects with orthostatic hypotension at the screening evaluation<br>h) History of drug or alcohol abuse or regular use of ≥ 3 cigarettes per day<br>i) Use of alcohol or other drugs of abuse within 72 hours before test article administration<br>j) Any condition, in the investigator's opinion, that places the subject at undue risk<br>k) Subjects who have donated blood within 8 weeks before study entry<br>l) Subjects with ocular onchocerciasis in cohorts intended to enroll subjects with mild infection. Ocular onchocerciasis is defined by the presence of live or dead microfilariae, onchocercal punctate opacities, onchocercal lesions of the posterior segment or lesions that mimic those seen in onchocerciasis.<br>m) Subjects with hyperreactive onchodermatitis<br>n) Antifilarial therapy within the previous 5 years<br>o) Coincidental infection with Loa Loa<br>p) Female subjects of childbearing potential with a contraindication to DMPA if not on Norplant<br>q) Any other condition which the investigator feels would exclude the subject from the study
**Approximate Number of Subjects**	A total of 192 subjects will be enrolled in 9 consecutive cohorts:<br>2 mg: 16 subjects with > 0 and < 10 microfilariae/mg of skin<br>2 mg: 16 subjects with 10-20 microfilariae/mg of skin and the sum of microfilariae in the two eyes must be ≤ 10<br>2 mg: 32 subjects with > 20 microfilariae/mg of skin with or without ocular involvement (to be enrolled in 2 consecutive groups of 16 subjects)<br><br>4 mg: 16 subjects with > 0 and < 10 microfilariae/mg of skin<br>4 mg: 16 subjects with 10-20 microfilariae/mg of skin and the

	sum of microfilariae in the two eyes must be ≤ 10<br>4 mg: 32 subjects with > 20 microfilariae/mg of skin with or without ocular involvement (to be enrolled in 2 consecutive groups of 16 subjects)<br><br>8 mg: 16 subjects with > 0 and < 10 microfilariae/mg of skin<br>8 mg: 16 subjects with 10-20 microfilariae/mg of skin and the sum of microfilariae in the two eyes must be ≤ 10<br>8 mg: 32 subjects with > 20 microfilariae/mg of skin with or without ocular involvement (to be enrolled in 2 consecutive groups of 16 subjects)
**Approximate Number of Study Centers**	1
**Concomitant Treatment**	Depo-medroxyprogesterone acetate (DMPA), 150 mg, will be given twice to all women of childbearing potential. The first dose will be given on study day 11 and the second dose will be at month 2 (±1 week). Women already receiving a parenterally administered contraceptive ~~who have contraceptive protection by the Norplant system~~ will be excluded from the protocol-defined DMPA injections schedule.<br>Chlorpheniramine may be administered to reduce adverse reactions to fluorescein sodium and paracetamol may be administered for painful conditions. All medication given after admission to the hospital will be documented in the CRF. Treatment with ivermectin, albendazole, or other anthelmintic agents will be prohibited for the duration of the study.
**Test Article(s) and Administration**	Moxidectin, ivermectin and placebo capsules are identical in appearance. Moxidectin tablets in capsules (TIC) will be supplied as 2 mg tablets encapsulated in hydroxypropyl methylcellulose (HPMC) capsules. The capsules are filled with a mixture of inert ingredients including lactose, microcrystalline cellulose, sodium starch glycolate and magnesium stearate. One (1) moxidectin TIC is required for a 2 mg dose; 2 TICs are required for a 4 mg dose; 4 TICs are required for an 8 mg dose.<br>Ivermectin TIC will be supplied as 3 mg tablets encapsulated in HPMC capsules filled with the same inert ingredients as the moxidectin capsules. Ivermectin will be dosed according to the weight-based dosing schedule approved for the treatment of onchocerciasis in the ivermectin package insert ranging from 6-12 mg. Placebo will be supplied as HPMC capsules filled with the same inert ingredients as the moxidectin and ivermectin capsules. Each subject will receive a single dose of 4 capsules, including moxidectin, moxidectin plus placebo, ivermectin or ivermectin plus

	placebo depending on body weight and treatment assignment.
**Safety Evaluation**	Vital signs, ECGs, physical examinations (including a neurologic examination), ocular examinations, AEs, serum chemistries, hematology studies, prothrombin times, and urinalyses.
**Efficacy Evaluation**	Efficacy evaluations (skin snips) will be performed at day 8, 1 month (± 1 week), 2 months (± 1 week), 3 months (± 1 week), 6 months (± 1 month), 12 months (± 1 month) and 18 months (± 1 month) after dosing and the number of microfilariae/mg of skin determined. Evaluation of the viability and fertility of the adult worms will occur at 18 months. All located nodules will be excised at 18 months, processed for histopathology, and slides interpreted by one or more observers. Microfilarial migration into blood and urine will be used as indirect evidence of the speed of microfilaricidal activity.<br>All subjects who have skin microfilariae at the 18-month visit will be treated with the approved dosage of ivermectin.
**Pharmacokinetics**	Blood samples for determination of moxidectin plasma concentrations will be collected within 2 hours prior to dosing (0 hr), and at 1, 2, 4, 8, 24, and 72 hours after study drug administration and on study days 8, 13, 18, 1 month (± 1 week), 2 months (± 1 week), 3 months (± 1 week), 6 months (± 1 month), and 12 months (± 1 month). Pharmacokinetic (PK) samples will be drawn from all subjects in all cohorts with the exception of the 2nd group of 16 subjects with >20 microfilariae/mg of skin in cohorts 3, 6, and 9. Samples from subjects will be analyzed by a validated bioanalytical method.<br>A PK analysis will be performed using model independent methods to characterize the PK parameters of moxidectin, such as maximum plasma concentration (C_{max}), time to reach the maximum plasma concentration (t_{max}), area under the plasma concentration vs. time curve (AUC) and the apparent terminal elimination half-life ($t_{1/2}$).
**Pharmacodynamics**	Analysis of a correlation between PK parameters, gender, severity of infection, efficacy, and efficacy-related AEs.
**Statistical Analysis**	This is a randomized, double blind, active-control, dose-escalation trial comparing 3 doses of moxidectin (2, 4, and 8 mg) with ivermectin, stratified by microfilariae density (mild, moderate, severe) and gender. Subjects will be treated as inpatients for the first 18 days and as outpatients for the remainder of the 18-month study.<br>The primary analysis population for safety will be the modified intent-to-treat (mITT) population, which consists of all subjects who receive study

	medication. For efficacy, the primary analysis population will be the evaluable subject population, which is defined as all subjects who receive study medication and have baseline and 18 month microfilariae count data. An mITT sensitivity analysis will also be performed to evaluate the effect on the efficacy results of dropouts occurring before 18 months.<br>The primary objective of the study is to evaluate the safety of escalating moxidectin regimens. Both the incidence of clinical AEs and clinically significant laboratory test results will be used to assess safety. The primary efficacy endpoint is the reduction from baseline in skin microfilariae at 18 months. Microfilariae count will be defined as the geometric mean of skin microfilariae/mg taken at 4 sites (iliac crests and calves).<br>In addition, important secondary endpoints are the reduction from baseline in skin microfilariae at 8 days, 1 month, 2 months, 3 months), 6 months, and at 12 months, area under the curve (calculated by using the trapezoidal method through the 18-month visit), ocular examination (time of maximum reduction of microfilariae and number (%) of subjects with complete clearance) and nodulectomy (viability and fertility of macrofilariae) at 18 months.
**Rationale for Number of Subjects**	At each dose level, 16 mildly, 16 moderately and 32 severely infected subjects will be treated with moxidectin or ivermectin in a 3:1 ratio. The probability of detecting at least 1 AE of grade 2 or higher among 12 moxidectin-treated subjects will be 0.114 and 0.718 when the true rates are 1% and 10%, respectively. The probability of detecting at least 1 AE of grade 2 or higher among 24 moxidectin-treated subjects will be 0.214 and 0.92 when the true rates are 1% and 10%, respectively.

8. SUBJECT FLOWCHART

	Visit Number																									
**Clinical Planned Events**	1	2	3	4	5	6	7	8	9	10	11	12	13	14	15	16	17	18	19	20	21	22	23	24	25	26
	Study Day																									
**Study Procedures**	**-4 to -2**	**-1**	**1**	**2**	**3**	**4**	**5**	**6**	**7**	**8**	**9**	**10**	**11**	**12**	**13**	**14**	**15**	**16**	**17**	**18**	**1 mo ±1 wk**	**2 mo ±1 wk**	**3 mo ±1 wk**	**6 mo ± 1 mo**	**12 mo ±1 mo**	**18 mo ±1 mo**
**Informed consent**[a]	~~X~~																									
**Demographics**	X																									
**Admission**	X																									
**Medical history**	X																									
**Medication history**	X																									
**Physical examination**	X																				X	X	X	X	X	X
**Height**	X																									
**Weight**	X		X	X	X	X	X	X	X	X	X	X	X	X	X	X	X	X	X		X	X	X	X	X	X
**Vital signs**	X	X	X	X	X	X	X	X	X	X	X	X	X	X	X	X	X	X	X	X	X	X	X	X	X	X
**ECG**	X		X[b]	X	X					X																
**Ocular examination**	X				X				X							X					X	X	X'	X	X	X
**Retinal Colour Photographs and Fluorescein angiogram**[c]	X				X				X							X					X	X	X	X	X	X
**Interim physical examination**			X	X	X	X	X	X	X	X	X	X	X	X	X	X	X	X	X							
**Hematology**[d]	X		X	X		X				X					X					X	X	X	X	X	X	X
**Serum chemistry**	X		X	X		X				X					X					X	X	X	X	X	X	X
**Pregnancy test**		X																								
**Urinalysis**[d]	X		X	X		X				X					X					X	X	X	X	X	X	X
**Clinical Planned Events**	1	2	3	4	5	6	7	8	9	10	11	12	13	14	15	16	17	18	19	20	21	22	23	24	25	26

a. ~~Will~~ Informed consent will be obtained in the village prior to transport of potential study participants to the study centre and before any study-related procedures are performed~~admission~~. Informed consent may be obtained prior to day -4.
b. ECG to be done approximately 4 hours after study drug administration. Can be repeated on days 4 through 7 if clinically indicated.
c. For all subjects until month 3, then only in subjects with lesions or visual defects.
d. All blood and urine samples will be filtered and stained for microfilariae.

SUBJECT FLOWCHART (Continued)

	Visit Number																									
**Clinical Planned Events**	**1**	**2**	**3**	**4**	**5**	**6**	**7**	**8**	**9**	**10**	**11**	**12**	**13**	**14**	**15**	**16**	**17**	**18**	**19**	**20**	**21**	**22**	**23**	**24**	**25**	**26**
	**Study Day**																									
**Study Procedures**	**-4 to -2**	**-1**	**1**	**2**	**3**	**4**	**5**	**6**	**7**	**8**	**9**	**10**	**11**	**12**	**13**	**14**	**15**	**16**	**17**	**18**	**1 mo ±1 wk**	**2 mo ±1 wk**	**3 mo ±1 wk**	**6 mo ± 1 mo**	**12 mo ±1 mo**	**18 mo ±1 mo**
**Skin snips**	X									X											X	X	X	X	X	X
**Randomization**		X																								
**Drug packaging and dispensing**		X																								
**Double-blind drug administration**			X[e]																							
**Moxidectin Plasma Samples**		X[f]	X[g]	X		X				X					X					X	X	X	X	X	X	
**Adverse events**	X	X	X	X	X	X	X	X	X	X	X	X	X	X	X	X	X	X	X	X	X	X	X	X	X	X
**DMPA[h]**													X									X				
**Discharge**																				X						
**Nodulectomy**																										X[i]
**Clinical Planned Events**	1	2	3	4	5	6	7	8	9	10	11	12	13	14	15	16	17	18	19	20	21	22	23	24	25	26

e. Will be given after an overnight fast and 2 hours before breakfast.
f. Day -1 sample will be taken approximately 2 hours before drug administration.
g. Day 1 samples will be taken at 1, 2, 4, and 8 hours after drug administration.
h. Depo-medroxyprogesterone acetate (DMPA) on women of childbearing potential not ~~protected by Norplant system.~~already receiving a parenterally administered contraceptive.
i. All located nodules will be processed for histopathology and slides read by 1 or more blinded observers.

9. INTRODUCTION

Moxidectin is a macrocyclic lactone drug that is derived from the actinomycete *Streptomyces cyanogriseus* (eg, milbemycin). The compound was developed by Fort Dodge Animal Health as a veterinary product, and is registered worldwide for the prevention of canine heartworm (ProHeart®) and for the treatment of internal and external parasites in cattle, sheep, and horses (Cydectin®).

The World Health Organization (WHO) is interested in using moxidectin for the control of human onchocerciasis. Onchocerciasis, often referred to as river blindness, is a parasitic disease caused by the filarial worm *Onchocerca volvulus* and is transmitted between humans through the bite of black flies of the genus *Simulium*. Onchocerciasis is still endemic in 34 countries: 27 in WHO's African region, 6 in the region of the Americas, and 1 in the Eastern Mediterranean region. Each adult female worm, which can live for an average of 11 years but up to 18 years in the human body, produces millions of microfilariae that migrate throughout the body. The manifestations of onchocerciasis are caused by the microfilariae. Onchocerciasis primarily affects the skin, eyes, and lymph nodes. Severe itching and rash are the most frequent manifestations of the disease. Visual impairment, including blindness, is the most serious consequence. Both manifestations have serious social and economic consequences that justify the vigorous efforts being made to control the disease. The current estimate is that approximately 18 million people are infected, of whom 6.5 million suffer from severe itching or dermatitis, 270,000 are blind, and a further 500,000 are severely visually disabled.

The ultimate goal of the WHO's African Programme for Onchocerciasis Control is to eliminate onchocerciasis as a disease of public health and socioeconomic importance. Currently, the strategies to control the disease include killing the larvae of the black fly vector with insecticides and the treatment of populations at risk with the macrocyclic lactone drug ivermectin (Mectizan®). Ivermectin is a microfilaricide that must be administered at least once per year to control the disease by reducing the numbers of microfilariae in the skin and eyes. Because this treatment has to be given for the life span of adult worms and does not protect subjects from reinfection, it is widely recognized that an alternate treatment is needed, preferably a macrofilaricide that can effect a cure. In addition to its microfilaricidal properties, ivermectin has been shown to have some activity against other stages of *Onchocerca volvulus*: it has a partial in-vivo effect against third-stage, but not fourth-stage, larvae in experimentally infected

chimpanzees[1]; after multiple dosage, it reduces insemination and impairs oogenesis in adult female worms[2]; and causes some decrease in adult worm numbers and viability.[3]

Moxidectin has some known mechanistic differences from ivermectin. It is considered significantly more potent than ivermectin and may also disrupt additional stages within the parasitic life cycle. Although structurally related to ivermectin, moxidectin is more lipophilic and is therefore expected to have a longer duration of action in humans than ivermectin after oral administration.

Pharmacology

The exact mechanism of action of moxidectin is still under investigation. Studies indicate that moxidectin binds to glutamate-gated chloride channels in the neurons and muscle cells of parasites.[4] Binding to the ion channel results in hyperpolarization of the nerve and muscle fibers leading to paralysis and death of the parasitic organism. Specificity of moxidectin for the parasite versus the mammalian host results from this compound having a low affinity for the mammalian chloride channels. An additional action of moxidectin is its activity at the GABA-A (gamma aminobutyric acid-A) receptor complex.[5] The multidrug transporter P-glycoprotein has been implicated in the mechanism of resistance to macrocyclic lactones.[6] However, moxidectin has been shown to control parasites that are resistant to other macrocyclic lactones as well as benzimidazoles[7,8] suggesting a different mechanism or susceptibility to resistance. A different binding activity has been demonstrated for a GABA-gated chloride ion channel between ivermectin and moxidectin.[9]

Various in vitro and in vivo studies in mice, jirds, dogs, and cattle have evaluated the activity of moxidectin against *Onchocerca* species and *Brugia pahangi*. In in vitro test systems, moxidectin, ivermectin, doramectin, and milbemycin oxime were evaluated against microfilariae from *Onchocerca volvulus* and *Onchocerca lienalis* in 7-day motility assays. Worm motility was significantly reduced by all 4 compounds at 2 hours, followed by a steady decline up to 120 hours. At the end of the study, the mean percentage reductions in worm motility for ivermectin, moxidectin, doramectin, and milbemycin oxime were 86%, 93%, 93%, and 74%, respectively.[10,11]

Studies in CBA/Ca mice experimentally infected with *Onchocerca lienalis* and *Onchocerca volvulus* consistently showed that subcutaneous or oral moxidectin was more efficacious than ivermectin in clearing microfilariae.[10, 11]

Investigation of Guadali cattle in Cameroon addressed the effect of moxidectin and ivermectin on preventing infection of *Onchocerca ochengi* L3 to L4 stage larvae in naïve calves under very high natural challenge (infection) conditions. Ivermectin (150 µg/kg) or moxidectin (200 µg/kg) was subcutaneously administered monthly or at 3-month intervals. At the end of the study, 11 of 14 control animals had 110 nodules. Animals treated with ivermectin every month had no nodules, but 2 of 10 animals treated with ivermectin every 3 months had 2 nodules. In moxidectin-treated animals, no nodules were found in animals treated monthly or once every 3 months.[12]

The activity of moxidectin against *Brugia pahangi* was evaluated in a jird and a dog model. In the jird model, the effect of moxidectin, ivermectin, and doramectin on microfilariae was determined. Moxidectin induced a rapid reduction in microfilariae, with none detectable after day 224. Ivermectin and doramectin treatment resulted in a gradual reduction in microfilariae through day 336; however, most jirds still had few microfilariae remaining. The effect on macrofilariae (adult worms) was also assessed. Treatment of jirds with moxidectin resulted in >90% killing of adult worms, whereas ivermectin or doramectin did not have any effect on adult worm burden.[13] In the dog model, moxidectin at doses of 250 µg/kg or 1000 µg/kg was given 1, 3, 6, or 12 times over a 12-month period. After the first dose of moxidectin, the microfilariae count for all treated dogs gradually declined from pretreatment levels during the first 2 weeks after treatment. By 3 months, the microfilariae counts were suppressed 90% or more from pretreatment levels. The microfilariae counts remained suppressed to the end of the study at 12 months, with 3 of the 78 moxidectin-treated dogs not clearing microfilariae.[14] In the same study, the effect of moxidectin on the macrofilariae was evaluated. At 6 months after the first dose, dogs treated with moxidectin at 250 µg/kg for 3 or 6 doses had 77% and 92% fewer worms, respectively, than controls. At 12 months after the first dose, dogs given 1 or more doses of moxidectin at either 250 µg/kg or 1000 µg/kg had at least 92% fewer worms than controls, with only 6 of 48 treated dogs having live adult worms at necropsy. The worm recoveries from all of the moxidectin treatments were significantly less ($p<0.05$) than the recoveries for the controls, but there was no significant difference ($p> 0.05$) among the worm recoveries for the different moxidectin treatments. In conclusion, moxidectin was a potent microfilaricidal agent. The suppression of microfilariae is independent of the dosage or the number of treatments and, in the dog model, the effect is sustained for at least 1 year. Furthermore, moxidectin was also effective against *B. pahangi* adults.[14]

Animal data indicate that moxidectin is highly lipophilic, residing primarily in fatty tissue. The compound has a long half-life, approximately 20 days in dogs, sheep, and horses. Based on initial PK

data for the 3 mg to 36 mg dose groups in the single-ascending-dose FIM study (protocol 3110A1-100-EU) conducted in volunteer subjects, mean half-life ($t_{1/2}$) in humans ranges from approximately 19.9 to 37.4 days.

Toxicology

Safety studies have been completed in mice, rats, dogs, sheep, cattle, horses, and humans. Repeated-dose toxicity was evaluated in oral (diet) studies in mice (4 weeks), rats (4 and 13 weeks), and dogs (4 and 13 weeks and 1 year), and in oral (diet) carcinogenicity studies in mice and rats (2 years). Results of the repeated-dose studies indicate that moxidectin was well tolerated with no toxicologic effects at calculated dosages of up to 6.9 mg/kg/day in mice for 4 weeks, 3.9 mg/kg/day in rats for 13 weeks, or 1.1 mg/kg/day in dogs for 1 year. At toxic dose levels, events such as decreased activity, prostration, tremors, chromodacryorrhea, decreased respiration, diarrhea, hypersensitivity to touch and sound, and epistaxis were observed. However, the no–toxicologic-effect levels (NTELs) in all animal models were high multiples of the anticipated human dosage on the basis of body weight. The anticipated maximum human dose is a single 8 mg dose per year (0.16 mg/kg for a 50 kg human). The lowest NTEL in single dose studies in the most sensitive species (mice) was 78 mg/kg, the NTEL in repeated dose studies were 6.9 mg/kg/day in mice for 4 weeks, 3.9 mg/kg/day in rats for 13 weeks, and 0.8 mg/kg/day and 1.1 mg/kg/day in dogs for 4 week and 1 year, respectively.

Reproductive Toxicology

Moxidectin was administered by oral gavage at dosages of 0, 2.5, 5, 10, and 12 mg/kg/day once daily to presumed pregnant rats from gestation day 6 through 15. Moxidectin was maternally toxic at dosages of ≥ 10 mg/kg/day as evidenced by decreased body weight, body-weight gain, and food consumption during the daily dosing period. Although not statistically significant, fetal body weights at ≥ 5 mg/kg/day tended to be decreased (≤ 6%) in comparison with controls. At ≥ 10 mg/kg/day, there were statistically significant increases in the number of fetuses with malformations and/or variations (3.1%, 4.3%, 5.5%, 7.9% and 8.4% at 0, 2.5, 5, 10, and 12 mg/kg/day, respectively). The numbers of litters with malformations and/or variations also increased (26.1%, 41.7%, 40.0%, 54.2% and 54.2% at 0, 2.5, 5, 10, and 12 mg/kg/day, respectively). These increases largely reflected increases in cleft palate and/or reversible delays in ossification that were not considered related to reduced fetal weight. It is well

established in the scientific literature that maternal toxicity can result in fetal alterations including malformations and delays in ossification.

There were no moxidectin-related effects on any other maternal or embryo-fetal parameter examined. The maternal and developmental no-toxic-effect level (NTEL) was concluded to be 5 mg/kg/day, with increased incidences of cleft palate and delayed ossification in the presence of maternal toxicity at the higher dosages of 10 and 12 mg/kg/day.

Moxidectin was also administered by oral gavage at dosages of 0, 1, 5, or 10 mg/kg/day to groups of artificially inseminated rabbits from gestation day 7 through 19. Maternal toxicity was evident at dosages ≥ 5 mg/kg/day as evidenced by decreased body weight, body-weight gain, and food consumption during the dosing period. Reduced litter size (6.2 compared with 7.1 in controls), reflective of increased early resorptions (1.2 compared with 0.2 in controls) was observed at 10 mg/kg/day. There were no other moxidectin-related effects, including malformations or other fetal alterations, observed in this study. The maternal and developmental NTELs were concluded to be 1 and 5 mg/kg/day, respectively.

The highest dose of moxidectin to be studied in Phase 2 and possibly Phase 3 clinical trials is a single, oral dose of 8 mg, which is a dosage of 0.16 mg/kg or 5.4 mg/m^2 (based on body surface area) for a 50kg human. Moxidectin was well tolerated in healthy male subjects after oral administration (liquid formulation) of single doses as high as 36 mg (24.5 mg/m^2). The developmental NTEL in both the rat and rabbit studies was 5 mg/kg/day administered in 10 and 13 consecutive, daily doses in rats and rabbits, respectively. The NTEL of 5 mg/kg is approximately 32 times the highest single dose (8 mg) to be administered to humans based on body weight, and approximately 3 times (rat) or 8 times (rabbit) based on body surface area. In the female rat, moxidectin has a half-life of 45 hours (data not available for the rabbit). Therefore, daily administration of moxidectin to pregnant rats, as was done in the teratogenicity study of moxidectin, would likely result in accumulation of the drug in the body, further increasing exposure of the fetus to the drug. Based on the dose per unit of body weight, or dose based on body surface area, the intake by a woman of a single 8 mg dose of moxidectin would be unlikely to pose a risk to the developing human fetus.

Developmental risk can also be assessed based on actual exposure of the body to moxidectin, assuming placental transfer is similar in rats and humans. In a Phase 1 clinical trial using the liquid formulation of moxidectin, a single oral dose to healthy males of 18 mg of moxidectin resulted in an area under the

concentration-time curve (AUC) value of 227 ng/day/mL, which is equivalent to 9.55 ng/hr/mL. In female rats, a single oral gavage dosage of 0.2 mg/kg of moxidectin resulted in an AUC value of 470 ng/hr/mL, which is approximately 50 times the human AUC after a single dose of 18 mg of moxidectin. Assuming linear PK in rats, the developmental NTEL of 5 mg/kg would correspond to an AUC value of approximately 11,750 ng/hr/mL, which is more than 1200 times the human AUC after a single dose of 18 mg of moxidectin. Assuming similar bioavailability of the liquid and tablet formulations of moxidectin in humans (as demonstrated in protocol 3110A1-101-EU), and based on the above exposure considerations, the oral administration of up to 8 mg of moxidectin is unlikely to pose a risk to the developing human fetus.

Prior Human Experience-Potential Risks and Benefits to Human Subjects

The results of nonclinical studies supported the development of moxidectin for the control of human onchocerciasis and the initiation of study 3110A1-100: a single-ascending dose, placebo-controlled, double-blind, safety, tolerability, and pharmacokinetic study of orally administered moxidectin in normal subjects. In this study, 38 healthy subjects were treated with single doses of 3 mg to 36 mg (approximately 50 μg/kg to 600 μg/kg). Subjects were randomized into placebo, 3 mg fasting, 9 mg fasting, 9 mg fed, 18 mg fasting, 36 mg fed and 36 mg fasting groups. Rapid absorption was seen in all dose groups. The results of the comparison of time of maximum concentration (T_{max}) between fasted and fed groups suggested that high-fat food may delay the absorption of moxidectin. Only 1 adverse event (AE) higher than grade 2 was reported in this study (enteritis due to food poisoning [grade 3] in the 36 mg fasting group) and was considered unrelated to study drug. The most common treatment emergent AEs (TEAEs) were headache (35%) and infection (29%). Only half of the headaches reported and none of the infections reported were regarded as study drug related. The reported infections, including type of infection, time of occurrence after moxidectin administration, and dose group were: upper respiratory tract infection (URTI) on Day 19 and Day 63 (same subject) and viral URTI on Day 14 in the 3 mg fasted group; toe infection on Day 52 in the 9 mg fasted group; URTI on Day 30 and Day 78 (different subjects) in the 9 mg fed group; tooth abscess on Day 47 and head cold on Day 50 and Day 66 (different subjects) in the 36 mg fasted group; cold on Day 42 and cold-like symptoms on Day 59 (same subject) in the 36 mg fed group. No SAEs were reported. Four (4) subjects discontinued from the study (1 before receiving study drug and 3 after dose administration [in the 3 mg fasting, 9 mg fed, and 36 mg fasting groups]). The withdrawal from the study was unrelated to AEs. Overall, moxidectin appears to be safe at the doses evaluated in this study.

In another study (protocol 3110A1-101-EU) the relative bioavailability of a tablet (to be used in this study) and a liquid formulation (used in the first study in healthy subjects; protocol 31101A1-100-EU) of moxidectin at the 10-mg dose strength were compared. This was an open-label, randomized, single-dose, parallel-design study conducted in 58 young healthy male subjects. Subjects' mean age (32 years, range 20 – 45 years) and weight (81 kg, range 57 – 107 kg) were similar for those receiving the tablet formulation (n = 29) and those receiving the liquid formulation (n =29). Each subject received 1 orally administered dose of moxidectin, either as five 2-mg tablets, or as a liquid. In both cases, moxidectin was administered under fasting conditions. Subjects were followed for 180 days.

During the course of the study, there were no SAEs and no subjects discontinued the study due to an AE.

A total of 36 (62.1%) subjects had TEAEs during the study, with the same number and percentage of subjects (18; 62.1%) reporting TEAEs in both the moxidectin liquid and tablet groups. The most commonly reported (by ≥ 5% of subjects) TEAEs were flu syndrome (17.2% and 20.7% in the liquid and tablet groups, respectively), headache (17.2% and 13.8% in the liquid and tablet groups, respectively), infection (13.8% and 6.9% in the liquid and tablet groups, respectively), diarrhea (10.3% in the liquid group), myalgia (6.9% in the tablet group), and dizziness (6.9% in the tablet group).

A total of 19 of 58 (32.8%) subjects reported TEAEs on or prior to day 7 post treatment (during the inpatient period) including 10 of 29 (34.5%) subjects in the group receiving moxidectin liquid and 9 of 29 (31.0%) subjects in the group receiving moxidectin tablets. The most commonly reported (by ≥ 5% of subjects) TEAEs during this period were asthenia (10.3% in the tablet group), headache (13.8% and 6.9% in the liquid and tablet groups, respectively), infection (6.9% in the liquid group), diarrhea (6.9% in the liquid group), myalgia (6.9% in the tablet group), and dizziness (6.9% in the tablet group).

A total of 22 (37.9%) subjects reported TEAEs during the outpatient period of the study (after study day 7 and up to study day 180) including 10 (34.5%) subjects in the liquid group and 12 (41.4%) subjects in the tablet group. The most commonly reported events were flu syndrome (17.2% and 20.7% in liquid and tablet groups, respectively), headache (6.9% in the tablet group), and infection (6.9% in the tablet group).

All of the TEAEs that were reported during the study were mild to moderate in intensity, and none were considered to be related to treatment. No clinically relevant abnormalities were observed in vital sign measurements, ECGs or laboratory tests during the study.

The data from the normal subject studies (protocol 3110A1-100-EU and 3110A1-101 EU) suggests that progression to a safety and tolerability study in subjects with different degrees of severity of infection with *Onchocerca volvulus* as described in this protocol is warranted. Based on the preclinical pharmacology data, moxidectin may be microfilaricidal and in addition may eliminate the reproductive capacity of the macrofilaria or be macrofilaricidal. If moxidectin has either of these effects on the macrofilaria, it has the potential to cure the disease in the trial subjects who are recruited from an area without onchocerciasis control, i.e. areas without vector control or ivermectin distribution. If moxidectin does not have either this effect on macrofilaria or a microfilaricidal effect, subjects will benefit from participating in the protocol by the nodulectomies performed, which will free them of all palpable nodules and reduce the number of macrofilaria in their bodies that can generate microfilariae.

Route of Administration, Dosage and Treatment Period

In this study, a tablet formulation will be available for both ivermectin and moxidectin. A single dose regimen was chosen for this study, as well as for the whole development program, because a longer dosing regimen is not compatible with the ultimate use of moxidectin within onchocerciasis control programs.

Moxidectin dose selection (2 mg, 4 mg and 8 mg) was based on comparison of the maximum plasma levels obtained during the first study in human subjects (protocol 3110A1-100-EU, 3 mg dose: mean 22 ng/mL range 17.1-28.5 ng/mL; 9 mg: mean 54-58 ng/mL, range 38.9 – 66.5ng/mL) with those of the approved dose of ivermectin (mean 46.6 ng/mL after a 12 mg dose, equivalent to 150 µg/kg for an 80 kg adult), and on the pre-clinical pharmacology data on the relative efficacy of moxidectin and ivermectin. The frequency and severity of AEs after ivermectin treatment are related to the amount of microfilariae killed. Most of the serious and/or severe AEs after ivermectin treatment occur within the first 72 hours after dosing. To reduce the risk associated with AEs related to microfilaria killing after moxidectin treatment, three measures have been included in this protocol: (1) the initial dose of moxidectin in subjects was chosen to be only 2 mg, (2) subjects are enrolled at each dose level sequentially by increasing severity of infection and (3) subjects are hospitalized during the first 18 days post treatment (see section 11).

Animal veterinary experience

Moxidectin Canine SR Injectable (ProHeart® 6, PH6) is a novel sustained release injectable product, consisting of 10% moxidectin in microspheres containing glyceryltristerate, developed by Fort Dodge Animal Health (FDAH) for the prevention of heartworm. PH6 has been marketed in the United States since June 2001, and is also approved for use in Canada, Italy, Japan, France, Greece, Portugal, Spain and Korea. The same formulation providing 12 months protection (ProHeart SR® 12, PH12) is marketed in Australia since October 2000. PH12 contains three times as much moxidectin as PH6.

After the launch of the product, the Center of Veterinary Medicine (CVM) of the United States Food and Drug Administration (FDA) expressed concern about the number and seriousness of adverse event reports (AERs), most of which were submitted by FDAH to FDA based on field reports from veterinarians and dog owners. On 03 Sep 2004, based on continued FDA concerns, FDAH voluntarily recalled the product from the US market. The recall prompted regulatory authorities in Canada, Japan, Europe and Australia to review the safety of PH6 and PH12, respectively, including the PH6 adverse event reports from the US. These authorities subsequently allowed continued marketing of PH6 or PH12, respectively, for canine heartworm control. Neither the US FDA, nor any of the other regulatory agencies voiced any concerns about an oral tablet formulation of moxidectin for the prevention of heartworm in dogs, nor any other moxidectin containing drugs approved for veterinary use in other species.

The initiation of this study was put on hold to thoroughly examine the PH6-related data, all moxidectin related toxicology data and the adverse event data from the human volunteer studies within the organization of each sponsor as well as with outside consultants. The conclusion of these examinations was that the PH6 related data do not suggest that the assessment of the risk for subjects in this trial needs to be changed. This conclusion is based on the following facts:

- The additional review of the existing Phase I safety data by Wyeth medical personnel, did not reveal any signals for potential safety concerns.
- The additional preclinical data on moxidectin acquired by FDAH in collaboration with Wyeth have reconfirmed that moxidectin is a very safe drug candidate.

- In May 2005, WHO consulted with independent experts on the safety data for moxidectin and Proheart 6 and their implications for the conduct of the planned study in Ghana. The experts unanimously concluded that the data on PH6 have no relevance for the evaluation of moxidectin tablets in humans and that they do not provide any scientific reason not to initiate the study in Ghana and recommended that the study be initiated as soon as possible.

- In May 2005, FDA CVM approved an injectable formulation of moxidectin for treatment of cattle (CYDECTIN®), which clearly shows that any FDA CVM concerns are related to the Proheart 6 formulation, not moxidectin.

10. TRIAL DESIGN

10.1 Description

This is a randomized, ivermectin-controlled, double-blind, single-ascending-dose, parallel design, inpatient/outpatient study of moxidectin administered to subjects of both sexes with different degrees of severity of *O. volvulus* infection. A total of 192 subjects will be enrolled in consecutive cohorts and will receive a single oral dose of moxidectin or a standard dose of ivermectin. Moxidectin is available in 2 mg TIC (tablets in capsules). Three (3) dose levels of moxidectin will be studied: 2 mg, 4 mg, and 8 mg. Therefore, the dose of moxidectin will be either 1 TIC for the 2mg, 2 TIC for the 4 mg, or 4 TIC for the 8 mg dose group.

Ivermectin will be dosed according to the approved weight based dosing schedule (see section 17.1).

To ensure blinding, moxidectin-treated and ivermectin-treated subjects may receive a number of placebo capsules so that each subject receives 4 capsules. All doses will be given after an overnight fast and breakfast will be delayed for about 2 hours.

Enrollment will be by dose and severity of infection as indicated below. The intensity of infection will be based on the mean of the densities at the iliac crests and calves. At each dose level, subjects with > 0 and < 10 microfilariae/mg of skin must have no ocular involvement. The sum of microfilariae in the two eyes must be ≤ 10 in subjects with 10-20 microfilariae/mg of skin.

- Cohort 1: 2 mg, 16 subjects with > 0 and < 10 microfilariae/mg of skin

- Cohort 2: 2 mg, 16 subjects with 10-20 microfilariae/mg of skin and the sum of microfilariae in the two eyes must be ≤ 10
- Cohort 3: 2 mg, 32 subjects with > 20 microfilariae/mg of skin with or without ocular involvement (this cohort will be enrolled in two groups of 16 subjects due to the capacity of the site)

- Cohort 4: 4 mg, 16 subjects with > 0 and < 10 microfilariae/mg of skin
- Cohort 5: 4 mg, 16 subjects mg, with 10-20 microfilariae/mg of skin and the sum of microfilariae in the two eyes must be ≤ 10
- Cohort 6: 4 mg, 32 subjects with > 20 microfilariae/mg of skin with or without ocular involvement (this cohort will be enrolled in two groups of 16 subjects due to the capacity of the site)

- Cohort 7: 8 mg, 16 subjects with > 0 and < 10 microfilariae/mg of skin
- Cohort 8: 8 mg, 16 subjects with 10-20 microfilariae/mg of skin and the sum of microfilariae in the two eyes must be ≤ 10
- Cohort 9: 8 mg, 32 subjects with > 20 microfilariae/mg of skin with or without ocular involvement (this cohort will be enrolled in two groups of 16 subjects due to the capacity of the site)

The decision to move to the next cohort within one dose group will be made by the PI based on evaluation of all blinded data obtained during the first 30 days post treatment of the previously enrolled cohort(s). The PI will inform the sponsors of this decision prior to enrollment of the next cohort. If either of the sponsors disagrees, cohort progression will be put on hold until the disagreement is resolved.

The decision to move to the next higher dose group, will be made based on blinded review of the safety and microfilaricidal data obtained during the first 30 days post treatment of all previously enrolled cohorts. The PI will submit a recommendation to proceed or not to proceed after a detailed examination of the blinded data. The Wyeth Medical Monitor, the WHO Clinical Monitor and the WHO Project Manager will conduct their separate review of the blinded data. Wyeth will include a representative of its safety surveillance group in addition to the medical monitor for purposes of review. WHO may include its Clinical Coordinator in the review.

The Project Review Team (PRT), consisting of Wyeth and WHO/TDR reviewers and the PI, will discuss the blinded data and formulate a recommendation to progress or not. The recommendation will be provided together with the rationale for this recommendation to the Clinical Expert Review Team (CERT)

consisting of three clinical experts (not otherwise associated with the conduct of the study) who have access to the randomized dose assignments in patient envelopes and have reviewed the data in parallel with the PRT. The CERT will initially review the data in a blinded fashion. They can make their recommendation on dose progression based on blinded data. If they need to unblind, they may do so for individual patient(s), or they may unblind the entire cohort. In either case, they will inform the PRT of their recommendation on dose progression without disclosing if the decision was made on blinded or unblinded data. If there is a difference of opinion within the PRT regarding the dose progression recommendation, the different recommendations and their rationale will be provided to the CERT. The CERT will provide their recommendation to PRT. If there is not unanimous agreement among the CERT, they will provide their differing recommendations and their rationale. If there is not unanimous agreement on proceeding to the next dosing cohort between the PRT, and a majority of the CERT, the data will be discussed in a blinded fashion. If no agreement can be reached, unblinded data will be made available to the PRT and discussed. If upon discussion of the unblinded data there is no unanimous agreement to proceed to the next higher dose group, dose progression will not occur.

The diagram below provides an overview of the process from enrollment of the first cohort (2 mg) to decision to progress to the 4 mg dose as well as treatment and study participation for each subject. The same process will be followed for the cohorts in the 4 mg dose group and the decision to move to the 8 mg dose.

Enrollment Process and Dose Progression Decision Diagram

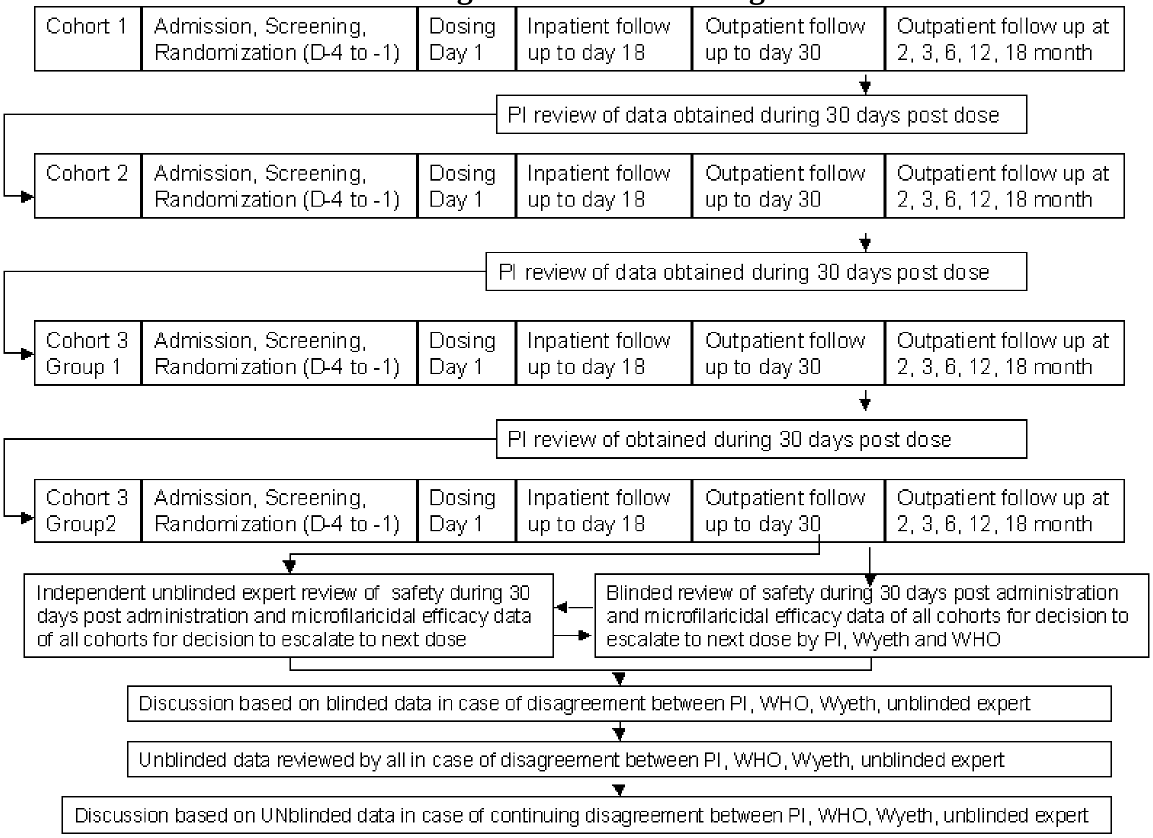

A schematic presentation of procedures is provided in the subject flowchart (section 9).

11. STUDY OBJECTIVES

11.1 Primary

The primary objective of this study is to determine the safety and tolerability of orally administered moxidectin in subjects with *O. volvulus* infection, as measured by the incidence of clinical AEs and clinically significant laboratory test results.

11.2 Secondary

The secondary objectives of this study are:

1. To determine doses that effectively eliminate microfilariae and prevent their reaccumulation in the skin as measured by the skin microfilarial loads at day 8, 1 month, 2 months, 3 months, 6 months, 12 months and 18 months after treatment.
2. To determine the viability and fertility of adult worms at 18 months.
3. To assess the pharmacokinetics of moxidectin in adult male and female subjects.

12. SELECTION OF STUDY POPULATION

This study will be conducted in otherwise healthy subjects with *O. volvulus* infection from the OCRC study area in the basin of River Tordzi, located in the forest area of south-eastern Ghana and within a 2 to 2⅔ hours journey from Hohoe, the location of the study site. The Onchocerciasis Control Programme (OCP) had established in 1988, during the southern extension of the program, that transmission of onchocerciasis in the area involved forest vectors exclusively and hence no control activities were instituted. The area was also not included in the ivermectin distribution program launched by the Directorate for Onchocerciasis Control of the Ministry of Health, as the area is largely hypoendemic for onchocerciasis. Extensive clinical, ophthalmologic and parasitological surveys carried out by the OCRC showed that morbidity due to onchocerciasis was low. The vast majority of adults had no skin or ocular lesions. Where ocular lesions were found they were mainly non-onchocercal. This was in line with the observation during initial contact with the area that most villages had never heard of onchocerciasis, the *Simulium* fly, or of ivermectin and very few had attended any health institution for skin or ocular problems or for the excision of nodules. Thus the OCRC only offered individualized treatment with ivermectin to those found during surveys to have clinically significant onchocercal lesions. The communities were informed of the existence elsewhere of mass treatment with ivermectin and the availability of the drug at nearby centers.

Subjects will be members of a “grand cohort” who were previously prescreened in their villages and the hospital and who have been dormant, pending further testing and enrollment in the appropriate clinical trial.

The ‘grand cohort’ was identified previously in a step-wise process consisting of (1) identification of potentially suitable villages based on entomological data collected by the Onchocerciasis Control Programme, and/or indirectly via OCRC out-patients with high skin microfilaria counts from these villages, and/or known breeding sites of *Simulium* close to these village and/or village residents reporting a high prevalence of onchocerciasis and *Simulium* bite rates, (2) an initial meeting with the village chief, elders and opinion leaders during which the information provided in a ‘generic information sheet’ (e.g.

background on onchocerciasis, purpose of the OCRC, purpose and procedures for screening, general purpose and procedures for three types of studies (clinical drug efficacy and safety study, 'ex vivo' study of the efficacy of drugs on the macrofilariae, PK study) is presented and discussed, (3) a meeting with the community in which the same information was presented and discussed, after the village chief, elders and opinion leaders had provided their agreement to such a meeting, (4) a village survey, after the community had agreed to that, (5) a screen at Onchocerciasis Chemotherapy Research Centre in the Government Hospital at Hohoe for those subjects that, based on the village survey, had *O. volvulus* infection, were 18-60 years old, had no obvious exclusion factors, and had agreed to the hospital screen. The results of the screening were communicated to the subjects. Subjects who could potentially qualify for a clinical trial form the 'grand cohort' from which subjects for future studies will be recruited. Informed consent was obtained at all stages of contact with the communities listed above. The "generic" informed consent form was the basis for the transfer of information and the subsequent discussions. The consent prior to the hospital-screening phase was verbal and involved thousands of people in the initial stages and a few hundred in the hospital-screening phase.

For recruitment for this study, subjects on the 'grand cohort' list will be asked to come for screening per cohort, based on position of their name on the list, gender and microfilaria counts. If a subject asked to come for screening for a particular cohort meets all inclusion criteria and has none of the exclusion criteria, but has a microfilaria count too high or too low for that cohort, they will be offered the choice between (1) enrollment into the next cohort for which they qualify based on their microfilaria count or (2) nodulectomy and ivermectin treatment. If their microfilaria count is too low or too high for any cohort planned in the future, they will be offered nodulectomy and ivermectin treatment. The same will happen, if they do not meet one of the other inclusion criteria or meet an exclusion criterion (see figure below) or if 16 subjects higher on the grand cohort list have already qualified for the study.

All members of the grand cohort who are invited for this study participate in a written and signed or thumb printed informed consent process. During this process, the PI or sub-investigator informs the subjects of all the elements specific to the study. An information sheet that is specific to the study and is available in English and the local language replaces the generic information sheet. Adequate time is then allowed, as during the earlier discussion, for the subject to ask questions and make a voluntary decision. No protocol-specific procedures, including transport to the study site and screening, will be performed until the subject has signed or thumb printed and dated an ethics committee (EC) approved informed consent form. The form will be witnessed by a member of the community (impartial witness) and

countersigned by the investigator. A copy will be given to the subject. The study begins at the time the first subject is admitted to the hospital for the study. Subjects must meet all the inclusion and none of the exclusion criteria to be enrolled in the study.

OCRC informs each subject individually about the details of the study, which concern him/her from the time of enrollment to completion. This includes AEs, lab results etc. and the final status. If there are any outstanding medical issues, further advice is given.

Following completion of the study, the PI and his staff will seek an appointment with the chiefs and the elders and discuss the overall findings and conclusions of the study with them. Subsequently, study results and conclusions are presented and discussed at a village durbar. The discussions with chiefs and elders as well as those at the village durbar include the implications of the study results for future studies on moxidectin as well as other research.

Consent Process Diagram

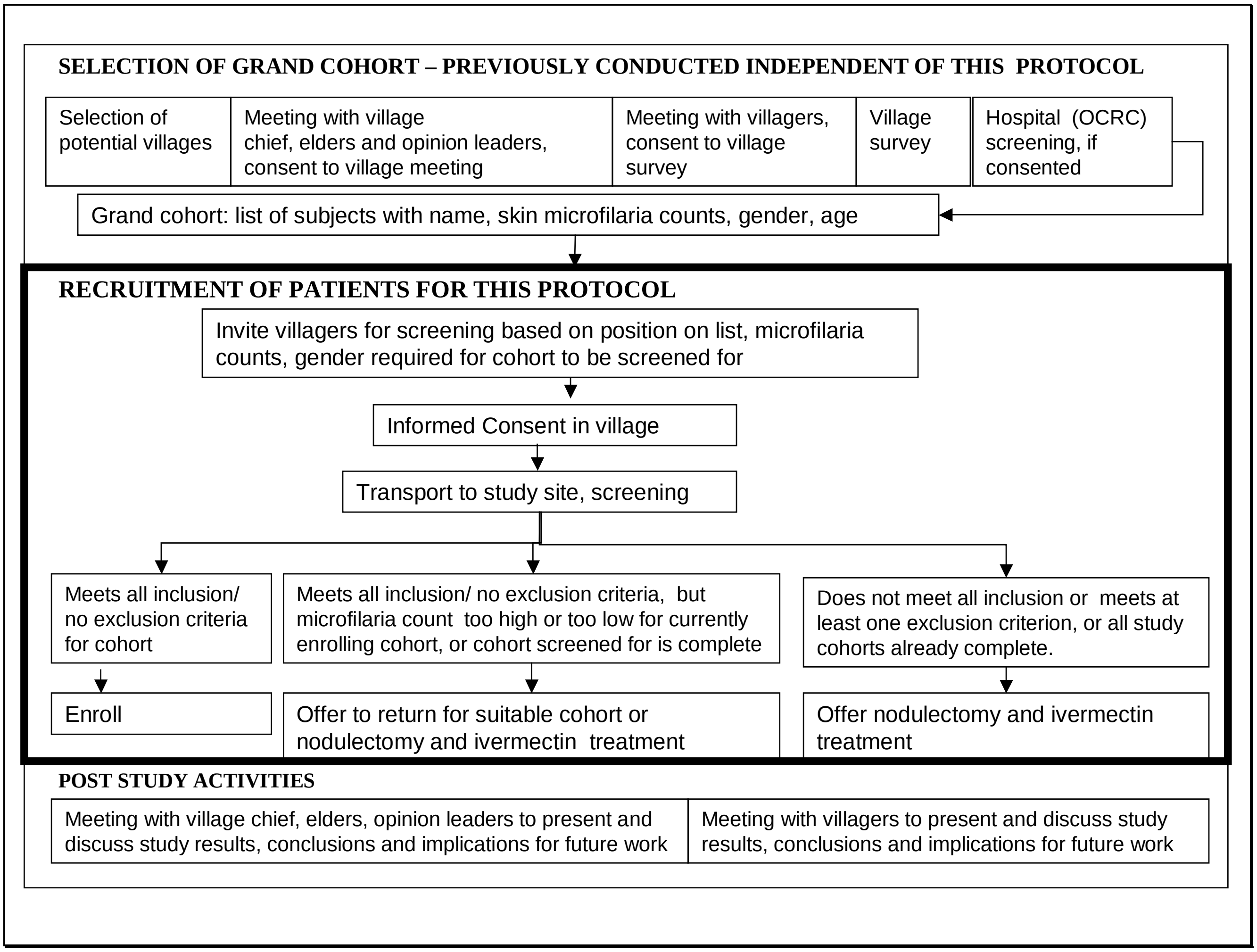

12.1 Inclusion Criteria

Men and women in good general health, with *O. volvulus* infection and:

a) Written, signed (or thumb-printed), and dated informed consent

b) Aged 18 to 60 years, inclusive

c) Body weight ≥ 40 kg for women and ≥ 45 kg for men

d) Nonpregnant, nonbreastfeeding women. Women of child-bearing potential must agree to use birth control during the first 150 days after treatment.

e) Healthy, as determined by a physician on the basis of a physical examination, ECG, and a thorough review of the medical history and clinical laboratory results

f) Adequate hematologic, renal, and hepatic function, defined as:

1) White blood cell (WBC) count ≥ 2,800 and ≤ 11,300 cells/mL

2) Hemoglobin: ≥ 11.0 g/dL for men and ≥ 10.0 g/dL for women

3) Platelet count: ≥ 110,000 mm3

4) Serum creatinine: ≤ 1.25 x upper limit of normal (ULN)

5) Total bilirubin: ≤ 1.25 x ULN

6) AST/SGOT: ≤ 1.25 x ULN

7) AP: ≤ 1.25 x ULN

8) Prothrombin time WNL

9) Urinalysis WNL

g) Skin microfilarial density within the required range for the cohort

12.2 Exclusion Criteria

a) Participation in any studies other than purely observational ones, within 4 weeks before test article administration.

b) Any vaccination within 4 weeks before test article administration

c) Acute infection requiring therapy within the last 10 days before test article administration

d) Administration of any medication (with the exception of medication required to treat any reactions during the screening fluorescein angiography (chlorpheniramine) or paracetamol) or herbal preparation within 10 days prior to test article administration or any condition currently requiring regular medication

e) Clinically significant ECG abnormalities or history of cardiac abnormality

f) Past or current history of neurological or neuropsychiatric disease or epilepsy

g) Subjects with orthostatic hypotension at the screening evaluation

h) History of drug or alcohol abuse or regular use of ≥ 3 cigarettes per day

i) Use of alcohol or other drugs of abuse within 72 hours before test article administration

j) Any condition, in the investigator's opinion, that places the subject at undue risk

k) Subjects who have donated blood within 8 weeks before study entry

l) Subjects with ocular onchocerciasis in cohorts intended to enroll subjects with mild infection. Ocular onchocerciasis is defined by presence of live or dead microfilariae, onchocercal punctate opacities, onchocercal lesions of the posterior segment or lesions that mimic those seen in onchocerciasis.

m) Subjects with hyperreactive onchodermatitis

n) Antifilarial therapy within the previous 5 years

o) Coincidental infection with *Loa Loa*

p) Female subjects of childbearing potential with a contraindication to DMPA if not on Norplant

q) Any other condition which the investigator feels would exclude the subject from the study

Subjects who are excluded from the study or subjects who are withdrawn from the study for medical reasons will be advised where to seek assistance.

12.3 Number of Subjects

One hundred and ninety-two (192) subjects including both sexes, aged 18 to 60 years are expected to enroll in this study. There will be 9 cohorts. The first 2 cohorts at each dose level will comprise 16 subjects while the third cohort will comprise 32 subjects, which will be enrolled in 2 successive groups of 16. The study will aim to enroll equal numbers of men and women at each dose level and intensity of infection.

12.4 Screen Failures

Subjects who sign an informed consent form and fail to meet the inclusion and/or exclusion criteria are defined as screen failures. All screen failures should be recorded in the screening and enrollment log. A copy of the log should be retained in the investigator's study files. No case report forms (CRFs) will be completed for screen failures. Subjects who are excluded on medical grounds will be advised where to seek assistance. All screen failures will be offered nodulectomy and ivermectin treatment.

13. PRIOR TREATMENT

Reasonable efforts will be made to determine all relevant treatment received by the subject within 10 days prior to study drug administration. All relevant information, including the names of any procedures, drugs, or herbal or dietary supplements used will be recorded in the subject's CRF. Include the name of the procedure or drug and other information required on the CRF. Subjects must not have taken any medication, prescription or over-the-counter (OTC), within 10 days before administration of the study drug (with the exception of medication required to treat any reactions during the screening fluorescein angiography (chlorpheniramine) or paracetamol). Paracetamol 1.0g as required (maximum 3.0g per day) will be given to subjects with painful conditions. Subjects are transported on a 2½-hour journey on bumpy roads from their villages to the centre. Inevitably, diffuse aches and pains that require relief result. Additionally, headaches often occur after maximal papillary dilation for colour fundus photography and fluorescein angiography using phenylephrine hydrochloride 2.5% followed by tropicamide 1%. Subjects must also have discontinued use of herbal and dietary supplements at least 10 days before study drug administration.

14. CONCOMITANT TREATMENT

14.1 Additional Required Drugs

Depo-medroxyprogesterone acetate (DMPA), 150 mg, will be administered twice to all women of childbearing potential. The first dose will be given on study day 11 and the second dose will be at month 2 (±1 week). Women already receiving a parenterally administered contraceptive ~~who have contraceptive protection by the Norplant system~~ will be excluded from the protocol-defined DMPA injections schedule. Dosing will be collected on the CRF.

14.2 Permitted Treatment

All medication given after admission to the hospital will be documented in the CRF.

Chlorpheniramine 4mg will be administered 30 minutes before injection to subjects who have reacted to a previous dose of fluorescein sodium. Adverse reactions to fluorescein sodium occur in 18 to 38% of subjects. They manifest as nausea, vomiting, abdominal pain, itching urticaria, facial or lip swelling, chills and dry cough. Pretreatment with chlorpheniramine ameliorates the adverse events in most, but not all subjects

14.3 Prohibited Treatment

Smoking, the ingestion of alcoholic beverages, grapefruit, or grapefruit juice, and the use of non-prescribed drugs will not be permitted during the 18 day admission period. Treatment with other anthelmintic agents including ivermectin will be prohibited for the duration of the study. If anthelmintic treatment has been taken for any reason, the subject will be withdrawn from the study.

15. TREATMENT OF SUBJECTS

15.1 Procedures

See flowchart(s) for the procedures to be performed at each visit.

15.1.1 Days -4 to -2 (Visit 1)

Informed consent will be obtained in the village prior to transport of potential study participants to the study centre and before any study-related procedures are performed. Informed consent may be obtained prior to day -4.~~admission. Subjects will be Once admitted to the OCRC,~~ T~~at the beginning of this period and t~~he following procedures and evaluations will be performed:

- ~~Informed consent will be obtained prior to transport of the potential study participants from the village to the study center and before any study-related procedures are performed.~~
- Demographics (age, tribal origin, gender), medical history, medication history (all prescription and over-the-counter medications taken within 10 days prior to study drug administration) will be recorded.
- A complete physical examination, including the subject's height and weight, and a review of body systems, will be performed by a licensed physician.
- A twelve-lead ECG will be performed (See section 16.3.3, Special Methods, Electrocardiograms).
- An ocular examination, including fundus colour photography and fluorescein angiography for all subjects.
- Vital sign measurements (supine pulse rate and blood pressure [see section 16.3.2 Vital signs], repeated after standing for at least 2 minutes; respiratory rate, and oral temperature) will be taken up to 12 times throughout this screening period.
- An evaluation of any AEs will be conducted during waking hours as other activities permit.
- A complete laboratory evaluation including hematology with prothrombin time, G6PD, hemoglobin electrophoresis and nucleopore filtration for microfilariae, serum chemistry (first morning fasted values) and a routine urinalysis and nucleopore filtration for microfilariae (see section 16.3.5, Special Methods, Laboratory Evaluations/Specimen Collection).
- Skin snips will be taken from both iliac crests and calves to determine microfilarial density.

15.1.2 Day -1 (Visit 2)

On day -1, the final list of study participants will be drawn up.

- A pregnancy test (serum β-hCG) will be performed for all women of childbearing potential and the results known prior to study drug administration.
- Vital sign measurements (supine pulse rate and blood pressure [see section 16.3.2 Vital signs], repeated after standing for at least 2 minutes; respiratory rate, and oral temperature).
- Adverse events assessment
- Randomization (once all screening data have been completed, and the subject meets the inclusion/exclusion criteria, a subject will be randomly assigned to receive moxidectin or ivermectin therapy).
- Packaging, dispensing and labeling of test articles. These will be locked up until the morning of day 1.

15.1.3 Day 1 (Visit 3)

On day 1, the following evaluations/procedures will be performed:

- A pre-treatment blood sample for PK analysis (obtained within 2 hours before drug administration)
- Test articles administration at 0700 ±1hours
- Vital sign measurements (supine pulse rate and blood pressure [see section 16.3.2 Vital signs], repeated after standing for at least 2 minutes; respiratory rate, and oral temperature) will be taken approximately 3 times in the 24 hour period.
- Adverse events assessment
- Blood samples for PK analysis at 1, 2, 4, and 8 hours (each time point ±15 minutes) post study drug administration
- Interim physical examination including weight
- Twelve-lead ECG approximately 4 hours after dose administration
- Laboratory evaluations including hematology with prothrombin time and nucleopore filtration for microfilariae, serum chemistries (first morning fasted values), and routine urinalysis and nucleopore filtration for microfilariae.

15.1.4 Day 2 (Visit 4)

On day 2, the following evaluations/procedures will be performed:

- Vital sign measurements (supine pulse rate and blood pressure [see section 16.3.2 Vital signs], repeated after standing for at least 2 minutes; respiratory rate, and oral temperature) will be taken approximately 5 times in the 24 hour period.
- Adverse events assessment
- Interim physical examination including weight
- Twelve-lead ECG
- Laboratory evaluations including hematology with prothrombin time and nucleopore filtration for microfilariae, serum chemistries (first morning fasted values), and routine urinalysis and nucleopore filtration for microfilariae.
- Blood sample for 24 hour (±15 min) post study drug administration for PK analysis.

15.1.5 Day 3 (Visit 5)

On day 3, the following evaluations/procedures will be performed:

- Vital sign measurements (supine pulse rate and blood pressure [see section 16.3.2 Vital signs], repeated after standing for at least 2 minutes; respiratory rate, and oral temperature) will be taken approximately 5 times in the 24 hour period.
- Adverse events assessment
- Interim physical examination including weight
- Twelve-lead ECG
- Ocular examination for all subjects, including fundus colour photography and fluorescein angiography (Day 3 or 4)

15.1.6 Day 4 (Visit 6)

On day 4, the following evaluations/procedures will be performed:

- Vital sign measurements (supine pulse rate and blood pressure [see section 16.3.2 Vital signs], repeated after standing for at least 2 minutes; respiratory rate, and oral temperature) will be taken approximately 5 times in the 24 hour period.
- Adverse events assessment
- Interim physical examination including weight
- Blood sample for 72 hour (±15 min) post study drug administration for PK analysis.
- Laboratory evaluations including hematology with prothrombin time and nucleopore filtration for microfilariae, serum chemistries (first morning fasted values), and routine urinalysis and nucleopore filtration for microfilariae.

15.1.7 Day 5 (Visit 7)

On day 5, the following evaluations/procedures will be performed:

- Vital sign measurements (supine pulse rate and blood pressure [see section 16.3.2 Vital signs], repeated after standing for at least 2 minutes; respiratory rate, and oral temperature) will be taken approximately 5 times in the 24 hour period.
- Adverse events assessment
- Interim physical examination including weight

15.1.8 Day 6 (Visit 8)

On day 6, the following evaluations/procedures will be performed:

- Vital sign measurements (supine pulse rate and blood pressure [see section 16.3.2 Vital signs], repeated after standing for at least 2 minutes; respiratory rate, and oral temperature) will be taken approximately 5 times in the 24 hour period.
- Adverse events assessment
- Interim physical examination including weight

15.1.9 Day 7 (Visit 9)

On day 7, the following evaluations/procedures will be performed:

- Vital sign measurements (supine pulse rate and blood pressure [see section 16.3.2 Vital signs], repeated after standing for at least 2 minutes; respiratory rate, and oral temperature) will be taken approximately 5 times in the 24 hour period.
- Adverse events assessment
- Interim physical examination including weight
- Ocular examination for all subjects, including fundus colour photography and fluorescein angiography (Day 7 or 8)

15.1.10 Day 8 (Visit 10)

On day 8, the following evaluations/procedures will be performed:

- Vital sign measurements (supine pulse rate and blood pressure [see section 16.3.2 Vital signs], repeated after standing for at least 2 minutes; respiratory rate, and oral temperature) will be taken approximately 5 times in the 24 hour period.
- Adverse events assessment

- Twelve lead ECG
- Interim physical examination including weight
- Blood sample for Day 8 (±1 hour) post study drug administration for PK analysis.
- Laboratory evaluations including hematology with prothrombin time and nucleopore filtration for microfilariae, serum chemistries (first morning fasted values), and routine urinalysis and nucleopore filtration for microfilariae.
- Skin snips

15.1.11Day 9 (Visit 11)

On day 9, the following evaluations/procedures will be performed:

- Vital sign measurements (supine pulse rate and blood pressure [see section 16.3.2 Vital signs]; respiratory rate, and oral temperature) will be taken 2 times per day.
- Adverse events assessment
- Interim physical examination including weight

15.1.12 Day 10 (Visit 12)

On day 10, the following evaluations/procedures will be performed:

- Vital sign measurements (supine pulse rate and blood pressure [see section 16.3.2 Vital signs], respiratory rate, and oral temperature) will be taken 2 times per day.
- Adverse events assessment
- Interim physical examination including weight

15.1.13 Day 11 (Visit 13)

On day 11, the following evaluations/procedures will be performed:

- Vital sign measurements (supine pulse rate and blood pressure [see section 16.3.2 Vital signs], respiratory rate, and oral temperature) will be taken 2 times per day.
- Adverse events assessment
- Interim physical examination including weight
- DMPA injection to women of child bearing potential (except those already receiving a parenterally administered contraceptive~~protected by the Norplant system~~)

15.1.14 Day 12 (Visit 14)

On day 12, the following evaluations/procedures will be performed:

- Vital sign measurements (supine pulse rate and blood pressure [see section 16.3.2 Vital signs], respiratory rate, and oral temperature) will be taken 2 times per day.
- Adverse events assessment
- Interim physical examination including weight

15.1.15 Day 13 (Visit 15)

On day 13, the following evaluations/procedures will be performed:

- Vital sign measurements (supine pulse rate and blood pressure [see section 16.3.2 Vital signs], respiratory rate, and oral temperature) will be taken 2 times per day.
- Adverse events assessment
- Interim physical examination including weight
- Laboratory evaluations including hematology with prothrombin time and nucleopore filtration for microfilariae, serum chemistries (first morning fasted values), and routine urinalysis and nucleopore filtration for microfilariae.
- Blood sample for Day 13 (±1 hour) post study drug administration for PK analysis

15.1.16 Day 14 (Visit 16)

On day 14, the following evaluations/procedures will be performed:

- Vital sign measurements (supine pulse rate and blood pressure [see section 16.3.2 Vital signs], respiratory rate, and oral temperature) will be taken 2 times per day.
- Adverse events assessment
- Interim physical examination including weight
- Ocular examination for all subjects, including fundus colour photography and fluorescein angiography (Day 14 or 15)

15.1.17 Day 15 (Visit 17)

On day 15, the following evaluations/procedures will be performed:

- Vital sign measurements (supine pulse rate and blood pressure [see section 16.3.2 Vital signs], respiratory rate, and oral temperature) will be taken 2 times per day.
- Adverse events assessment

- Interim physical examination including weight

15.1.18Day 16 (Visit 18)

On day 16, the following evaluations/procedures will be performed:

- Vital sign measurements (supine pulse rate and blood pressure [see section 16.3.2 Vital signs], respiratory rate, and oral temperature) will be taken 2 times per day.
- Adverse events assessment
- Interim physical examination including weight

15.1.19 Day 17 (Visit 19)

On day 17, the following evaluations/procedures will be performed:

- Vital sign measurements (supine pulse rate and blood pressure [see section 16.3.2 Vital signs], respiratory rate, and oral temperature) will be taken 2 times per day.
- Adverse events assessment
- Interim physical examination including weight

15.1.20 Day 18 (Visit 20)

On day 18, the following evaluations/procedures will be performed. Subjects will be discharged after all procedures are completed.

- Vital sign measurements (supine pulse rate and blood pressure [see section 16.3.2 Vital signs], respiratory rate, and oral temperature) will be taken 1 time prior to discharge.
- Adverse events assessment
- Laboratory evaluations including hematology with prothrombin time and nucleopore filtration for microfilariae, serum chemistries (first morning fasted values), and routine urinalysis and nucleopore filtration for microfilariae
- Blood sample for Day 18 (±1 hour) post study drug administration for PK analysis.

15.1.21 1-month (Visit 21)

At 1 month (±1 week), subjects will return to the center for an outpatient visit. They will be questioned about their overall health, including any changes in health status and the indication for use of any medication. The following evaluations/procedures will be performed:

- Vital sign measurements (supine blood pressure and heart rate [see section 16.3.2 Vital signs], respiratory rate, and oral temperature)

- Adverse events assessment
- Physical examination including weight
- Laboratory evaluations including hematology with prothrombin time and nucleopore filtration for microfilariae, serum chemistries (first morning fasted values), and routine urinalysis and nucleopore filtration for microfilariae
- Skin snips
- Blood sample for 1 month (±1 week) post study drug administration for PK analysis.
- Ocular examination for all subjects, including fundus colour photography and fluorescein angiography

15.1.22 2-Months (Visit 22)

At 2 months (±1 week), subjects will return to the center for an outpatient visit. They will be questioned about their overall health, including any changes in health status and the indication for use of any medication. The following evaluations/procedures will be performed:

- Vital sign measurements (supine blood pressure and heart rate [see section 16.3.2 Vital signs], respiratory rate, and oral temperature)
- Adverse events assessment
- Physical examination including weight
- Laboratory evaluations including hematology with prothrombin time and nucleopore filtration for microfilariae, serum chemistries (first morning fasted values), and routine urinalysis and nucleopore filtration for microfilariae
- Skin snips
- Blood sample for 2 months (±1 week) post study drug administration for PK analysis.
- Ocular examination for all subjects, including fundus colour photography and fluorescein angiography.
- DMPA injection to women of child bearing potential (except those already receiving a parenterally administered contraceptive~~protected by the Norplant system~~)

15.1.23 3-Months (Visit 23)

At 3 months (±1 week), subjects will return to the center for an outpatient visit. They will be questioned about their overall health, including any changes in health status and the indication for use of any medication. The following evaluations/procedures will be performed:

- Vital sign measurements (supine blood pressure and heart rate[see section 16.3.2 Vital signs], respiratory rate, and oral temperature)
- Adverse events assessment
- Physical examination including weight
- Laboratory evaluations including hematology with prothrombin time and nucleopore filtration for microfilariae, serum chemistries (first morning fasted values), and routine urinalysis and nucleopore filtration for microfilariae
- Skin snips
- Blood sample for 3 months (±1 week) post study drug administration for PK analysis.
- Ocular examination for all subjects, including fundus colour photography and fluorescein angiography.

15.1.24 6-Months (Visit 24)

At 6 months (±1 month), subjects will return to the center for an outpatient visit. They will be questioned about their overall health, including any changes in health status and the indication for use of any medication. The following evaluations/procedures will be performed:

- Vital sign measurements (supine blood pressure and heart rate [see section 16.3.2 Vital signs], respiratory rate, and oral temperature)
- Adverse events assessment
- Physical examination including weight
- Laboratory evaluations including hematology with prothrombin time and nucleopore filtration for microfilariae, serum chemistries (first morning fasted values), and routine urinalysis and nucleopore filtration for microfilariae.
- Skin snips
- Ocular examination for all subjects. Colour fundus photography and fluorescein angiography only for subjects with lesions or visual defects determined during ocular examination or previous fundus color photography or fluorescein angiography.
- Blood sample for 6 months (±1 month) post study drug administration for PK analysis.
- Blood and urine samples will be filtered and examined for microfilariae and ultrasonography of all located nodules will be performed for the WHO substudies.

15.1.25 12-Months (Visit 25)

At 12 months (± 1 month), subjects will return to the center for an outpatient visit. They will be questioned about their overall health, including any changes in health status and the indication for use of any medication. The following evaluations/procedures will be performed:

- Vital sign measurements (supine blood pressure and heart rate [see section 16.3.2 Vital signs], respiratory rate, and oral temperature)
- Adverse events assessment
- Physical examination including weight
- Laboratory evaluations including hematology with prothrombin time and nucleopore filtration for microfilariae, serum chemistries (first morning fasted values), and routine urinalysis and nucleopore filtration for microfilariae
- Skin snips
- Ocular examination for all subjects. Colour fundus photography and fluorescein angiography only for subjects with lesions or visual defect determined during ocular examination or previous fundus color photography or fluorescein angiography.
- Blood sample for 12 months (±1 week) post study drug administration for PK analysis.

15.1.26 18-months (Visit 26)

At month 18 (± 1 month), subjects will return to the center for an outpatient visit. They will be questioned about their overall health, including any changes in health status and the indication for use of any medication. The following evaluations/procedures will be performed:

- Vital sign measurements (supine blood pressure and heart rate [see section 16.3.2 Vital signs], respiratory rate, and oral temperature)
- Adverse events assessment
- Physical examination including weight
- Laboratory evaluations including hematology with prothrombin time and nucleopore filtration for microfilariae, serum chemistries (first morning fasted values), and routine urinalysis and nucleopore filtration for microfilariae
- Skin snips
- Ocular examination for all subjects. Color fundus photography and fluorescein angiography only for subjects with lesions or visual defect determined during ocular examination or previous fundus color photography or fluorescein angiography.
- Nodulectomy

- All subjects who have skin microfilariae will be treated with the approved dosage of ivermectin.

15.2 Discontinuation and Withdrawal of Subjects

- Subjects who withdraw their consent before administration of the test article will be replaced.
- Subjects cannot be withdrawn from therapy because this study involves only a single dose administration.
- A subject can be withdrawn from the study after test article administration if continued follow up jeopardizes the subject's health in the judgment of the principal investigator (e.g. an illness that contra-indicates follow up visits).
- A subject who terminates participation or is withdrawn from the study by the PI after test article administration will be considered as having discontinued from the study and will not be replaced unless the number exceeds 2 subjects in any 16 subject group and the terminations occur before day 7.
- PK data will not be collected on subjects who replace those who have withdrawn post drug administration.
- Replacement subjects will be assigned to the same cohort and treatment as the subjects leaving the study and will need to complete safety evaluations at 1 month (± 1 week) before subsequent cohorts are enrolled.
- Replacement subject numbers will be the number of the subject that they replace plus 10000. Subject numbers for the 9 cohorts without replacements are from 101-1216, replacements would be assigned numbers 10101-11216.

Subjects manifesting serious medical illnesses should be discussed with the clinical coordinator and medical monitor(s). The PI will be responsible for any decision regarding the subject's discontinuation from the study.

Subjects that terminate before the end of the study should have the procedures performed that are indicated for the 18-month visit on the study flow chart. Nodulectomy will be performed if the subject agrees but the results will not be collected on the CRF. A reasonable effort will be made to ascertain the reasons for withdrawal from the study or failure to return and these will be recorded in the CRF. This protocol only requires administration of one dose of study drug; therefore, subjects with AEs will not have to be withdrawn since no further study drug will be administered.

The administration of the test article(s) may be delayed for up to 2 days to permit all predose examinations to be performed on ~~replacement~~ subjects.

15.3 Special Methods

15.3.1 Physical Examinations and Medical History

A complete physical examination will be performed at baseline, and at 1, 2, 3, 6, 12, and 18 months. These will include height (cm, on admission only), weight (kg), vital signs, supine pulse rate and blood pressure, respiratory rate, and oral temperature, and a complete review of body systems. A summary of the dermatologic findings including nodules and onchocercal skin lesions will be entered in the CRFs.

Medical history will be obtained at baseline and will include a history of both systemic and ocular symptoms. These symptoms will be monitored for any changes in severity that could be attributed to test drug administration.

Interim physical examinations conducted during the admission period will include weight (kg), vital signs, and a review of body systems to determine whether there have been any significant changes since the previous examination.

At each post-discharge follow-up visit the subject will be questioned regarding any recent illness or changes in health status, and any medication taken and for what indication. All findings will be recorded, any significant changes investigated, and relationship to test drug administration defined.

15.3.2 Vital Signs

Vital signs including pulse rate, blood pressure, respiratory rate, and oral temperature (centigrade) will be measured after the subject has been in a supine position for at least 5 minutes. During the screening period and on Day 1 to Day 8, pulse rate and blood pressure measurements will be repeated after the subject has been standing for 2 minutes. Measurements will be taken up to 12 times during the 4 day screening period, approximately 3 times per day on day 1, 5 times per day on day 2 to day 8, and twice a day for the remainder of the inpatient period. Vital signs measurements including pulse rate, supine blood pressure, respiratory rate, and oral temperature (centigrade) will also be made at each outpatient visit.

15.3.3 Electrocardiograms

Twelve-lead ECGs will be performed during baseline and on day 1 (approximately 4 hours following test article administration) and on days 2, 3, and 8. A physician experienced in evaluating ECGs will provide the interpretations of all ECGs. This should include the rate, rhythm, length of the PR, QRS and Q-T intervals, and any abnormalities noted. The results will be recorded in the CRFs. Additional ECGs will be performed at the discretion of the investigator, or where clinically indicated.

15.3.4 Ocular Examination

Ocular examinations will include examination of visual acuity, visual fields using a calibrated Goldmann perimeter, color vision, external ocular structures, ocular mobility and pupillary reflex. The anterior segment will be examined with a Haag-Streit 900 slit-lamp. Microfilariae in the anterior chamber will be counted after head-down positioning for 5 minutes. Living and dead microfilariae in the cornea and punctate opacities will be counted. Intraocular pressure will be measured and the dilated fundus examined by direct and indirect ophthalmoscopy. Color fundus photography and fluorescein angiography (using 20% fluorescein sodium) will be done on all subjects before dosage and at days 3 or 4, 7 or 8, 14 or 15, and at 1 and 3 months and will not be repeated unless lesions or visual defects are demonstrated during ocular examination or previous fundus color photography or fluorescein angiography. The color photographs and fluorescein angiograms will be retained at the study site. Descriptive abnormalities will be recorded on the case report forms.

15.3.5 Laboratory Evaluations/Specimen Collection

Hematologic studies, including prothrombin time, a complete blood cell count (CBC), including hematocrit, hemoglobin, WBC with differential, and platelet count, will be obtained. A G6PD and hemoglobin electrophoresis will also be obtained with the screening CBC. Blood will be filtered through a nucleopore membrane, stained with Giemsa and examined for microfilariae.

Serum chemistry tests will be performed, including sodium, potassium, chloride, bicarbonate, glucose, total protein, albumin, urea, creatinine, alkaline phosphatase, lactic dehydrogenase (LDH), total bilirubin, gamma-glutamyl transpeptidase (GGT), aspartate aminotransferase (AST/SGOT), and alanine aminotransferase (ALT/SGPT).

Routine urinalysis, using a 10-parameter dipstick (specific gravity, pH, protein, glucose, ketones, blood, bilirubin, urobilinogen, nitrite, leukocyte esterase) and microscopic evaluation will be performed. Urine will be filtered through a nucleopore membrane, stained with Giemsa and examined for microfilariae.

All specimens for hematologic and serum chemistry tests will be obtained with subjects in the fasting state.

A pregnancy test (serum β-hCG) will be performed at baseline on all women of childbearing potential.

Blood samples for PK analysis for moxidectin will be collected in 10 mL collection tubes containing heparin as an anticoagulant. The plasma will be processed and separated into 2 tubes according to ATTACHMENT 2. ~~One set of~~All samples will be sent to the bioanalytical laboratory for analyses in the following manner (see also ATTACHMENT 3): The first set of samples will be sent to the bioanalytical laboratory. The ~~other~~ second set of samples will be stored at ~~Hohoe until~~ OCRC until the bioanalytical laboratory confirms receipt of the first set of samples. ~~all analyses are complete~~At that time, the second set of samples will be sent to the bioanalytical laboratory. After all analyses are complete and ~~after approval from~~ Wyeth has informed the bioanalytical laboratory that the quality and GCP compliance of the data has been verified, all remaining samples will ~~then~~ be discarded. PK data will not be collected on subjects who replace those who have withdrawn post drug administration.

15.3.6 Skin Snips

A total of 4 skins snips will be taken at each time point (from iliac crests and calves) using a corneoscleral punch (Walser or Holth-type). Each snip will be weighed on an analytical balance and incubated overnight in isotonic saline in a well of a flat-bottomed microtitre plate. The microfilariae that have emerged will be counted using an inverted microscope. The skin microfilarial density at each site will be documented as the number per mg of skin. One punch will be used per subject and punches will be sterilized between subjects using steam under pressure (autoclave).

16. TEST ARTICLE

16.1 Test Article(s) and Administration

Test article is administered only to subjects who have provided informed consent, fulfill all inclusion and exclusion criteria and have been assigned a randomization number by the unblinded third party pharmacist (see section 18.1). Once a randomization number and the associated test article has been assigned to a subject it must not be reassigned to another subject. All subjects will receive a total of 4 capsules. Matching placebo capsules will be administered as necessary so that each subject receives the same number of capsules independent of their treatment assignment.

The doses of moxidectin for this study are 2 mg, 4 mg, and 8 mg and will be administered to subjects in TIC, each HPMC capsule containing 2 mg moxidectin.

The moxidectin group will receive either 1, 2 or 4 moxidectin capsules based on the dosage for that group. (see Table 17.4.A and Attachment 4).

Ivermectin will be supplied as 3 mg tablets encapsulated in HPMC capsules and will be dosed according to the weight based dosing schedule approved for the treatment of onchocerciasis in the ivermectin package insert. (See Table 17.4.A and Attachment 4).

The recommended dosage of ivermectin in the treatment of onchocerciasis is a single oral dose designed to provide approximately 150 μg/kg of body weight.

The capsules will be swallowed with water.

Ivermectin Weight based Dosing Schedule[15]	
**Bodyweight (kg)**	**Dose<br>Number of 3 mg tablets**
26 to 44	Two
45 to 64	Three
65 to 84	Four

The doses of moxidectin for this study were selected based on safety data from an initial PK study in man and expected therapeutic blood levels based on those of ivermectin, a similar compound. The starting dose (2 mg or approximately 33 μg/kg for a 60 kg subject) is similar to the minimal effective dose of ivermectin (30 μg/kg).

16.2 Packaging and Labeling

The moxidectin tablets used in this study contains the following ingredients:

Component	% w/w
Moxidectin solid	2.00
Microcrystalline Cellulose	44.625
Lactose, Anhydrous	44.625
Croscarmellose Sodium	5.00
Sodium Lauryl Sulfate	3.00
Colloidal Silicon Dioxide	0.25
Magnesium Stearate	0.5

The placebo contains all ingredients listed above except the moxidectin solid.

Each ivermectin tablet contains 3 mg of active drug. In addition, each tablet contains the following inactive ingredients: microcrystalline cellulose, pregelatinized starch, magnesium stearate, butylated hydroxyanisole, and citric acid powder (anhydrous).

For blinding purposes each 2 mg moxidectin tablet and each 3 mg ivermectin tablet was inserted into an HPMC capsule filled with a mixture of inert ingredients (lactose, microcrystalline cellulose, magnesium stearate and sodium starch glycolate). Detailed instructions for the unblinded designated individual who prepares the drug are provided in Section 17.4 and Attachment 4.

The three capsules used in this study contain the following ingredients:

**Component**	**Moxidectin**	**Placebo**	**Ivermectin**
	**% w/w**		
Moxidectin 2 mg Tablet	1 Tablet	0	0
Ivermectin 3 mg Tablet	0	0	1 Tablet
Microcrystalline Cellulose	40	96.5	40
Lactose, Anhydrous	59	0	59
Sodium Starch Glycolate	0.5	0	0.5
Magnesium Stearate	0.5	0.5	0.5
Croscarmellose Sodium	0	3.0	0
Capsule #0 HPMC Opaque (Brown)	1 capsule	1 capsule	1 capsule

16.3 Storage and Stability

Moxidectin 2 mg TICs and Ivermectin 3 mg TICs as well as the placebo capsules should be stored at room temperature up to 30°C with protection from light.

16.4 Preparation

The drug will be prepared by an unblinded pharmacist on the day before dosing according to Table 17.4A and Attachment 4. The prepared drug, identified for each subject by subject name, initials, age, sex, weight, subject number and randomization number, will then be provided to the clinical study staff who will administer the drug.

Table 17.4.A: STUDY DRUG PREPARATION

Cohort	2 mg						4 mg						8 mg					
Treatment Assignment	Moxidectin			Ivermectin			Moxidectin			Ivermectin			Moxidectin			Ivermectin		
Capsule[a]	M	P	I	M	P	I	M	P	I	M	P	I	M	P	I	M	P	I
Weight	Number of Capsules																	
26-44 kg	1	3	0	0	2	2	2	2	0	0	2	2	4	0	0	0	2	2
45-64 kg	1	3	0	0	1	3	2	2	0	0	1	3	4	0	0	0	1	3
65-84 kg	1	3	0	0	0	4	2	2	0	0	0	4	4	0	0	0	0	4

a: M - Capsule containing 2 mg moxidectin tablet, P - Placebo capsule, I - Capsule containing 3 mg ivermectin tablet

16.5 Test Article Accountability, Reconciliation, and Return

The European Medicines Evaluation Agency (EMEA), and Special Program for Research and Training in Tropical Diseases (TDR) require accounting for the disposition of all investigational drugs received by each clinical site. Information on drug disposition required by law consists of the date received, date administered, quantity administered, and the subject to whom the drug was administered. Storage conditions must also be documented. The PI is responsible for accounting for all unused test articles and all used test article containers.

Supplies will be shipped to the principal investigator site for the study initiation. The OCRC will use the relevant form to document test article disposition and record storage conditions from initial receipt through the completion of the study. This form will be completed by the unblinded third party pharmacist and kept locked in a cabinet accessible to no one else. Each time a dose is prepared for a subject, the following information will be recorded by the unblinded third party pharmacist:

- the subject's initials
- the subject number
- randomization number
- the total dose prepared
- the number and type of capsules
- the packaging control number (lot number)from which the different types of capsules were taken
- the initials of the person preparing the dose

At a minimum, drug accounting will be reviewed by an unblinded monitor not involved in subject treatment or dose progression decision after the 30 day safety review of the last cohort to be enrolled has been completed.

At the termination of the study, a final drug accountability review and reconciliation will be completed, any discrepancies will be investigated, and their resolution will be documented. All unused test articles will be returned to Wyeth or shall be properly disposed of at the direction of Wyeth, including all empty containers. The test article accountability, reconciliation, and return procedures also apply to all other test articles (i.e. comparator, placebo) that are required by the protocol and supplied by the sponsors.

16.6 Study Subject Compliance

The study staff will directly observe compliance. Moxidectin and ivermectin will be administered in a single oral dose under medical supervision in the hospital and administration recorded on the source documents and CRF.

16.7 Other Supplies

Wyeth will provide additional clinical supplies such as CRFs, SAE reporting forms, PK specimen tubes and PK specimen labels.

17. MEASURES TO MINIMIZE/AVOID BIAS

17.1 Subject Identification

Subjects will be enrolled by dose and severity of *O. volvulus* infection. The intensity of infection will be based on the mean of the densities at the iliac crests and calves. Each cohort will consist of 16 subjects (12:4 moxidectin:ivermectin). They will receive a single oral dose followed by frequent safety monitoring over a 30-day period. There will be a total of 9 cohorts, based on the dose of moxidectin to be administered and the intensity of infection as follows:

- 2 mg: subjects with > 0 and < 10 microfilariae/mg of skin (16 subjects)
- 2 mg: subjects with 10-20 microfilariae/mg of skin and the sum of microfilariae in the two eyes must be ≤ 10 (16 subjects)
- 2 mg: subjects with > 20 microfilariae/mg of skin with or without ocular involvement (32 subjects) to be enrolled in 2 consecutive groups of 16 subjects.

- 4 mg: subjects with > 0 and <10 microfilariae/mg of skin (16 subjects)
- 4 mg: subjects with 10-20 microfilariae/mg of skin and the sum of microfilariae in the two eyes must be ≤ 10 (16 subjects)
- 4 mg: subjects with > 20 microfilariae/mg of skin with or without ocular involvement (32 subjects) to be enrolled in 2 consecutive groups of 16 subjects.

- 8 mg: subjects with > 0 and < 10 microfilariae/mg of skin (16 subjects)

- 8 mg: subjects with 10-20 microfilariae/mg of skin and the sum of microfilariae in the two eyes must be ≤ 10 (16 subjects)
- 8 mg: subjects with > 20 microfilariae/mg of skin with or without ocular involvement (32 subjects) to be enrolled in 2 consecutive groups of 16 subjects.

At the time of initiation of screening, each subject is assigned a unique subject number in the order in which they are scheduled for screening. Each subject randomized will keep this subject number for the duration of the study. Subject numbers will not be reassigned or reused for any reason. However, a subject who does not qualify for one cohort based on his/her skin microfilaria count or because the cohort currently being screened for is already complete, but qualifies for another cohort, can be re-screened prior to enrollment of the cohort for which he/she qualifies based on skin microfilaria counts and assigned the next subject number in sequence.

Screening evaluations (history, physical and ocular exam, ECG, lab evaluations, skin snip) will be performed during the 4-day screening period prior to administration of test article on day 1.

All subjects who are screened (including screen failures) will be recorded in an Onchocerciasis Chemotherapy Research Centre (OCRC) screening and enrollment log. Information to be collected includes:

- Date of screening
- Subject number
- Subject initials
- Ethnic origin
- Reason for screen failure (if applicable)

After completing the baseline investigations and qualifying for the study, each subject will be assigned a randomization number by an unblinded third party pharmacist. This number will automatically assign him/her to 1 of 2 treatment regimens (12:4 moxidectin:ivermectin) according to a predetermined schedule (section 18.2, Randomization). Subjects will be dosed according to the schedule in Table 17.4.A and Attachment 4. Treatment will be given after an overnight fast of at least 8 hours on day 1 under medical supervision. Breakfast will be delayed for 2 hours. Smoking, the ingestion of alcoholic beverages, grapefruit, or grapefruit juice, and the use of non-prescribed drugs will not be permitted during the 18-day

admission period. Subjects will be encouraged to continue this practice at least until the day 30-31 follow-up and to maintain their normal exercise patterns.

The unblinded third party pharmacist will maintain confidentiality of the randomization number lists and prepare the test articles for administration. The PI will provide the unblinded third party pharmacist with the names, subject numbers, baseline severity and gender of those who are eligible for the trial.

Subjects should be identified to the sponsors only by their assigned numbers, initials, age, and sex. Te investigator must maintain a list of subject names and the identifying information indicated above.

17.2 Randomization

Subjects will be assigned a randomization number by the unblinded third party pharmacist. The unblinded third party pharmacist will be supplied with 12 randomization schedules - one for each of the 3 severity levels (mild, moderate, severe) within each of the 3 dose cohorts (2 mg, 4 mg, 8 mg) and 3 additional schedules for the most severe (> 20 mf/mg of skin) subjects. Each randomization schedule will have 16 unique randomization numbers. The randomization numbers will be assigned by the unblinded third party pharmacist at the time of preparing the study medication and will be recorded on to the case report forms. Treatment groups will be blocked in groups of 4 with a 3 to 1 ratio of moxidectin to ivermectin. When the first male subject is enrolled, the assigned treatment (randomization number) will be selected from the top of the randomization list that corresponds to the subject’s assigned stratum. The treatment (randomization number) for the first female subject will be selected from the bottom of the randomization list that corresponds to the subject’s assigned stratum. The second male subject will be assigned the next available treatment from the top of the randomization list that corresponds to the subject’s assigned stratum. The second female subject will be assigned the next available treatment from the bottom of the randomization list that corresponds to the subject’s assigned stratum. Assignment to treatment group will continue in this manner until the entire cohort has been assigned to a treatment group. A new randomization schedule should be used when a new cohort is started.

At the end of the study, the randomization numbers will be used to identify the treatment group assigned to the subject.

17.3 Blinding and Unblinding

The study monitor, the investigator(s), and the subjects at the site will be blinded to treatment assignment. The unblinded third party pharmacist will not participate in the evaluation of any study subject. This will permit all other participants to remain blinded. The unblinded third party pharmacist will prepare and label all treatments with the names and randomization numbers of the subjects based on the randomization list. Individuals who are unaware of the test article random assignment will administer the treatments to the subjects. Contact between the unblinded third party pharmacist and study subjects will be kept to a minimum. The investigator, sub-investigator, research nurse, and any study participants other than the unblinded third party pharmacist will not be allowed to know the treatment assigned to any study subject and will not have access to the randomization treatment records.

The subject’s treatment will be unblinded electively only if an AE occurs that the investigator, the Wyeth medical monitor and the WHO clinical coordinator or project manager believe requires identification of the treatment assignment. This may occur in the case of a SAE, as the investigator’s decision to enroll the next cohort within the same dose group may be influenced by whether a SAE occurred in a subject or subjects who received moxidectin or in one who received ivermectin. This will also affect the sponsors’ agreement with the investigator’s decision. In this case, the unblinded third party pharmacist will inform the investigator, who will inform the Wyeth medical monitor of the subject’s treatment assignment and the reason(s) for breaking the randomization code.

Routine unblinding of SAEs will not be performed in this protocol. Ivermectin and moxidectin are macrocyclic lactones and are expected to have similar adverse event profiles. This is a single-dose study and thus there will be no question regarding continuation of therapy after an SAE. The treatment of SAEs will not be affected by whether the subject receives ivermectin or moxidectin.

Emergency unblinding will be at the discretion of the Principal Investigator. The investigator must submit a written explanation describing the event to the sponsors within 5 working days. All randomization envelopes must be returned to the sponsors at the completion of the trial.

18. SAFETY

18.1 Safety Variables

All subjects enrolled in this study will be closely monitored for safety and evaluated for any AEs. Safety evaluation will include: symptoms, physical examinations, vital signs measurement, ECGs, ocular exams, and laboratory evaluations. All evaluations will be monitored as outlined in section 16.1, Procedures, and section 16.3, Special Methods. A key safety component of the study drug will be determined by the presence and severity of the Mazzotti reaction. An important consideration in the administration of microfilaricides to *O. volvulus* infected subjects is the potential for the combination of AEs due to the pharmacologic properties of the drug with the constellation of symptoms, signs and laboratory events that occur when microfilariae are killed (the Mazzotti reaction).[16] These need to be differentiated one from the other and from any coincidental illnesses that may occur in temporal relationship with test drug administration. Common systemic clinical manifestations of the Mazzotti reaction include pruritus, rash, lymphadenitis, headache, myalgia, arthralgia, hypotension, fever and swellings of the face and limbs. Ocular events include epiphora, photophobia, conjunctival injection, limbitis, anterior uveitis, chorioretinitis and optic neuritis.

The clinical laboratory changes involve the peripheral blood leucocytes, aspartate aminotransferase (AST/SGOT) and alanine aminotransferase (ALT/SGPT) and sometimes lactate dehydrogenase (LDH) and gamma-glutamyl-transferase (GGT); microfilariae also appear in blood, urine, and other body fluids. The eosinophils exhibit the most prominent changes. There may be an initial eosinopenia followed several days later by a marked increase above pretreatment levels. A complete disappearance of eosinophils from the peripheral blood is the laboratory hallmark of a severe reaction. Lymphocyte counts may fall initially, followed by lymphocytosis, but not to the same extent as with eosinophils. Leucocytosis with neutrophilia is less common. Elevations in liver enzymes occur, but rarely exceed grade 2 and usually normalize by day 30. Levels of bilirubin and alkaline phosphatase are usually unchanged. Any deviation from this pattern of liver function abnormalities should prompt investigations for an intrinsic drug effect or a coincidental illness. Proteinuria may occur. Although the laboratory changes per se have little clinical significance, they give indirect evidence of the death of microfilariae and may even indicate the speed and severity of the reaction to the event. [17]

The factors that govern the Mazzotti reaction include the intensity of infection,[18] the dose regimen, and the microfilaricide used. These determine the onset, evolution, reaction severity, extent of the laboratory changes, and whether the reaction is mono- or biphasic. A notable exception occurs in subjects with hyper-reactive onchodermatitis (Sowda),[19] where severe, predominantly cutaneous adverse effects occur even with very low skin microfilarial counts.

For a given intensity of infection, the severity of the Mazzotti reaction to ivermectin is independent of dose within the range of 150 to 800 μg/kg (approximately 9 mg-48 mg).[20] Dangerous and alarming reactions result from the simultaneous occurrence of severe reactions in multiple systems.[21] This phenomenon is rare with ivermectin. Biphasic reactions, characterized by a marked recrudescence of cutaneous or lymph node symptoms or the development of an acute febrile polyarthritis, [22]several days after the initial reaction, have also not been observed with ivermectin. There is no reason to believe that the Mazzotti response to moxidectin would differ radically from those due to ivermectin. However, a more rapid elimination of microfilariae may result in an earlier onset, or a more severe reaction, for a given initial infection intensity.

Severe reactions can usually be aborted by the administration of 100 to 200 mg of hydrocortisone hemisuccinate intravenously aided by supportive therapy.

The clinical events will be graded according to the National Cancer Institute common toxicity table, version 2.0, with additions specific for this protocol summarized in Attachments 5 and 6.

More frequent safety evaluations may be made if clinically indicated or at the discretion of the investigator. All adverse effects (AEs) will be recorded in the CRFs throughout the entire study.

18.2 Safety Assessment Methods and Dose Progression Decision

The decision to move to the next higher dose group, will be made based on blinded review of the safety and microfilaricidal data obtained during the first 30 days post treatment of all previously enrolled cohorts.

The PI will submit a recommendation to proceed or not to proceed after a detailed examination of the blinded data. The Wyeth medical monitor, the WHO clinical monitor and the WHO Project Manager will

conduct their separate reviews. Wyeth will include a representative of its safety surveillance group in addition to the medical monitor for purposes of review. WHO may include its Clinical Coordinator or another physician in the review.

The PRT will discuss the blinded data and the recommendation to progress or not. The recommendation will be provided together with the rationale for this recommendation to CERT who have access to the randomized dose assignments and have reviewed the data in parallel with the PRT. If there is a difference of opinion within the PRT regarding the dose progression recommendation, the different recommendations and their rationale will be provided to the CERT. The CERT will provide their recommendation to the PRT.

The experts will have access to the treatment codes and will review the data initially in a blinded fashion with the option to unblind at a patient or cohort level, with special focus on the following:

- The number of possibly, probably or definitively drug related non-CNS AEs ≥ grade 2 among moxidectin treated subjects.
- The number of subjects with possibly, probably or definitively drug related CNS AEs >grade 1 that are not related to the efficacy of moxidectin (i.e. not characterized by the PI as Mazzotti reactions) among moxidectin treated subjects.
- Any moxidectin treated subject with a possibly, probably or definitively drug related SAE not related to the efficacy of moxidectin (i.e. not characterized by the PI as Mazzotti reactions).

The review will take the clinical significance of the AEs in the context of the control programs into consideration.

If there is not unanimous agreement among the CERT, they will provide their differing recommendations and their rationale. If there is not unanimous agreement on proceeding to the next dosing cohort between the PRT and a majority of the CERT, the data will be discussed in a blinded fashion. If no agreement can be reached, unblinded data will be made available to the PRT for the subject(s) in question and discussed. If upon discussion of the unblinded data there is no unanimous agreement to proceed to the next higher dose group, dose progression will not occur. A more detailed description of the procedures in the dose progression monitoring plan will be reviewed and approved by the PRT and the CERT.

18.3 Safety Laboratory Determinations

All clinical and laboratory evaluations that deviate from baseline values after study drug administration and are considered to be clinically significant will be repeated and followed until normalization. If normalization does not occur within 2 weeks or by day 30, the etiology will be identified, if possible, and the Wyeth medical monitor, the WHO clinical monitor, and the WHO project manager, will be notified.

19. EFFICACY

19.1 Efficacy Variables

Efficacy against the microfilariae will be determined by reductions from initial counts in skin and ocular microfilariae (where applicable).

19.1.1 Primary

This study will give initial indications of the efficacy of moxidectin against the microfilariae of *O. volvulus*. The primary efficacy variable will be:

- The change from baseline in the mean number of skin microfilariae per milligram of skin sampled at 4 body locations (right and left iliac crests, right and left calves) at 18 months after test article administration

19.1.2 Secondary

Secondary efficacy variables will include:

- The mean reduction from baseline, as well as the percentage of subjects with undetectable microfilariae, will be calculated by dose and severity of infection for each post treatment evaluation time point.
- The change from baseline in the mean number of skin microfilariae per milligram of skin sampled at 4 body locations (right and left iliac crests, right and left calves) at 12 months after test article administration.

- The change from baseline in the mean number of skin microfilariae per milligram of skin sampled at day 8, 1 month, 2 months, 3 months, and 6 months.
- The change from trough values to 12 months and 18 months post treatment in the mean number of skin microfilariae per milligram of skin sampled at 4 body locations (right and left iliac crests, right and left calves) to quantify the effect on the reproductive capacity of adult worms.
- The area under the curve (calculated by using the trapezoidal method through the 18 month visit)
- Ocular examination (time of maximum reduction of microfilariae and number (%) of subjects with complete clearance). The change from baseline in the mean number of ocular microfilariae measured in the anterior chamber. Living and dead microfilariae in the cornea and punctate opacities will also be counted. Retinal changes secondary to the disease will also be examined. Ocular examinations will be performed at baseline and on days 3 or 4, 7 or 8, 14 or 15, and at 1 month, 3 months, 6 months, 12 months and 18 months.
- Evidence of an effect on the viability and/or fertility of the macrofilaria will be obtained from the histologic examination of nodules excised at 18 months. Parameters to be evaluated include:

  a. Viability (number live, moribund, dead)
  b. Calcification (presence/absence)
  c. Reproductive status:
     i. Females:
        Proportion:
        1. Number producing embryos up to the microfilarial stage
        2. Degenerated microfilariae in uterus
        3. Relict embryos
     ii. Males:
        Proportion with:
        1. Spermatogenesis normal or abnormal
     iii. Other criteria:
        Proportion of nodules with:
        1. Microfilariae in the capsule
        2. No male worms

19.2 Efficacy Assessment Methods

- The maximal microfilaricidal effect on skin microfilariae will be determined by skin snips taken at day 8 and 1 month (± 1 week).

- Nodules will be processed at 18 months for histological examination and assessed by the OCRC parasitology department (blinded to the treatment).

20. PHARMACOKINETIC ANALYSIS

Blood samples for determination of moxidectin plasma concentrations will be collected within 2 hours prior to dosing (0 hr), and at 1, 2, 4, 8, 24, and 72 hours after study drug administration, on study days 8, 13, and 18 and on months 1 (± 1 week), 2 (± 1 week), 3 (± 1 week), 6 (± 1 month) and 12 (± 1 month). PK samples will be drawn from all subjects in all cohorts with the exception of the 2nd group of 16 subjects with > 20 microfilariae/mg of skin in cohorts 3, 6, and 9. Samples from subjects will be analyzed by a validated bioanalytical method.

A PK analysis will be performed using model independent methods to characterize the PK parameters of moxidectin, such as maximum plasma concentration (C_{max}), time to reach the maximum plasma concentration (t_{max}), area under the plasma concentration vs. time curve (AUC) and the apparent terminal elimination half-life ($t_{1/2}$).

21. STATISTICAL ANALYSIS

21.1 Statistical Methods

This is a randomized, double-blind, active-control, dose-escalation trial comparing 3 doses of moxidectin (2, 4, and 8 mg) with ivermectin, stratified by microfilariae density (mild, moderate, severe) and gender. Subjects will be treated as in subjects for the first 18 days and as outpatients for the remainder of the 18 month study.

The primary analysis population for safety will be the modified intent-to-treat (mITT) population, which consists of all subjects who receive study medication. For efficacy, the primary analysis population will be the evaluable subject population, which is defined as all subjects who receive study medication and have baseline and 18-month microfilariae count data. An mITT sensitivity analysis will also be performed to evaluate the effect on the efficacy results of dropouts occurring before 18 months.

The primary objective of the study is to evaluate the safety of escalating moxidectin regimens. Both the incidence of clinical AEs and clinically significant laboratory test results will be used to access safety.

The primary efficacy endpoint is the reduction from baseline in skin microfilariae at 18 months. Microfilariae count will be defined as the geometric mean of skin microfilariae/mg taken at 4 sites (iliac crests and calves).

In addition, important secondary endpoints are the reduction from baseline in skin microfilariae load at day 8, 1 month, 2 months, 3 months, 6 months and 12 months, area under the curve (calculated by using the trapezoidal method through the 18 month visit), ocular examination (time of maximum reduction of microfilariae and number (%) of subjects with complete clearance) and nodulectomy (viability and fertility of macrofilariae) at 18 months.

21.2 Methodology

Initially, an overall among-group test will be performed to determine if any significant differences exist among the 4 treatment groups (2 mg, 4 mg, 8 mg moxidectin, and ivermectin). For this analysis the ivermectin control will be pooled over all nine cohorts (three dose groups). Following a significant overall result, paired comparisons (i.e., each moxidectin group versus ivermectin) will be performed to characterize the results. Prior to pooling the nine cohorts the ivermectin response for the nine cohorts will be graphically evaluated to ensure that there is no time effect on the pooled ivermectin results. For safety, moxidectin will be compared with ivermectin with respect to the incidence of AEs by using the Fisher’s exact test. Laboratory data will be analyzed by using the analysis of covariance, adjusting for baseline. For efficacy, the microfilariae counts will be logarithmically transformed (Mosteller and Tukey) and analyzed by using the analysis of covariance, adjusting for baseline counts and the number of sites from which the microfilariae were taken. The latter covariate will be used to adjust for subjects who have fewer than 4 microfilarial collection sites. Factors in the model will include treatment group, covariates, level of microfilariae density (mild, moderate, severe), and gender. Also, a sensitivity analysis will be performed to examine the effect on results of second-order interactions. Data results will be summarized as adjusted geometric means. If the assumptions of normality or log-normality are not met, the data will be analyzed by using the Friedman Test, blocking on baseline skin microfilariae density and gender. In order to evaluate the robustness of the results, the microfilariae data will also be analyzed as a percent change from baseline. The proportion of subjects cured (i.e. with undetectable microfilariae) will also be evaluated.

For the analysis of microfilariae viability and fertility endpoints, proportions will be analyzed by using the Cochran-Mantel-Haenszel procedure blocking on baseline skin microfilariae density. If the overall test is significant, paired comparisons will be made comparing each moxidectin group with ivermectin.

For ocular microfilariae, time to maximum reduction will be analyzed by using the Friedman Test blocking on baseline skin microfilariae density. The proportion of subjects with complete clearance will be analyzed by the Cochran-Mantel-Haenszel procedure blocking on baseline skin microfilariae density. If the overall test is significant, paired comparisons will be made comparing each moxidectin group with ivermectin.

All tests will be performed as 2-sided tests with $\alpha = 0.05$.

21.3 Sample Size and Power

For the mild and moderate severity levels in each dose group, 16 subjects will be randomly assigned in a 3:1 ratio to either moxidectin or ivermectin, for the severe level in each dose group 32 subjects will be randomly assigned to treatment using the same ratio. The probability of detecting at least 1 AE of ~~NCI~~ grade 2 or higher among 12 moxidectin-treated subjects will be 0.114 and 0.718 when the true rates are 1% and 10%, respectively. The probability of detecting at least 1 AE of ~~NCI~~ grade 2 or higher among 24 moxidectin-treated subjects will be 0.214 and 0.92 when the true rates are 1% and 10%, respectively.

For efficacy, given that the subjects in the three severity levels within a moxidectin dose group can be pooled and that all ivermectin subjects can be pooled, there will be 48 subjects per moxidectin dose group and 48 subjects in the pooled ivermectin treatment group. With 48 subjects per treatment group there is 78.3% power to detect a statistically significant difference between a response (% of baseline microfilariae) of approximately 0.1% (moxidectin) and 20% (ivermectin) at 18 months after treatment.

22. CONTRAINDICATIONS, PRECAUTIONS, AND WARNINGS

Safety studies have been completed in mice, rats, dogs, sheep, cattle, horses, and humans. At toxic dose levels, events such as decreased activity, prostration, tremors, chromodacryorrhea, decreased respiration, diarrhea, hypersensitivity to touch and sound, and epistaxis were observed. However, the NTELs in all of the animal models were high multiples of the anticipated human dosage on the basis of body weight.

Further information is given in the Moxidectin Investigator's Brochure provided. The data from the first study in man, a single ascending dose study in uninfected subjects followed for 80 days after dose administration show that moxidectin is safe and well tolerated at single doses between 3 mg and 36 mg. No SAEs and no grade 4 AEs were reported. Only 1 AE higher than grade 2 was reported in this study (enteritis due to food poisoning [grade 3] in the 36 mg fasting group) and was considered unrelated to study drug. The most common AEs were headache (35%) and infection (29%). Only half of the headaches reported and none of the infections reported were regarded as study drug related. The reported infections, including dose group, infection type and days after moxidectin administration, were: 3 mg fasted – upper respiratory tract infection (URTI) on Day 19 and Day 63 (same subject), viral URTI on Day 14; 9 mg fasted – toe infection on Day 52; 9 mg fed – URTI on Day 30 and Day 78 (different subjects); 36 mg fasted – tooth abscess on Day 47, head cold on Day 50 and Day 66 (different subjects); 36 mg fed – cold on Day 42 and cold-like symptoms on Day 59 (same subject). Four (4) subjects discontinued from the study (1 before receiving study drug and 3 after dose administration [in the 3 mg fasting, 9 mg fed, and 36 mg fasting groups]). The withdrawal from the study for these subjects was unrelated to AEs.

In a bioavailability study comparing the tablet and liquid forms of moxidectin (protocol 3110A1-101-EU) 58 healthy male subjects received a single dose of 10 mg of moxidectin either as the liquid formulation (29 subjects) used in the FIM study or as tablets (29 subjects) and were followed up for 180 days. During the course of this study there were no SAEs and no subjects discontinued the study due to an AE. A total of 36 (62.1%) subjects had treatment emergent AEs (TEAEs) during the study, with the same number and percentage of subjects (18; 62.1%) reporting TEAEs in both the moxidectin liquid and tablet groups. During the first 7 days post treatment, 10 subjects (34.5%) receiving liquid moxidectin and 9 subjects (31.0%) receiving tablet reported AEs, including asthenia (10.3% in the tablet group), headache (13.8% and 6.9% in the liquid and tablet groups, respectively), infection (6.9% in the liquid group), diarrhea (6.9% in the liquid group), myalgia (6.9% in the tablet group), and dizziness (6.9% in the tablet group). Between day 8 and 180 of follow up, 10 subjects (34.5%) receiving liquid and 12 subjects (41.4%) receiving tablet reported AEs. The most commonly reported events were flu syndrome (17.2% and 20.7% in liquid and tablet groups, respectively), headache (6.9% in the tablet group), and infection (6.9% in the tablet group). All of the TEAEs were mild to moderate in intensity, and none were considered to be related to treatment. No clinically relevant abnormalities were observed in vital sign measurements, ECGs, or laboratory tests during the study.

For a complete description of all (AEs) reported during the Phase 1 clinical studies with moxidectin, please refer to the most recent moxidectin Investigator's Brochure provided.

23. ADVERSE EVENTS

23.1 Definitions

The term "adverse event," as used by the sponsors, is synonymous with the term "adverse experience," which is used by the FDA.

An ***adverse event*** is any untoward, undesired, unplanned clinical event in the form of signs, symptoms, disease, or laboratory or physiological observations occurring in a human being participating in a clinical study with a sponsor's test article, regardless of causal relationship. This includes the following:

- Any clinically significant worsening of a preexisting condition.
- An AE occurring from overdose of a sponsor test article whether accidental or intentional (ie, a dose higher than that prescribed by a health care professional for clinical reasons).
- An AE occurring from abuse of a sponsor's test article (ie, use for nonclinical reasons).
- An AE that has been associated with the discontinuation of the use of a sponsor's test article.

Note: A procedure is not an AE, but the reason for a procedure may be an AE.

A ***preexisting condition*** is a clinical condition (including a condition being treated and AEs occurring between ICF signing to the subject's arrival at the clinic) that is diagnosed or occurs before the screening of the subject for this study is initiated in the hospital. A preexisting condition can be part of the subject's medical history or part of baseline conditions and are recorded in the source records and on the appropriate page of the CRF (for randomized subjects).

Baseline conditions are all those clinical conditions, including those related to infection with Onchocerca volvulus, present at the time of screening. Medical history are all clinical conditions known to have been present prior to screening. A condition can be both part of the medical history and baseline conditions if the condition started prior to screening and is still present at screening (e.g. any condition related to infection with Onchocerca volvulus).

Adverse events occurring during and due to screening procedures will be recorded as adverse events.

AE collection on source documents starts on the date of subject's arrival at the clinic / start of screening. From this time point, any SAEs are reported to Wyeth within 1 business day from their occurrence.

The questions concerning whether the condition existed before the start of the active phase (i.e. treatment) of the study and whether it has increased in severity and/or frequency will be used to determine whether an event is a treatment-emergent AE (TEAE). An AE is considered to be treatment emergent if (1) it was not present when the active phase of the study began and is not a chronic condition that is part of the subject's medical history, or (2) it was present at the start of the active phase of the study or as part of the subject's medical history, but the severity or frequency increased during the active phase.

The active phase of the study begins at the time of the administration of the test article and ends at the 18 month visit.

A ***serious adverse event*** (SAE) is any AE occurring at any dose that meets 1 or more of the following criteria:

- Results in death
- Is life threatening (see below)
- Requires inpatient hospitalization or prolongation of an existing hospitalization (see below)
- Results in a persistent or significant disability or incapacity (see below)
- Results in cancer
- Results in a congenital anomaly or birth defect

Additionally, important medical events that may not result in death, be life threatening, or require hospitalization may be considered SAEs when, based on appropriate medical judgment, they may jeopardize the subject and may require medical or surgical intervention to prevent one of the outcomes listed above. Examples of such events include allergic bronchospasm requiring intensive treatment in an emergency room or at home, blood dyscrasias or convulsions that do not require hospitalization, or development of drug dependency or drug abuse.

A ***life threatening adverse event*** is any AE that places the subject at immediate risk of death from the event as it occurred. A life-threatening event does not include an event that might have caused death had it occurred in a more severe form but that did not create an immediate risk of death as it actually occurred. For example, drug-induced hepatitis that resolved without evidence of hepatic failure would not be considered life threatening, even though drug-induced hepatitis of a more severe nature can be fatal.

Hospitalization is to be considered only as an overnight admission. Hospitalization or prolongation of a hospitalization is a criterion for considering an AE to be serious. In the absence of an AE, the participating investigator should not report hospitalization or prolongation of hospitalization on a Serious Adverse Event form (a 7443 form). This is the case in the following situations:

- Hospitalization or prolongation of hospitalization is needed for a procedure required by the protocol. Day or night survey visits for biopsy or surgery required by the protocol are not considered serious.

- Hospitalization or prolongation of hospitalization is part of a routine procedure followed by the study center (eg, stent removal after surgery). This should be recorded in the study file.

- Hospitalization for survey visits or annual physicals fall in the same category.

In addition, a hospitalization planned before the start of the study for a preexisting condition that has not worsened does not constitute an SAE (eg, elective hospitalization for a total knee replacement due to a preexisting condition of osteoarthritis of the knee that has not worsened during the study).

Disability is defined as a substantial disruption in a person’s ability to conduct normal life functions.

If there is any doubt whether the information constitutes an AE or SAE, the information is treated as an AE or SAE.

23.2 Timing for Reporting Serious Adverse Events

Any SAE, regardless of causal relationship, must be reported to the Wyeth medical monitor, the WHO clinical monitor, the WHO project manager and the Coordinator of the Ghana National Center for

Pharmacovigilance (GNCP) (or their designees) immediately (no later than 24 hours after the investigator becomes aware of the SAE) by faxing a completed serious adverse event form (form 7443) to the numbers indicated in the front of this protocol and then confirming by telephone that the faxes were received. Compliance with this time requirement is essential so that the sponsors may comply with their regulatory obligations.

Follow-up information relating to an SAE must be reported to the Wyeth medical monitor, the WHO clinical monitor, the WHO project manager and the Coordinator of the Ghana National Center for Pharmacovigilance (or their affiliates or designees) within 24 hours of receipt by the investigator by faxing a completed serious adverse event form (form 7443) to the numbers indicated in the front of this protocol and confirming by telephone that the faxes were received. The subject should be observed and monitored carefully until the condition resolves or stabilizes or its cause is identified.

Any emergency must be reported to the Wyeth medical monitor, the WHO clinical monitor, the WHO project manager and the Coordinator of the Ghana National Center for Pharmacovigilance (or their affiliates or designees) immediately (within 24 hours).

For all other inquiries and information about this study, contact a clinical scientist listed in the front of this protocol.

23.3 Reportable Events/Information

- An AE or SAE can occur from the time that the subject begins the hospital screening period to the completion of the final study visit at month 18; regardless of test article or protocol relationship. All AEs and SAEs will be recorded on source documents. All AEs and SAEs for subjects who are not screen failures will be recorded in the CRFs. The investigator must follow up as is medically necessary on all AEs, SAEs, and other reportable events until the event has subsided or values have returned to baseline, or in case of permanent impairment, until the condition stabilizes.

  For SAEs: The investigator will provide all documentation pertaining to the event (eg, additional laboratory tests, consultation reports, discharge summaries, postmortem reports, etc) to the Wyeth medical monitor, the WHO clinical monitor, the WHO project manager and the Coordinator of the Ghana National Center for Pharmacovigilance (or their affiliates or designees) in a timely manner. Reports relative to the subject's subsequent course must be submitted to the Wyeth medical monitor,

the WHO clinical monitor, the WHO project manager and the Coordinator of the Ghana National Center for Pharmacovigilance (or their affiliates or designees) until the event has subsided or, in case of permanent impairment, until the condition stabilizes.

- The following events will be recorded and reported in the same time frame and following the same process as for SAEs:

  1. All pregnancies that occur during the study and their outcome. If a pregnancy is confirmed during the study, the Wyeth medical monitor, the WHO clinical monitor, the WHO project manager and the Coordinator of the Ghana National Center for Pharmacovigilance (or their affiliates or designees) should be notified immediately. When possible, all reports of pregnancy must be followed up for information about the course of the pregnancy and delivery, as well as the condition of the newborn. When the newborn is healthy, additional follow-up is not needed. The investigator will provide follow-up information concerning the outcome of the pregnancy to the sponsors and the Coordinator of the GNCP in a timely manner. This information will be provided regardless of whether the subject has discontinued participation in the study.

  2. Test article abuse and overdose (ie, use for nonclinical reasons) with or without AEs. An overdose is a dose higher than that prescribed by a health care professional for clinical reasons. It is up to the participating investigator to decide whether a dose was an overdose.

  3. Inadvertent or accidental exposure to test article with or without an AE.

     - Poststudy test article-related SAEs.
     - SAEs occurring after unauthorized or accidental use in persons not participating in the study.
     - Abnormal biological or vital signs values that are considered clinically relevant by the participating investigator. These must be reported in the same time frame and following the same process as for an AE or an SAE.

23.4 Recording and Reporting

At each required study visit, all AEs that have occurred since the previous visit must be recorded in the adverse event record of the subject’s CRF. The information recorded should be based on the signs and symptoms detected during the physical examination and clinical evaluation of the subject. In addition to the information obtained from those sources, the subject should be asked the following nonspecific

question: "How have you been feeling since your last visit?" Signs and symptoms should be recorded using standard medical terminology.

The following AE information must be included (when applicable): the specific condition or event and direction of change; whether the condition was preexisting (ie, an acute condition present at the start of the study or history of a chronic condition) and, if so, whether it has worsened (eg, in severity and/or frequency); the dates and times of occurrence; severity; causal relationship to test article; action taken; and outcome. The causal relation between an AE and the test article will be determined by the investigator on the basis of his or her clinical judgment and the following definitions:

- ***Definitely related***: Event can be fully explained by administration of the test article.
- ***Probably related***: Event is most likely to be explained by administration of the test article rather than the subject's clinical state or other agents/therapies.
- ***Possibly related***: Event may be explained by administration of the test article or by the subject's clinical state or other agents/therapies.
- ***Probably not related***: Event is most likely to be explained by the subject's clinical state or other agents/therapies, rather than the test article.
- ***Definitely not related***: Event can be fully explained by the subject's clinical state or other agents/therapies.

When assessing the relationship between administration of a test article and an AE, the following should be considered:

- Temporal relationship between administration of the test article and the AE
- Biological plausibility of relationship
- Subject's underlying clinical state or concomitant agents and/or therapies
- When applicable, whether the AE abates on discontinuation of the test article (dechallenge)
- When applicable, whether the AE reappears on repeat exposure to the test article (rechallenge)

SAEs that are not test article related may nevertheless be considered by the participating investigator or the medical monitor (or designee) to be related to the conduct of the clinical study, ie, to a subject's participation in the study. For example, a protocol-related SAE may be an event that occurs during a washout period or that is related to a procedure required by the protocol.

The severity of AEs will be assessed according to the Onchocerciasis Chemotherapy Research Centre Common Toxicity Criteria (OCRC CTC), version 2.0, 1980, 1992, 1999[23,24] 2006 and according to the National Cancer Institute Common Toxicity Criteria (NCI CTC), version 2.0, April 30, 1999.[25] for all those AEs not included in the OCRC CTC a.Additions to this grading scale for oOCRC CTC can be found in ATTACHMENT 5cular, hematological and biochemical adverse events can be found in Attachments 5 and 6 respectively.

24. DATA QUALITY ASSURANCE

The sponsors perform quality control and assurance checks on all clinical studies that they sponsor. Before enrolling any subjects in this study, sponsors' personnel and the investigator review the protocol, the brochure for clinical investigators, the CRFs and instructions for their completion, the procedure for obtaining informed consent, and the procedure for reporting AEs and SAEs. A qualified representative of the sponsors monitors the conduct of the study by visiting the site and by contacting the site by telephone. During the visits, information recorded on the CRFs is verified against source documents. All data on the CRFs will have corresponding source records in patient charts or laboratory records. After the sponsors receive the CRFs, the data are entered into the database by using a double data entry procedure. The sponsors' medical monitor reviews the data for safety information. The sponsor clinical data associates review the data for legibility, completeness, and logical consistency. Additionally, the sponsors clinical data associates use automated validation programs to help identify missing data, selected protocol violations, out-of-range data, and other data inconsistencies. Requests for data clarification or correction are forwarded to the investigative site for resolution. A sample set of records from the final database will be fully audited against the corresponding CRFs.

25. INVESTIGATORS REGULATORY OBLIGATIONS

25.1 Independent Ethics Committee (GFDB/IEC) Approval

The protocol and the informed consent document must have the initial and at least annual (when required) approval of the GFDB/IEC. The signed GFDB/IEC approval letter must identify the documents approved (ie, list the investigator's name, the protocol number and title, the date of the protocol and informed consent document, and the date of approval of the protocol and the informed consent document). Written information to be provided to the subject (eg, diary cards, instructions for test article administration) and

any advertisement used to recruit subjects must also be reviewed by the GFDB/IEC. The sponsors will not ship clinical supplies until a signed approval letter from the GFDB/IEC has been received and a contractual agreement has been signed by the sponsors and the clinical site. Copies of the regulations relating to IECs are available from the sponsors.

25.2 Prestudy Documentation

The investigator must provide the sponsors with the following documents BEFORE enrolling any subjects:

- All applicable country-specific regulatory forms.
- Current signed and dated curricula vitae for the investigator, subinvestigators, and all key personnel listed on the clinical study information form.
- Copy of the GFDB/IEC approval letter for the protocol and informed consent. Written assurance of continuing approval (at least annually) as well as a copy of the annual progress report submitted to the GFDB/IEC must also be provided to the sponsors. Any changes in this study or unanticipated problems involving risks to the subjects must be reported promptly to the GFDB/IEC. An investigator must not make any changes in a study without GFDB/IEC and sponsors approval except when necessary to eliminate apparent immediate hazards to the subjects. All protocol amendments must be submitted to the GFDB/IEC and approved.
- Copy of the GFDB/IEC-approved informed consent document to be used.
- When applicable, a list of the GFDB/IEC members and their qualifications, and a description of the committee’s working procedure.
- Copy of the protocol sign-off page signed by the investigator.
- Fully executed clinical study agreement (CSA).
- A written document containing the name, location, certification number, and date of certification of the laboratory to be used for laboratory assays and those of other facilities conducting tests. This document should be returned along with the statement of investigator form. The sponsors must be notified if the laboratory is changed or if any additional laboratory is to be used.
- List of normal laboratory values and units of measure for all laboratory tests required by the protocol. This is required for each laboratory to be used during the study. The sponsors must be notified if normal values or units of measurement change.

25.3 Informed Consent

Regulatory agencies have issued regulations to provide protection for human subjects in clinical investigations and to describe the general requirements for informed consent.

A copy of your proposed informed consent document should be submitted to the sponsors for review and comment before submission to your IEC. The study should not begin until the document has been reviewed by the sponsors and must not begin until the document has been approved by the IEC. In some instances the study must not begin until the document has been approved by a regulatory agency.

The informed consent document shall contain all of the elements of informed consent specified in the regulations. Some regulations may require the disclosure of additional information to the subject and/or inclusion of additional information in an informed consent document. Copies of the regulations relating to informed consent and the protection of human subjects in clinical studies are available from the sponsors.

Nothing in these regulations is intended to limit the authority of a physician to provide emergency medical care under applicable regulations. In addition, the PI should be aware that some regulations require that he/she permit regulatory agencies to conduct inspections and review records pertaining to this clinical investigation.

The delegation of investigator responsibilities including informed consent will be documented on the clinical study information form.

25.4 Declaration of Helsinki

This study will be conducted in accordance with the ethical principles that have their origin in the Declaration of Helsinki and that are consistent with good clinical practice (GCP) and the applicable regulatory requirements.

25.5 Case Report Forms

1) All data will be recorded on CRFs provided by the sponsors. Black ballpoint pens should be used.

2) The white and yellow originals, or the white originals for North American studies, must be returned to the sponsors; the investigator must retain the pink copy for his/her file. For some studies, the specified copy will be sent to the central lab.
3) CRFs and other pertinent records are to be submitted to the sponsors during and/or at completion or termination of the study. The investigator also must submit all incomplete CRFs that document subject experience with the test article, including retrievable data on subjects who withdraw before completion of the study.

25.6 Adverse Event Reporting

The investigator agrees to report all AEs to the sponsors as described in the Adverse Events section. Furthermore, the investigator is responsible for ensuring that any sub-investigator promptly brings AEs to the attention of the investigator. The investigator is responsible for reporting any AE to the Ghana National Center for Pharmacovigilance (GNCP) and to send copies of all correspondence with the GNCP to Wyeth and the WHO. If applicable, the investigator also is responsible for informing the participating IEC of any SAEs.

25.7 Review of Source Records

The investigator agrees that qualified representatives of the sponsors and regulatory agencies will have the right, both during and after this study, to conduct inspections and to audit and review medical records pertinent to the clinical study as permitted by the regulations. Subjects will not be identified by name, and confidentiality of information in medical records will be preserved. The confidentiality of the subject will be maintained unless disclosure is required by regulations. Accordingly, the following statement (or similar statement) will be included in the informed consent document:

Representatives of regulatory agencies GFDB/IECs, the sponsors, the WHO, the Review Board of the Ghana National Center for Pharmacovigilance, and your personal physician may review your medical records and all information related to this study as permitted by law. Identifying information will not appear on any record received by the sponsors. Your identity will remain confidential unless disclosure is required by law.

25.8 Monitoring of the Study

This study is monitored by a representative from the WHO. Site visits are made before the study begins, at regular intervals during the study, and at the study closeout. Communication by telephone and mail and e-mail may be used as needed to supplement site visits. The investigator and study personnel will cooperate with the sponsors, provide all appropriate documentation, and be available to discuss the study. The purpose of the site visits is to verify the following:

1) Adherence to the protocol. (The investigator should document and explain any deviation from the approved protocol.)
2) The completeness and accuracy of the CRFs and the dispensing and inventory record. (Adequate time and space for these visits should be allocated by the investigator.)
3) Compliance with regulations. The verification will require comparison of the source documents to the CRFs.

25.9 Protocol Amendments

Any significant change in the study protocol will require an amendment. The investigator will outline the reasons and justification for the amendment in line with the TDR SOP for clinical investigators, and will discuss with the WHO clinical monitor. The monitor will append his comments and recommendations to the document that will then be submitted to the Wyeth medical monitor and WHO Project manager. A protocol amendment will be generated and signed by the Wyeth medical monitor and the WHO Clinical Coordinator. A protocol amendment may be implemented at this stage. Where applicable, the amendment must receive a favorable opinion from the GFDB/IEC and WHO ERC.

A protocol change intended to eliminate an apparent immediate hazard to subjects may be implemented immediately, but the change must then be documented in an amendment and submitted to the Ghana Food and Drugs Board (IEC) and WHO ERC within 5 working days. The protocol amendment will be sent in the same time period to the Wyeth medical monitor and the WHO project manager.

25.10 Change in Investigator

If the investigator retires, relocates, or otherwise withdraws from conducting a study, the responsibility for maintaining records may be transferred to the sponsors, GFDB/IEC, or another investigator. The

sponsors must be notified of and agree to the change. The Ghana Food and Drugs Board will be notified with the appropriate documentation.

25.11 Termination of the Study

25.11.1 Termination by the Sponsors

Either sponsor may terminate the study at any time for any of the following reasons:

1. Failure to enroll subjects.
2. Protocol violations.
3. Inaccurate or incomplete data.
4. Unsafe or unethical practices.
5. Questionable safety of the test article.
6. Suspected lack of efficacy of the test article.
7. Administrative decision.

25.11.2 Termination by the Investigator

If the investigator terminates the study prematurely, the investigator does the following:

1. Returns all test articles, CRFs, and related study materials to the sponsors.
2. Provides the GFDB and the sponsors with a written statement describing why the study was terminated prematurely. Prompt compliance with this requirement is essential so that the sponsors may comply with its regulatory obligations.

25.12 Final Study Report

The investigator should complete a report notifying the GFDB of the conclusion of the clinical study. This report should be made within 3 months of completion or termination of the study.

The final report sent to the GFDB should also be sent to the sponsors and, along with the completed CRFs, constitutes the final summary to the sponsors, thereby fulfilling the investigator's regulatory responsibility.

25.13 Confidentiality

All unpublished information that the sponsors give to the investigator shall be kept confidential and shall not be published or disclosed to a third party without the prior written consent of the sponsors.

When the sponsors generate reports for presentations to regulatory agencies, the investigator may be asked to endorse the final report. The endorsement is required by some regulatory agencies.

The investigator shall not make a patent application based on the results of this study and shall not assist any third party in making such an application without the written authorization of the sponsors unless otherwise specified in the CSA.

25.14 Records Retention

The investigator shall retain and preserve one copy of all data generated in the course of the study, specifically including but not limited to those documents defined by GCP as essential documents, for the longer of:

i. 2 years after the last marketing authorization for the study drug has been approved or the sponsors have discontinued its research with respect to such drug or

ii. Such longer period as required by applicable global regulatory requirements. At the end of such period, the investigator shall notify in writing the sponsors of its intent to destroy all such material. The sponsors shall have 30 days to respond to the investigator’s notice, and the sponsors shall have a further opportunity to retain such materials at the sponsor’s expense.

25.15 Publications

If on completion of the study the data warrant publication, the investigator may publish the results in recognized (refereed) scientific journals subject to the provisions of the CSA. Unless otherwise specified in the CSA, the following process shall occur:

The principal investigator shall submit reports, abstracts, manuscripts and or other presentation materials to the sponsors for review prior to submission for publication or presentation. The sponsors shall have 60 calendar days to respond with any requested revisions, including without limitation, the deletion of

confidential information. The principal investigator shall act in good faith upon such requested revisions, except the principal investigator shall delete any confidential information from such proposed publication. The principal investigator shall delay submission of such publication or presentation materials for up to an additional 90 calendar days in order to have a patent application(s) filed.

25.16 Subject Injury

WYETH shall indemnify WHO, the Principal Investigator and his/her institution, and pay, for the medical expenses of reasonable and necessary medical treatment if a Study patient is injured during the Study and the injury is a result of the effects of the Study Drug, to the extent that the medical expenses are not covered by the Study patient's medical insurance, a government program or any other responsible third party, and provided that the Study patient has reasonably followed the instructions of the Principal Investigator, and the WHO, Principal Investigator and each Other Clinical Investigator involved in administering the Study Drug to the patient or otherwise providing medical care to such patient have reasonably complied with relevant obligations in this Agreement, the Protocol and all applicable laws and regulations.

WHO shall procure and maintain liability insurance with reputable and financially secure insurance carriers to cover its obligations that may arise in connection with any injury to any patient for which, pursuant to the second paragraph of Section 13.1, WYETH shall not be liable.

26. SPONSOR OBLIGATIONS IN CASE OF PREMATURE TRIAL TERMINATION

If the sponsors should jointly decide to end the clinical trial prematurely, they will notify the investigator, the ethics committees and the relevant authorities of this decision and the reasons for the termination.

27. REFERENCES

[1] Taylor HR, Trpis M, Cupp EW, et at. Ivermectin prophylaxis against experimental Onchocerca volvulus infection in chimpanzees. Am J Trop Med Hyg. 1988;39(1):86-90.

[2] Chavasse DC, Post RJ, Davies, JB, Whitworth JA. Absence of sperm from the seminal receptacle of female Onchocerca volvulus following multiple doses of ivermectin. Trop Med Parasitol. 1993;44(3):155-158.

[3] Duke BO, Zea-Flores G, Castro J, Cupp E, Munoz B. Effects of multiple monthly doses of ivermectin on adult Onchocerca volvulus. Am J Trop Med Hyg. 1990;43(6):657-664.

[4] Fisher MH. Structure-activity relationships of the avermectins and milbemycins. American Chemical Society Symposium Series, Phytochemicals for Pest Control. 1997;17:220-238.

[5] Ingle D, Wood IB. Princeton, NJ, Wyeth (Fort Dodge) Report, 1990.

[6] Sangster NC, Bannan SC, Weiss AS, Nulf SC. Haemonchus contortus: Sequence heterogeneity of internucleotide binding domains from P-glycoproteins and an association with avermectin/milbemycin resistance. Exp Parasitol. 1999; 91:250-257.

[7] Besier RB, Lyon J, Kieran PJ. The effect of moxidectin against benzimidazole- and levamisole-resistant nematodes of sheep in Western Australia. Aust Vet J.1993;70(11):422-423.

[8] Oosthuizen WTJ, Erasmus JB. Efficacy of moxidectin against a strain of Haemonchus contortus resistant to ivermectin, a benzimidazole and a salicylanilide. J S Afr Vet Assoc. 1993;64(1):9-12.

[9] Cole LM, Casida JE. GABA-gated chloride channel: Binding site for 4-ethynyl-4-n-[2,3-3H2] propylbicyclo-ortho benzoate ([3H]EBOB) in vertebrate brain and insect head. Pesticide Biochemistry and Physiology. 1992;44: 1-8.

[10] Townson S. TDR project 960089 progress report, 23 December 1998.

[11] Tagboto SK, Townson S. Onchocerca volvulus and O. lienalis: The microfilaricidal activity of moxidectin compared with that of ivermectin in vitro and in vivo. Ann Trop Med Parasitol. 1996;90(5):497-505.

[12] Trees A. TDR project 980383 progress report, January 2000.

[13] Anonymous. TDR progress report project 980386 & 980389, January 2000.

[14] McCall JW, Dzimianski MT, Supakomdej P, Jun JJ. The antifilarial activity of moxidectin against patent infections of Brugia Pahangi in dogs. Abstract 707. Am J Trop MedHyg. (1999);61 (Suppl 444).

[15] MECTIZAN Package Insert, 5 July 1999.

[16] Awadzi, K, Dadzie KY, De Sole G, Remme J. Reactions to ivermectin treatment in onchocerciasis patients. Acta Leiden. 1990;59: 93-199.

[17] Awadzi K: The chemotherapy of onchocerciasis II. Quantitation of the clinical reaction to microfilaricides. Ann Trop Med Parasitol. 1980;74:189-197.

[18] Francis H, Awadzi K, Ottesen EA. The Mazzotti reaction following treatment of onchocerciasis with diethylcarbamazine: clinical severity as a function of infection intensity. Am J Trop Med Hyg. 1985;34:529-536.

[19] Baraka OZ, Mahmoud BM, Ali MM, Ali MH, el Sheikh EA, Homeida MM, Mackenzie CD, Williams JF. Ivermectin treatment in severe asymmetric reactive onchodermatitis (sowda) in Sudan. Trans R Soc Trop Med Hyg. 1995;89:312-315.

[20] Awadzi K, Opoku NO, Addy ET, Quartey BT: The chemotherapy of onchocerciasis XIX: the clinical and laboratory tolerance of high dose ivermectin. Trop Med Parasitol. 1995;46:131-137.

[21] Bryceson AD, Warrell DA, Pope HM: Dangerous reactions to treatment of onchocerciasis with diethylcarbamazine. Br Med J. 1977;1:742-744.

[22] Awadzi K, Gilles HM: Diethylcarbamazine in the treatment of patients with onchocerciasis. Br J Clin Pharmacol. 1992;34: 281-288.

[23] Awadzi K: The chemotherapy of onchocerciasis II: Quantitation of the clinical reaction to microfilaricides. Ann Trop Med Parasitol. 1980;74:189-197.

[24] Hero M, Bird A C, Awadzi K: Quantification of the ocular reactions to microfilaricides in the chemotherapy of onchocerciasis. Eye 1992;6(Pt 1):93-96.

[25] Cancer Therapy Evaluation Program; Common Toxicity Criteria, Version 2.0, DCTD, NCI, DHHS, April 1999.

ATTACHMENTS

ATTACHMENT 1. RESPONSIBILITIES OF THE UNBLINDED THIRD PARTY PHARMACIST

1. The unblinded third party pharmacist signs a statement (Wyeth form 6732C) that assures Wyeth Research and the regulatory authorities that he or she understands his or her obligations and responsibilities as specified in the protocol, ie, correct preparation of the study medication and the manner in which it is to be administered. The original signed statement is forwarded to the Wyeth Research clinical scientist. Copies of this statement are retained by the unblinded third party pharmacist and the investigator.

2. The unblinded third party pharmacist is responsible for all clinical materials provided by Wyeth Research, such as the study medication, randomization chart, drug accountability and dispensing forms, and any other supplies.

3. The unblinded third party pharmacist will be responsible for maintaining the confidentiality and security of the randomization chart.

4. The unblinded third party pharmacist will

   a. Identify the correct treatment for the subject based on the randomization chart generated by Wyeth Research and prepare the doses according to the detailed instructions provided (see Attachment 4).
   b. Adhere to good preparation practices, eg, clear an appropriate space; minimize distractions; open only 1 medication container at a time.
   c. Prepare medication that is appropriately labeled with the subject or subject number and initials, and the randomization number (include other label requirements specific to the protocol).
   d. Safely and efficiently deliver the prepared drug directly to the investigator or his or her designee.
   e. Complete all drug accountability and dispensing information on the drug accountability and dispensing records, including any amount returned by the subject or investigator. Quantities that are wasted or discarded must also be recorded.
   f. Return the randomization chart to the Wyeth Research Clinical Pharmacy Section.
   g. Not participate in the evaluation of any subject or subject.

5. The unblinded third party pharmacist may not administer the medication to the subjects. Contact between the unblinded third party pharmacist and subjects should be kept to a minimum.

ATTACHMENT 2. COLLECTION, PREPARATION, AND LABELING OF PLASMA SAMPLES

1. **Plasma (for drug concentration data):**
   - Collect 6 mL of blood into a single evacuated blood tube containing lithium heparin.
   - Invert the tubes gently 4 or 5 times (avoid shaking).
   - Blood must be placed on ice immediately, and centrifuged (at 1500g for 10 minutes) in a refrigerated centrifuge within 15 minutes following collection.
   - Aliquot into **two** watertight, labeled polypropylene containers. Distribute plasma evenly between containers
   - Store frozen in an upright position at about -70°C until shipped.

2. Pre-printed labels with a bar code will be provided by Wyeth which will identify each sample with the analyte to be assayed, protocol number, investigator name, subject number, matrix (plasma) and protocol day and time of sample collection. Hand writing information on the labels is not required, but in the event that it should be necessary to hand write information, only indelible ink should be used. Additional information handwritten on a computer-printed label **must not obscure the bar code**.

3. Apply preprinted labels to tubes at room temperature at least 2 hours before refrigerating or freezing. Adequate adhesion cannot be guaranteed if the labels are applied to cold tubes.

4. Apply the labels along the length of the tube with the colored bar at the top. Do not spiral or wrap around the circumference of the tube as this interferes with reading the bar code.

5. Secure the labels by wrapping with clear tape.

ALL SAMPLES MUST BE KEPT FROZEN AT APPROXIMATELY -70°C AFTER COLLECTION AND UNTIL SHIPMENT IS INITIATED.

ATTACHMENT 3. INSTRUCTIONS FOR SHIPPING FROZEN SAMPLES

1. The shipping list is included as the white page of the PK shipping worksheet from the case report form. When sending the second set of samples, a photocopy of the remaining NCR PK shipping worksheet from the case report form should be utilized as the shipping list. If a separate shipping list is used, be sure to include the same information collected on the tube label.

2. Place the watertight plastic transfer tubes from each subject in watertight plastic bags, preferably 1 to 10 tubes per bag. Place the plastic bags in an insulated shipping container with the appropriate completed case report form shipping page(s). Fill the container with sufficient dry ice to maintain the samples in the frozen state for at least 48 hours (72 hours for international shipments), and seal with tape.

3. Label the containers as clinical specimens. If further information is needed, contact the WHO monitor.

4. Ship the sealed containers in sturdy outside packaging in accordance with the shipper's guidelines.

5. Make shipments as early induring the week as possible - not later than Wednesday noonon days that do not delay shipment duration (to be arranged with carrier). They never should be made on a Friday or two days before a holiday. Consult your analytical laboratory representative for the dates of site-specific holidays.

6. Choose the carrier in collaboration with the WHO monitor.

7. Be sure that the carrier will pick up the container from your office. If they do not, however, a member of your staff should deliver the containers to the carrier's office.

8. It is extremely important that the airway bill prepared by the shipping agent contain the exact instructions and address given on the package label. The fact that the shipment contains a frozen biological packed in dry ice should be noted on the airway bill.

9. At the time shipment is initiated, notify the recipients (see instruction 11) and the Wyeth clinical scientist and/or clinical trial manager (see instruction 12) by fax and/or (if appropriate) e-mail with the following information:

   - Carrier, Airway bill number, Expected time of arrival, Number of pieces in the shipment, Protocol number, and your name and fax number.

   - Note: The e-mail notification described here serves as an adjunct to the fax notification but does not replace it.

10. Ship plasma samples for moxidectin to:

Dr. Larry Fleckenstein
College of Pharmacy
University of Iowa
S4 33 PHAR
115 South Grand Avenue
Iowa City, IA 52242

Phone: +1 (319)-335-8804
Fax: +1 (319)-353-5646

11. The recipient will acknowledge receipt of the sample shipment by fax and (if appropriate) e-mail within 24 hours to the responsible party at the clinical site, with a copy to the clinical scientist. Any irregularities in the shipment received will be promptly and duly noted by the recipient in the sample receipt notification.
12. Similarly, the status of delayed or missing shipments will be communicated within 24 hours of the expected time of arrival. Such issues will be resolved by follow-up communication between the responsible clinical site personnel and the recipient.
13. If you have any questions regarding shipment, please do not hesitate to call:

Russ Orrico
Senior Clinical Scientist
Wyeth Research-US
Phone: +1 (484)-865-5587
Fax: +1 (484)-865-0071
E-mail: orricor@wyeth.com

ATTACHMENT 4. INSTRUCTIONS FOR PREPARATION OF STUDY DRUG

1. The drugs are encapsulated moxidectin 2 mg tablets (TIC), encapsulated 3 mg ivermectin tablets (TIC) or matching placebo capsules. Moxidectin 2 mg TIC is the only strength available. Therefore, 1 TIC is required for the 2 mg dose; 2 TICs are required for the 4 mg dose; 4 TICs are required for the 8 mg dose.

2. Three dose regimens of moxidectin (2 mg, 4 mg, 8 mg) will be studied at 3 levels of intensity of infection (mild, moderate, severe). The weight-based ivermectin dosing schedule approved for the treatment of onchocerciasis will be used. The first 2 cohorts at each moxidectin dose level will comprise 16 subjects while the third cohort will comprise 32 subjects enrolled in 2 successive groups of 16. Thus, one hundred and ninety-two (192) subjects including both sexes are expected to enroll in this study; and will be admitted in 12 groups.

3. Subjects will be assigned a number on admission (subject number). The unblinded third party pharmacist will be supplied with 12 randomization schedules (one for each of the three severity levels within each of the three treatment strata and 3 additional schedules for the most severely infected subjects). Each randomization schedule will contain the 16 randomization numbers, as well as the corresponding treatment assignments. Treatment groups will be blocked in groups of 4, with a 3 to 1 ratio of moxidectin to ivermectin. Where there are less than 16 subjects enrolled, the leftover randomization numbers will be ignored and a new randomization schedule will be used when a new cohort is started.

4. The PI or clinical assessor will provide the unblinded third party pharmacist with a list of subjects who qualify for enrollment, and will identify the cohort for which drug is to be prepared by the dose group and the severity of infection. For the severely infected cohorts, the PI will add the information whether this is the first or the second 16 subject group to be treated. For each subject, the name, initials, age, sex, weight and subject number will be stated. The unblinded third party pharmacist will assign the randomization numbers at the time of preparing the study medication. Each randomization number will be matched with the corresponding subject number and, WITH THE EXCLUSION OF ALL PREPARATION INFORMATION, be provided to the PI to be placed onto the Case Report Form. At the end of the study, they will be used to identify the treatment group assigned to the subject.

5. The drugs will be prepared the night before administration (day –1). Moxidectin/ivermectin/placebo capsules will be stored locked at room temperature up to 30˚C.

Materials

Moxidectin capsules (containing 2 mg tablets)
Ivermectin capsules (containing 3 mg tablets)
Matching placebo capsules
Plastic cups (about 600 mL) with lids
Ordinary tap water
Markers
Sterile Disposable gloves
Refrigerator with key
Metal cabinet with key
Teaspoon (to dispense the tablets)
Dispensing tray
Small plastic envelops for dispensing tablets
Medium sized envelops (opaque)
Medium size trays-2
Self adhesive labels
Disposal Bin

Procedure

A. Day –1.

1. Treatment Allocation (Unblinded Third Party Pharmacist)

Using the randomization schedule supplied by Wyeth Research and the subject list provided, allocate each subject to the appropriate treatment regimen.

- Select the first male subject to be enrolled from the top of the randomization list that corresponds to the subject's assigned stratum. Similarly, select the first female from the bottom of the randomization list that corresponds to the subject's assigned stratum.

- Assign the second male subject to the next available treatment from the top of the randomization list that corresponds to the subject's assigned stratum. Assign the second female subject to the next available treatment from the bottom of the randomization list that corresponds to the subject's assigned stratum. Continue in this manner until the entire cohort has been assigned to the relevant treatment group.

- Complete the treatment allocation on the drug accountability form.

THIS FORM MUST NOT BE AVAILABLE TO ANYONE EXCEPT THE UNBLINDED MONITOR UNTIL THE END OF THE STUDY.

2. Preparing and Dispensing the Study Drugs (Unblinded Third Party Pharmacist)

- Label 16 medium size envelopes (one for each subject) with the subject's identification i.e. name, subject number, randomization number, age, sex, and date of drug administration.
- Wear the disposable gloves.
- Using the teaspoon and dispensing tray, dispense the appropriate number of moxidectin, placebo and/or ivermectin capsules for each subject into the small plastic envelopes and seal them. There should be a total number of 4 capsules in each envelope as per the tables below:

**Cohort**	**2 mg**					
Treatment Assignment	Moxidectin			Ivermectin		
Capsule	Moxidectin Capsule	Placebo Capsule	Ivermectin Capsule	Moxidectin Capsule	Placebo Capsule	Ivermectin Capsule
Subject Weight	Number of Capsules					
26-44 kg	1	3	0	0	2	2
45-64 kg	1	3	0	0	1	3
65-84 kg	1	3	0	0	0	4

**Cohort**	**4 mg**					
Treatment Assignment	Moxidectin			Ivermectin		
Capsule	Moxidectin Capsule	Placebo Capsule	Ivermectin Capsule	Moxidectin Capsule	Placebo Capsule	Ivermectin Capsule
Subject Weight	Number of Capsules					
26-44 kg	2	2	0	0	2	2
45-64 kg	2	2	0	0	1	3
65-84 kg	2	2	0	0	0	4

**Cohort**	**8 mg**					
Treatment Assignment	Moxidectin			Ivermectin		
Capsule	Moxidectin Capsule	Placebo Capsule	Ivermectin Capsule	Moxidectin Capsule	Placebo Capsule	Ivermectin Capsule
Subject Weight	Number of Capsules					
26-44 kg	4	0	0	0	2	2
45-64 kg	4	0	0	0	1	3
65-84 kg	4	0	0	0	0	4

- Put the small envelopes containing the tablets capsules into the corresponding labeled medium size envelopes. Seal the envelopes. Lock them up in a small cabinet at ≤30°C and keep the key.

- Dispose of all used disposable items (e.g. gloves, facemask) into a bin provided for that purpose.

- Prepare a list with the name, initials, age, sex, weight, subject number, and randomization number.

B. Day 1.

1. Morning of Drug Administration (Unblinded Third Party Pharmacist)

- Remove the medium size envelopes containing the capsules from the cabinet. Re-check all the labeling and arrange them according to subject numbers on the tray.
- Hand over the tray to the clinical staff in-charge of administering the drugs. Ensure he/she acknowledges receipt.
- Hand over the list with the name, initials, age, sex, weight, subject number and randomization number to the PI.
- After administration of the drugs, collect all the empty drug envelopes from the personnel that has dispensed the drugs. Check that all have been handed over.
- Complete the Wyeth-provided label that lists the 16 subject numbers and their corresponding randomization numbers. This adhesive label will then be affixed to the outside of the opaque envelope.~~Write the name, subject number, randomization number, age, sex, weight, treatment regimen and date for each subject on a piece of paper and insert into the corresponding opaque envelope. Ensure that each tightly sealed envelope has been locked in a cabinet by the hospital pharmacist only to be opened if required for emergency unblinding.~~
- Record the drugs dispensed on the “Drug Stocks, Distribution and Accountability” Form.
- Lock up all documents in the cabinet for drug accounting purposes.

2. Administration of Study Drugs (Clinical Staff)

The study drugs will be administered after an overnight fast. Breakfast will be delayed for about 2 hours.

- Retrieve the drug envelopes for the subject from the tray. Put them in a kidney dish.

- Half fill the 600 mL plastic cups with ordinary tap water.
- With the assistance of one or more nurses, check the labeling on the drug envelopes containing the capsules. Ensure that the subject name and number correspond with those on the board above the head of the bed. Check the date.
- Call out the name on the label and listen to the subject verifying that he/she is the named individual.
- Open the drug envelope and give the capsules and the plastic cup containing about 300 mL of tap water to the subject.
- Observe the subject swallow the capsules and inspect the mouth to ensure compliance.
- Record the date and time in the source documentation that the capsules were swallowedb.

ATTACHMENT 5. ~~RECOMMENDATIONS FOR GRADING OF OCULAR EXAMINATIONS~~OCRC CTC VERSION 2~~1~~.0

	Grade				
**Toxicity**	**0**	**1**	**2**	**3**	**4**
MAZZOTTI TOXICITY CRITERIA (MTC) SYSTEMIC (1)					
Itching	None	Mild	Moderate or 'Severe' but with only occasional scratching	'Severe' and with fairly vigorous scratching "Windmill Effect"	'Severe' and with restlessness, agitation, loss of epithelium or prolonged vigorous scratching
Note: 'Severe' is patient's perception of grade of discomfort. "Windmill Effect" = arms in fairly continuous motion with scratching					
Headache	None	Mild	Moderate or 'Severe' but patient comfortable	'Severe' and with obvious distress	Unbearable
Note: 'Severe' is patient's perception of grade of discomfort					
Joint pain (arthralgia)	None	Mild	Moderate or 'Severe' but without change in gait or function	'Severe' and with a definite limp or change in function due to joint pain	'Severe' and with marked interference with motion or function (commonly "Pillar of Salt" Effect)
Note: 'Severe' is patient's perception of grade of discomfort. "Pillar of salt Effect" = rooted to the spot and unable to walk due to severe pain					
Muscle pain (myalgia)	None	Mild	Moderate or 'Severe' but without change in gait or function	'Severe' and with a definite limp or change in function due to muscle pain	'Severe' and with marked interference with motion or function (commonly "Pillar of Salt" Effect)
Note: 'Severe' is patient's perception of grade of discomfort. "Pillar of salt Effect" = rooted to the spot and unable to walk due to severe pain					
Gland pain	None	Mild	Moderate or 'Severe' but without change in gait or function	'Severe' and with a definite limp or change in function due to gland pain ("Hydrocele Gait" may be present)	'Severe' and with marked interference with motion or function (commonly "Pillar of Salt" Effect; "Knee Elbow Position")
Note: 'Severe' is patient's perception of grade of discomfort. "Pillar of salt Effect" = rooted to the spot and unable to walk due to severe pain "Hydrocele Gait" = Walking on a broad base with trunk slightly flexed.					
Gland tenderness	None	Very firm pressure needed to elicit pain	Moderate pressure elicits pain	Very light touch elicits severe pain	Patient refuses palpation on account of severe pain
Note: Assessment carried out by Medical Officer					
Rash	None	< ⅓ body surface	⅓-< ⅔ body surface	≥⅔ body surface	-
Note: Assessment carried out by Medical Officer					
MTC SYSTEMIC (2)					
Temperature increase	<38°C	38.0 - 39.0°C	39.1 - 40.0°C	> 40.0°C for < 24hrs	> 40.0°C > 24hrs
Increase in Pulse Rate in beats per minute	0 -<20	>20-<36	≥36-<52	≥52-<68	≥68
Note: Readings taken lying as well as after standing for 2 minutes					
Fall in Mean Arterial Pressure	0-<20	>20-<25	≥25-<30	≥30-<35	≥35 or CNS*
Note: Readings are taken lying as well as after standing for 2 minutes. * CNS means Could Not Stand long enough for pressure to be taken due to severe postural hypotension					
Increase in Respiratory Rate per minute	0-<6	≥6-<12	≥12-<18	≥18-<24	≥24
Note: Contributes little to overall score					
MTC OTHER					
Lymphatics	Normal	Mild lymphedema	Moderate lymphedema requiring compression; lymphocyst	Severe lymphedema limiting function; lymphocyst requiring surgery	Severe lymphedema limiting function with ulceration
Note: An acute brawny edema of one or more limbs a manifestation of the Mazzotti reaction					
Facial swelling	None	Mild swelling	Moderate swelling	Severe with eyes completely shut	-

		Grade			
**Toxicity**	**0**	**1**	**2**	**3**	**4**
Note: A fairly common manifestation of the Mazzotti reaction					
Neuropathy-sensory	Normal	Loss of deep tendon reflexes or paresthesia (including tingling) but not interfering with function	Objective sensory loss or paresthesia (including tingling), interfering with function, but not interfering with activities of daily living	Sensory loss or paresthesia interfering with activities of daily living	Permanent sensory loss that interferes with function
Note: Peripheral Sensory Phenomena (PSP) can be a manifestation of the Mazzotti reaction					
Rigors, chills	None	Mild, requiring symptomatic treatment (e.g., blanket) or non-narcotic medication	Severe and/or prolonged, requiring narcotic medication	not responsive to narcotic medication	-
Fatigue (lethargy, malaise, asthenia)	None	Increased fatigue over baseline, but not altering normal activities	Moderate (e.g., decrease in performance status by 1 ECOG level <u>or</u> 20% Karnofsky or *Lansky*) <u>or</u> causing difficulty performing some activities	Severe (e.g., decrease in performance status by ≥2 ECOG levels <u>or</u> 40% Karnofsky or *Lansky*) <u>or</u> loss of ability to perform some activities	Bedridden or disabling
Note: Performance status scales presented later					
OTHER SYSTEMIC TOXICITY					
Anorexia	None	Loss of appetite	oral intake significantly decreased	Requiring IV fluids	Requiring feeding tube or parenteral nutrition
Nausea	None	Able to eat	oral intake significantly decreased	no significant intake, requiring IV fluids	-
'Bitter' mouth	None	Able to eat	oral intake significantly decreased	no significant intake, requiring IV fluids	-
Vomiting	None	1 episode in 24 hours over pretreatment	2-5 episodes in 24 hours over pretreatment	≥6 episodes in 24 hours over pretreatment; or need for IV fluids	Requiring parenteral nutrition; or physiologic consequences requiring intensive care; hemodynamic collapse
Diarrhea	None	Increase of < 4 stools/day over pre-treatment	Increase of 4-6 stools/day, or nocturnal stools	Increase of ≥7 stools/day or incontinence; or need for parenteral support for dehydration	Physiologic consequence requiring intensive care; or hemodynamic collapse
Abdominal pain or cramping	None	Mild pain not interfering with function	Moderate pain: pain or analgesics interfering with function, but not interfering with activities of daily living	Severe pain: pain or analgesics severely interfering with activities of daily living	Disabling
Cough	Absent	Mild, relieved by non-prescription medication	Requiring narcotic antitussive	Severe cough or coughing spasms, poorly controlled or unresponsive to treatment	-
Chest pain (non-cardiac and non-pleuritic)	None	Mild pain not interfering with function	Moderate pain: pain or analgesics interfering with function, but not interfering with activities of daily living	Severe pain: pain or analgesics severely interfering with activities of daily living	Disabling
Dyspnea (shortness of breath)	Normal	-	Dyspnea on exertion	Dyspnea at normal level of activity	dyspnea at rest or requiring ventilator support
Palpitations	None	Present	-	-	-
Note: Grade palpitations <u>only</u> in the absence of a documented arrhythmia.					

Grade					
**Toxicity**	**0**	**1**	**2**	**3**	**4**
Waistpain/Backache	None	Mild pain not interfering with function	Moderate pain: pain or analgesics interfering with function, but not interfering with activities of daily living	Severe pain: pain or analgesics severely interfering with activities of daily living	Disabling
Neckpain	None	Mild pain not interfering with function	Moderate pain: pain or analgesics interfering with function, but not interfering with activities of daily living	Severe pain: pain or analgesics severely interfering with activities of daily living	Disabling
Bodily pain/aches	None	Mild pain not interfering with function	Moderate pain: pain or analgesics interfering with function, but not interfering with activities of daily living	Severe pain: pain or analgesics severely interfering with activities of daily living	Disabling
Dizziness/lightheadedness	None	Not interfering with function	Interfering with function, but not interfering with activities of daily living	Interfering with activities of daily living	bedridden or disabling
Note: Grade only if unassociated with hypotension					
Constipation	None	Requiring stool softener or dietary modification or increased mobility	Requiring laxatives	Obstipation requiring manual evacuation or enema	obstruction or toxic megacolon
Dyspepsia/heartburn	None	Mild	Moderate	Severe	-
Insomnia	Normal	Occasional difficulty sleeping not interfering with function	Difficulty sleeping interfering with function, but not interfering with activities of daily living	Frequent difficulty sleeping, interfering with activities of daily living	-
Note: This toxicity is graded when insomnia is related to treatment. (Sleepless for no reason). If pain or other symptoms interfere with sleep do NOT grade as insomnia.					
Bone pain	None	Mild pain not interfering with function	Moderate pain: pain or analgesics interfering with function, but not interfering with activities of daily living	Severe pain: pain or analgesics severely interfering with activities of daily living	Disabling
Catarrh	Absent	Present	-	-	-
Earache (otalgia)	None	Mild pain not interfering with function	Moderate pain: pain or analgesics interfering with function, but not interfering with activities of daily living	Severe pain: pain or analgesics severely interfering with activities of daily living	Disabling
Note: It is essential to determine that otalgia is not present pretreatment					
Toothache	None	Mild pain not interfering with function	Moderate pain: pain or analgesics interfering with function, but not interfering with activities of daily living	Severe pain: pain or analgesics severely interfering with activities of daily living	Disabling
Note: It is essential to determine that toothache is not present pretreatment					
Weight gain	< 5%	5 - <10%	10 - <20%	≥ 20%	-
Weight loss	< 5%	5 - <10%	10 - <20%	≥20%	-
Erectile impotence	Normal	Mild (erections impaired but satisfactory)	Moderate (erections impaired, unsatisfactory for intercourse)	no erections	-
Libido	Normal	Decrease in interest	severe loss of interest	-	-
Dysmenorrhea	None	Mild pain not interfering with function	Moderate pain: pain or analgesics interfering with function, but not interfering with activities of daily living	Severe pain: pain or analgesics severely interfering with activities of daily living	Disabling

Toxicity	Grade 0	1	2	3	4
Irregular menses (change from baseline)	Normal	Occasionally irregular or lengthened interval, but continuing menstrual cycles	very irregular, but continuing menstrual cycles	Persistent amenorrhea	-
**MTC OCULAR**					
Ocular discomfort	Normal	mild: not interfering with function	Moderate: interfering with function, but not interfering with activities of daily living	Interfering with activities of daily living	-
Ocular itching	Normal	mild: not interfering with function	Moderate: interfering with function, but not interfering with activities of daily living	Interfering with activities of daily living	-
Tearing (watery eyes)	None	mild: not interfering with function	Moderate: interfering with function, but not interfering with activities of daily living	Interfering with activities of daily living	-
Vision- photophobia	Normal	-	Symptomatic and interfering with function, but not interfering with activities of daily living	Symptomatic and interfering with activities of daily living	-
Visual acuity	Unchanged or 6/4-6/6	Loss of 1 line	Loss of 2 lines	Loss of 3 lines	Loss of >3 lines
Visual fields					
IV/4e kinetic	None	1-12	13-24	25-48	49-72
I4/e kinetic	None	1-12	13-24	25-36	37-60
I/2e	None	1-12	13-20	21-28	29-36
I2/e	None	3-12	13-20	21-35	36-52
Note: Based on the number of missed targets					
Anterior Segment inflammation					
Conjunctivitis	None	-	Hyperaemia	-	Chemosis
Limbitis-vascular changes	None	-	Dilated capillaries	-	Limbal oedema
Limbitis-globular infiltrates	None	1-5	6-10	11-20	>20
Corneal punctate opacities	None	1-5	6-10	11-20	>20
Anterior uveitis					
No of cells/field *	None	1-10	11-20	21-40	>40
Flare**	None	Seen with no filter	Seen with filter 1	Seen with filter 2	Plasmoid aqueous
Note: * Light beam at 45 degrees; slit 2mm high by 0.2mm wide. ** Light beam at 45 degrees; slit 2mm high by 0.1mm wide. Filters 1 and 2 are neutral density filters in-built into the optical column of the slit lamp					
Posterior segment					
Optic neuritis (colour film)					
Hyperaemia	None	Sectorial	Overall	-	-
Swelling	None	-	-	Sectorial	Overall
Angiographic leakage					
Within disc margin	None	Sectorial	Overall	-	-
Beyond disc margin	None	-	-	Sectorial	Overall
Optic atrophy					
Colour film	None	Linear nerve fibre loss	Sectorial atrophy	Overall atrophy	Atrophy with pigment
Red free	None	Linear nerve fibre loss	Sectorial fibre loss	Total loss	Total loss with pigment
Pigment Epithelial Atrophy					
Distribution	None	Temporal to macula	More than temporal	Continuous round macula	Whole of macula
Intensity	None	RPE mottling only	RPE mottling with <50% atrophy	RPE mottling with ≥50% atrophy	RPE mottling with hypertrophy
Other onchocercal lesions					
Cotton wool spots	Absent	Present	-	-	-
Vasculitis	Absent	Present	-	-	-
Haemorrhage	Absent	Present	-	-	-

		Grade			
**Toxicity**	**0**	**1**	**2**	**3**	**4**
Note: Once an event has occurred the score is retained at subsequent visits whether the lesion persists or not.					
OTHER OCULAR TOXICITY					
Ocular discharge	None	Present	-	-	-
Vision- blurred vision	Normal	-	Symptomatic and interfering with function, but not interfering with activities of daily living	Symptomatic and interfering with activities of daily living	-
Vision- flashing lights/floaters	Normal	Mild, not interfering with function	Symptomatic and interfering with function, but not interfering with activities of daily living	Symptomatic and interfering with activities of daily living	-
Vision- night blindness (nyctalopia)	Normal	Present but asymptomatic	Symptomatic and interfering with function, but not interfering with activities of daily living	Symptomatic and interfering with activities of daily living	-
ALLERGIC REACTION commonly to FLUORESCEIN					
Allergic reaction/ hypersensitivity (including drug fever)	None	Transient rash, drug fever < 38°C (<100.4°F)	urticaria, drug fever ≥ 38°C (≥100.4°F), and/or asymptomatic bronchospasm	Symptomatic bronchospasm, requiring parenteral medication(s), with or without urticaria; allergy-related edema/angioedema	Anaphylaxis
Sneezing	Absent	Present	-	-	-
Dry cough, no bronchospasm	Absent	Present	-	-	-
Urticaria (hives, welts, wheals)	None	Requiring no medication	requiring PO or topical treatment or IV medication or steroids for <24 hours	requiring IV medication or steroids for ≥24 hours	-
Note: Also anorexia, nausea, vomiting , rigors, chills					
ELECTROCARDIOGRAPHY					
Conduction abnormality/ Atrioventricular heart block	None	Asymptomatic, not requiring treatment (e.g., Mobitz type I second-degree AV block, Wenckebach)	Symptomatic, but not requiring treatment	symptomatic and requiring treatment (e.g., Mobitz type II second-degree AV block, third-degree AV block)	Life-threatening (e.g., arrhythmia associated with CHF, hypotension, syncope, shock)
Nodal/junctional arrhythmia/dysrhythmia	None	Asymptomatic, not requiring treatment	symptomatic, but not requiring treatment	symptomatic and requiring treatment	Life-threatening (e.g., arrhythmia associated with CHF, hypotension, syncope, shock)
Palpitations<br>Note: Grade palpitations only in the absence of a documented arrhythmia.	None	Present	-	-	-
Prolonged QTc interval (QTc > 0.48 seconds)	None	Asymptomatic, not requiring treatment	symptomatic, but not requiring treatment	symptomatic and requiring treatment	Life-threatening (e.g., arrhythmia associated with CHF, hypotension, syncope, shock)
Sinus bradycardia	None	Asymptomatic, not requiring treatment	symptomatic, but not requiring treatment	symptomatic and requiring treatment	Life-threatening (e.g., arrhythmia associated with CHF, hypotension, syncope, shock)
Sinus tachycardia	None	Asymptomatic, not requiring treatment	symptomatic, but not requiring treatment	symptomatic and requiring treatment of underlying cause	-

			Grade		
**Toxicity**	**0**	**1**	**2**	**3**	**4**
Supraventricular arrhythmias (SVT/atrial fibrillation/ flutter)	None	Asymptomatic, not requiring treatment	symptomatic, but not requiring treatment	symptomatic and requiring treatment	Life-threatening (e.g., arrhythmia associated with CHF, hypotension, syncope, shock)
Vasovagal episode	None	-	present without loss of consciousness	present with loss of consciousness	-
Ventricular arrhythmia (PVCs/bigeminy/trigeminy/ ventricular tachycardia)	None	Asymptomatic, not requiring treatment	symptomatic, but not requiring treatment	symptomatic and requiring treatment	Life-threatening (e.g., arrhythmia associated with CHF, hypotension, syncope, shock)
Peaking of T wave	None	Mild-moderate	Marked	-	-
Cardiac- ischemia/infarction	None	Non-specific T-wave flattening or changes	Asymptomatic, ST- and T- wave changes suggesting ischemia	angina without evidence of infarction	acute myocardial infarction
HAEMATOLOGICAL					
G6PD	Normal	Partial defect	Total defect	-	-
Hemoglobin (Hgb)	WNL	10-<25% reduction	25-<50% reduction	50-<75% reduction	≥75%
Leukocytes (total WBC)	WNL	< LLN – 2.5x10^9 /L	1.5---< 2.5 x-10^9 /L	1.0 - < 1.5 x-10^9-/L	< 1.0 x 10^9 /L
	WNL	> 11.3-19.0 x 10^9 /L	>19.0-38.0 x 10^9 /L	>38.0-57.0 x 10^9 /L	> 57.0 x 10^9 /L
Note: Contributory factors include Mazotti reaction (leucocytosis), coincidental infection (exclude) drug effect					
Neutrophils/granulocytes (ANC/AGC)	WNL	≥1500 - <2000/mm^3	≥1000 - <1500/mm^3	≥500 - <1000/mm^3	< 500/mm^3
	WNL	>6080-12160/mm^3	>12160-24320/mm^3	>24320-36480/mm^3	>36480/mm^3
Note: Contributory factors include Mazotti reaction (neutrophilia), coincidental infection (exclude) drug effect					
Lymphocytes	WNL	<LLN – 1000/mm^3	≥500 - <1000/mm^3	<500/mm^3	-
	WNL	>636-5-12730/mm^3	>12730-25460/mm^3	>25460-38190/mm^3	>38190/mm^3
Note: Contributory factors include Mazotti reaction (initial lymphopenia, lymphocytosis), coincidental infection (exclude) drug effect					
Eosinophils	WNL	20-26/mm^3	≥14-19/mm^3	≥7-13/mm^3	<7/mm^3
	WNL	>1425-2850/mm^3	>2850-5700/mm^3	>5700-8550/mm^3	>8550/mm^3
Note: Contributory factors include Mazotti reaction (initial eosinopenia, eosinophilia), coincidental infection (exclude) drug effect					
Platelets	WNL	< LLN - <75.0 x 10^9 /L<br>< LLN – 75000/mm^3	≥50.0 - < 75.0 x 10^9 /L<br>≥50000 - < 75000/mm^3	≥10.0 - < 50.0 x 10^9 /L<br>≥10000 - < 50000/mm^3	< 10.0 x 10^9 /L<br>< 10000/mm^3
Hemolysis (e.g., immune hemolytic anemia, drug-related hemolysis, other)	None	Only laboratory evidence of hemolysis [e.g., direct antiglobulin test (DAT, Coombs') schistocytes]	Evidence of red cell destruction and ≥ 2gm decrease in hemoglobin, no transfusion	requiring transfusion and/or medical intervention (e.g., steroids)	catastrophic consequences of hemolysis (e.g., renal failure, hypotension, bronchospasm, emergency splenectomy)
BIOCHEMICAL					
Hyponatremia	WNL	<LLN – 130 mmol/L	-	120 - <130 mmol/L	<120 mmol/L
Hypernatremia	WNL	> ULN - 150 mmol/L	>150 - 155 mmol/L	>155 - 160 mmol/L	>160 mmol/L
Hypokalemia	WNL	<LLN - 3.0 mmol/L	-	2.5 - <3.0 mmol/L	<2.5 mmol/L
Hyperkalemia	WNL	> ULN - 5.5 mmol/L	> 5.5 - 6.0 mmol/L	> 6.0 - 7.0 mmol/L	> 7.0 mmol/L
Bicarbonate	WNL	< LLN - 16 mEq/dl	11 - 15 mEq/dl	8 - 10 mEq/dl	<8 mEq/dl
Hyperuricemia	WNL	> ULN - ≤ 590 micromol/L without physiologic consequences	-	>ULN - ≤ 590 micromol/L with physiologic consequences	> 590 micromol/L
Note: Also consider Renal failure, Creatinine, Potassium.					
Creatinine	WNL	> ULN - 1.5 x ULN	> 1.5 - 3.0 x ULN	> 3.0 - 6.0 x ULN	> 6.0 x ULN
SGPT (ALT) (serum glutamic pyruvic transaminase)	WNL	> ULN - 2.5 x ULN	> 2.5 - 5.0 x ULN	> 5.0 - 20.0 x ULN	> 20.0 x ULN
Note: Elevations occur as part of the Mazzotti reaction but rarely reach 5 x ULN and usually last for less than 30 days					
SGOT (AST) (serum glutamic oxaloacetic transaminase)	WNL	> ULN - 2.5 x ULN	> 2.5 - 5.0 x ULN	> 5.0 - 20.0 x ULN	> 20.0 x ULN

Toxicity	Grade 0	1	2	3	4
Note: Elevations occur as part of the Mazzotti reaction but rarely reach 5 x ULN and usually last for less than 30 days					
GGT (γ - Glutamyl transpeptidase)	WNL	> ULN - 2.5 x ULN	> 2.5 - 5.0 x ULN	> 5.0 - 20.0 x ULN	> 20.0 x ULN
Note: Elevations occur as part of the Mazzotti reaction but rarely reach 5 x ULN and usually last for less than 30 days					
LDH (Lactate dehydrogenase)	WNL	> ULN - 2.5 x ULN	> 2.5 - 5.0 x ULN	> 5.0 - 20.0 x ULN	> 20.0 x ULN
Note: Elevations occur as part of the Mazzotti reaction but rarely reach 5 x ULN and usually last for less than 30 days					
Alkaline phosphatase	WNL	> ULN - 2.5 x ULN	> 2.5 - 5.0 x ULN	> 5.0 - 20.0 x ULN	> 20.0 x ULN
Total Bilirubin	WNL	> ULN - 1.5 x ULN	> 1.5 - 3.0 x ULN	> 3.0 - 10.0 x ULN	> 10.0 x ULN
Hypoalbuminemia	WNL	<LLN - 30 g/L	≥20 - <30 g/L	<20 g/L	-
Hypoglycemia	WNL	<LLN - 3.0 mmol/L	2.2 - < 3.0 mmol/L	1.7 - < 2.2 mmol/L	< 1.7 mmol/L
Hyperglycemia	WNL	> ULN - 8.9 mmol/L	> 8.9 - 13.9 mmol/L	> 13.9 - 27.8 mmol/L	> 27.8 mmol/L or ketoacidosis
Hypercholesterolemia	WNL	> ULN - 7.75 mmol/L	> 7.75 - 10.34 mmol/L	>10.34 - 12.92 mmol/L	> 12.92 mmol/L
Hypertriglyceridemia	WNL	> ULN - 2.5 x ULN	> 2.5 - 5.0 x ULN	> 5.0 - 10 x ULN	> 10 x ULN
